# HILIC-IM-MS for Simultaneous Lipid and Metabolite Profiling of Bacteria

**DOI:** 10.1021/acsmeasuresciau.3c00051

**Published:** 2023-12-05

**Authors:** Jana M. Carpenter, Hannah M. Hynds, Kingsley Bimpeh, Kelly M. Hines

**Affiliations:** Department of Chemistry, University of Georgia, Athens, Georgia 30602, United States

**Keywords:** Ion mobility, mass spectrometry, lipidomics, metabolomics, microorganisms, bacteria

## Abstract

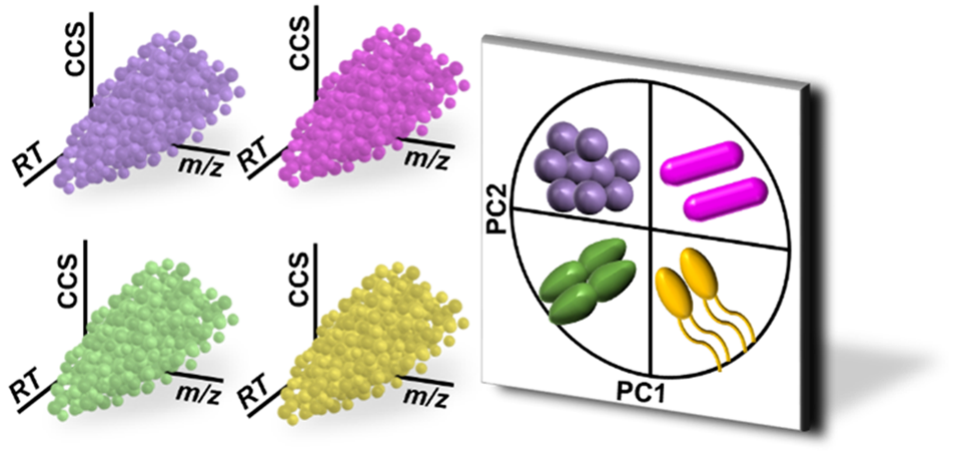

Although MALDI-ToF platforms for microbial identifications have found great success in clinical microbiology, the sole use of protein fingerprints for the discrimination of closely related species, strain-level identifications, and detection of antimicrobial resistance remains a challenge for the technology. Several alternative mass spectrometry-based methods have been proposed to address the shortcomings of the protein-centric approach, including MALDI-ToF methods for fatty acid/lipid profiling and LC-MS profiling of metabolites. However, the molecular diversity of microbial pathogens suggests that no single “ome” will be sufficient for the accurate and sensitive identification of strain- and susceptibility-level profiling of bacteria. Here, we describe the development of an alternative approach to microorganism profiling that relies upon both metabolites and lipids rather than a single class of biomolecule. Single-phase extractions based on butanol, acetonitrile, and water (the BAW method) were evaluated for the recovery of lipids and metabolites from Gram-positive and -negative microorganisms. We found that BAW extraction solutions containing 45% butanol provided optimal recovery of both molecular classes in a single extraction. The single-phase extraction method was coupled to hydrophilic interaction liquid chromatography (HILIC) and ion mobility-mass spectrometry (IM-MS) to resolve similar-mass metabolites and lipids in three dimensions and provide multiple points of evidence for feature annotation in the absence of tandem mass spectrometry. We demonstrate that the combined use of metabolites and lipids can be used to differentiate microorganisms to the species- and strain-level for four of the ESKAPE pathogens (*Enterococcus faecium*, *Staphylococcus aureus*, *Acinetobacter baumannii*, and *Pseudomonas aeruginosa*) using data from a single ionization mode. These results present promising, early stage evidence for the use of multiomic signatures for the identification of microorganisms by liquid chromatography, ion mobility, and mass spectrometry that, upon further development, may improve upon the level of identification provided by current methods.

## Introduction

As the number of bacterial pathogens with resistance to one or more antimicrobial therapeutics increases,^1,2^ there is growing significance in the treatment decisions made early in the management of bacterial infections. The choice of antimicrobial is important both for the health of the infected but also for preventing further spread of or applying additional selective pressure to dangerous multidrug resistant organisms.^3^ The first step in making the right treatment decisions is to determine the identity of the causative microorganism(s). The current standard for microbial identifications is matrix-assisted laser desorption ionization (MALDI) coupled to a time-of-flight mass spectrometer (ToF-MS). These systems (e.g., Biomeireux Vitek, or Bruker Biotyper) use the different protein signatures of microorganisms to provide identifications to the genus level.^4−8^ However, more in-depth identifications (i.e., to the level of species or strain) are challenging, since the key proteomic differences are based on ribosomal proteins that are conserved in closely related organisms.

A handful of alternative mass spectrometry-based approaches has been developed to improve upon the annotation depth provided by the protein-centric approach. Instead of proteins, these methods surveil the differences found in other types of biomolecules between different genera and species of bacteria. Laser desorption ionization (LDI) methods using traditional organic acid matrices or metal oxides, or no matrix at all, have been used to generate lipid profiles of microorganisms.^9−12^ These approaches take advantage of the differences in both lipid structures, headgroups, and fatty acyl tails to distinguish microorganisms. The use of metal oxide matrices for laser ionization (MOLI) facilitates efficient fragmentation of lipid structures to liberate free fatty acids, which provide accurate identifications of microorganisms to the species and strain level.^10,13^ For *Staphylococcus aureus*, the MOLI-MS fatty acid profiling method can distinguish strain-level susceptibility differences including methicillin-resistance.^13^ MALDI-ToF has been used to detect differences in the large lipid structures found in bacteria, such as cardiolipins (CLs), lipoteichoic acid, and lipid A.^12,14^ The glycolipid profile method outperformed protein-based identifications in terms of number of identification and the accuracy of identifications.^15^ Combined with rapid extraction methods, the glycolipid profiling approach can provide identifications within an hour (exclusive of culturing time).^14,16,17^ Although capable of detecting antibacterial resistance in very specific circumstances (colistin resistance in Gram-negative organisms^18^), the glycolipid profiling method has not been tested for strain-level identifications across multiple genera and species of bacteria.

While they are common platforms in diagnostic laboratories, liquid chromatography-MS (LC-MS) methods have lagged behind LDI methods for microbial identifications. The challenges associated with detecting small molecules among the high background of matrix ions in MALDI-ToF makes LC-MS the preferred choice to metabolomics. Similar to lipids, microorganisms of different genera produce metabolites in different quantities or produce unique small molecules. New approaches to microbial identifications take advantage of these differences and the quantitative nature of LC-MS methods to distinguish bacteria based on key metabolite markers. Rather than evaluate intracellular metabolites, which would require an extraction step, the throughput of the process is greatly increased by evaluating secreted, extracellular metabolites released in the culture broth.^19^ A specialized containment device creates a bacteria-free source of culture media while allowing diffusion of small polar metabolites between compartments.^20^ Alternatively, the consumption of metabolites provided in newly developed minimal media can be tracked as a means to monitor growth of bacteria cultured with antibiotics, where decreased consumption correlates to reduced growth caused by susceptibility to the antibiotic.^19,21^ Combined with advances in the throughput of LC methods,^22^ metabolite-based microbial identifications and susceptibility testing dramatically decrease the turnaround times compared to the standard approaches.^19^ Differentiation of closely related species and strains remains a challenge for metabolite-based assays, as compared to lipid- and protein-based methods.

Different strains of the same species may have only a few genomic differences, while susceptibility-level differences can be driven by alterations in a single genetic element. As demonstrated by the single-ome approaches described above, it is unlikely that a single type of biomolecule will be sufficient to universally detect the outcomes of subtle genetic differences in closely related organisms. However, the combined differences across several biochemical pools may provide stronger power to discriminate species- and strain-level differences. A major challenge to this idealized approach is the need for sample preparation methods that are compatible with analytes having very different chemical and physical properties. Direct sample analysis methods can circumvent this issue to some extent by foregoing sample preparation all together. Rapid evaporative ionization mass spectrometry (REIMS) can detect the lipid and metabolite profiles in the aerosol produced from the thermal disruption of bacteria colonies directly from agar plates.^23−26^ The MasSpec Pen (MS Pen) also provides complex spectra of lipids and metabolites from microorganisms but uses a nondestructive liquid sampling approach that is amenable to intraoperative detection of infected tissues.^27^ The requirement for direct contact between the MS Pen and REIMS probes makes them challenging to implement in large-scale sampling of microorganisms, and the resulting data provide qualitative, rather than quantitative, spectral profiles based on exact mass alone.

Ion mobility-mass spectrometry (IM-MS) is a hybrid technique that combines accurate mass-to-charge (*m*/*z*) separation of ionized biomolecules with rapid gas-phase structural separations on the basis of size-to-charge. Given the inherent relationship between size and mass and the differences therein between biomolecular classes, IM-MS spectra of complex biological samples are organized into distinct trendlines that represent different biomolecular classes.^28−30^ These trendlines facilitate rapid classification of unknown features and reduce interference from isobaric overlap between different types of molecules (e.g., lipids, and peptides). By normalizing or calibrating ion mobility drift times into collision cross sections (CCSs), the information from IM-MS experiments can be compared across experiments, laboratories, and instrumentation and used as an additional level of validation for the identifications of unknown *m*/*z* features.^31−33^ IM-MS methods have been used to detect metabolites, lipids, and small proteins/peptides from within a single sample when combined with preparative methods that avoid partitioning analytes into different samples.^34,35^ However, the challenges of analyzing IM-MS data have limited the use of this technology for large-scale, integrated multiomics analyses. Much of the work using IM-MS to investigate biological systems still relies upon chromatographic separation in order to utilize conventional “omics” data analysis pipelines. This need for LC separation, in turn, restricts the biochemical complexity of samples due to solubility issues.

Despite the current limitations, IM-MS presents an opportunity for improvement upon existing single-ome and direct sample analysis methods for the identification of microorganisms by incorporating multiple types of biochemicals into the classification process. Here, we describe the development of a multidimensional LC-IM-MS method for the simultaneous profiling of lipids and metabolites in microorganisms. Our approach utilizes single-phase extractions based on butanol, acetonitrile, and water (BAW^36^) for the recovery of lipids and metabolites from Gram-negative and Gram-positive bacteria. Hydrophilic interaction liquid chromatography (HILIC) enables separation of metabolites and lipids based on their polarities, while ion mobility provides an additional dimension for the separation of coeluting species on the basis of their *size-to-charge* ratios. Using a calibration approach, CCS values from the IM-MS data set are incorporated into the identification process for an extra level of validation. We demonstrate that the HILIC-IM-MS method for simultaneous lipid and metabolomics can easily resolve microorganisms by their Gram-stain status and genera, with promising evidence for the potential of deeper classifications. The contributions of both lipids and metabolites to the discrimination of microorganisms are validated using random forest (RF) and support vector machine (SVM) machine learning feature selection methods. This study provides the first step toward an IM-MS-based platform for identifications of microorganisms with the potential to provide species- and strain-level specificity across many genera through the analysis of multiple biomolecular classes.

## Methods

### Bacteria Species and Culture Conditions

Four species of bacteria, each represented by three strains, were analyzed in this study to cover the diversity of Gram-positive and Gram-negative microorganisms. All strains of the Gram-negative species *Acinetobacter baumannii* (strains NR-52187, NR-52189, and NR-52190) and *Pseudomonas aeruginosa* (strains NR-51517, NR-51588, and NR-51589) were obtained from BEI Resources (NIAID, NIH: provided by the Multidrug-Resistant Organism Repository and Surveillance Network (MRSN) at the Walter Reed Army Institute of Research). *Enterococcus faecium* and *Staphylococcus aureus* were selected as representative Gram-positive bacteria. *E. faecium* strain 700221 was obtained from the American Type Culture Collection (ATCC), and strains HM-952 and HM-959 were obtained from BEI Resources (NIAID, NIH) as part of the Human Microbiome Project. *S. aureus* strains 12600 and 29213 were obtained from ATCC. *S. aureus* strain JE2 (NR-46543) was obtained from BEI Resources (NIH, NIAID: provided by the Network on Antimicrobial Resistance in *Staphylococcus aureus* (NARSA)).

All work with microorganisms was performed under Biosafety Level 2 (BSL-2) conditions. Bacteria were streaked onto agar plates from stocks and incubated overnight at 37 °C. Single colonies were collected from the agar plates and suspended in sterile deionized (DI) water to a turbidity of 2.0–2.05 McFarlands (equivalent to *ca*. 6.0 × 10^8^ CFU/mL). Five biological replicates were prepared for each strain. Tryptic Soy Broth was inoculated at a 1:10 dilution (5 mL total volume) and incubated overnight at 37 °C with shaking (180 rpm). The cultures were then centrifuged at 2700 rpm for 10 min at 4 °C, after which the broth was discarded. The pelleted bacteria were washed and resuspended in 2 mL of sterile water.

### Extraction of Metabolites and Lipids

Prior to extraction, the suspended bacteria were normalized by turbidity to obtain equivalent amounts of bacteria. The suspensions were then aliquoted at 0.5 mL into 8 mL glass culture tubes (for biphasic extraction) or 2 mL polypropylene microcentrifuge tubes (for single-phase extraction) and pelleted by centrifugation. Before extraction solvents were added, stable isotope labeled internal standards of lipids and metabolites were added for recovery and quantitation purposes. The metabolite internal standards (Cambridge Isotope Laboratories) included ^13^C_5_-hypoxanthine (final concentration, 0.5 μg/mL), ^13^C_6_-sucrose (2.5 μg/mL), and ^13^C_5_-l-glutamine (5 μg/mL). The lipid internal standards (Avanti Polar Lipids) included phosphatidylethanolamine (PE) 15:0/d_7_-18:1 (final concentration, 0.375 ng/mL), diacylglycerol (DG) 15:0/d_7_-18:1 (5 ng/mL), and phosphatidylglycerol (PG) 15:0/d_7_-18:1 (0.125 ng/mL).

For the biphasic Bligh and Dyer (B&D) extraction,^37^ the pelleted bacteria were reconstituted with 0.5 mL of HPLC grade H_2_O and sonicated for 30 min at 4 °C. A chilled solution of 1:2 CHCl_3_/MeOH (2 mL) was added to the sample and vortexed for 5 min, followed by the addition of 0.5 mL of CHCl_3_ and 0.5 mL of H_2_O to induce phase separation. After an additional 1 min of vortexing, the samples were centrifuged for 10 min at 3500 rpm and 4 °C. The lower organic layer and the upper aqueous layer of the biphasic solution were collected into separate glass tubes and dried under vacuum. Both dried extracts were reconstituted in 200 μL of 2:2:1 ACN/MeOH/H_2_O and stored at −80 °C or directly diluted for LC-IM-MS analysis.

A single-phase extraction solvent system based on butanol, acetonitrile, and water (BAW) was evaluated for the recovery of both lipids and metabolites. We tested three compositions of the BAW extraction solution: 30% butanol/50% acetonitrile (30% Bu), 45% butanol/35% acetonitrile (45% Bu), and 60% butanol/20% acetonitrile (60% Bu), with H_2_O constant at 20% for all three compositions.^36^ For the extraction, 1 mL of chilled, premixed extraction solution was added to pelleted bacteria. The samples were vortexed and sonicated in an ice bath in alternating 5 min intervals for a total of 30 min. The samples were then chilled at 4 °C for 10 min and then centrifuged at 3500 rpm and 4 °C for 10 min. The supernatants were collected into fresh 2 mL microcentrifuge tubes and dried under vacuum. The dried single-phase extracts were reconstituted in 200 μL of 2:2:1 ACN/MeOH/H_2_O and stored at −80 °C freezer or diluted for LC-IM-MS analysis. Extraction recoveries were evaluated by comparing the peak areas of internal standards in samples spiked with the internal standard mixtures before extraction to those spiked after the extraction. Matrix effects were evaluated by comparing internal standard peak areas from samples to which the internal standard mixture was added after extraction against a neat solution of internal standards without any matrix.

### Hydrophilic Interaction Liquid Chromatography and Ion Mobility-Mass Spectrometry

A single chromatographic method based on hydrophilic interaction liquid chromatography (HILIC) was optimized for the analysis of lipids and metabolites from a single injection. Chromatographic separation was performed on an ACQUITY UPLC BEH Amide column (100 mm × 2.1, 1.7 μm) fitted with a matching precolumn (5 mm × 2.1 mm, 1.7 μm) by using a Waters ACQUITY I-Class Plus FTN UPLC system. The column was maintained at 45 °C with a flow rate of 0.4 mL/min. Solvent A was composed of H_2_O with 10 mM ammonium formate and 0.125% formic acid. Solvent B consisted of ACN/H_2_O (95/5 v/v) with 10 mM ammonium formate and 0.125% formic acid. The gradient, based on Ding et al.,^38^ was as follows: 0–2 min at 100% B, 2–7.7 min from 100% to 70% B, 7.7–9.5 min from 70% to 40% B, 9.5–10.25 min from 40% to 30% B, 10.25–12.75 min from 30% to 100% B, and 12.75–17 min to re-equilibrate to 100% B. An injection volume of 5 μL was used for all samples. Samples were maintained at 6 °C in the autosampler.

The UPLC was connected to the electrospray ionization source of the traveling wave ion mobility-mass spectrometer (Waters Synapt XS). Prior to acquisition of sample data, data were acquired for a mixture of CCS calibrants using direct infusion (see SI-1 Section 1 and SI-1 Tables 1–2 for CCS calibration method details). Randomized sample queues were analyzed in both positive and negative ionization modes. A pooled mixture of all samples was used as a quality control (QC). Data were collected across the entire 17 min chromatographic method using data-independent MS/MS (MS^E^) acquisition. Leucine enkephalin was monitored for postacquisition lockmass correction. Full details of the ionization, ion mobility, and mass spectrometry methods are provided in SI-1 Table 3. Chromatographic performance was evaluated against a mixture representative of bacterial lipid composition prepared with the following standards and reference materials: diacylglycerol (DAG) 16:0/18:1 (Avanti Lipids-800815O), monodiacylglycerol (MGlc-DAG) (*E. coli*) (Avanti Lipids 840522P), digalactosyldiacylglycerol (DGDG) (Avanti Lipids 840524P), phosphatidylethanolamine (PE) 16:0/18:1 (Avanti Lipids 850757C), phosphatidylethanolamine (PE) 18:0/18:1 (Avanti Lipids 850758P), phosphatidylglycerol (PG) 15:0/15:0 (Avanti Lipids 840446P), phosphatidylglycerol (PG) 16:0/16:0 (Avanti Lipids 840455P), phosphatidylglycerol (PG) 18:0/18:1 (Avanti Lipids 840503P), phosphatidylglycerol (PG) 18:0/18:0 (Avanti Lipids 840465P), cardiolipin (CL) 18:1 (Avanti Lipids 710335C), lysylphosphatidylglycerol (LysylPG) 16:0/16:0 (Avanti Lipids 840520P), phosphatidic acid (PA) 16:0/18:1 (Avanti Lipids 840857C), phosphatidylcholine (PC) 16:0/18:1 (Avanti Lipids 850457P), phosphatidylserine (PS) 18:1/18:1 (Avanti Lipids 840035P).

### Data Processing, Multivariate Statistical Analysis, and Feature Identification

Data analysis was performed in Progenesis QI (v3.0, Nonlinear Dynamics, Waters) and EZ Info (v3.0, Umetrics). Peak picking and alignment were performed by using a randomly selected QC sample as the reference. Data normalization was performed with the default “All Compounds” method. Score plots from principal component analysis (PCA) were inspected for clustering of QC samples, from which a maximum ANOVA p-value threshold was determined to reduce technical variability. QC data were then removed, and more stringent ANOVA p-value and fold-change filters were applied to the data set to minimize intragroup variability. For both positive and negative mode data sets, a filter based on an ANOVA p-value of 1 × 10^–9^ (uncorrected) and a minimum fold-change of 3 across all sample groups was applied to data set in Progenesis QI. Given the size of the data set, high significance thresholds were used to reduce the number of retained features for subsequent analysis steps. These filters retained 2249 and 2109 features in the positive and negative mode data sets, respectively. The PCA loadings plot, orthogonal partial least-squares discriminant analysis (OPLS-DA) S-plots, and volcano plots were used to visualize features that contributed to group differences. Additional statistical analyses were performed with MetaboAnalyst 5.0.^39,40^ Peak intensity matrices from Progenesis QI were uploaded to MetaboAnalyst. Interquartile range filtering was applied to reduce the data set by 40% and Pareto scaled. Support Vector Machines (SVMs, for binary classification) and Random Forest (RF, for classification of ≥2 groups) supervised machine learning classifications were performed on the data set using the default settings (SVM: 10-fold cross-validation; RF: 500 trees, 7 predictors, randomness on).

**Figure 1:**
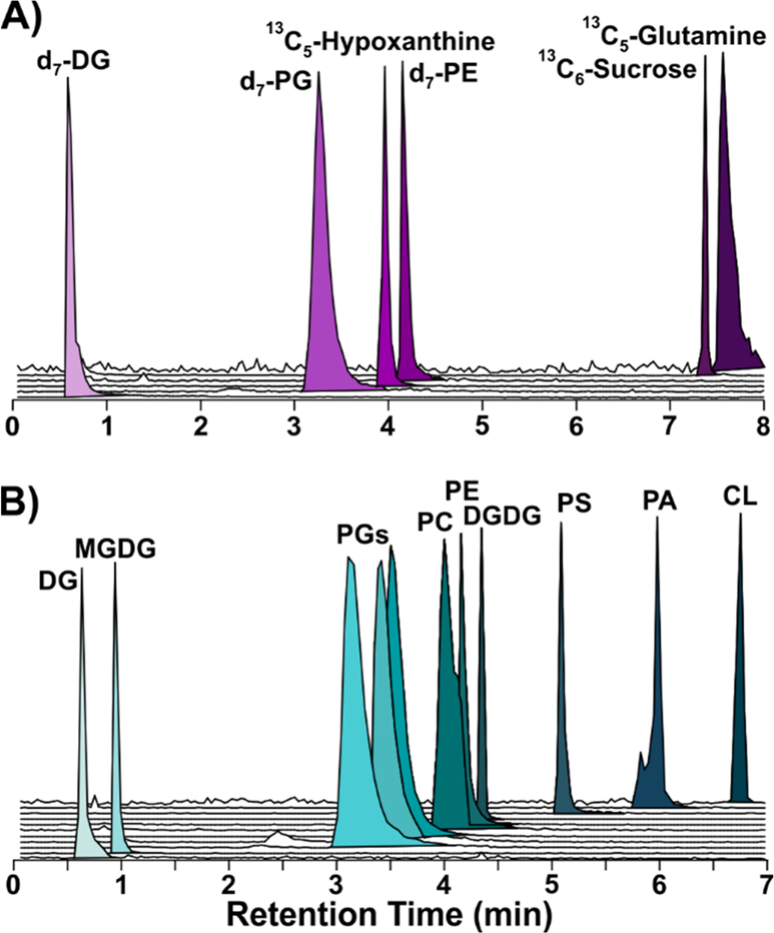
Extracted ion chromatograms from the HILIC separation of A) isotope labeled lipid (DG 15:0/d_7_-18:1, PG 15:0/d_7_-18:1, PE 15:0/d_7_-18:1) and metabolite internal standards and B) lipid standards representative of bacterial lipid species (Listed in order of retention time as follows: DG 16:0/18:1, MGlc-DAG 34:1 (from *E. coli* MGDG extract), PG 18:0/18:1, PG 16:0/16:0, PG 15:0/15:0, PC 16:0/18:1, PE 16:0/18:1, DGDG 36:6 (from plant DGDG extract), PS 18:1/18:1, PA 16:0/18:1, and CL 18:1/18:1/18:1/18:1. See Methods for product numbers and concentrations.).

Identification of significant features was based on accurate mass, retention time, MS/MS spectra, and CCS values. For lipid species, retention times were matched against a reference mixture containing standards of most major lipid species (Figure 1B) that were expected in the microorganisms. Accurate mass was evaluated against an in-house lipid database built from LipidPioneer^41^ and the *Pseudomonas aeruginosa* Metabolome Database (PAMDB)^42^ using a threshold of 10 ppm. Metabolite identifications were made against PAMDB, PubMed, and the Human Metabolome Database (HMDB)^43^ with a threshold of 10 ppm. Matches between calibrated TWIM CCS values and experiment DTIM or predicted CCS values were evaluated against the Unified CCS Compendium,^33^ AllCCS^44^ (as provided within HMDB), and CCSBase.^45^ Additional MS/MS spectra were obtained in a targeted manner to validate identifications of the top significant features. The MS/MS spectra were searched against the Global Natural Product Social Molecular Networking (GNPS) knowledge base.^46^ A minimum of three matching peaks and a mass accuracy with 0.05 Da were required for a positive match. Raw MS/MS spectra and GNPS mirror plots are provided in the Supporting Information (Figures SI-1 7–115).

## Results and Discussion

### Optimization and Evaluation of a Multi-Omics Extraction for Microorganisms

Single-phase extractions have been used with success for the simultaneous recovery of lipids and metabolites from biological matrices including tissue, plasma, yeast and mammalian cells.^47−50^ Among these methods, extractions based on mixtures of water and acetonitrile with butanol or methanol have proven to be highly robust across sample matrices.^51^ However, their application to microorganisms has remained limited.^52,53^ Given the structural differences between eukaryotic and microbial cells, a thorough optimization and evaluation process was performed to identify the proportions of butanol, acetonitrile, and water (BAW) that yielded the highest recovery and reproducibility in a bacterial matrix. *Staphylococcus aureus* strain NR-46543 and *Acinetobacter baumannii* strain NR-52190 were chosen as representative organisms for Gram-positive (G+) and Gram-negative (G-) bacteria. Stable isotope labeled internal standards were selected to match endogenous lipid classes (i.e., PEs and PGs) and common metabolite classes (e.g., amino acids, sugars, and purines) in both organisms (see Figure 1). Three compositions of the BAW extraction solution that varied in the butanol-to-acetonitrile ratio were evaluated against a classic two-phase liquid–liquid Bligh and Dyer (B&D) extraction.

The single-phase BAW extractions performed comparably to the B&D extractions for lipid internal standards in both the G+ and G- matrices (Table 1). Within the *S. aureus* matrix, the recoveries of the DG 15:0/d_7_-18:1, PG 15:0/d_7_-18:1, and PE 15:0/d_7_-18:1 lipid internal standards were mostly in the 90–100% range, with few exceptions. The recoveries of the PE and PG internal standards tended to be lower from the B&D method compared to the three BAW extractions, with PG 15:0/d_7_-18:1 having the largest difference between recoveries (79.5 ± 8.3% for B&D versus 93.7 ± 8.0% for BAW with 30% butanol). A similar trend was observed in the recoveries of PG 15:0/d_7_-18:1 in the *A. baumannii* matrix, whereas the PE and DG internal standards were recovered ca. 90–100% in the B&D and BAW extractions. No significant differences were detected in the recoveries of the lipid internal standards between the two matrices.

**Table 1:** Extraction Recoveries (%) of Lipid and Metabolite Internal Standards Using the BAW and B&D Methods

	*S. aureus*				*A. baumannii*			
Internal Standard	30% Bu	45% Bu	60% Bu	B&D^a^	30% Bu	45% Bu	60% Bu	B&D^a^
DG 15:0–18:1 (d_7_)	91.6 ± 10.0	97.2 ± 4.5	102.4 ± 15.2	88.7 ± 19.3	97.5 ± 9.6	101.5 ± 19.1	92.9 ± 13.4	99.5 ± 3.3
PG 15:0–18:1 (d_7_)	93.7 ± 8.0	95.9 ± 9.5	101.3 ± 3.3	79.5 ± 8.3	88.4 ± 7.7	96.8 ± 6.4	89.9 ± 12.9	77.6 ± 5.4
PE 15:0–18:1 (d_7_)	99.9 ± 3.1	95.9 ± 7.8	95.9 ± 3.6	91.0 ± 6.4	94.4 ± 5.8	94.8 ± 3.6	91.8 ± 5.5	93.0 ± 3.6
Hypoxanthine (^13^C_5_)	100.0 ± 11.3	95.3 ± 4.7	96.5 ± 3.4	87.9 ± 11.5	79.1 ± 23.7	123.5 ± 18.5	75.9 ± 37.5	54.7 ± 12.1
Sucrose (^13^C_6_)	86.5 ± 12.0	93.3 ± 8.9	91.1 ± 10.0	97.8 ± 12.9	67.1 ± 21.8	98.0 ± 8.6	93.1 ± 12.9	65.8 ± 20.4
l-Glutamine (^13^C_5_)	87.9 ± 14.6	96.3 ± 11.8	98.0 ± 7.0	97.6 ± 9.3	66.6 ± 14.5	90.3 ± 3.8	97.6 ± 16.1	66.0 ± 9.3

a Metabolite recoveries from B&D were determined from the aqueous fraction. Lipid recoveries from B&D were determined from the organic fraction.

The recovery of the ^13^C_5_-hypoxanthine, ^13^C_6_-sucrose, and _13_C_5_-l-glutamine metabolite internal standards had greater variability between the four methods as well as between matrices than was observed for the lipids. While all four extractions yielded satisfactory (<85%) recoveries in the *S. aureus* matrix, the recoveries of all three metabolite internal standards were poor (≤80%) in extractions of the *A. baumannii* matrix with the B&D method and the BAW method with 30% butanol. The BAW extractions with 45% and 60% butanol provided extraction recoveries > 90% for the majority of the metabolite internal standards in both matrices. The observation of extraction-specific differences in the recoveries between the Gram-positive and Gram-negative matrices, as in the case of the B&D method, is intriguing and highlights the importance of evaluating extraction recoveries in all relevant matrices.

Matrix effects were also evaluated for the internal standards across both the *S. aureus* and *A. baumannii* matrices and the different extraction methods (SI-1 Table S4). Within a given matrix, there was little influence from the type of extraction on the significance of the matrix effects for the lipid internal standards. The presence of endogenous PE in *A. baumannii* suppressed the intensity of the PE 15:0/d_7_-18:1 internal standard. However, the same effect was not observed with the *S. aureus* matrix that lacks natural PEs. Therefore, the ca. 40–60% decrease in PE 15:0/d_7_-18:1 abundance in the *A. baumannii* matrix is likely due to ionization competition with the more abundant natural PEs. In general, the metabolite internal standard experiences greater matrix effects than the lipids. The *S. aureus* and *A. baumannii* matrices had larger effects on the ^13^C_6_-sucrose and ^13^C_5_-l-glutamine internal standards than ^13^C_5_-hypoxanthine, but the BAW extractions with 30% and 45% butanol were impacted less than the B&D extracts.

**Figure 2:**
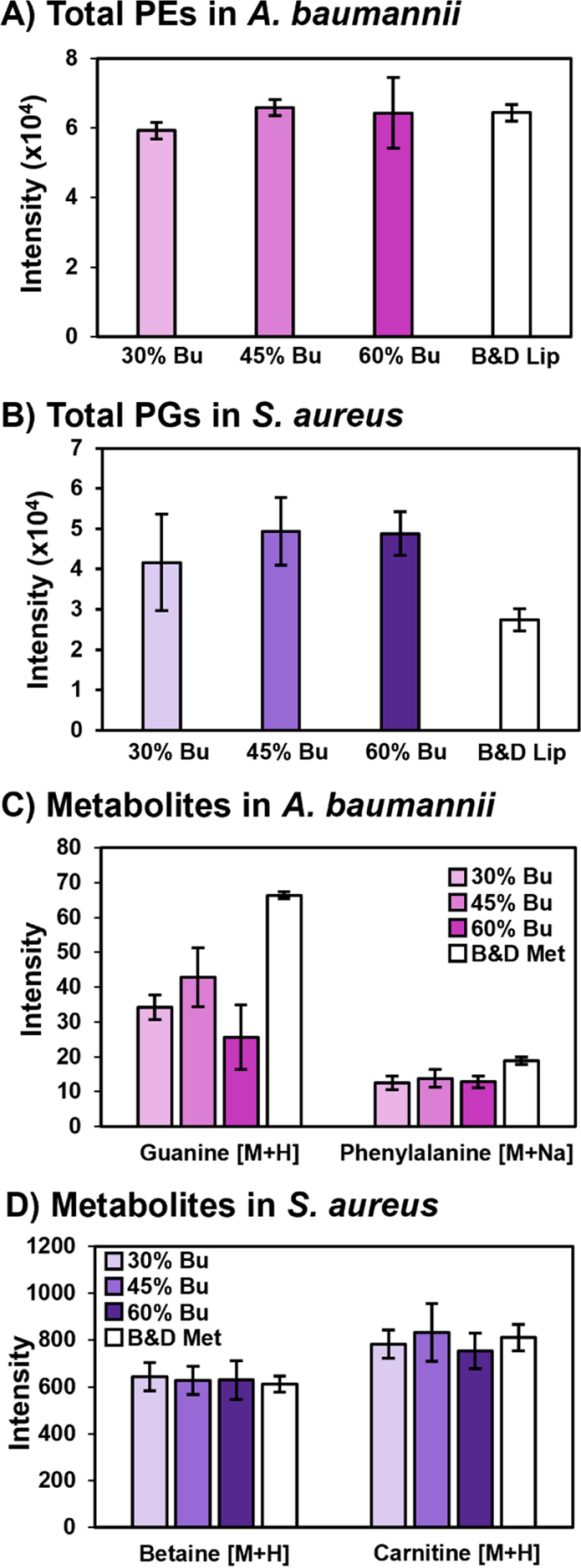
Recovery of endogenous A) PEs (*n* = 7) from *A. baumannii* in negative ionization mode, B) PGs (*n* = 6) from *S. aureus* in negative ionization mode, and metabolites from positive ionization mode analysis of C) *A. baumannii* and D) *S. aureus* using the Bligh and Dyer extraction or BAW extraction with 30%, 45%, or 60% butanol. Results for individual PE and PG species can be found in SI-2 Tables 3 and 4.

We next evaluated the performance of the BAW single-phase and B&D biphasic extractions for the recovery of endogenous lipids and metabolites in *S. aureus* and *A. baumannii*. As shown in Figure 2, more endogenous PEs were extracted from *A. baumannii* using the 45% and 60% butanol BAW extractions and B&D extraction compared to the 30% butanol BAW. Individual PE species in *A. baumannii* (n = 7) showed trends that were consistent with the total recoveries (see SI-2 Table 3). In *S. aureus*, the BAW extraction with 45% butanol yielded the highest total abundance of PGs, followed closely by the BAW extraction with 60% butanol. There was no significant difference between 45% and 60% butanol, while the B&D extraction recovered the least PG from *S. aureus*. Individual PG species (n = 6) had a similar trend where the recovery was worst for B&D, followed by 30% butanol (see SI-2 Table 4). Most of the major metabolites of *S. aureus* showed no preference between the BAW and B&D extractions. However, a higher intensity of adenosine was detected in the BAW extractions compared to B&D. The same trend was detected in *A. baumannii*, as well. Other metabolites detected in *A. baumannii*, including phenylalanine and guanine, were recovered in higher amounts with the B&D extraction though. Among the BAW extractions, the extraction with 45% butanol yielded more guanine and adenosine, whereas phenylalanine showed no difference in extraction yield for the three compositions of BAW. Although higher yields of endogenous metabolites were provided by the B&D method in *A. baumanii*, the poor recovery of the metabolite internal standards and the generation of two extracts (polar and nonpolar) make it less suited for the goals of this work. Based on the collective results, BAW extraction with 45% butanol was selected for the subsequent analysis of lipids and metabolites in microorganisms.

### Hydrophilic Interaction Liquid Chromatography for Simultaneous Metabolomics and Lipidomics

**Figure 3:**
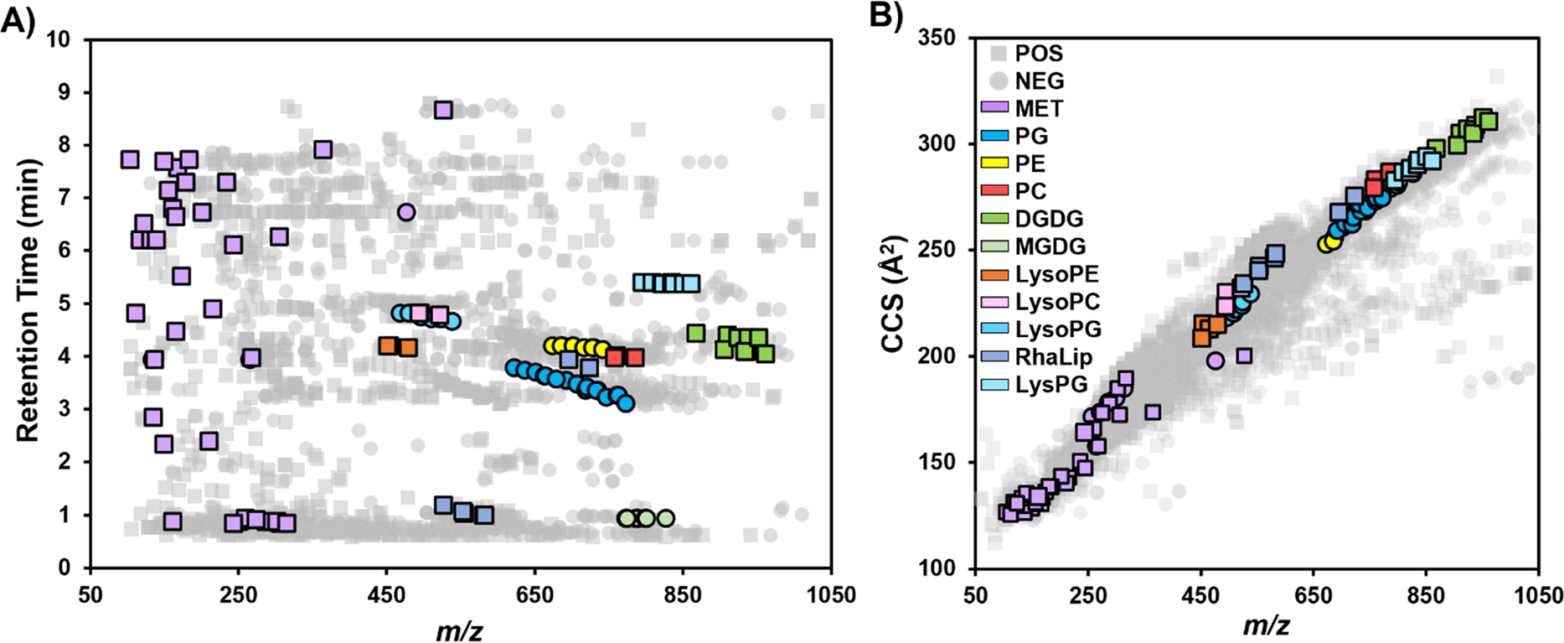
Distribution of identified features in the positive and negative mode data sets across A) HILIC retention time and *m*/*z* (see data in SI-2 spreadsheet ) and B) TWIM calibrated collision cross section (CCS) and *m*/*z*. Features are color coded by their biomolecular class and the ionization mode in which they were detected. Positive and negative mode data have been overlaid, with positive mode data represented by square data points and negative mode data represented by circular data points.

Chromatographic separations based on hydrophilic interaction with silica or amide stationary phases are well-suited for the retention and separation of polar species.^54,49,55,56^ HILIC methods have been developed for metabolomics and lipidomics, but the two are not often combined into a single method due to the partitioning of nonpolar and polar components during the extraction process. The use of single-phase extractions, which generate a single sample containing lipids and metabolites, facilitates the use of a single chromatographic method for the analysis of both biochemical classes.^57^ As shown in Figure 3A, lipids and metabolites elute throughout the gradient. The separation trend for lipids (Figure 3A) mirrors that of other HILIC methods tailored for lipids, with increased retention of lipids with polar headgroups (e.g., LysylPGs) and little retention on nonpolar lipids (e.g., MGDGs). A small amount of separation is achieved based on the acyl tail composition within each lipid class, as shown for PGs 18:0/18:1, 16:0/16:0, and 15:0/15:0 in Figure 1B and the endogenous PGs in Figure 3A.

### Collision Cross Section Calibration for Lipids and Metabolites

A calibration strategy was used to obtain CCS values from the TWIM drift time measurements. The choice of calibrant is well-documented to influence the accuracy of calibrated CCS values.^58^ Structurally matched calibrants are recommended whenever possible, but the simultaneous analysis of lipids and metabolites presents challenges for this strategy. We therefore selected a mixture of CCS calibrants that covered a wide range of masses, chemical classes, and CCS values. The Waters MajorMix contains poly-dl-alanine, small molecules (metabolites and drugs), and fluorinated phosphazines (UltraMark, used widely as Agilent Tune Mix) but lacks lipids or lipid-like structures. To address this gap, we supplemented the MajorMix calibration solution with phosphatidylcholine (PC, n = 6) and phosphatidylethanolamine (PE, n = 3) lipid standards that have been demonstrated to provide high-accuracy calibrated lipid CCS values from TWIM platforms.^59^ We evaluated the performance of calibrations using the combination of small molecules, lipids, and singly charged poly-dl-alanine versus calibrations with lipids and metabolites only. We found that the use of lipids and metabolites provided lower calibration errors for the lipids than the calibration containing poly-dl-alanine, lipids, and metabolites. The resulting mixtures contained 16 calibrant ions for positive (SI-1 Table 1) and 10 ions for negative mode CCS calibrations (SI-1 Table 2). Traditional power law drift time to CCS calibration curves yielded R^2^ values of 0.999, and calibration errors were less than 1% for 24 of 26 calibrant ions (average calibration errors: 0.44% in negative mode, 0.64% in positive mode).

The use of CCS values, calibrated or directly measured, is still challenging to implement as validating information in the identification of unknown metabolites or lipids due to the relatively low number of drift tube measured CCS values that are available in databases.^60^ The development of machine learning tools for predictive CCS determination has been beneficial, but these models still perform best for molecules similar to those on which they were trained.^44,61,62^ Using the calibration strategy described above, we determined the threshold for positive CCS matches against predicted CCS values using identified lipids (n = 28) and metabolites (n = 24) in bacteria. The deviations of calibrated CCS values for annotated lipids from 0.1 to 2.4% (average of 0.7 ± 0.6%) were relative to their predicted CCS values. Deviations for annotated metabolites covered a wider range from 0.1 to 9.3% (average of 2.3 ± 2.6%), with no clear trend in structure or mass for those with the highest deviations. Based on these results, a threshold of ±5% was selected for positive matches between experiment calibrated CCS values and database CCS values (experiment DTIM, calibrated TWIM, and predicted CCS) in support of metabolite and lipid identifications. The calibrated CCS values for the entire data set (positive and negative mode overlaid) are shown in Figure 3B. Unlike the retention time version *m*/*z* plot in Figure 3A, there are clear clusters metabolites, small lipids (i.e., lyso-phospholipids), and larger phospho- and glycolipid species due to the inherent relationship between size and mass. While the use of retention times or CCS values alone as validating evidence of identifications is challenging due to the inherent day-to-day and instrument-to-instrument variability of chromatographic and gas-phase separations, respectively, the two dimensions together can provide compelling support for preliminary identifications based on accurate mass or when MS/MS are ambiguous. For example, the feature 8.65 min_527.1596m/z with a CCS value of 200.3 Å^2^ can be quickly categorized as a polar compound based on its late retention time, but the combination of accurate mass and CCS (within ±5% of AllCCS predicted CCSs) reduces the plausible identifications in HMDB from 46 compounds to 3 similar trisaccharides when considering a 10 ppm mass accuracy threshold and six adduct types. Based on these data and MS/MS spectra, this feature was identified as a sodium adduct of the trisaccharide raffinose.

### Differentiation of Microorganisms by Simultaneous Lipid- and Metabolomics

**Figure 4:**
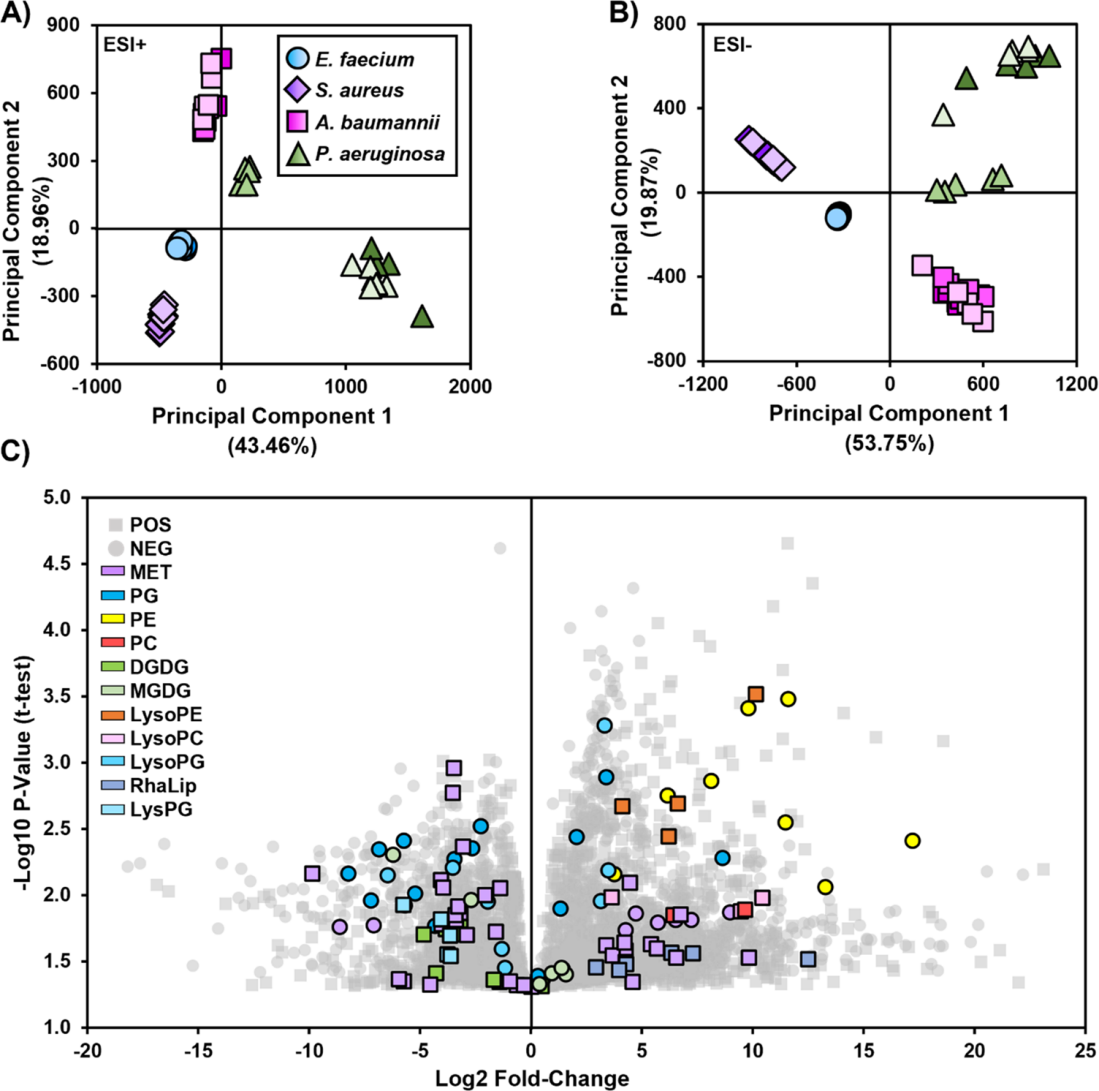
Summary of the differences between the 12 *E. faecium*, *S. aureus*, *A. baumannii*, and *P. aeruginosa* strains in the A) positive and B) negative mode ionization data sets using principal component (PC) analysis. Features contributing to the separation of microorganisms by Gram-stain status along PC1 (Gram-positive, negative PC1 scores; Gram-negative, positive PC1 scores) are indicated in the volcano plot (C), where positive and negative mode data are overlaid. Data points have been colored by their biomolecular classes and the ionization mode in which they were detected. P-values were calculated using the student’s *t* test (two-way, unpaired) and corrected for multiple comparisons using the Benjamini-Hochberg method. PC1 and PC2 here are defined as principal components 1 and 2.

We evaluated 12 individual strains of bacteria representing four different species by LC-IM-MS in positive and negative ionization modes. A summary of the data sets is presented in Figure 4. For both positive and negative mode data sets, over 60% of the variability is described within the first two principal components, and up to 90% variability is accounted for by principal component 3 (SI-1 Figure 2). The principal components analysis (PCA) score plots show a tight overlap of *E. faecium* and *S. aureus* strains in both ionization modes. Intraspecies variability was higher among the Gram-negative organisms, *A. baumannii* and *P. aeruginosa*, with a large difference between *P. aeruginosa* strain NR-51589 and the others that is more pronounced in the positive ionization mode data set (Figure 4A). In the negative mode data set (Figure 4B), the microorganisms separate along principal component 1 based on their Gram stain status, and each species is nearly in its own quadrant. Gram staining is a classical method for classification of microorganisms based on the absence (Gram-positive) or presence (Gram-negative) of an outer membrane. As one of the highest orders of classification, there are well-documented and substantial differences between the lipids and metabolites of Gram-positive and Gram-negative organisms.^63−65^ A volcano plot of student’s *t* test P-values (corrected for multiple comparisons) and fold-changes between Gram-positive versus Gram-negative microorganisms (Figure 4C) highlights some of the identified metabolites (purple) and lipids that differ between the two groups, although there remain many more unannotated than annotated features in the data set.

**Figure 5:**
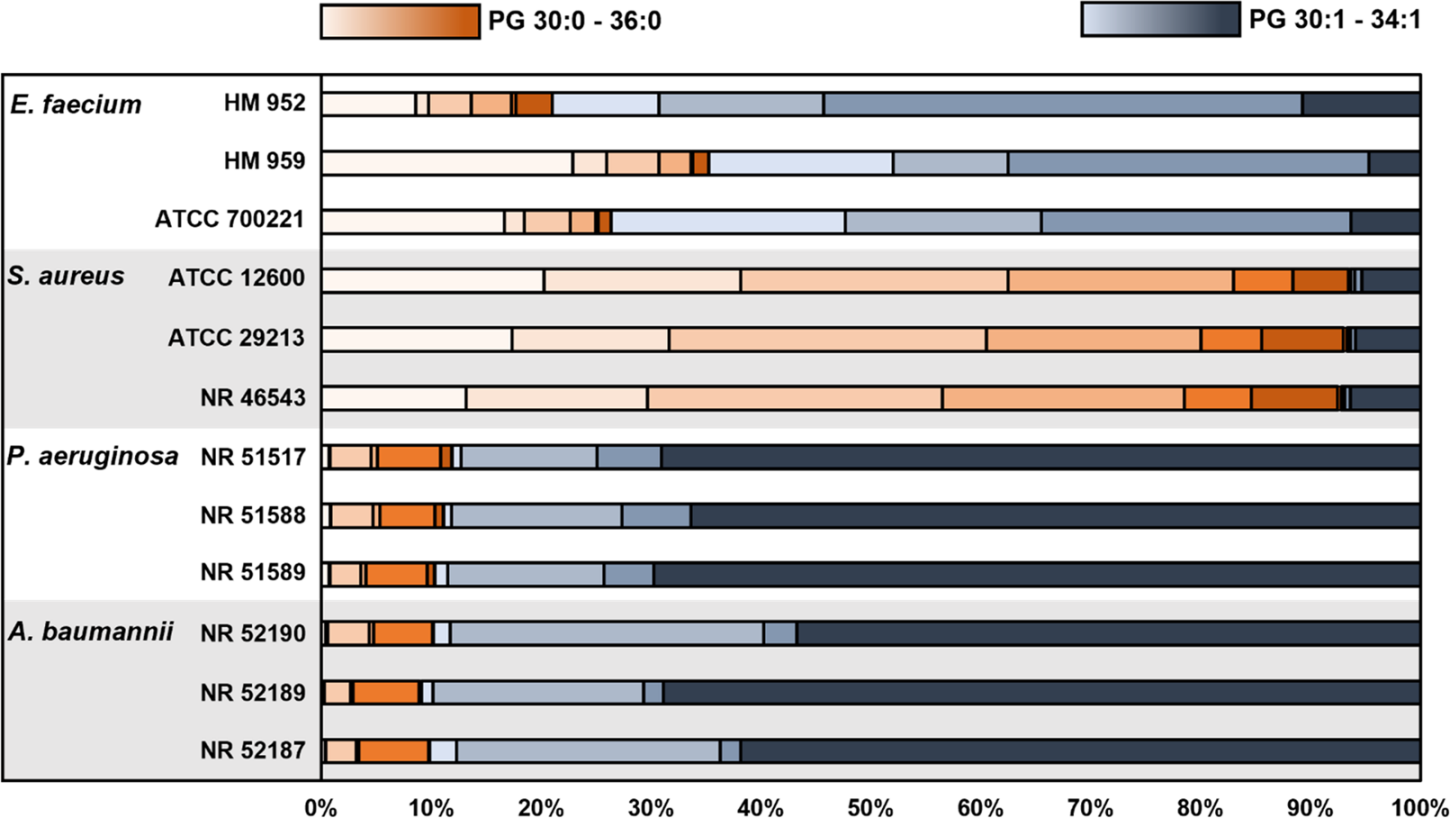
Distribution of saturated and unsaturated fatty acyl tails within the PG lipid species detected in *E. faecium*, *S. aureus*, *P. aeruginosa*, and *A. baumannii* strains. Results are presented using the total carbon: total degrees of unsaturation nomenclature. Acyl tail compositions have been confirmed by MS/MS (see Supporting Information Documents 1 and 2).

Specific to Gram-negative bacteria is the presence of PEs (yellow points), whereas Gram-positive bacteria contain only PGs in their membranes (blue data points). Although all four species contain PGs, Figure 4C shows that there are PGs specific to Gram-positive and Gram-negative organisms. These differences arise from the unique fatty acid content of each organism, which then imparts unique fatty acyl tail compositions within PGs (Figure 5). The Gram-negative species, *A. baumannii* and *P. aeruginosa*, predominantly contain PG with one monounsaturated acyl tail (i.e., PGs 34:1 and 32:1). *E. faecium*, a Gram-positive organism, also contains PGs with monounsaturated acyl tails but differs in the distribution of those lipids (i.e., PG 33:1 > 32:1 > 34:1) and also contains PGs with fully saturated acyl tails that make up 20–40% of total PGs. The acyl tail composition of *S. aureus* (Gram-positive) PGs stands out from the other three species due to its exclusively saturated fatty acid profile.

Several other lipid features can be used to distinguish *A. baumannii* and *P. aeruginosa* from the Gram-positive organisms. Both of the Gram-negative species that we investigated contained phosphatidylcholine (PC, red data points in Figure 4C) lipids that are less commonly detected in bacteria than in PGs and PEs. LysoPCs (pink data points in Figure 4C) were detected as well. *S. aureus* and *E. faecium* both produce a modified form of PG, lysyl-PGs (LysPG), that contains a lysine residue attached to the glycerol headgroup as well as diglucosyl diacylglycerols (DGDGs). In addition to phospholipids, *P. aeruginosa* produces a class of glycolipids known as rhamnolipids (RhaLip) that act as surfactants. Rhamnolipids contain one or two units of rhamnose and up to two beta-hydroxy fatty acids. We detected rhamnolipids in two regions of the HILIC chromatograms (Figure 3A). Rhamnolipids containing one rhamnose unit eluted at *ca*. 1 min, whereas rhamnolipids with two rhamnose units eluted at *ca*. 3.9 min. This is consistent with the retention mechanism of HILIC, where polar compounds are retained longer. In positive ionization mode, rhamnolipids with one rhamnose were detected as sodium adducts, and those with two rhamnose units were detected as ammonium adducts.

**Figure 6:**
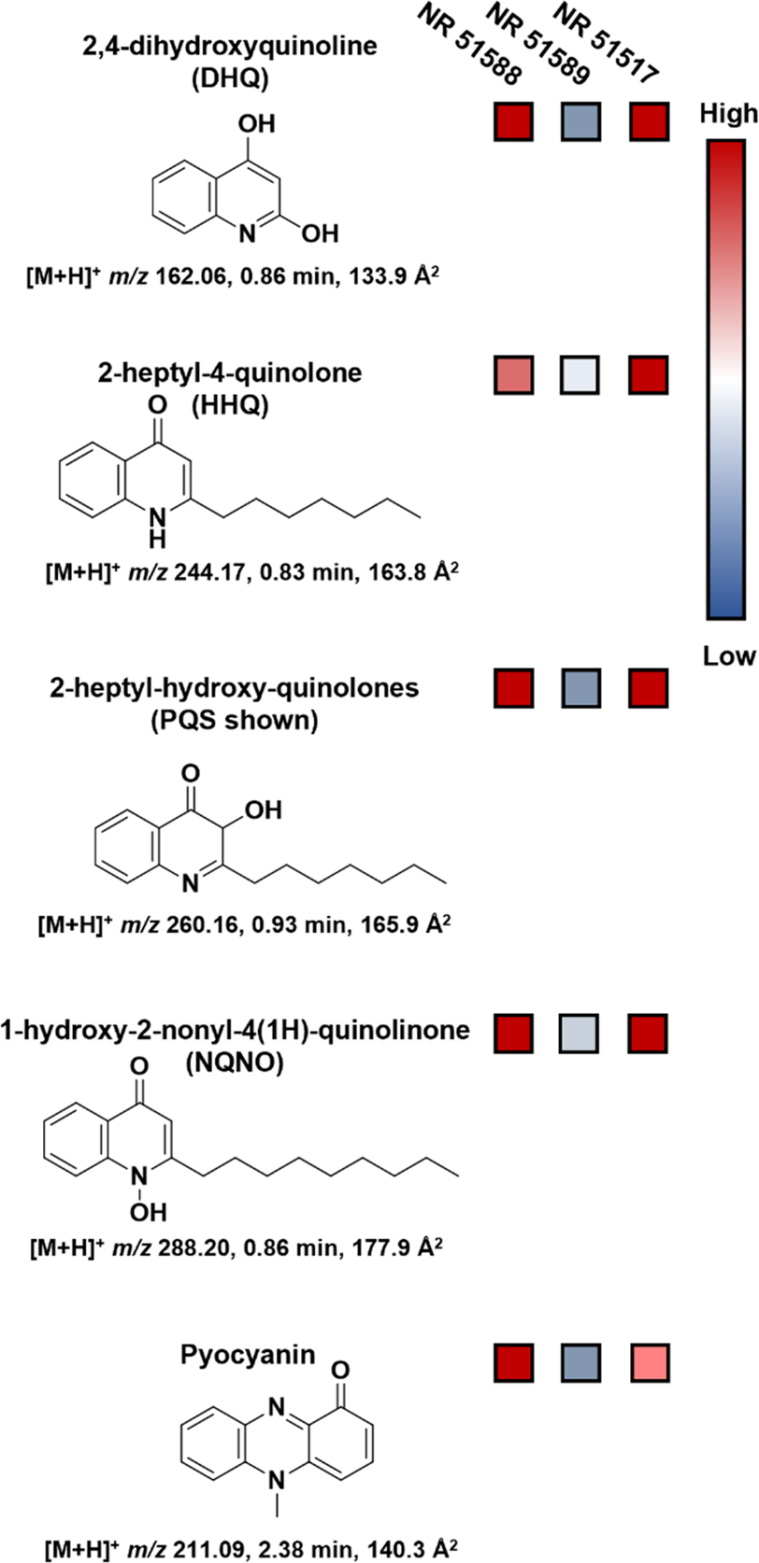
Structures and abundances of molecules in the *Pseudomonas* quinoline signal (PQS) quorum sensing system that were detected in *P. aeruginosa* with the HILIC-IM-MS method. The relative abundance of each molecule in the three *P. aeruginosa* strains (NR-51588, -51589, and -51517) is indicated to the right using a color scale where red is high abundance and blue is low abundance.

Among the identified metabolites, the Gram-positive organisms had higher levels of adenine/adenosine and the osmoregulatory molecules betaine, choline, and carnitine. The majority of the metabolites identified from the right side of the volcano plot, although meant to encompass both Gram-negative organisms, was determined to be *Pseudomonas* quinolone signal (PQS) quorum sensing molecules.^63^ These small molecules are used by *P. aeruginosa* for cell-to-cell signaling to regulate gene expression in response to environmental cues such as population density or external stressors.^66^ Four of the major quinolone quorum sensing molecules are shown in Figure 6 along with their relative intensities in the three *P. aeruginosa* strains. Pyocyanin, a blue pigment responsible for the unique blue-green color of *P. aeruginosa*, was also detected.^67,68^ Although not a quorum sensing molecule itself, the PQS system controls pyocyanin synthesis as a redox-active toxin against other microorganisms.^65,69^ The color scale shows that one *P. aeruginosa* strain, NR-51589, produces significantly less of all of the PQS-related small molecules compared to the others. This pattern, along with a similar decrease in rhamnolipids, is among the driving factors for the overall separation of NR-51589 (midgreen data points) away from the other *P. aeruginosa* strains and toward the *A. baumannii* cluster in the positive mode PCA score plot (Figure 4A).

Although significant strain-level differences are detectable for *P. aeruginosa* from the score plots shown in Figure 4, the two-dimensional score plots for these data sets, which have 9 and 15 components in total, mask differences that are present within the strains of *S. aureus*, *E. faecium*, and *A. baumannii*. To visualize these differences, the data were processed to contain only a single microorganism at a time. The resulting score and loadings plots are shown in SI-1 Figures 3–6. Both the *S. aureus* (SI-1 Figure 5) and *E. faecium* (SI-1 Figure 3) data sets show clear strain-level separations in the more focused analyses that are driven by both lipids and small molecules. Although beyond the scope of this work, the separation of the *S. aureus* and *E. faecium* strains is promising given the known differences in their antibacterial susceptibility profiles (*S. aureus*: 1 methicillin sensitive, 2 methicillin resistant;^70^*E. faecium*: 1 vancomycin-sensitive, 2 vancomycin resistant).^71^

### Machine Learning Selection of Lipid and Metabolite Features for Microorganism Classifications

**Figure 7:**
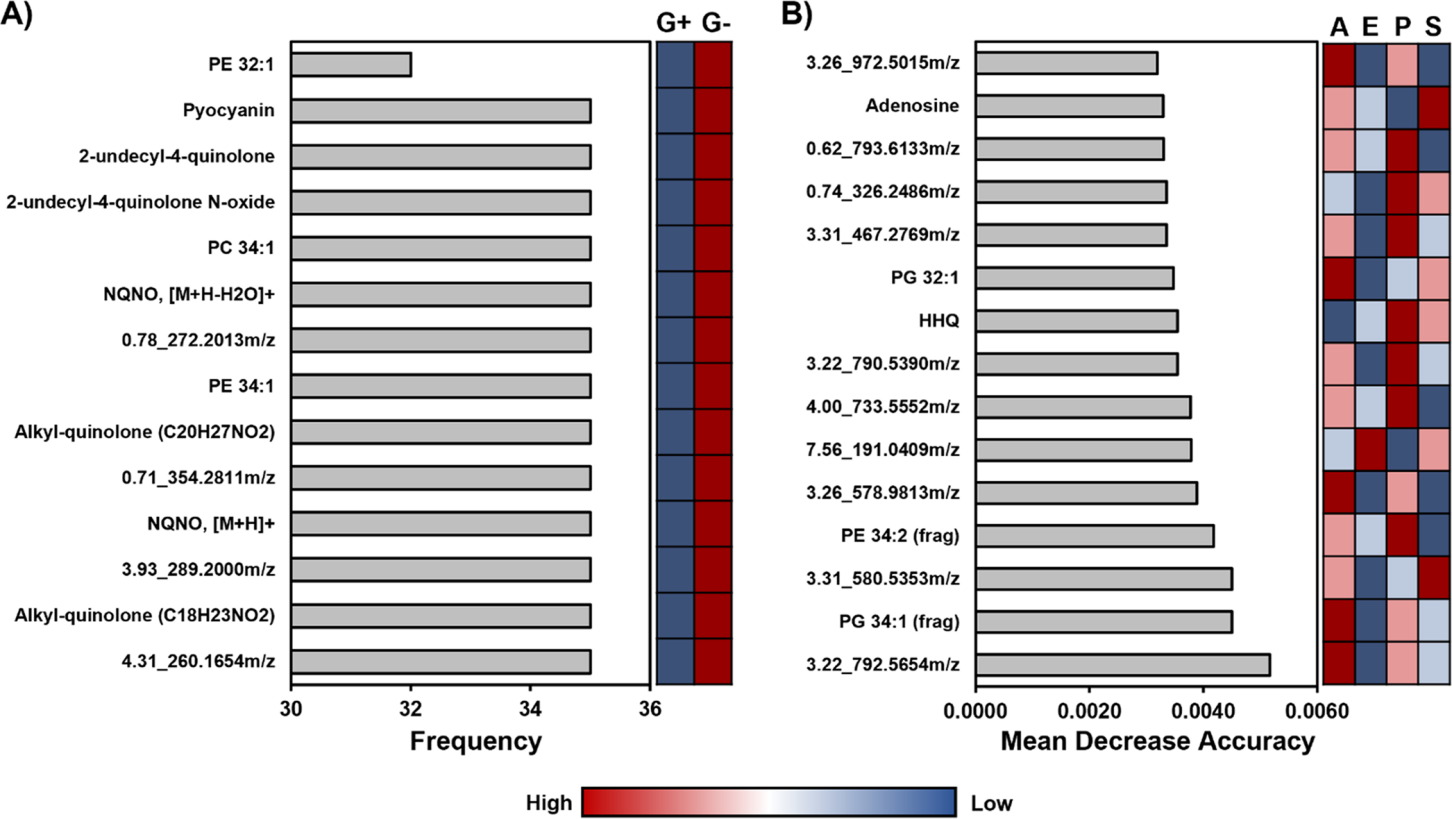
Results of machine learning classification methods for the selection of features that best discriminate A) Gram-negative (G-) vs Gram-positive (G+) microorganisms (support vector machine classification) and B) the four different genera (A, *A. baumannii*; E, *E. faecium*; P, *P. aeruginosa*; S, *S. aureus*) of microorganisms (random forest classification). The relative abundance of each feature is indicated with a color scale where red is a high abundance and blue is a low abundance.

We have demonstrated that HILIC-IM-MS analysis of single-phase microbial extracts can detect the major lipids and small molecules that are different between Gram-positive and Gram-negative organisms, different species of bacteria, and even among different strains of the same microorganism. Although we have evaluated the positive and negative mode data sets, it is desirable for the purpose of throughput to limit analyses to a single-ionization mode. We evaluated whether the data set from a single ionization mode would provide high classification accuracy for Gram-negative versus Gram-positive organisms, as well as discrimination of the four unique genera, using machine learning classification methods. Support vector machines (SVMs) were used for the pairwise classification of Gram-negative versus Gram-positive microorganisms using the full positive mode data set containing 2249 features. The top 14 significant features selected by the SVM method (with error rate of 0.0% at 44 variables/levels) to distinguish Gram-negative and Gram-positive organisms are shown in Figure 7A. Among the features selected with the highest frequency are both small molecules and lipids that are found at higher intensity in the Gram-negative organisms, including PE 34:1, PC 34:1, and pyocyanin. Several more molecules tentatively identified as components of the PQS quorum sensing system were included as well as four small molecules that were not identified.

While the results from the Gram-negative versus Gram-positive classification demonstrate the importance of both lipids and metabolites, it is not a realistic challenge since a well-trained microbiologist could perform such classifications under the microscope. We next evaluated whether the same positive mode data set could be used to discriminate between the four genera of microorganisms. Here, a random forest machine learning method was used for classification of more than 2 groups. The top 15 features selected by the random forest method are shown in Figure 7B. Again, the machine learning approach selected a mixture of both lipids and small molecules for the classification of *Staphylococcus aureus*, *Enterococcus faecium*, *Acinetobacter baumannii*, and *Pseudomonas aeruginosa* that provided high accuracy (i.e., out of bag error of 0%) against this data set. In this case, many of the selected variables are not identified. Although not intended to serve as a model for microbial classifications at this stage, it is promising that both machine learning methods independently selected mixtures of small molecules and lipids to best differentiate the microorganisms from among the 2000+ features within the positive ionization mode data set.

## Conclusions

Using single-phase extractions and hydrophilic interaction liquid chromatography coupled with IM-MS, we developed a method for the simultaneous profiling of lipid and metabolites within four microbial pathogens of high-concern (*S. aureus*, *E. faecium*, *A. baumannii*, and *P. aeruginosa*). The single-phase BAW extraction with 45% butanol was sufficient for the recovery of major lipid species, including phospholipids and small glycolipids with carbohydrate or glycerol backbones (i.e., rhamnolipids, mono-, and diglucosyl diacylglycerols). The HILIC and IM dimensions revealed a high diversity of structures and polarities within the small molecules detected in the microorganisms. CCS values were helpful for the identification of small molecules within essential or central metabolic pathways where their overlap with eukaryotes increased the likelihood that high-quality experimental or predicted CCS values were available. However, many of the small molecules, and even lipids, that are known to distinguish microorganisms, such as the unique quorum sensing molecules of *P. aeruginosa*, are not annotated in existing CCS databases (experimental or otherwise). Other challenges to using CCS values arise from the choice of calibrants for TWIM platforms and the errors associated with CCS calibration, which necessitate generous tolerances for positive matches against CCS repositories.

Although lipidomics and metabolomics are often performed separately, we have demonstrated that both lipids and metabolites are fairly represented in the discriminant analyses of different classifications and genera of microorganisms using the multiomic HILIC-IM-MS data set. The simple discrimination of Gram-positive versus Gram-negative organisms captured many of the known molecular differences that have been used to classify bacteria in singular metabolomics and lipidomics experiments, including lipid classes, acyl tail composition, osmoregulatory molecules, and quorum sensing signals. Using supervised machine learning methods, we demonstrate that data from a single ionization mode can be used to generate classifications that rely upon both lipid and small molecule features. These results hold promise for the development of classification models for microbial identifications based on the HILIC-IM-MS method for simultaneous lipid and metabolite profiling.

## Data Availability

Processed data matrices, raw data files, and other experiment metadata are available from the Metabolomics Workbench (Study ID-ST002854).

## Supporting Information Available

Supporting Information 1. Additional information for spectral analysis from MS/MS experiments, database matching, and statistical analysis (PDF)
Supporting Information 2. Experimental *m*/*z* used for identification with MS/MS experiments, CCS identifications, and lipid acyl tail assignments (XLSX)
