# Supplementary material for: HILIC-IM-MS for Simultaneous Lipid and Metabolite Profiling of Bacteria
Source: ACS Meas Sci Au. 2023 Dec 5;4(1):104–16. doi: 10.1021/acsmeasuresciau.3c00051 (PMC10885331; doi:10.1021/acsmeasuresciau.3c00051)
Supplement: Supplementary file 1 — tg3c00051_si_001.pdf [file tg3c00051_si_001.pdf]

Supporting Information for

HILIC-IM-MS for Simultaneous Lipid and Metabolite Profiling of Bacteria

Jana M. Carpenter[1], Hannah M. Hynds[1], Kingsley Bimpeh[1], and Kelly M. Hines[1]*
[1]Department of Chemistry, University of Georgia, Athens, Georgia 30602, United States

*Correspondence should be addressed to: Dr. Kelly Hines, kelly.hines@uga.edu

Table of Contents

Section 1: Ion Mobility and Mass Spectrometry Methods

CCS Calibration Protocol……………………………………………………………………S3

SI Figure 1. CCS Calibration Comparisons (Progenesis vs. Manual) ………………………S5

Table S1. Positive Mode CCS Calibrants ……………………………………………………S6

Table S2. Negative Mode CCS Calibrants……………………………………………………S7

Table S3. Ionization Settings…………………………………………………………………S8

Section 2: Extraction Evaluation

Table S4. Matrix Effects Results……………………………………………………………S9

Section 3: PCA and S-Plots/Loadings

SI Figure 2. Goodness of Fit…………………………………………………………………S10

SI Figure 3. *E. faecium*………………………………………………………………………S11

SI Figure 4. *S. aureus*………………………………………………………………………S12

SI Figure 5. *A. baumannii*……………………………………………………………………S13

SI Figure 6. *P. aeruginosa*……………………………………………………………………S14

Section 4: MSMS Spectra

SI Figures 7-43. Positive Mode (Metabolites)…………………………………………………S15

SI Figures 44-58. Negative Mode (Lipids)……………………………………………………S50

Section 5: GNPS Mirror Plots

SI Figures 59-86. Negative Mode Data (Lipids)………………………………………………S65

SI Figures 87-115. Positive Mode (Metabolites)………………………………………………S93

Section 6: MetaboAnalyst Analysis

SI Figure 116. Random Forest: Classification…………………………………………………S122

SI Figure 117. Random Forest: Variable Outlier Detection……………………………………S123

SI Figure 118. Random Forest: Variable Importance of Projection (VIP)………………………S124

SI Figure 119. Support Vector Machine (SVM): Classification…………………………………S125

SI Figure 120. Support Vector Machine (SVM): Feature Frequency……………………………S126

CCS Calibration Protocol

The Major Mix IMS/ToF Calibration Kit (Waters, Manchester, UK) solution was spiked with saturated phosphatidylcholine (PC) and phosphatidylethanolamine (PE) standards for collision cross section (CCS) calibration. Ion drift times and CCS values were extracted using Water's Progenesis QI. Calibrated CCS values were also calculated manually using a modified version of the Mason-Schamp equation (**SI Eqn 1-3**)[1] with optimized parameters for a power law regression that utilizes analyte drift time and $^{DT}CCS_{N2}$ ($Å^2$) values obtained from literature.[2] A comparison of the CCS values for a set of identified experimental features obtained manually and through Progenesis software can be seen in the supporting information (**Fig SI X**). On average, the power law CCS values were more accurate, so these values will be reported. Due to the percent CCS error of the calculated CCS values for the set of experimental features compared to literature values[3, 4] and predicted values[5], a percent error in CCS values ≤ 5% was deemed acceptable for reported features.

Power Law CCS Calibration

Manual CCS Calibration was performed using optimized parameters for a power law regression that relates corrected analyte drift time and corrected CCS.

$$t'_d = DT - \left(\frac{\left(\sqrt{m/_z} * EDC\right)}{1000}\right)$$

SI Eqn 1. Corrected drift time formula where t_d' is corrected drift time, *m/z* is mass-to-charge of the calibrant, and EDC is the enhanced duty cycle delay coefficient.

$$\Omega' = {}^{DT}CCS_{N2} * \sqrt{\mu}$$

SI Eqn 2. Corrected CCS formula where Ω' is corrected CCS, $^{DT}CCS_{N2}$ is literature CCS values obtained using drift-tube ion mobility, and μ is the reduced mass.

$$\Omega' = A' * (t'_d + t_0)^B$$

SI Eqn 3. Modified Mason-Schamp equation where Ω' is corrected CCS, t_d' is corrected drift time, and A', t_0, and B are parameters optimized through power law regression.

$$\Omega = \frac{A'}{\sqrt{\mu}} * (t'_d + t_0)^B$$

SI Eqn 4. Calculated CCS formula where Ω is an unknown feature's calculated CCS, t_d' is corrected drift time, μ is the reduced mass, and A', t_0, and B are parameters optimized through power law regression.

(1) Mason, E. A.; Shamp Jr., H. W., Mobility of gaseous ions in weak electric fields. *Annals of Physics* **1958,** *4* (3), 233-270.
(2) Hines, K. M., et al., Evaluation of Collision Cross Section Calibrants for Structural Analysis of Lipids by Traveling Wave Ion Mobility-Mass Spectrometry. *Anal. Chem.* **2016,** *88* (14), 7329-7336.

(3) Hines, K. M., et al., Characterization of the Mechanisms of Daptomycin Resistance among Gram-Positive Bacterial Pathogens by Multidimensional Lipidomics. *mSphere* **2017,** *2* (6).
(4) Picache, J. A., et al., Collision cross section compendium to annotate and predict multi-omic compound identities. *Chem. Sci.* **2019,** *10* (4), 983-993.
(5) Ross, D. H., et al., Breaking Down Structural Diversity for Comprehensive Prediction of Ion-Neutral Collision Cross Sections. *Anal. Chem.* **2020,** *92* (6), 4548-4557.

CCS Calibration Comparison (Progenesis vs. Manual)

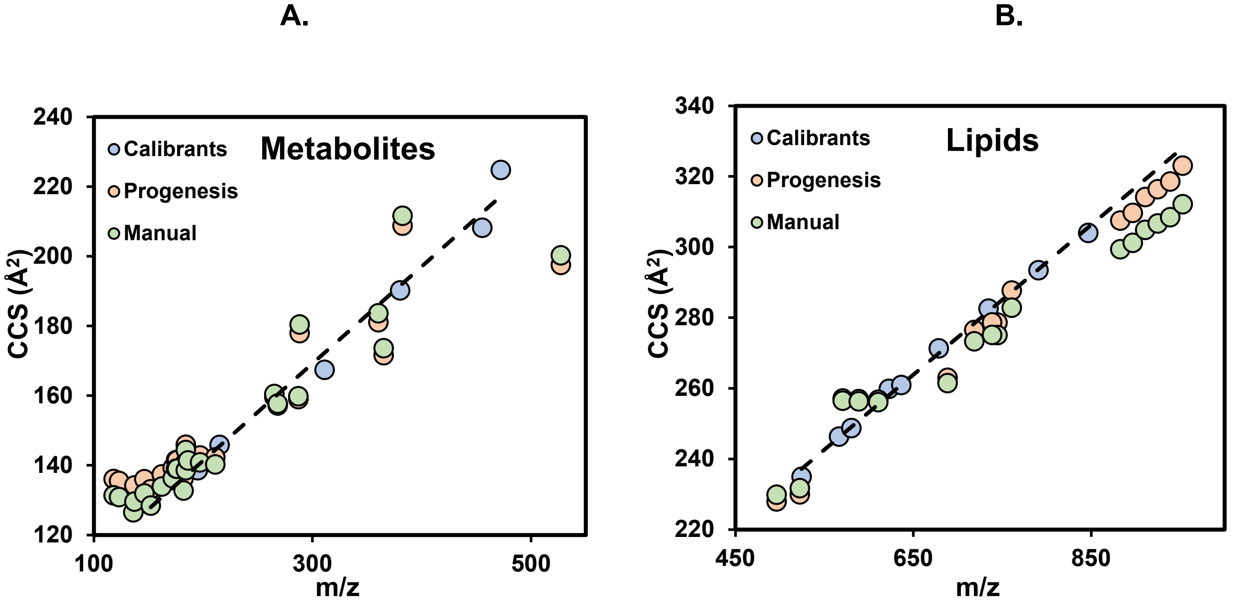

SI Figure 1. The results of the manual CCS calibration versus the Progenesis QI CCS calibration are shown in the endogenous metabolites (**panel A**) and endogenous lipids (**panel B**) above panels. The extracted calibrant data is also plotted as a reference. There is an average 3.43% error in CCS calibrated values for Progenesis versus 2.30% error for Manual calibration for metabolites. In the case of lipids, there is only a 0.95% error in the Progenesis calculated CCS values as compared to a 0.96% error in the manual CCS calibration in lipids.

SI Table-1. CCS calibrants for positive mode.

Calibrant	Adduct	m/z	DT	Cal. CCS (Å)	Lit. CCS (Å)
**acetaminophen**	[M+H]+	152.0714	1.601	132.0	130.4
**caffeine**	[M+H]+	195.089	1.801	138.6	138.2
**sulfaguanidine**	[M+H]+	215.0578	1.989	145.9	146.8
**sulfadimethoxine**	[M+H]+	311.0821	2.592	167.4	168.4
**Val-Tyr-Val**	[M+H]+	380.2163	3.237	190.2	191.7
**verapamil**	[M+H]+	455.2901	3.779	208.2	208.8
**terfenadine**	[M+H]+	472.3174	4.275	224.8	228.7
**PC 10:0**	[M+H]+	566.3805	4.975	246.4	245.4
**PC 12:0**	[M+H]+	622.4373	5.426	259.9	258.4
**PC 14:0**	[M+H]+	678.5057	5.819	271.3	270.4
**PC 16:0**	[M+H]+	734.5673	6.211	282.6	282.5
**PC 18:0**	[M+H]+	790.6263	6.598	293.5	294.5
**PC 20:0**	[M+H]+	846.6873	6.979	304.0	306.4
**PE 10:0**	[M+H]+	524.3317	4.605	235.0	233
**PE 12:0**	[M+H]+	580.3925	5.055	248.7	246.7
**PE 14:0**	[M+H]+	636.4563	5.465	260.9	259.2

SI Table-2. CCS calibrants for negative mode.

Calibrant	Adduct	m/z	DT	Cal. CCS (Å)	Lit. CCS (Å)
**PE 10:0**	[M-H]-	522.3232	4.031	225.2	223.2
**PE 12:0**	[M-H]-	578.3909	4.464	236.7	235.7
**PE 14:0**	[M-H]-	634.4495	4.907	247.9	247.7
**PE 16:0**	[M-H]-	690.5175	5.366	259.0	259.4
**PE 18:0**	[M-H]-	746.5817	5.87	270.7	271.6
**acetaminophen**	[M-H]-	150.06	1.293	132.5	131.5
**theophylline**	[M-H]-	179.06	1.323	132.3	132.4
**sulfaguanidine**	[M-H]-	213.04	1.592	144.3	145.2
**sulfadimethoxine**	[M-H]-	309.07	2.263	170.2	170.1
**Val-Tyr-Val**	[M-H]-	378.20	2.858	190.6	192.5

SI Table-3. Ionization settings for both positive and negative mode, with optimization for small molecules.

Tab	Name	Setting
ES+	Capillary	+3 kV
ES-	Capillary	-2 kV
ES+	Sampling Cone	30 V
ES-	Sampling Cone	25 V
ES+/-	Source Offset	40 V
ES+/-	Source Temp	150 ºC
ES+/-	Desolvation Temp	400 ºC
ES+/-	Cone gas flow	50 L/h
ES+/-	Desolvation gas flow	650 L/h
ES+/-	Nebulizer gas flow	7 Bar
StepWave	SW1 wave height	0 V
StepWave	SW1 wave velocity	300 m/s
StepWave	SW2 wave height	0 V
StepWave	SW2 wave velocity	300 m/s
StepWave	SW2 offset	200
StepWave1	Stepwave RF offset	150
StepWave2	Ion Guide RF	300
Instrument	IMS gas flow	70 (mL/min)
TriWave	IMS wave velocity	650 (m/s)
TriWave	IMS wave height	35 V
TriWave DC	Trap Wave entrance	3
TriWave DC	Trap bias	40
TriWave DC	Trap DC	-4 V
TriWave DC	Trap Exit	0
TriWave DC	IMS entrance	12
TriWave DC	IMS He cell DC	35
TriWave DC	IMS He exit	12
TriWave DC	IMS bias	10
TriWave DC	IMS exit	0
MS Profile	Mass 1	75
MS Profile	Dwell time	20 ms
MS Profile	Ramp time	30 ms
MS Profile	Mass 2	300
MS Profile	Dwell time	20 ms
MS Profile	Ramp time	30 ms
MS Profile	Mass 3	600

Table S4. Matrix effects (%) for lipid and metabolite internal standards in BAW and B&D extracts of *S. aureus* and *A. baumannii*.

	S. aureus				***A. baumannii***			
**Internal Standard**	**30% Bu**	**45% Bu**	**60% Bu**	**B&D***	**30% Bu**	**45% Bu**	**60% Bu**	**B&D***
DG 15:0-18:1 (d_7)	343.8 ± 22.4	342.2 ± 50.4	301.2 ± 52.1	198.4 ± 30.9	165.5 ± 10.6	215.0 ± 32.1	173.7 ± 12.2	147.0 ± 16.6
PG 15:0-18:1 (d_7)	112.0 ± 8.6	113.9 ± 13.2	108.9 ± 6.0	102.9 ± 5.2	170.6 ± 15.2	161.1 ± 20.4	156.3 ± 12.3	155.8 ± 14.3
PE 15:0-18:1 (d_7)	115.8 ± 14.9	124.4 ± 18.9	113.0 ± 13.3	151.8 ± 20.3	68.1 ± 5.5	65.1 ± 9.1	68.9 ± 4.0	42.4 ± 3.4
Hypoxanthine ($^{13}C_5$)	59.0 ± 2.3	60.5 ± 4.5	55.2 ± 2.2	78.4 ± 3.0	91.0 ± 4.7	87.7 ± 4.3	87.5 ± 5.0	33.3 ± 1.5
Sucrose ($^{13}C_6$)	17.1 ± 6.1	18.7 ± 2.4	4.9 ± 0.6	3.7 ± 0.5	8.7 ± 1.2	8.3 ± 0.6	8.6 ± 1.1	2.5 ± 0.2
L-Glutamine ($^{13}C_5$)	15.9 ± 1.0	19.9 ± 1.5	11.0 ± 1.9	12.2 ± 1.2	20.6 ± 3.7	20.0 ± 4.8	16.7 ± 5.1	0.5 ± 0.3

*Metabolite recoveries from B&D were determined from the aqueous fraction. Lipid recoveries from B&D were determined from the organic fraction.

Goodness of Fit

A. **B.**

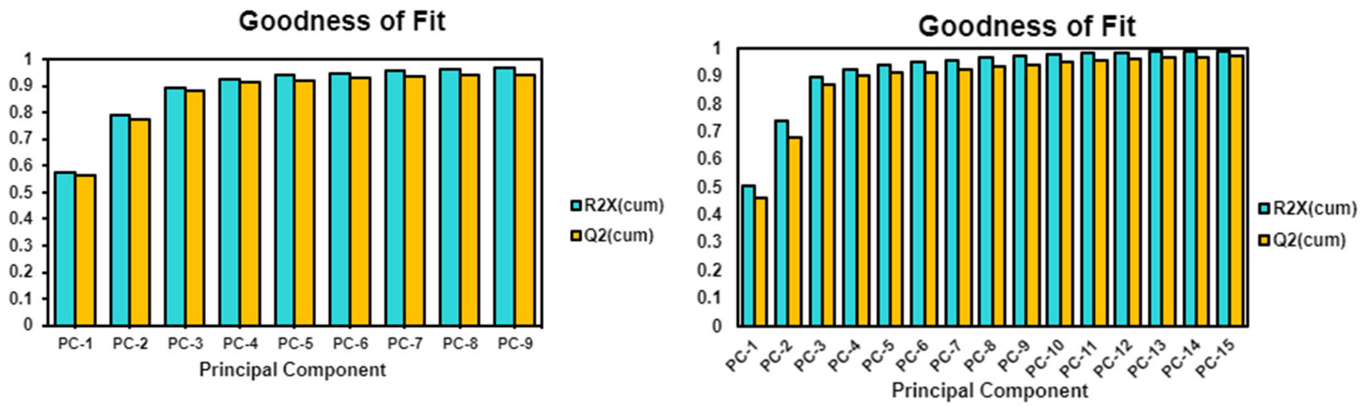

SI Figure 2. Given the goodness of fit plot, over 90% of the variability presented in the positive mode dataset (**panel A**) is explained by 9 principal components. Likewise, over 90% of the variability in the negative mode dataset (**panel B**) is explained by 15 principal components.

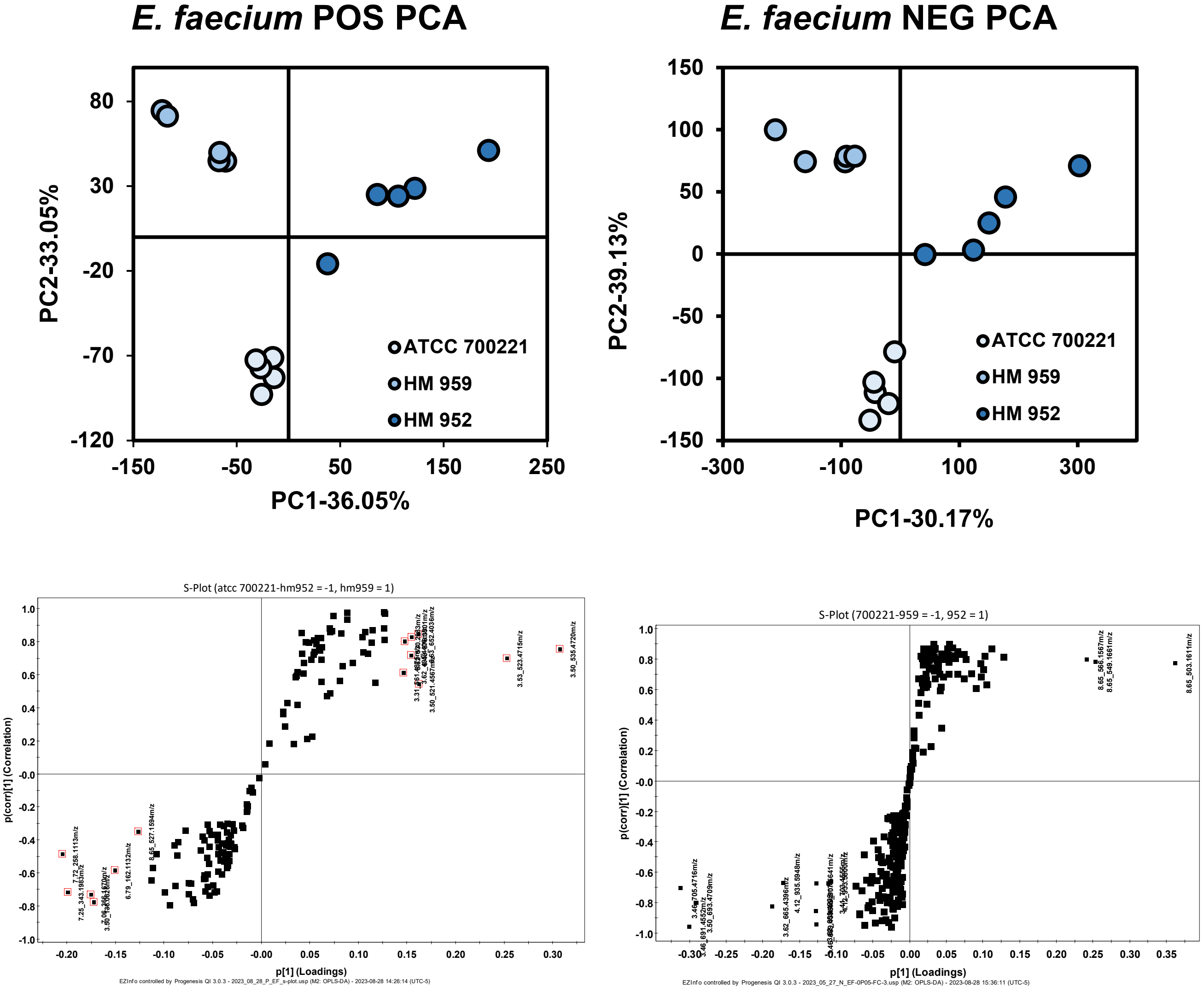

SI Figure 3. Shows the differences between strains using Principal Components Analysis, where for positive mode data 36.05% of the overall variance is explained by PC 1 and 33.05% of the variance if explained by PC 2. For negative mode data, 30.17% of the overall variance is explained by PC 1 and 39.13% of the variance is explained by PC 2. Additionally, the corresponding S-plots are based on the orthogonal-partial-least squares discriminant analysis of two groups separated by PC 1 in both positive (bottom left) and negative (bottom right) ionization modes.

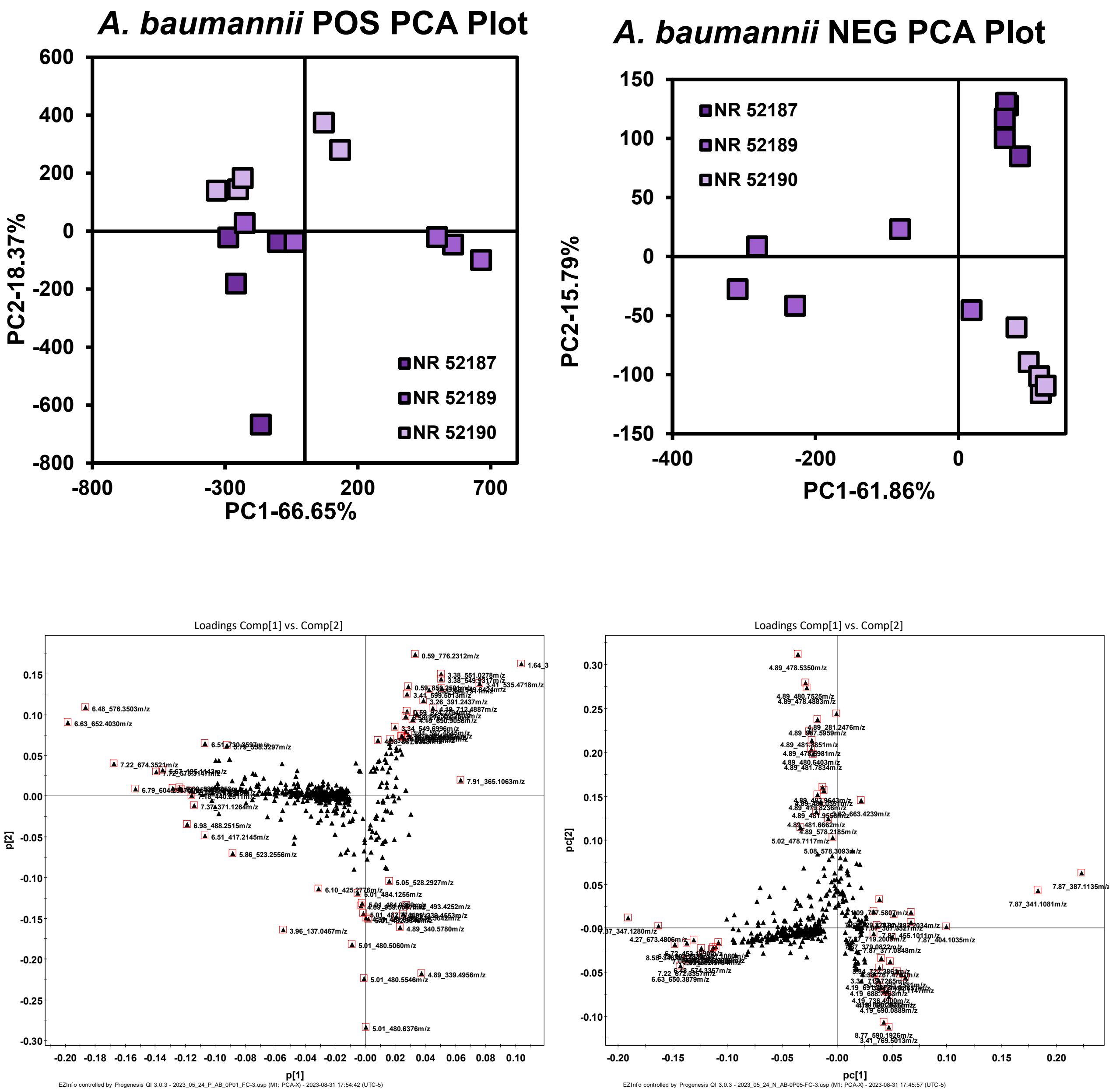

SI Figure 4. Shows the differences between strains using Principal Components Analysis, where for positive mode data 66.65% of the overall variance is explained by PC 1 and 18.37% of the variance if explained by PC 2. For negative mode data, 61.86% of the overall variance is explained by PC 1 and 15.79% of the variance is explained by PC 2. Additionally, the corresponding loadings plots are based on the partial-least squares discriminant analysis of all three groups in both positive (bottom left) and negative (bottom right) ionization modes.

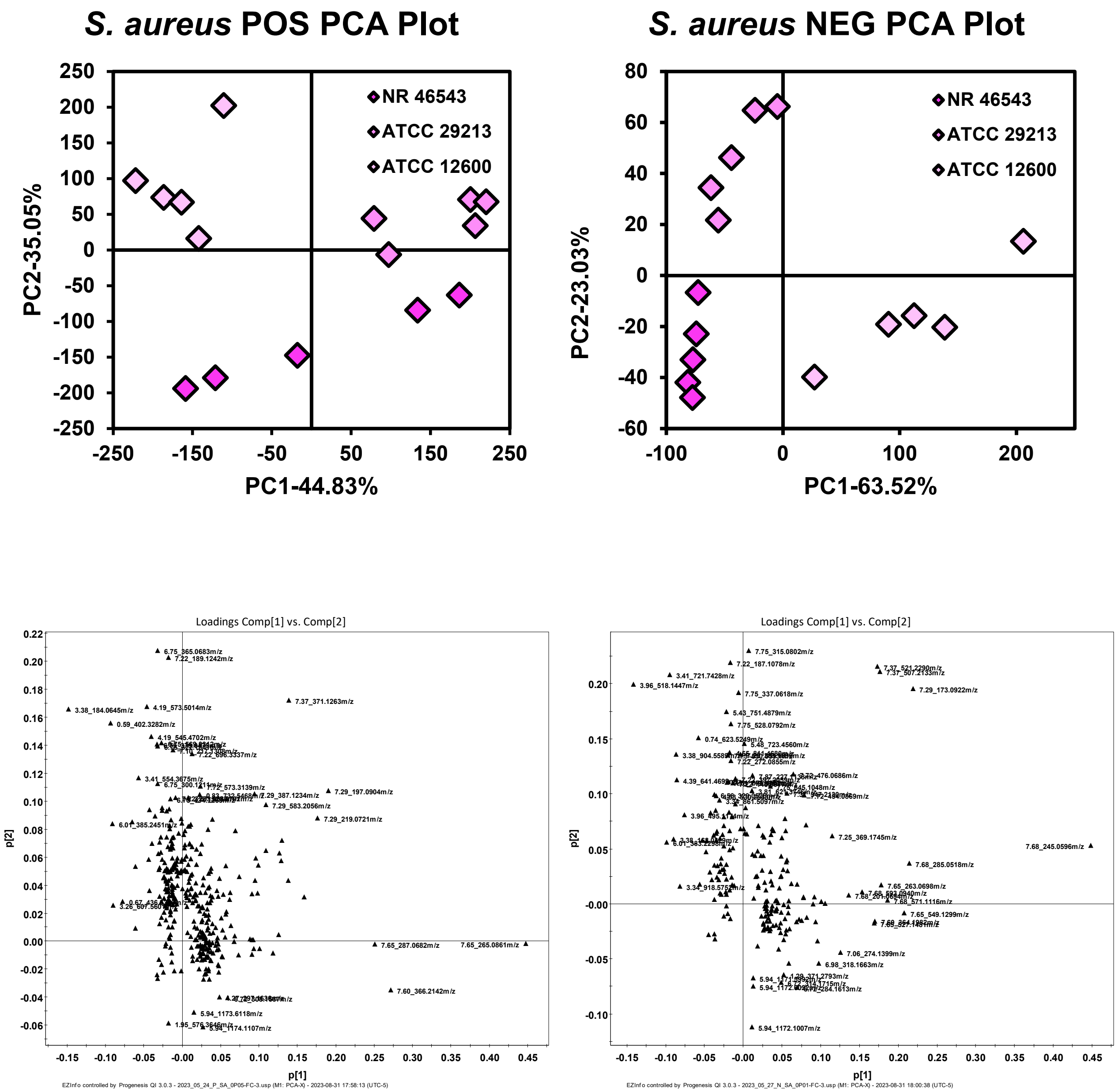

SI Figure 5. Shows the differences between strains using Principal Components Analysis, where for positive mode data 44.83% of the overall variance is explained by PC 1 and 35.05% of the variance if explained by PC 2. For negative mode data, 63.52% of the overall variance is explained by PC 1 and 23.03% of the variance is explained by PC 2. Additionally, the corresponding loadings plots are based on the partial-least squares discriminant analysis of all three groups in both positive (bottom left) and negative (bottom right) ionization modes.

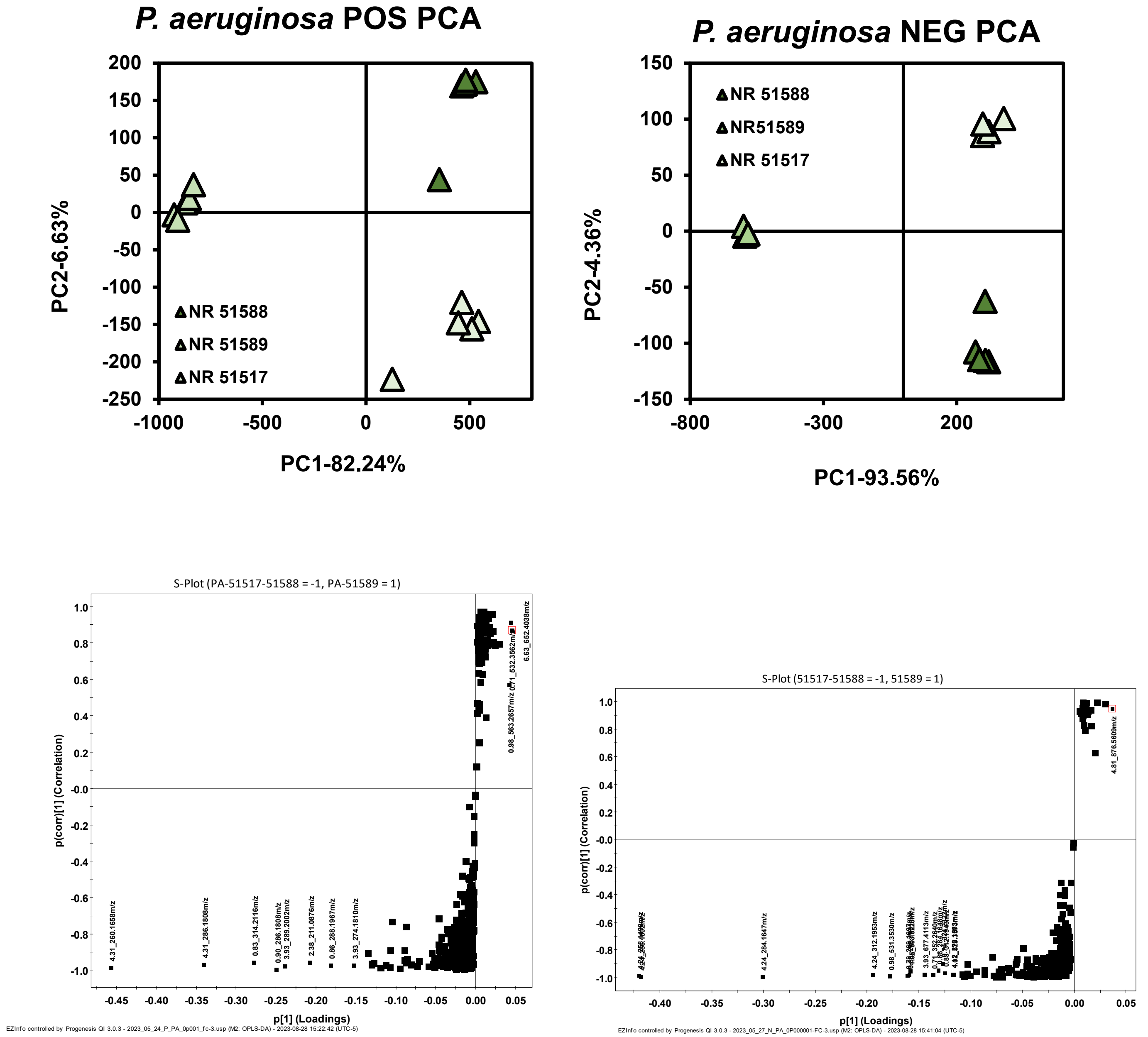

SI Figure 6. Shows the differences between strains using Principal Components Analysis, where for positive mode data 82.24% of the overall variance is explained by PC 1 and 6.63% of the variance if explained by PC 2. For negative mode data, 93.56% of the overall variance is explained by PC 1 and 4.36% of the variance is explained by PC 2. Additionally, the corresponding S-plots are based on the partial-least squares discriminant analysis of all three groups in both positive (bottom left) and negative (bottom right) ionization modes.

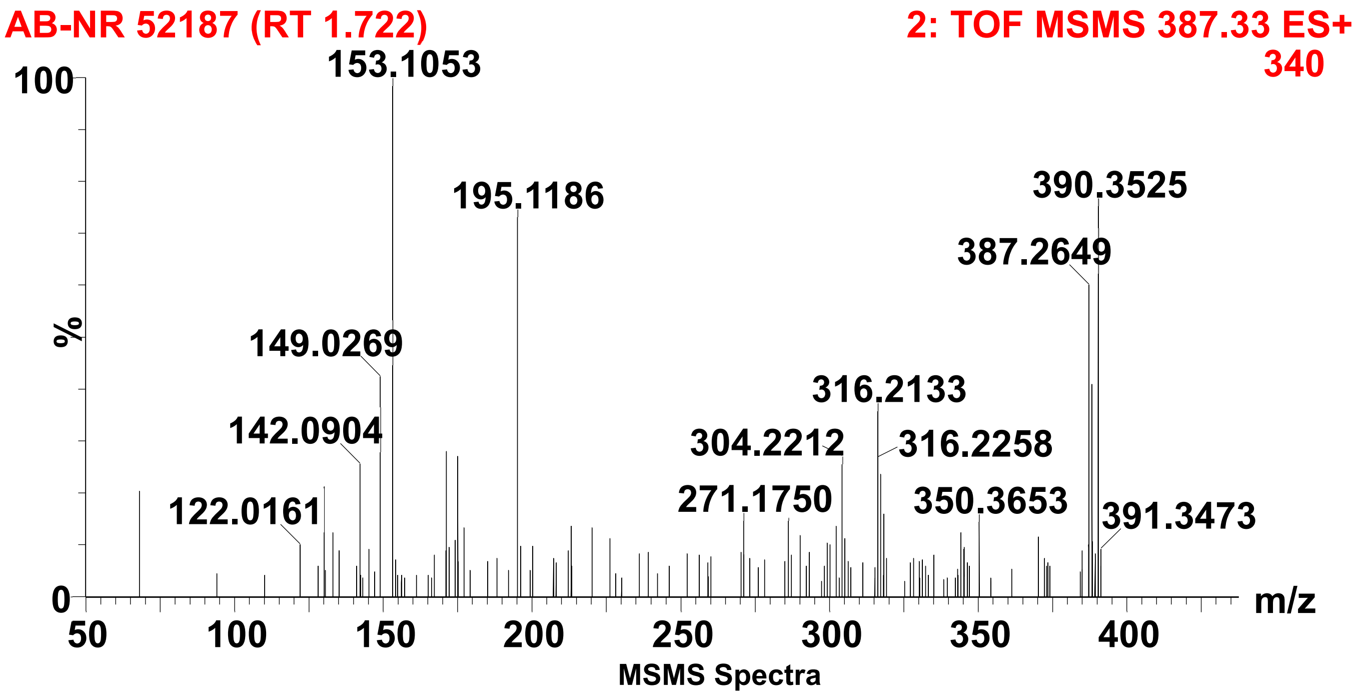

SI Figure 7. MSMS Spectra for compound ID 1.722_387.33.

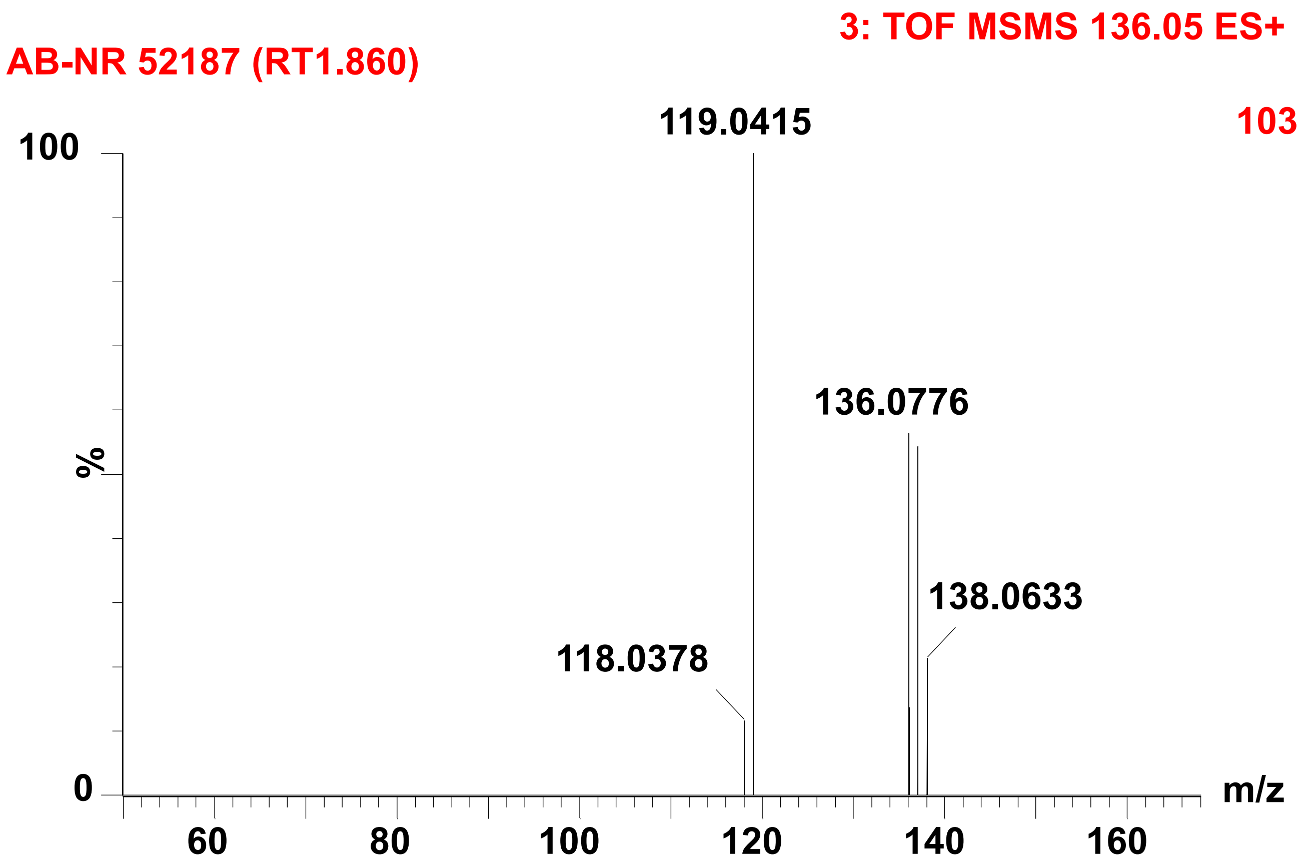

SI Figure 8. MSMS Spectra for compound ID 1.860_136.046.

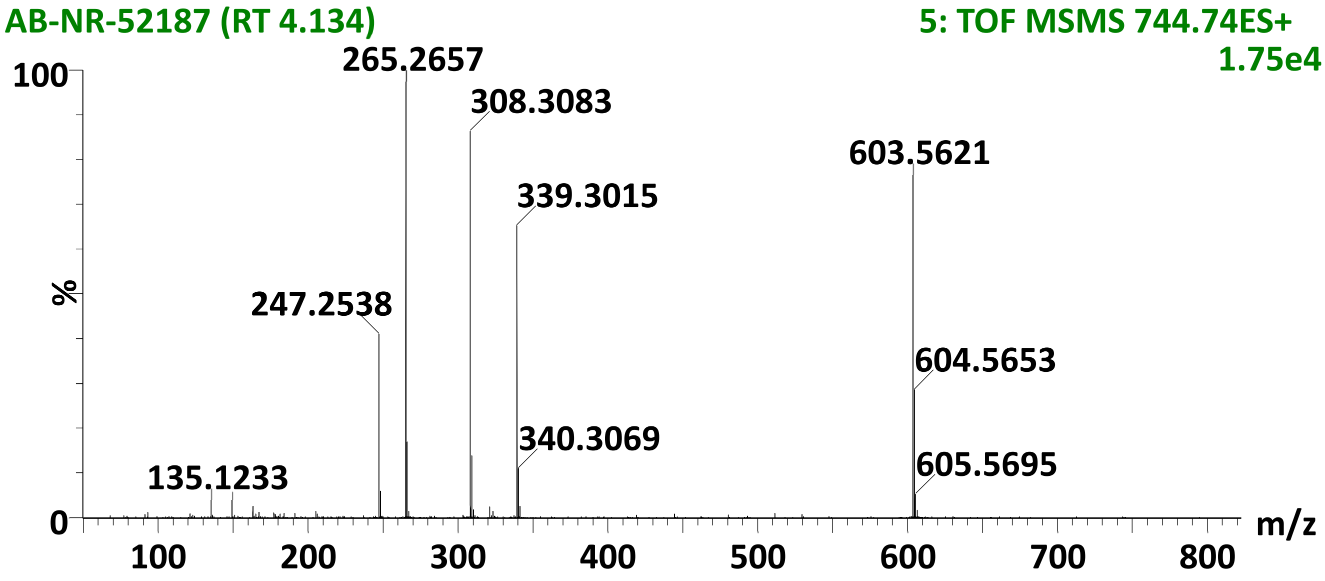

SI Figure 9. MSMS Spectra for compound ID 4.134_744.74.

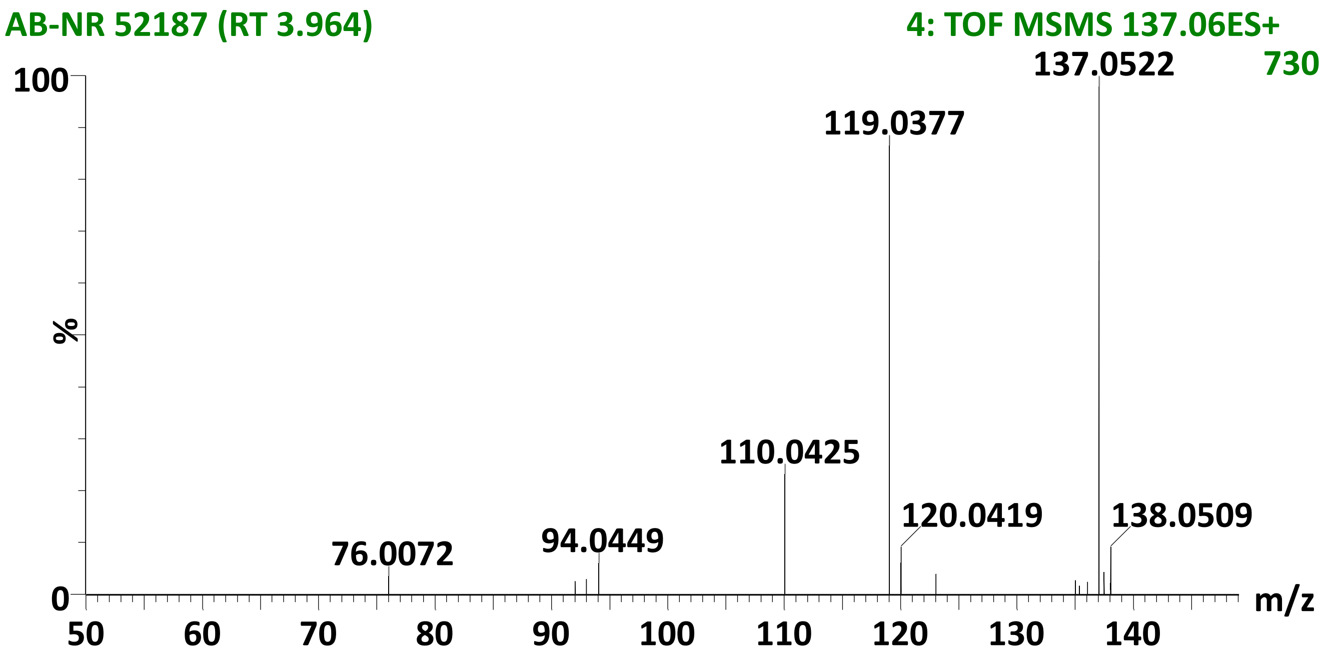

SI Figure 10. MSMS Spectra for compound ID 4.134_744.74.

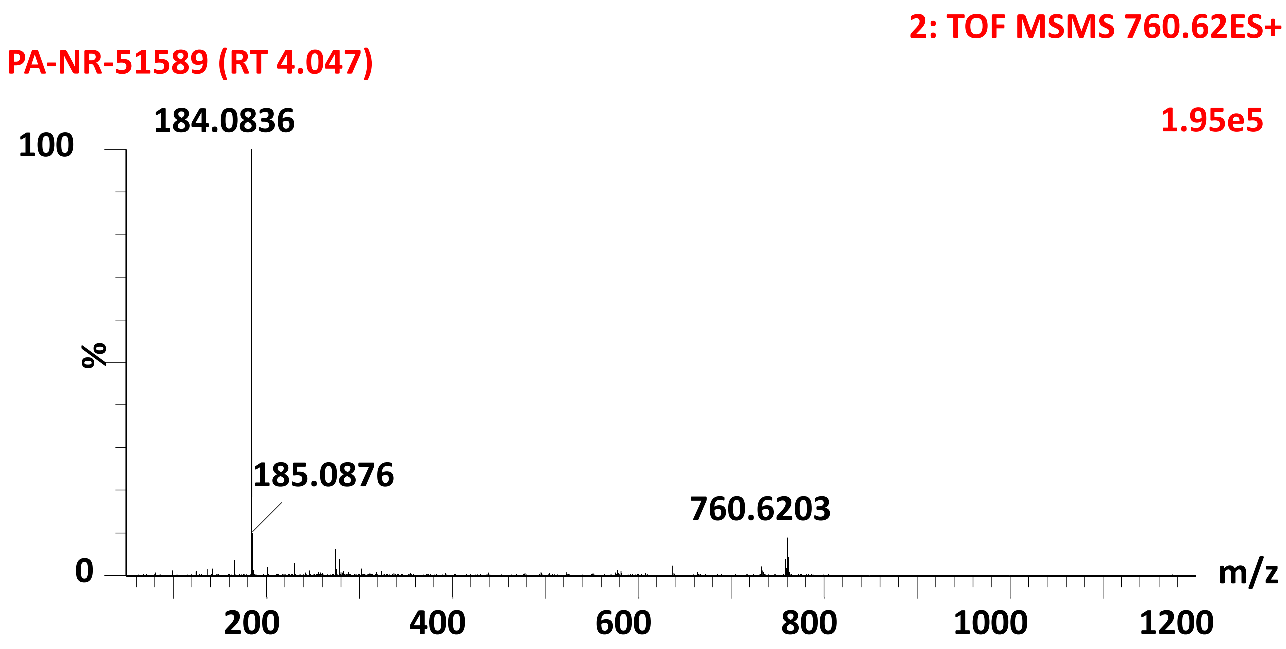

SI Figure 11. MSMS Spectra for PC 16:0/18:1.

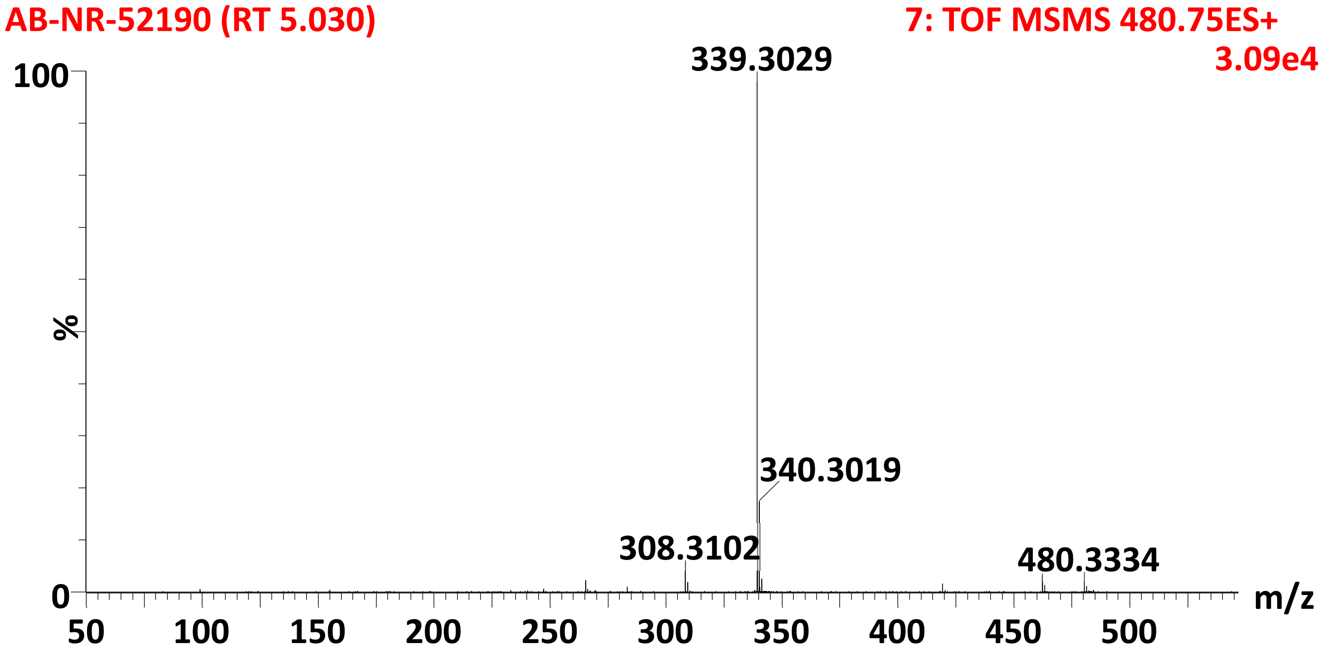

SI Figure 12. MSMS Spectra for compound ID 5.030_480.75.

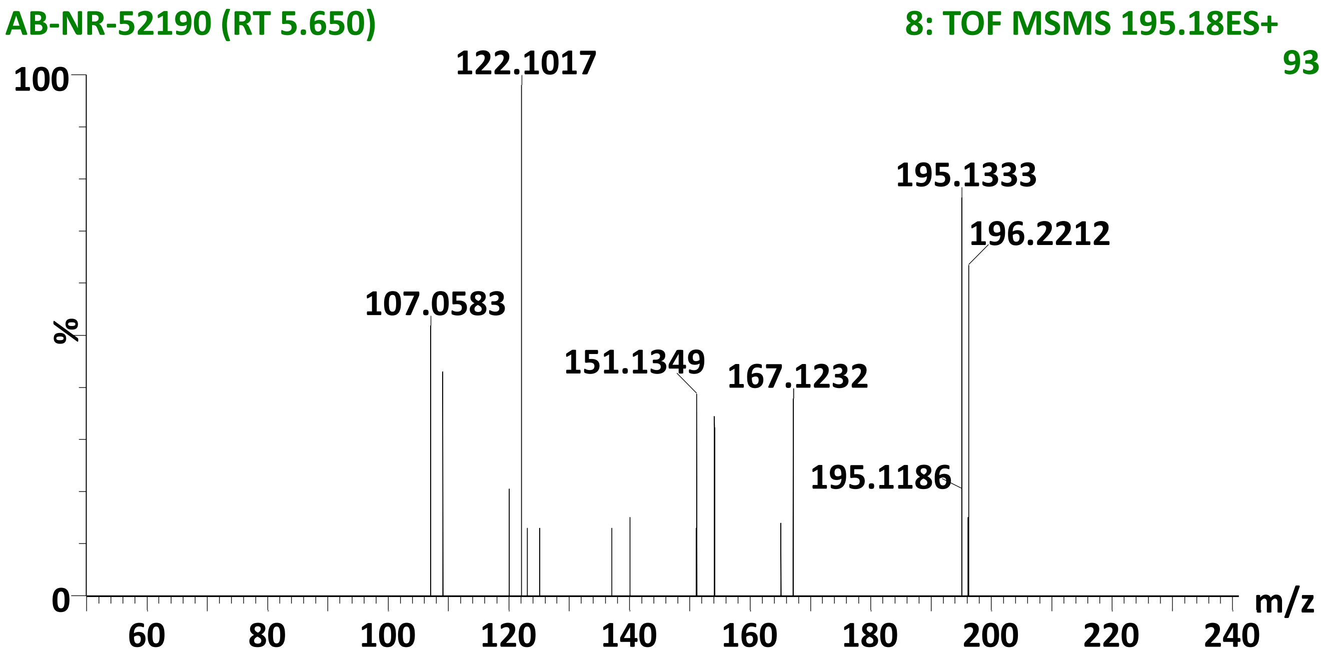

SI Figure 13. MSMS Spectra for compound ID 5.650_195.18.

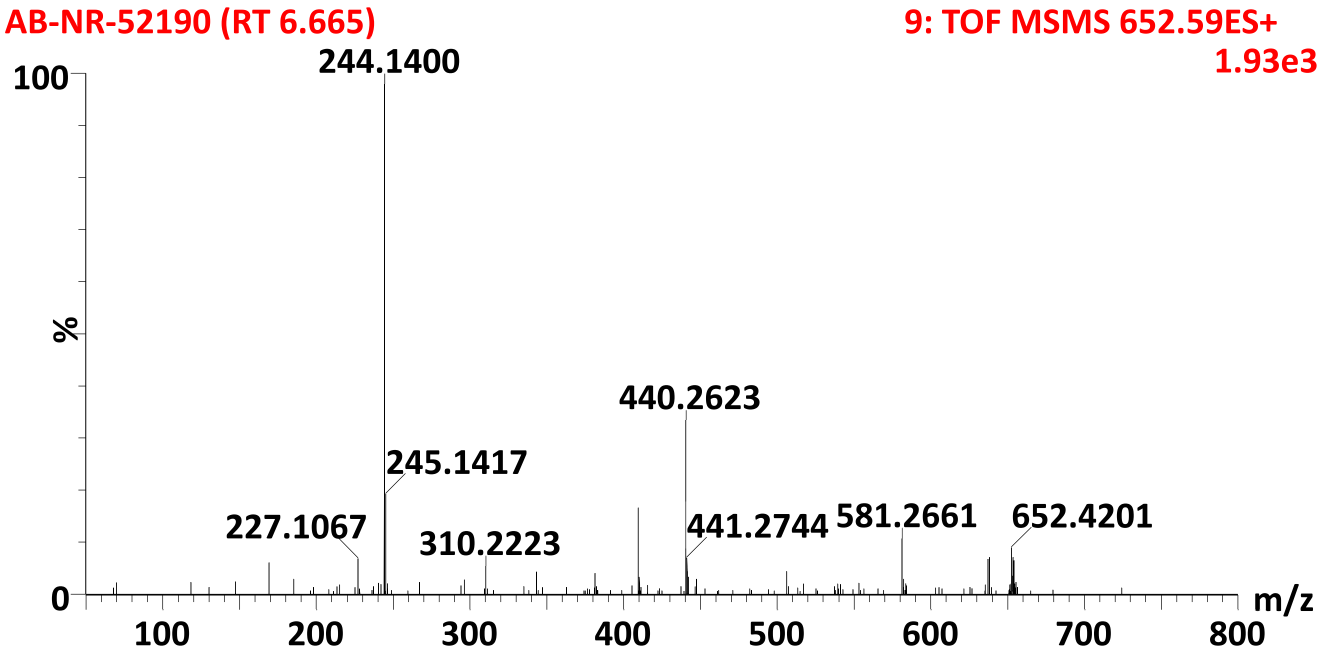

SI Figure 14. MSMS Spectra for compound ID 6.665_652.59.

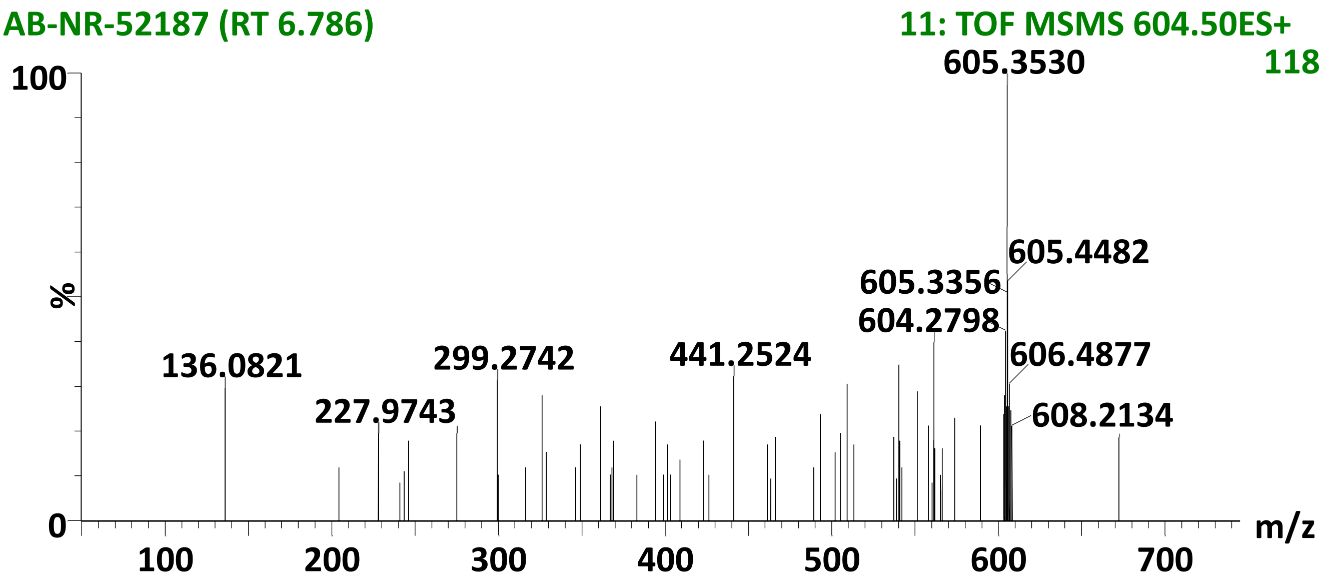

SI Figure 15. MSMS Spectra for compound ID 6.786_604.50.

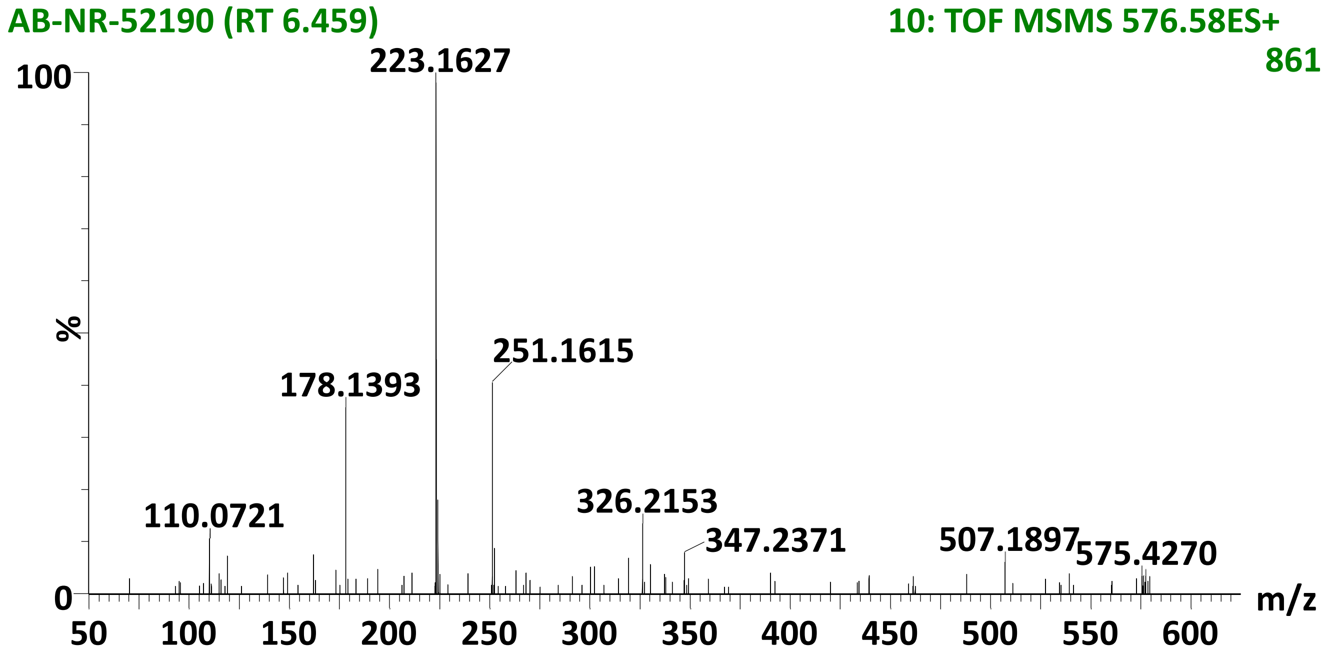

SI Figure 16. MSMS Spectra for compound ID 6.527_576.59.

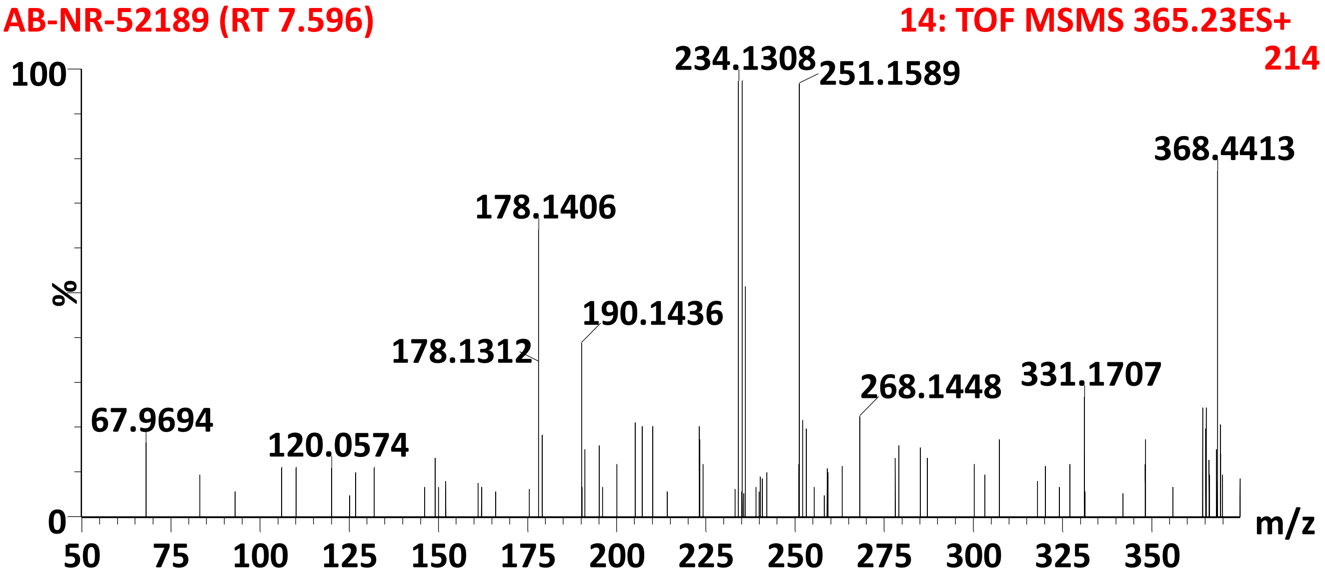

SI Figure 17. MSMS Spectra for compound ID 7.596_365.23.

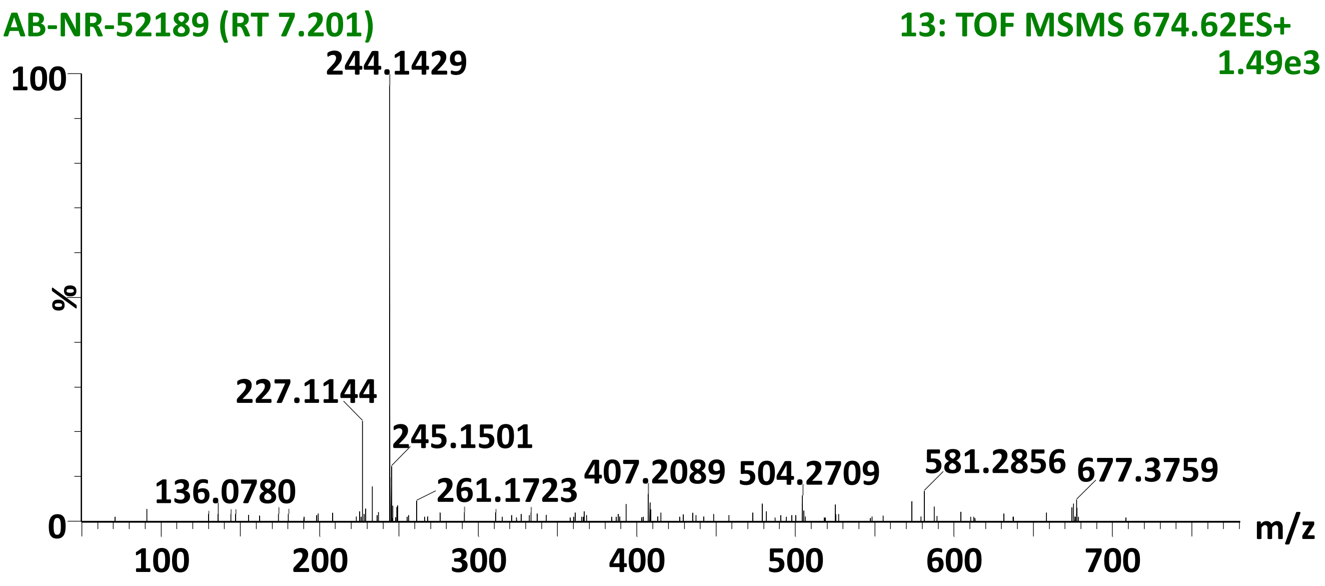

SI Figure 18. MSMS Spectra for compound ID 7.184_674.60.

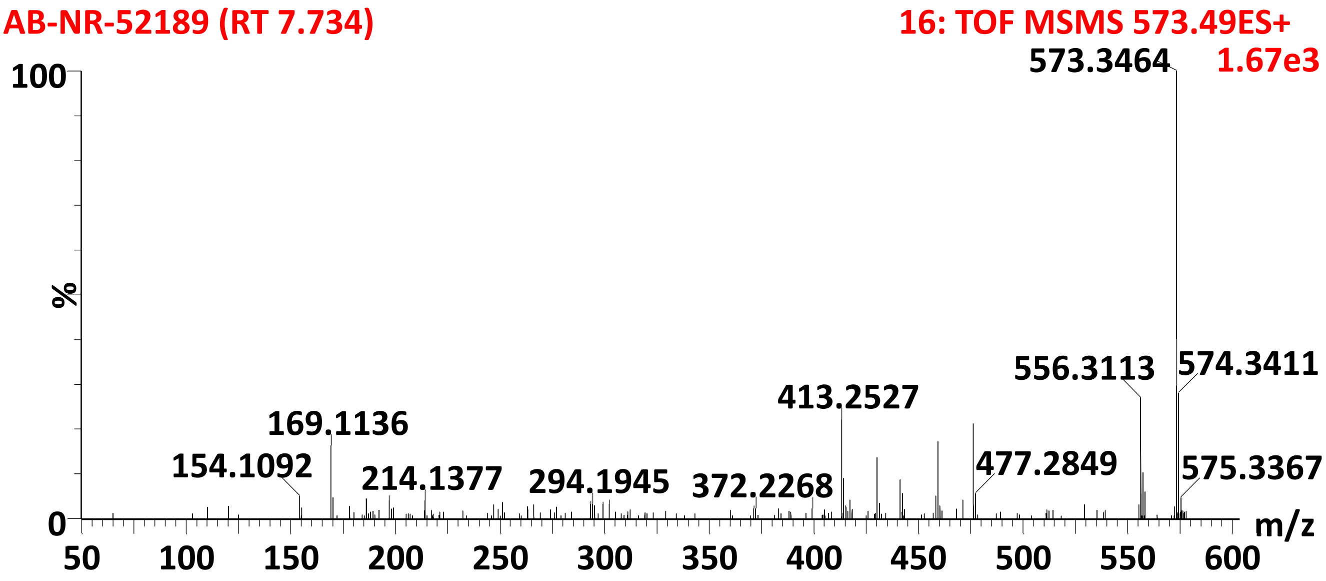

SI Figure 19. MSMS Spectra for compound ID 7.734_573.49.

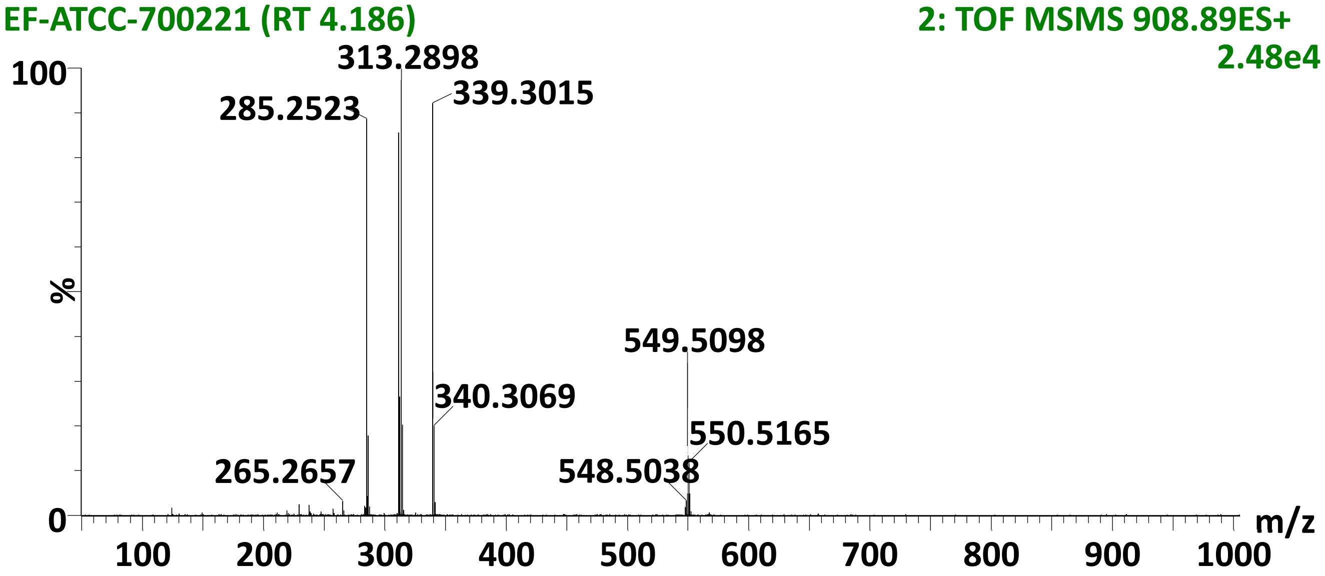

SI Figure 20. MSMS Spectra for compound ID 4.186_908.89.

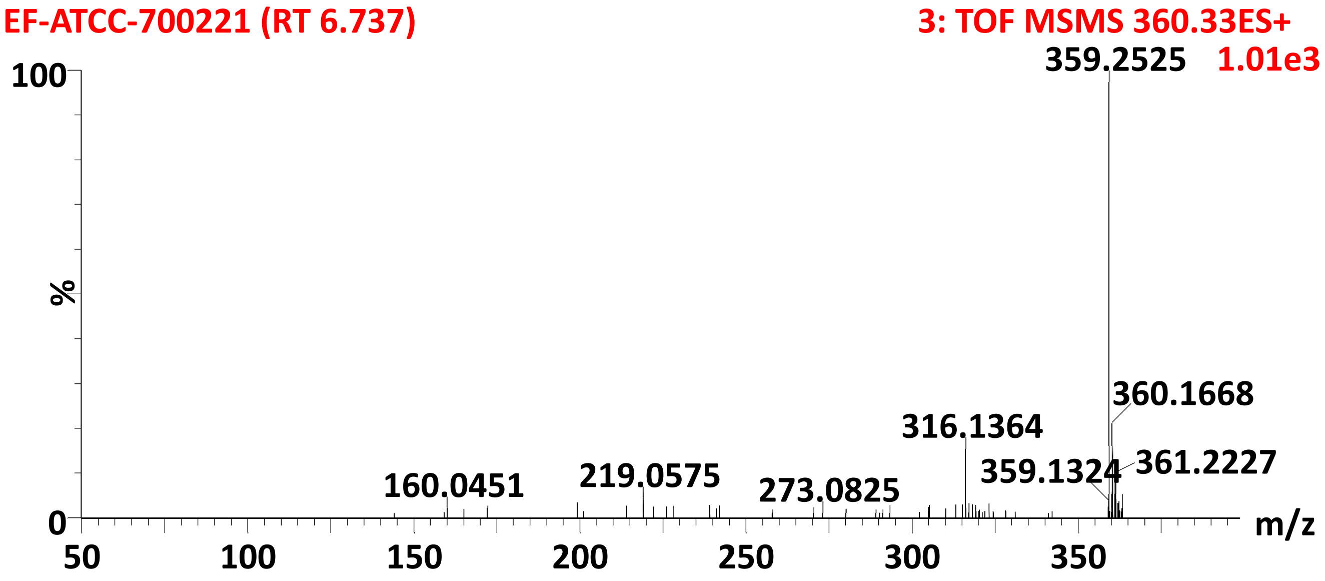

SI Figure 21. MSMS Spectra for compound ID 6.737_359.2525.

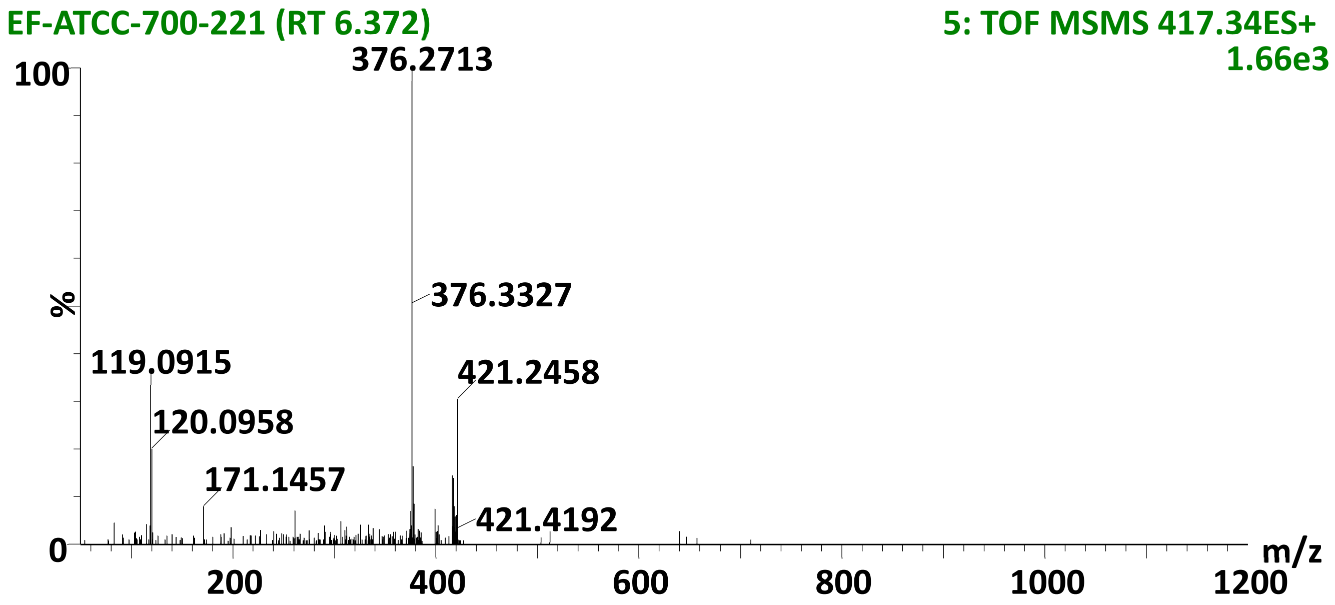

SI Figure 22. MSMS Spectra for compound ID 6.372_417.34.

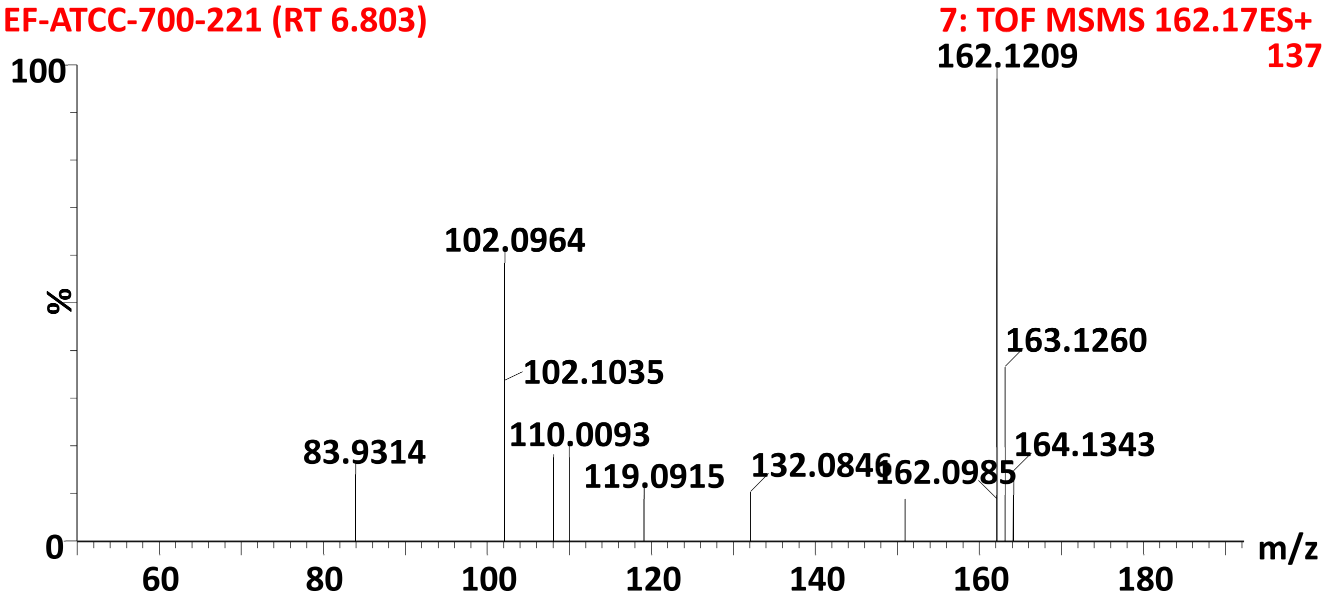

SI Figure 23. MSMS Spectra for compound ID 6.803_162.1209.

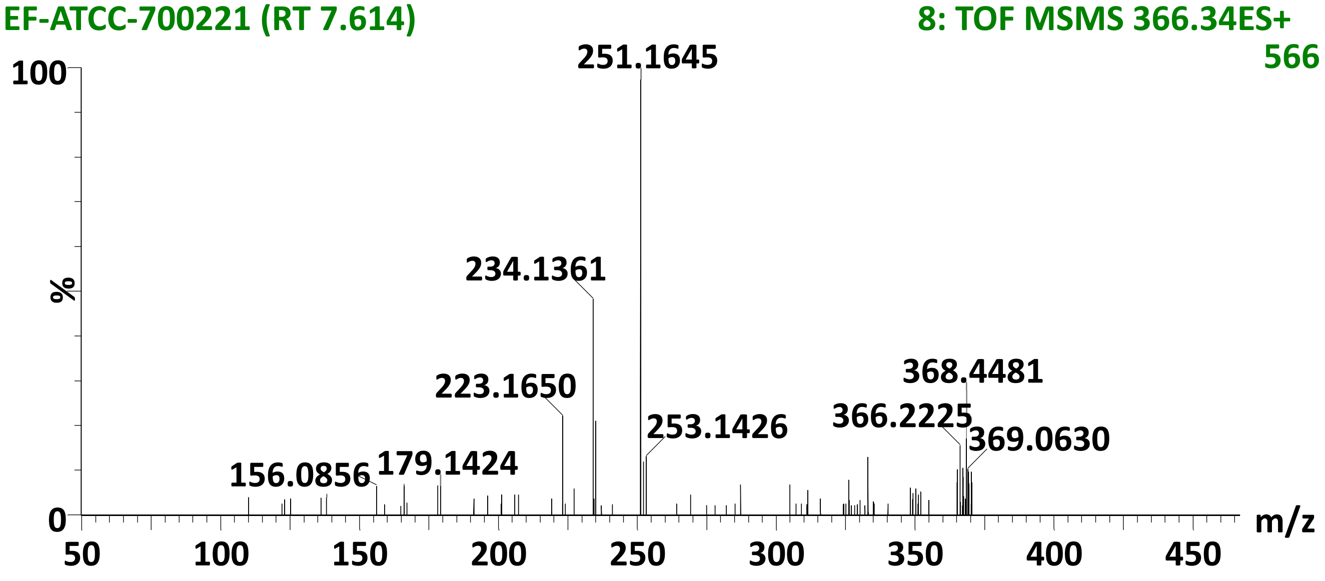

SI Figure 24. MSMS Spectra for compound ID 7.614 _366.34.

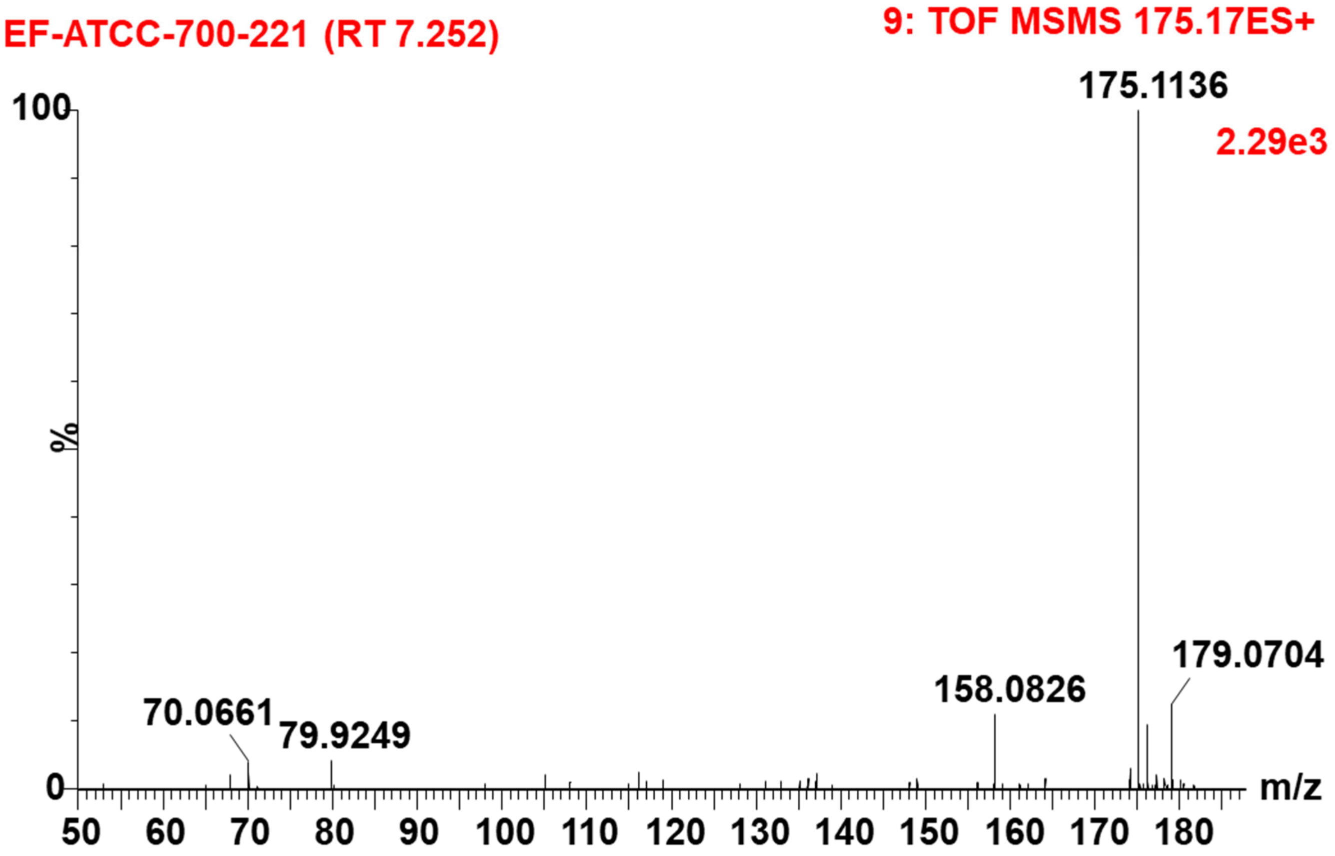

SI Figure 25. MSMS Spectra for compound ID 7.252 _175.17.

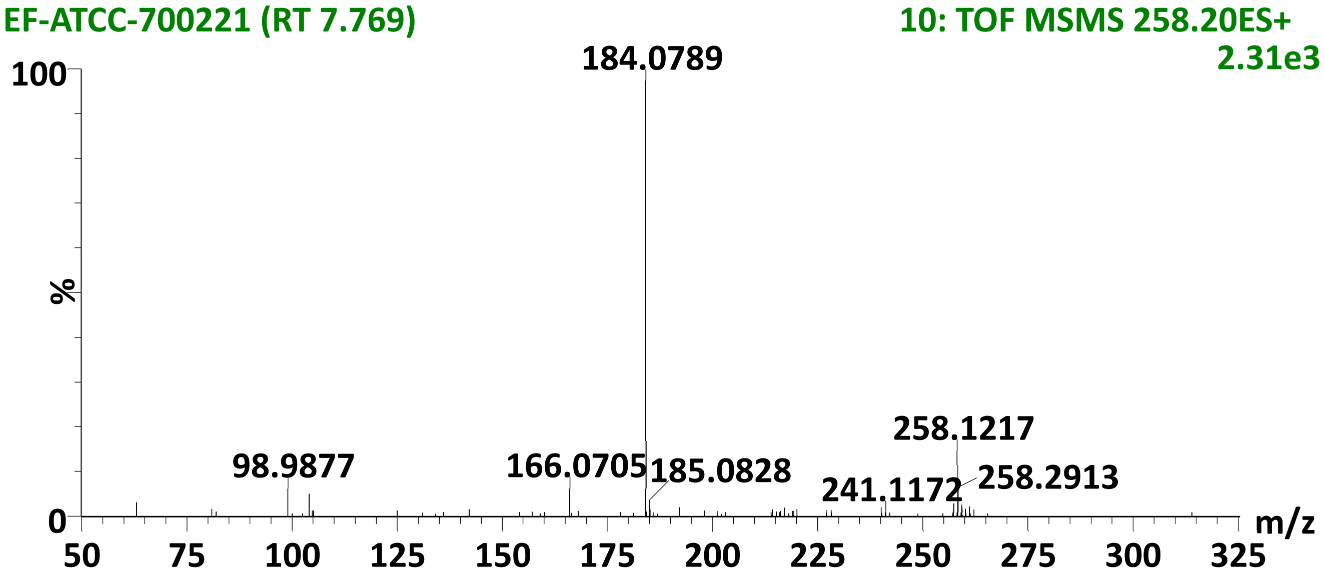

SI Figure 26. MSMS Spectra for compound ID 7.769 _258.20.

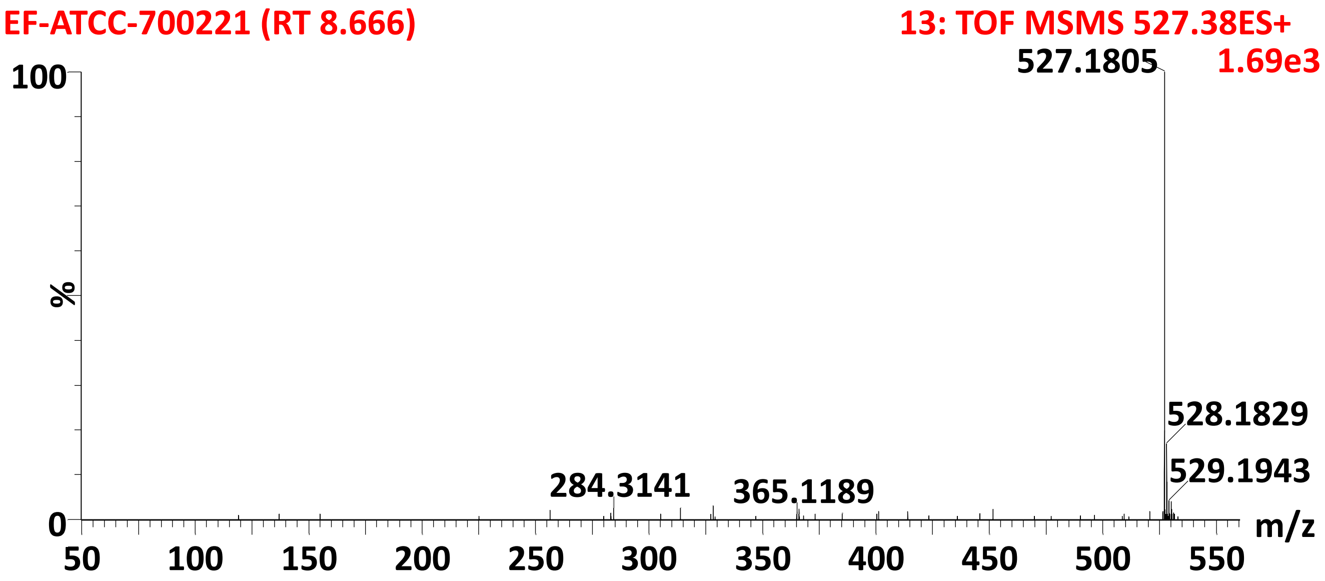

SI Figure 27. MSMS Spectra for compound ID 8.6 _527.18.

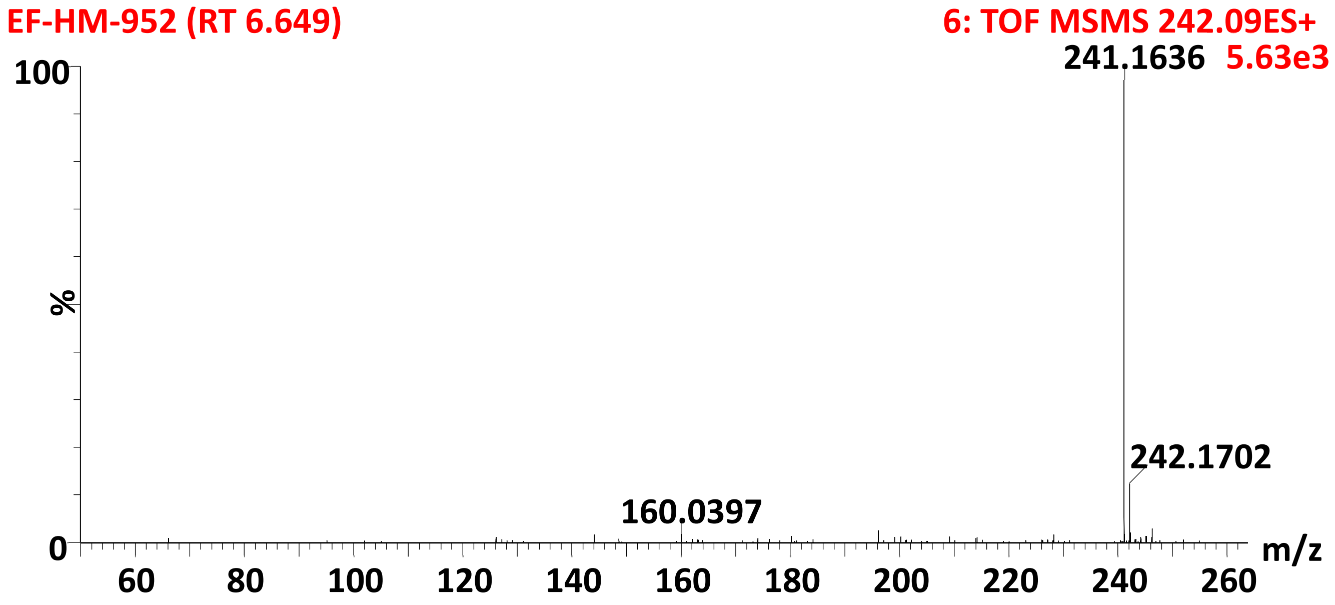

SI Figure 28. MSMS Spectra for compound ID 6.649 _242.09.

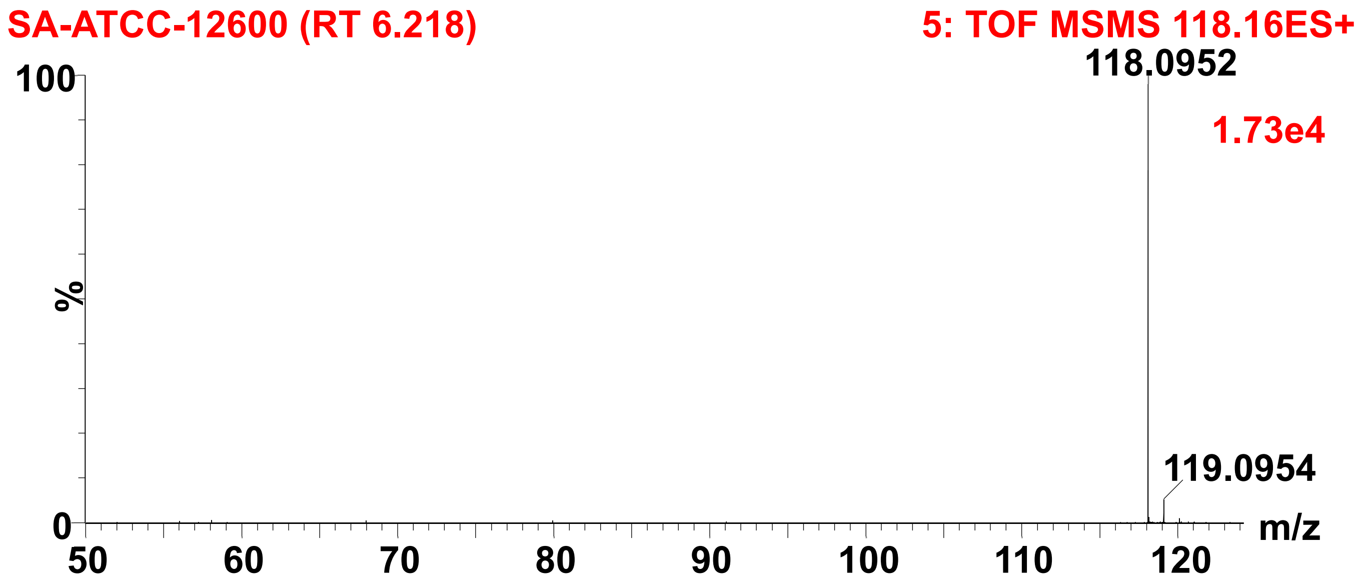

SI Figure 29. MSMS Spectra for compound ID 6.218 _118.0952.

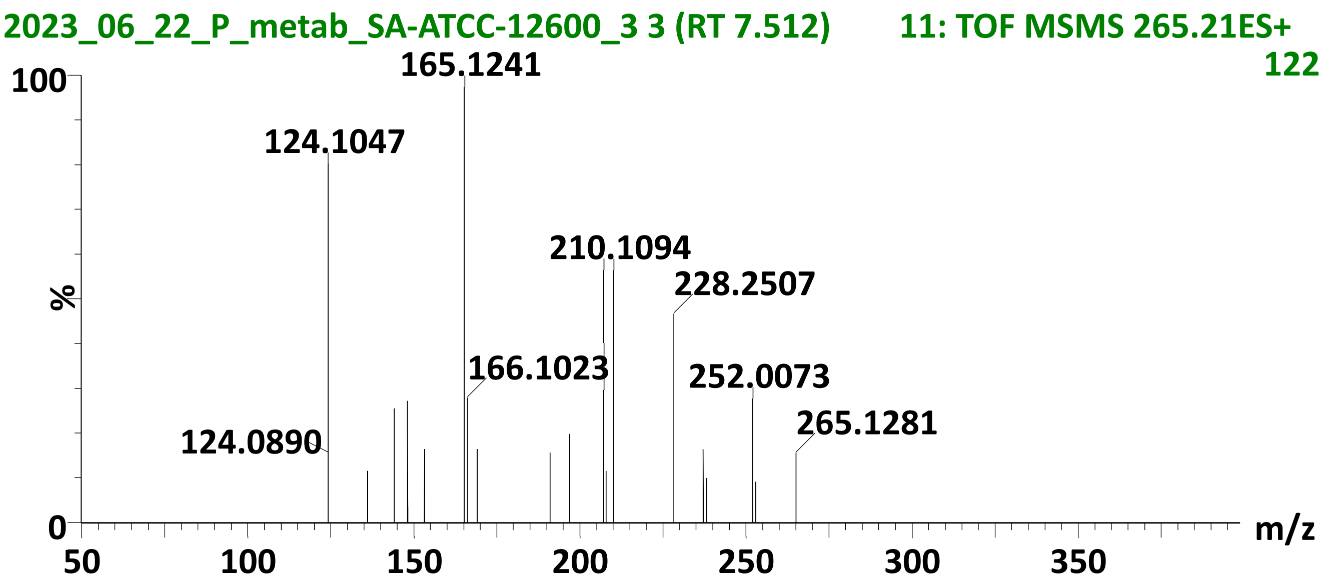

SI Figure 30. MSMS Spectra for compound ID 7.512 _265.21.

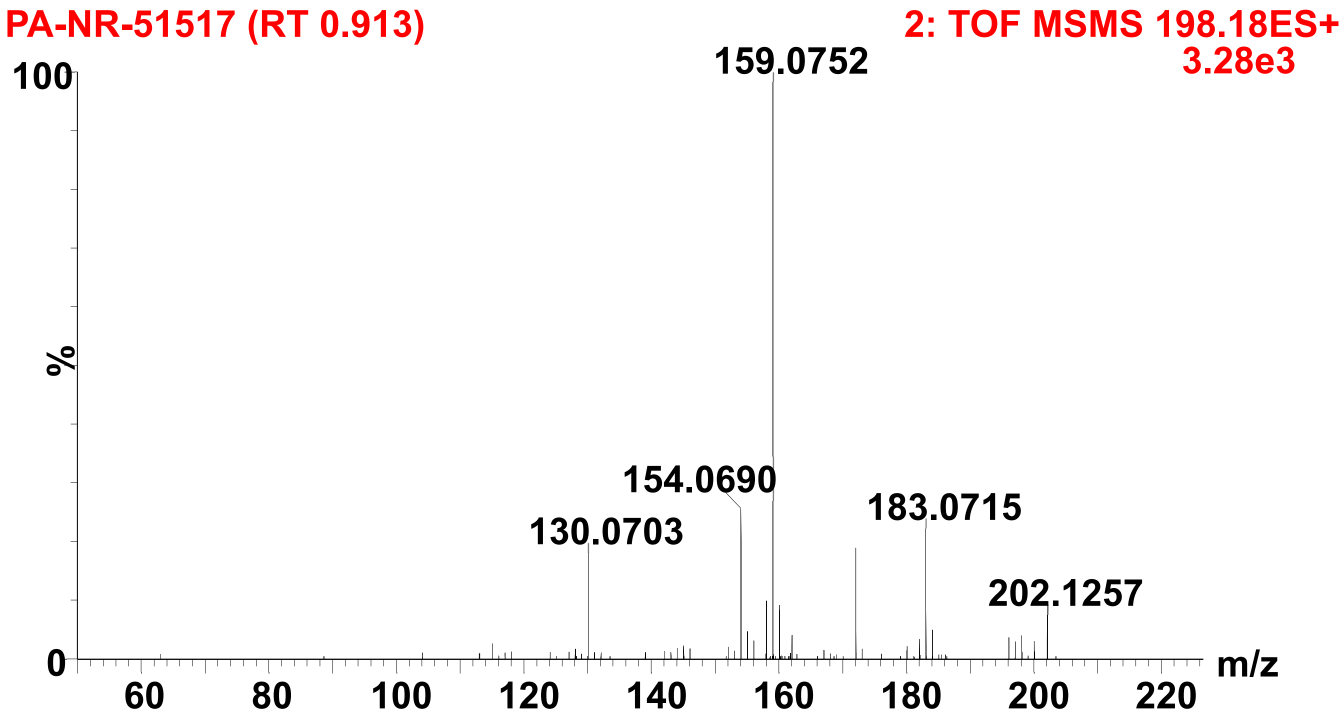

SI Figure 31. MSMS Spectra for compound ID 0.913 _198.18.

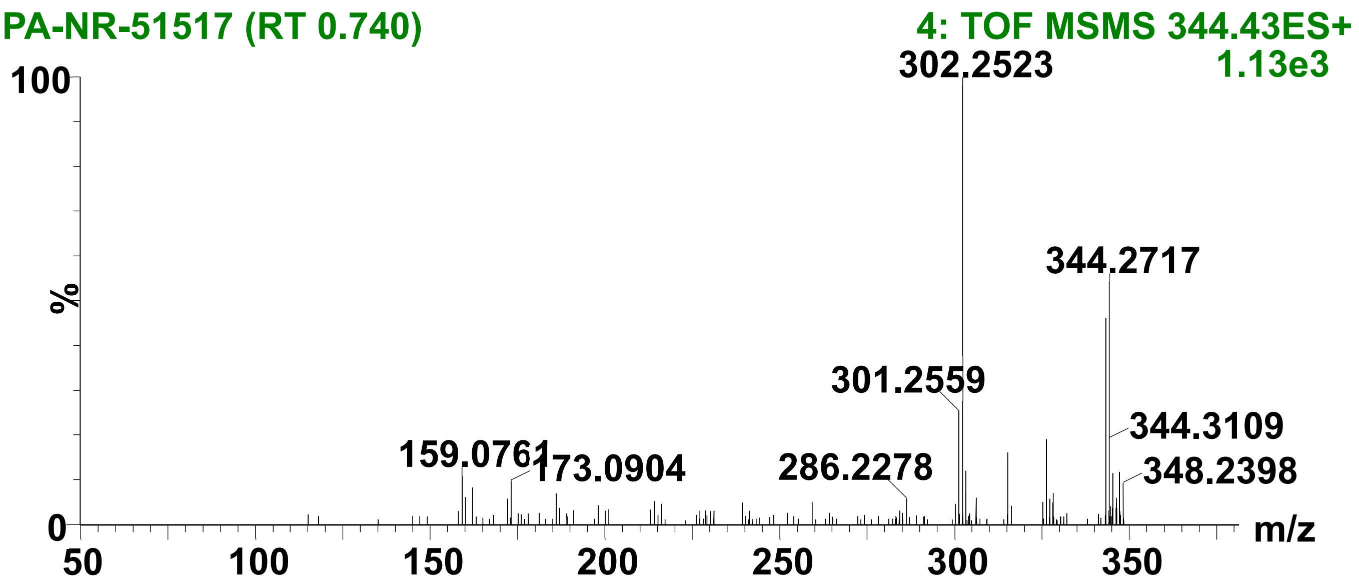

SI Figure 32. MSMS Spectra for compound ID 0.740 _344.43.

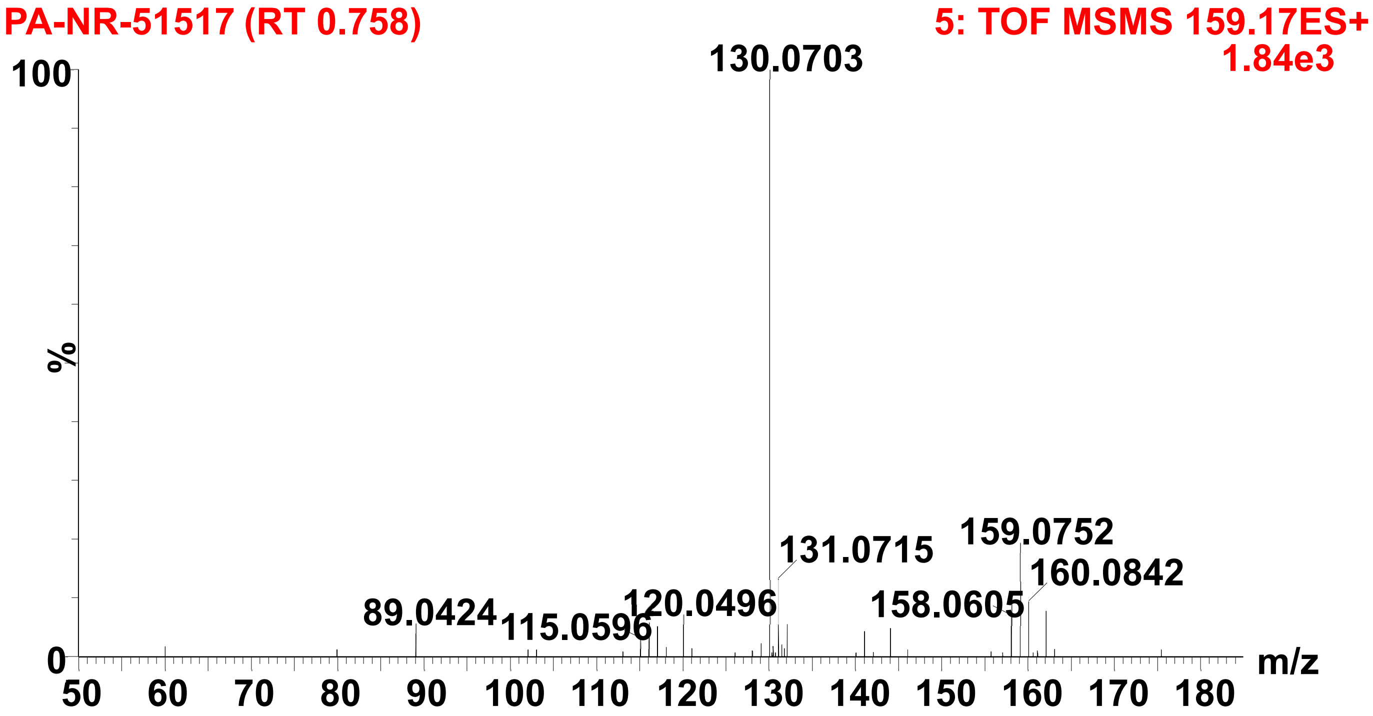

SI Figure 33. MSMS Spectra for compound ID 0.758 _159.17.

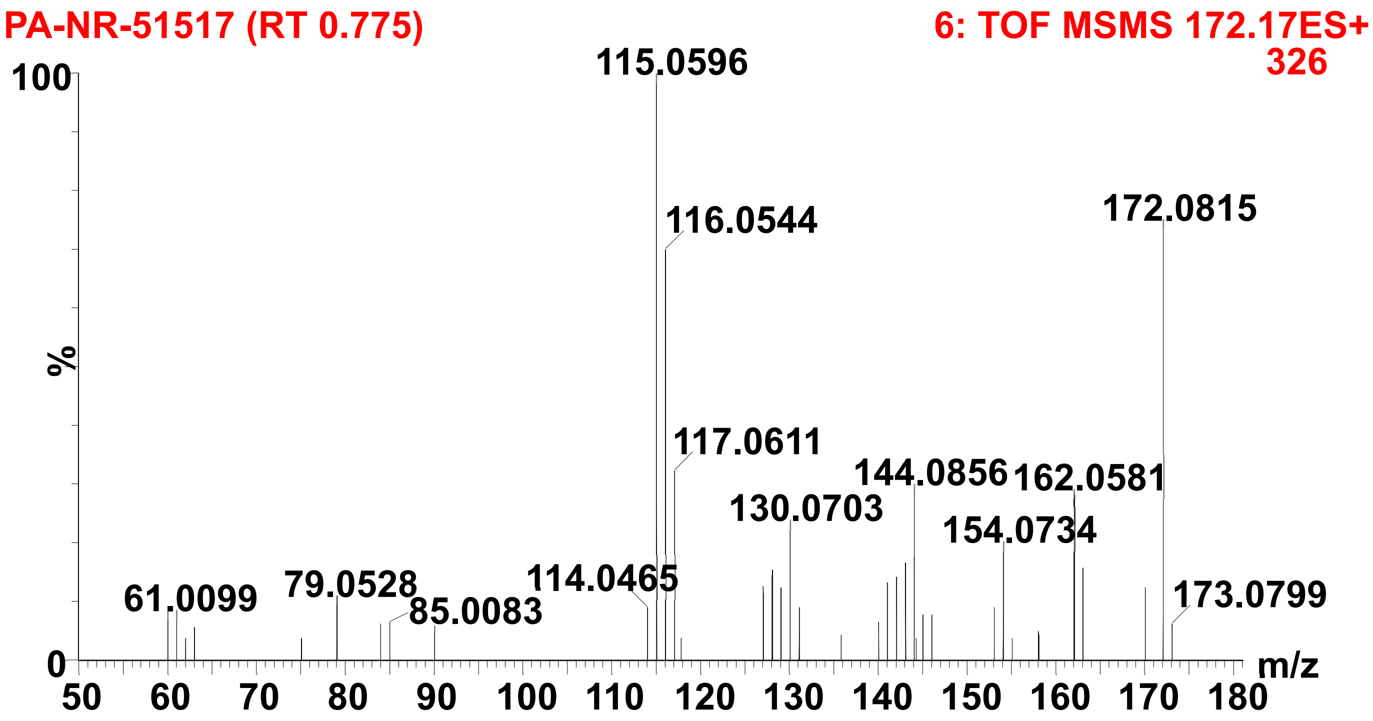

SI Figure 34. MSMS Spectra for compound ID 0.775 _172.17.

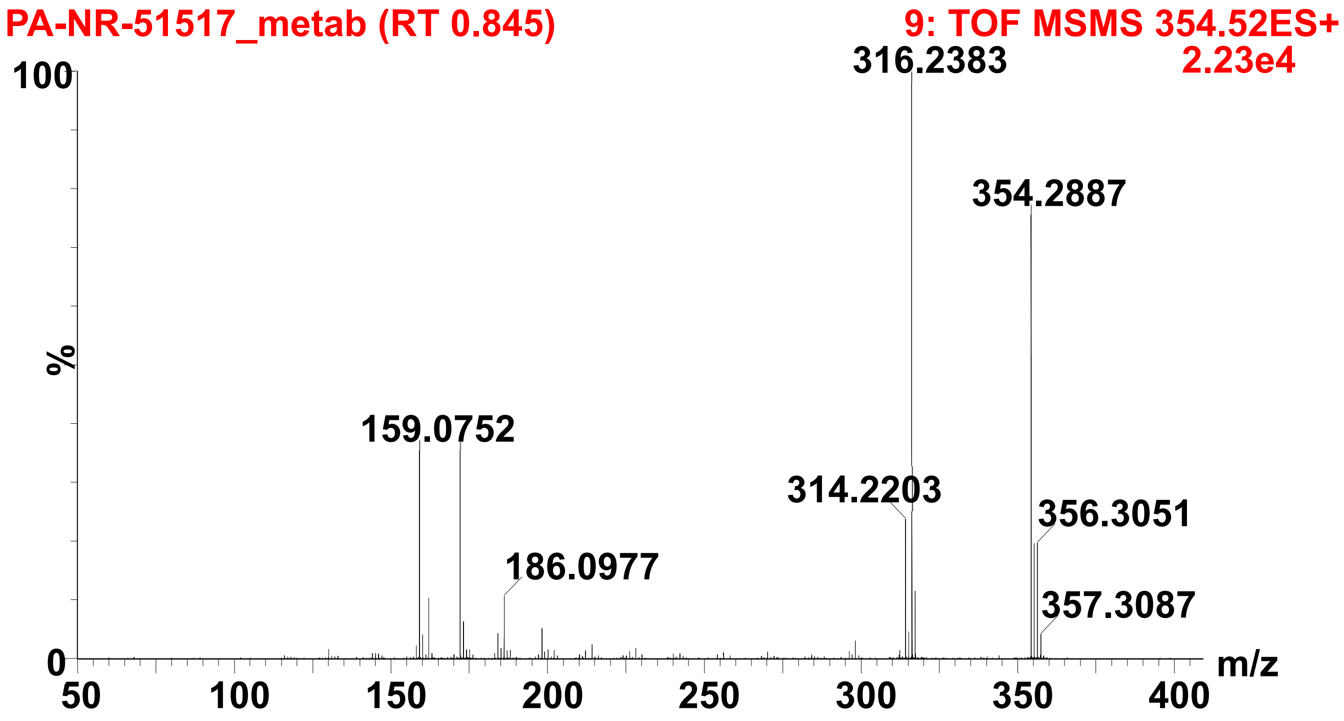

SI Figure 35. MSMS Spectra for compound ID 0.845 _354.52.

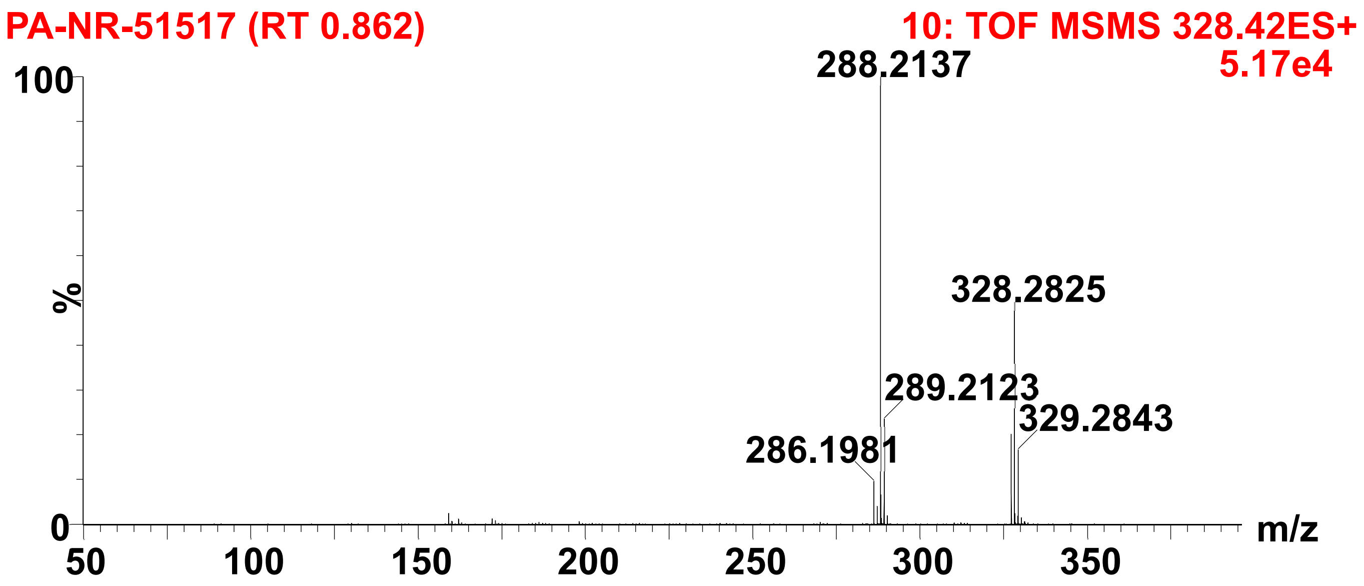

SI Figure 36. MSMS Spectra for compound ID 0.862 _328.42.

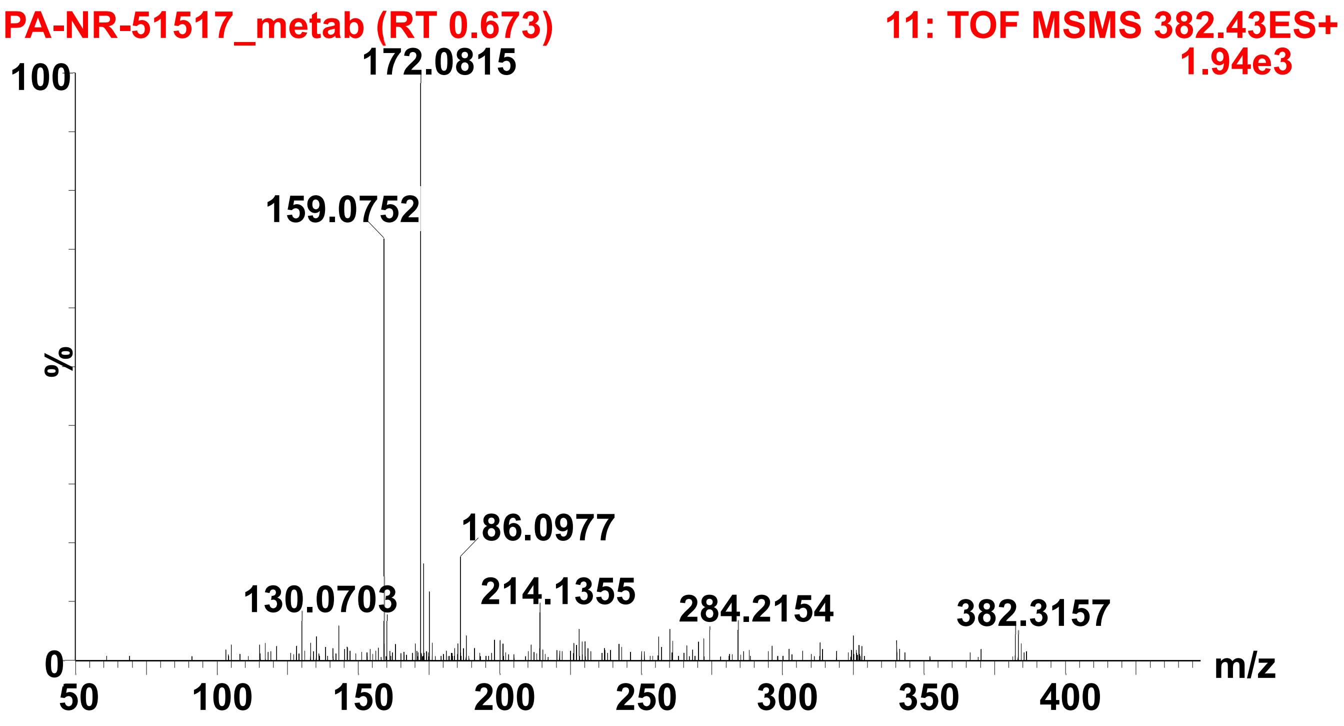

SI Figure 37. MSMS Spectra for compound ID 0.673_382.43.

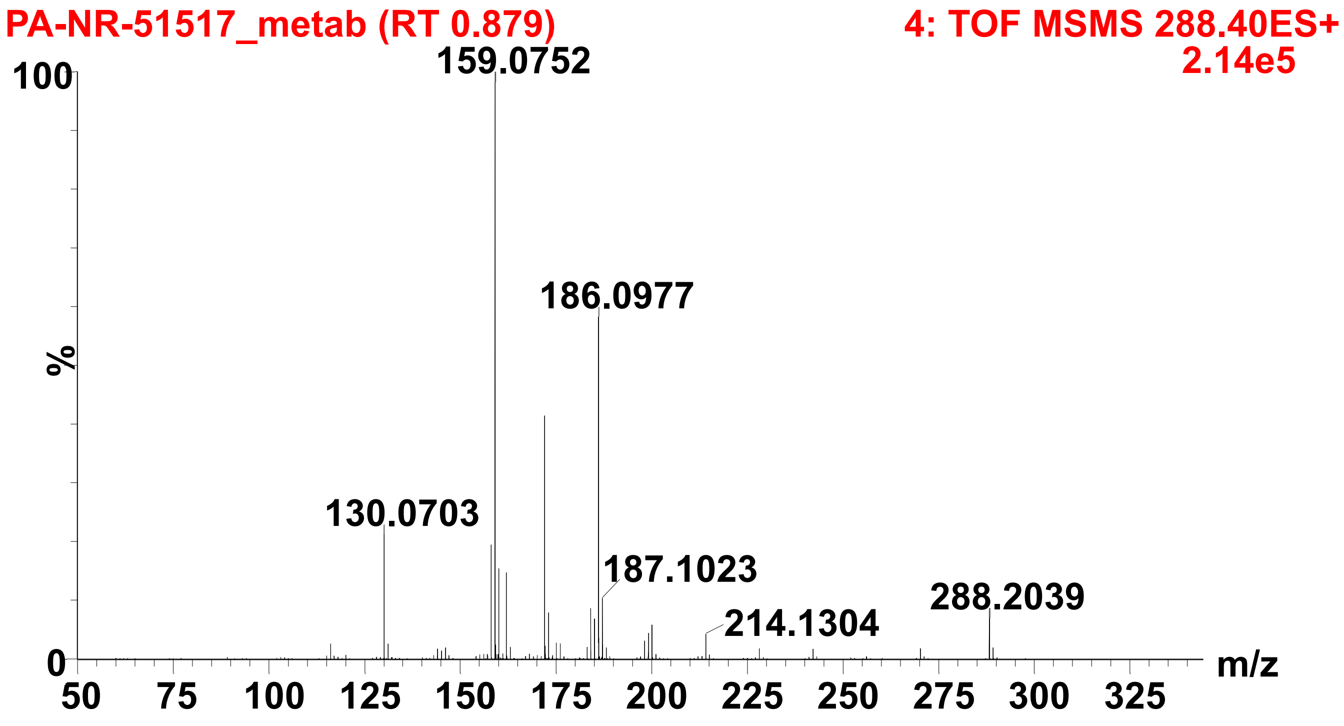

SI Figure 38. MSMS Spectra for compound ID 0.879_288.40.

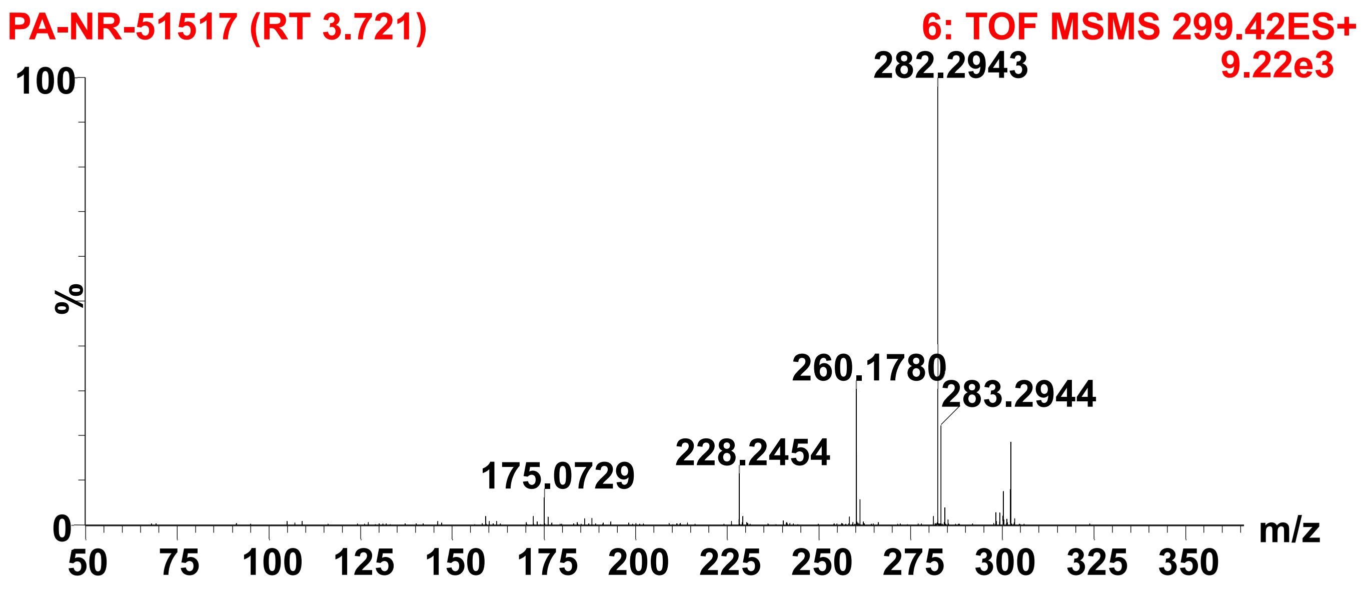

SI Figure 39. MSMS Spectra for compound ID 3.721_299.42.

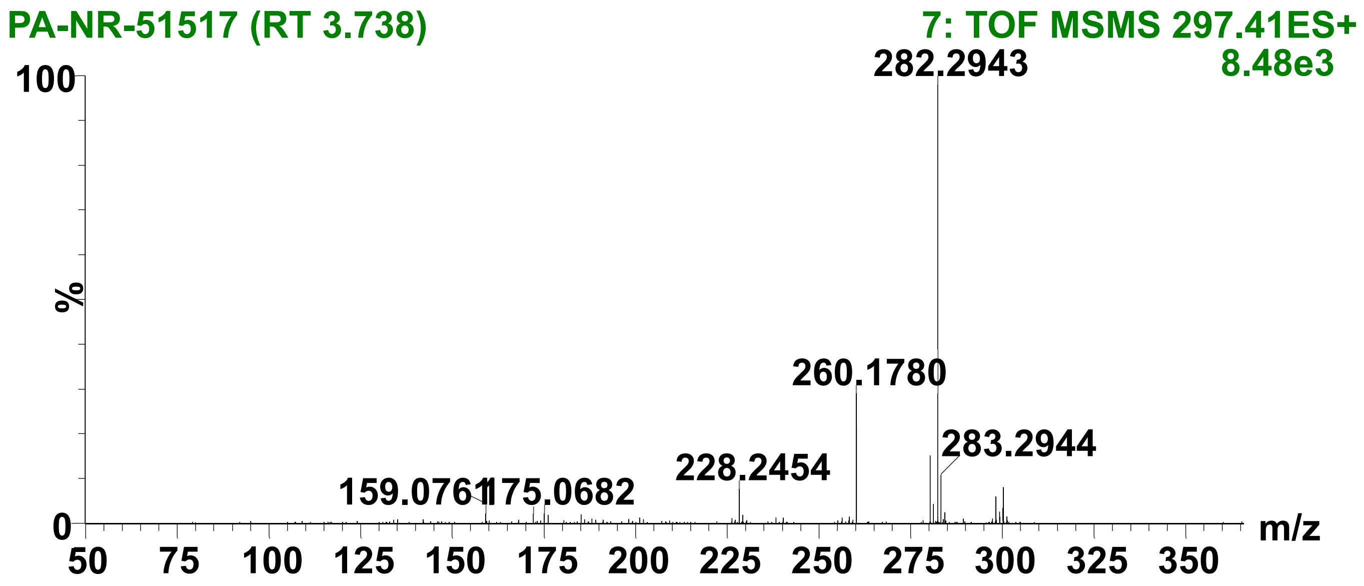

SI Figure 40. MSMS Spectra for compound ID 3.738_297.41.

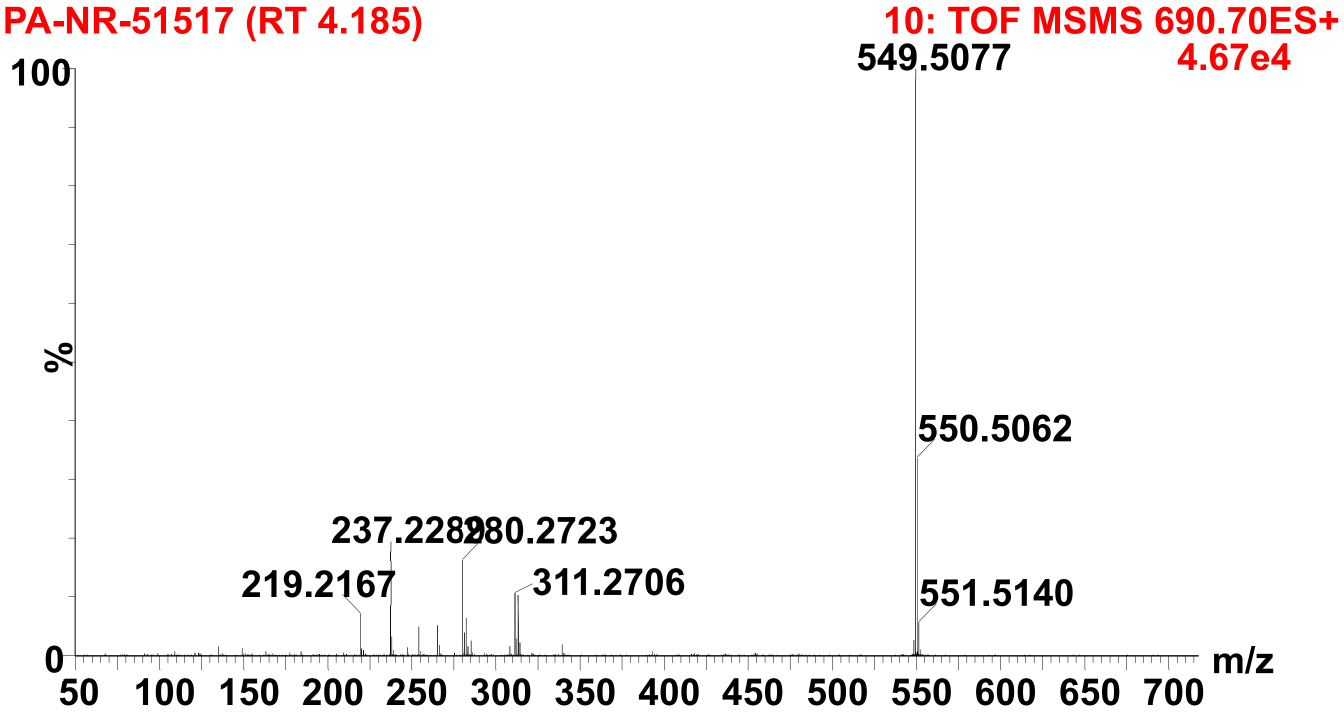

SI Figure 41. MSMS Spectra for compound ID 4.185_690.70.

Negative Mode MSMS Spectra

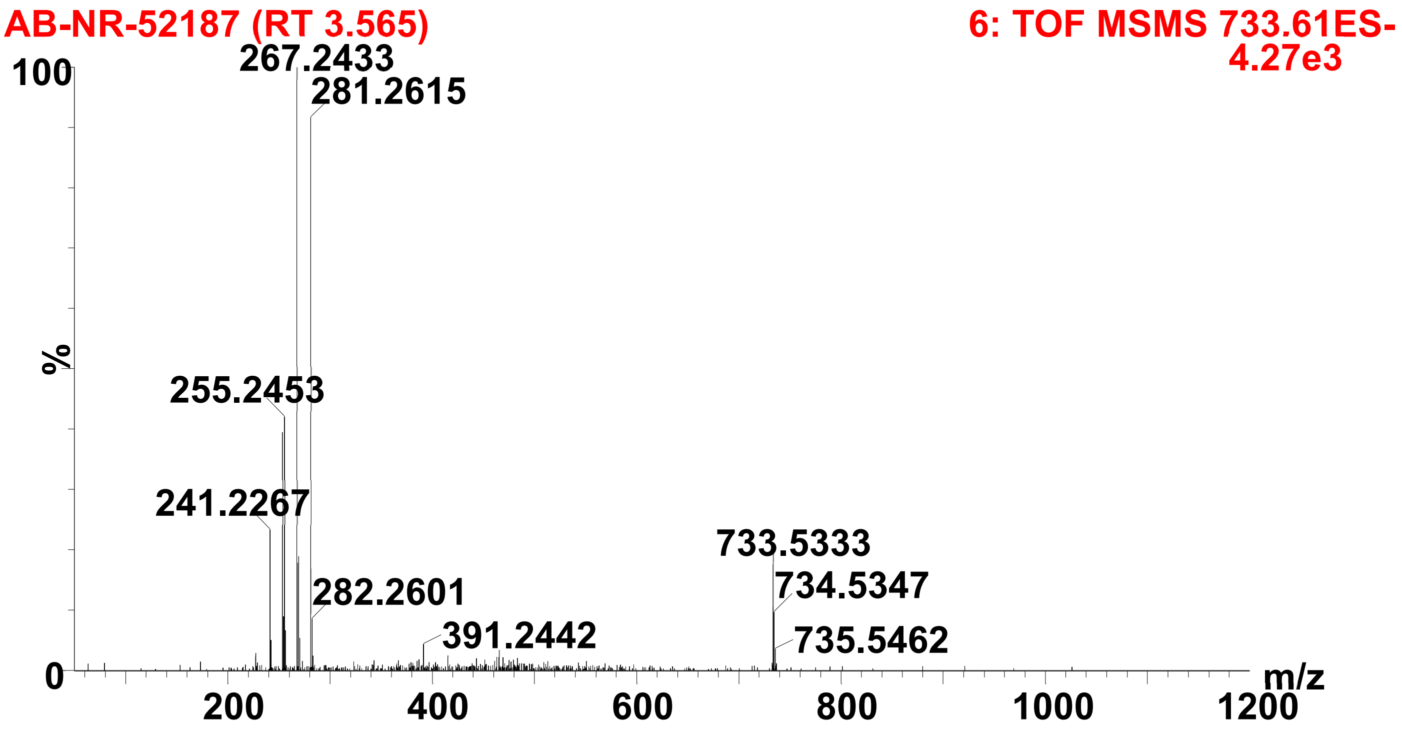

SI Figure 42. MSMS spectra of compound ID 3.565_733.61.

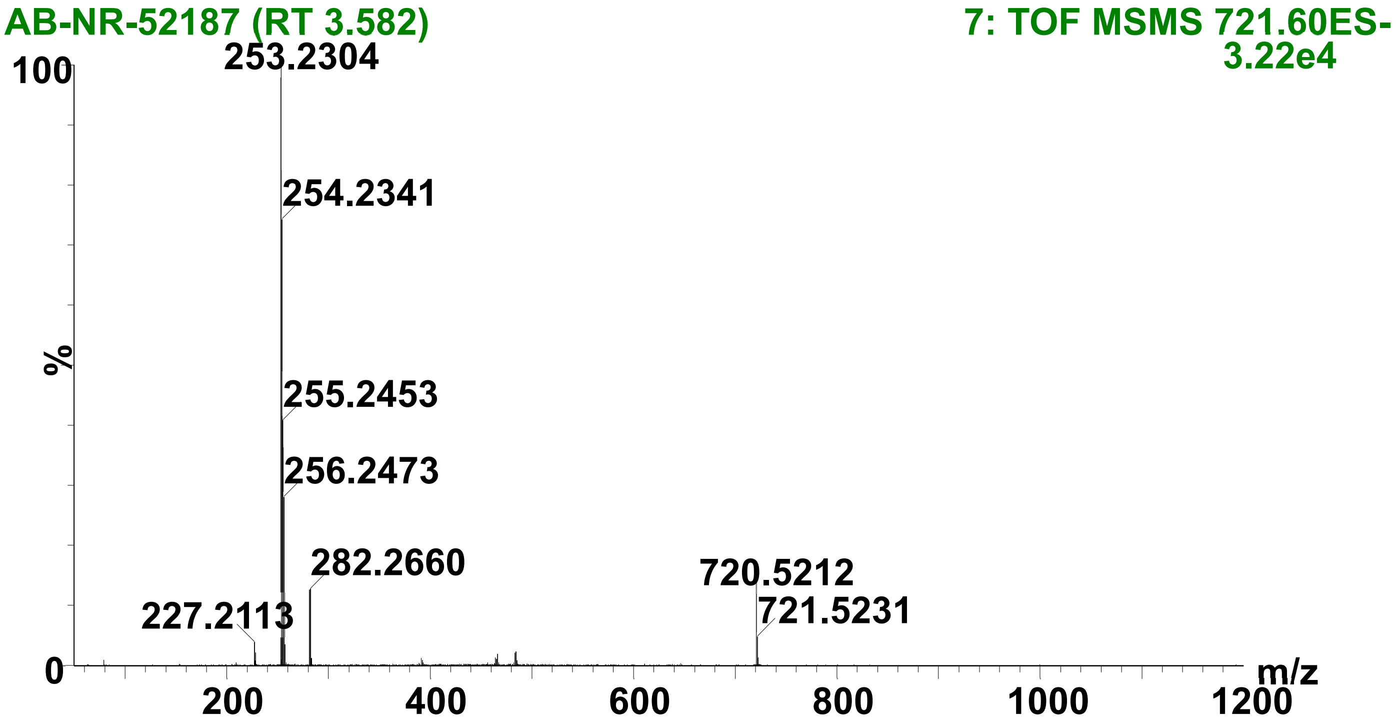

SI Figure 43. MSMS spectra of compound ID 3.586_721.60.

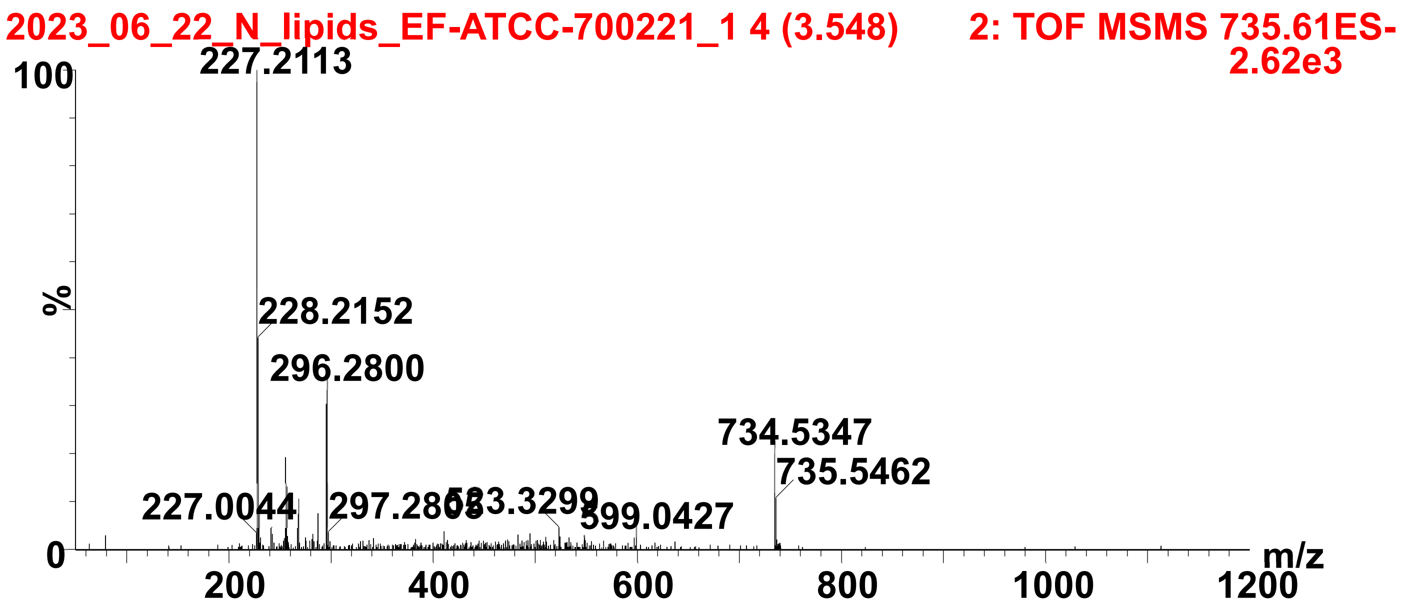

SI Figure 44. MSMS spectra of compound ID 3.548_735.61.

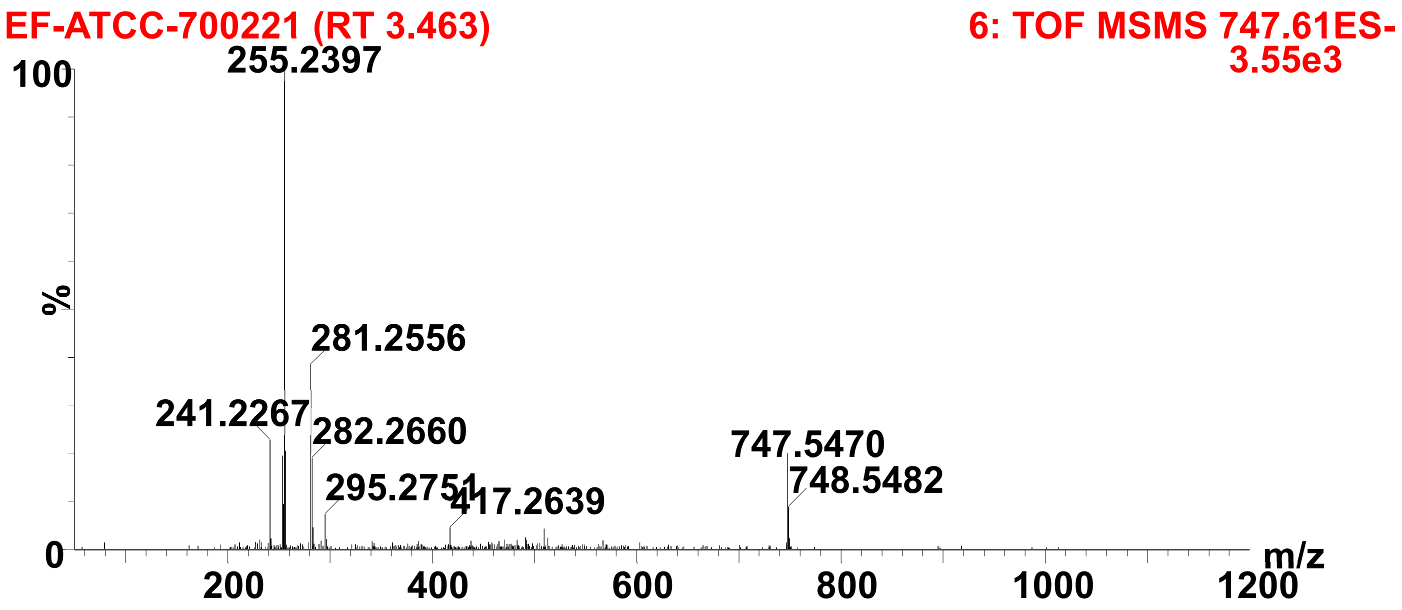

SI Figure 45. MSMS spectra of compound ID 3.463_747.61.

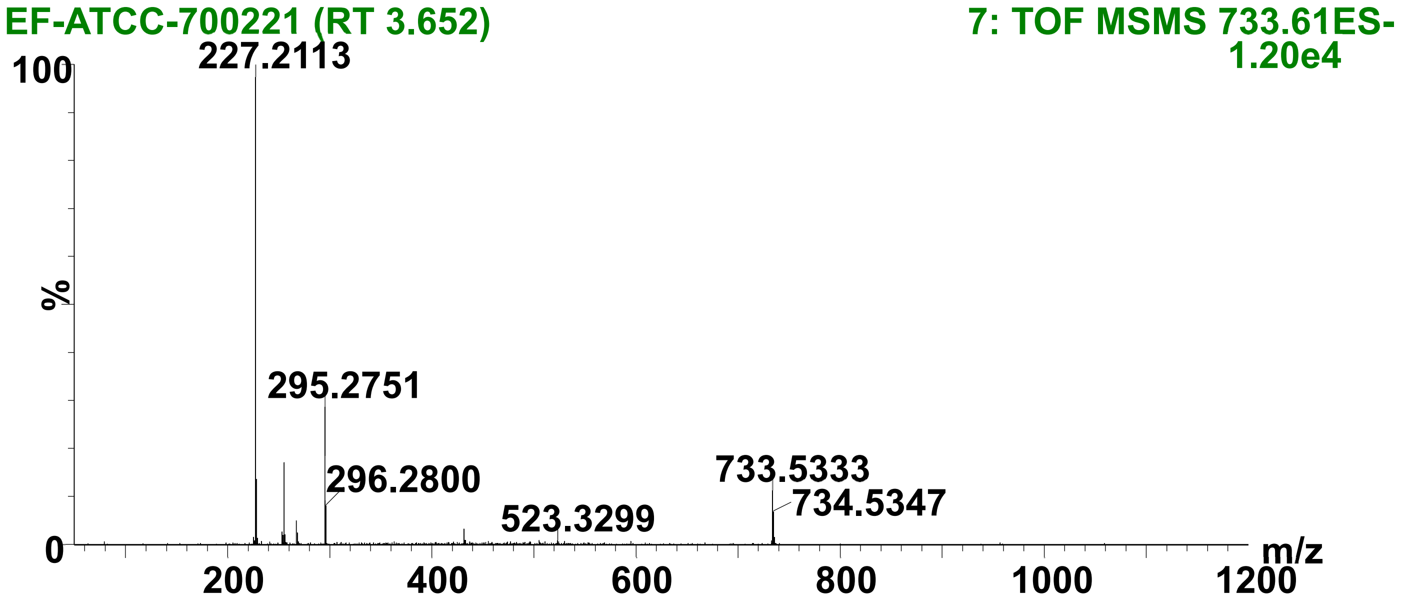

SI Figure 46. MSMS spectra of compound ID 3.652_733.61.

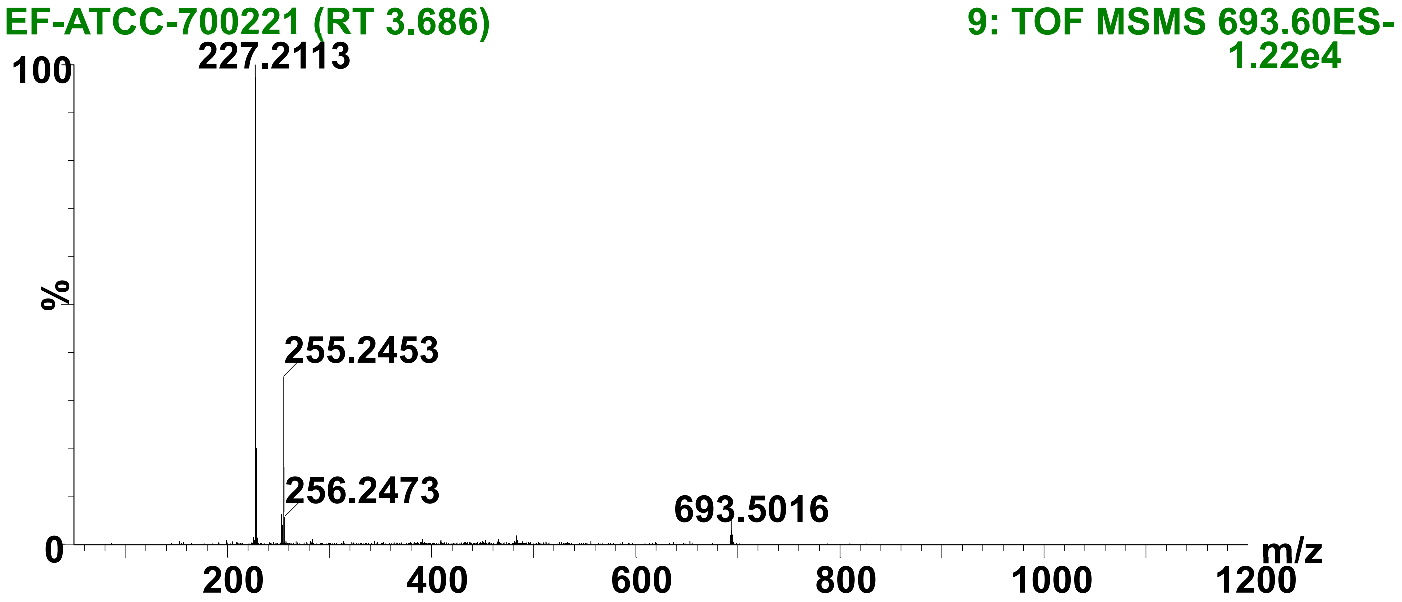

SI Figure 47. MSMS spectra of compound ID 3.686_693.60.

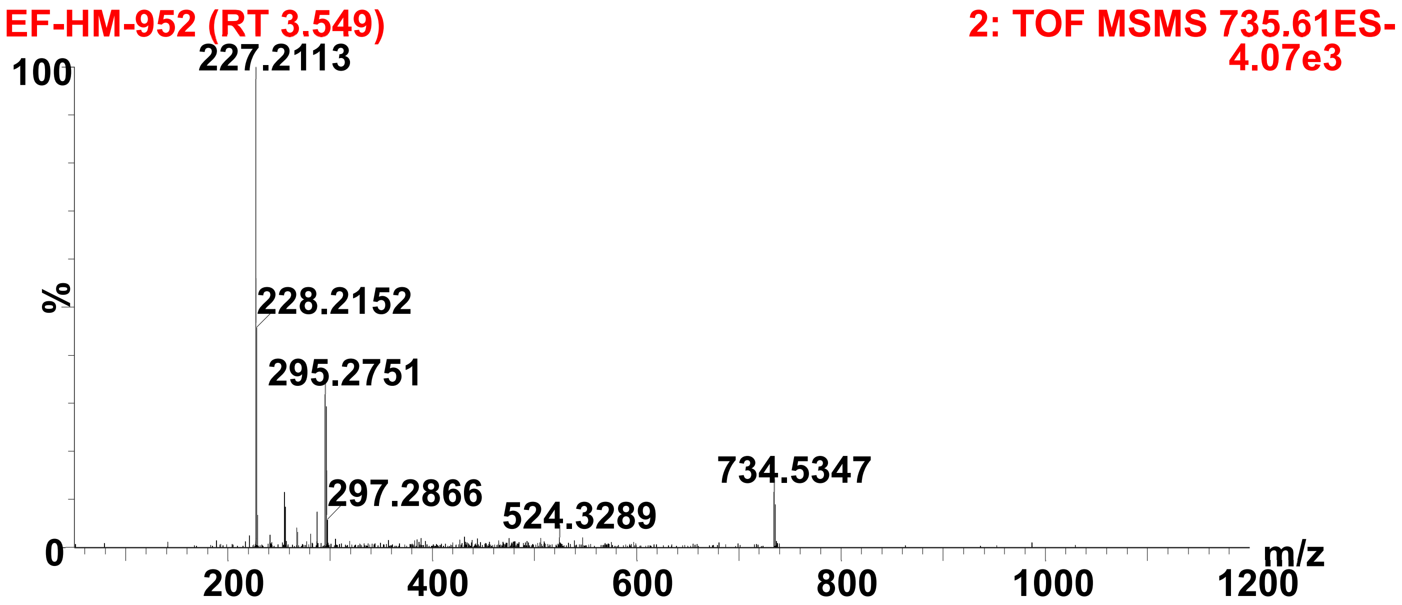

SI Figure 48. MSMS spectra of compound ID 3.549_735.61.

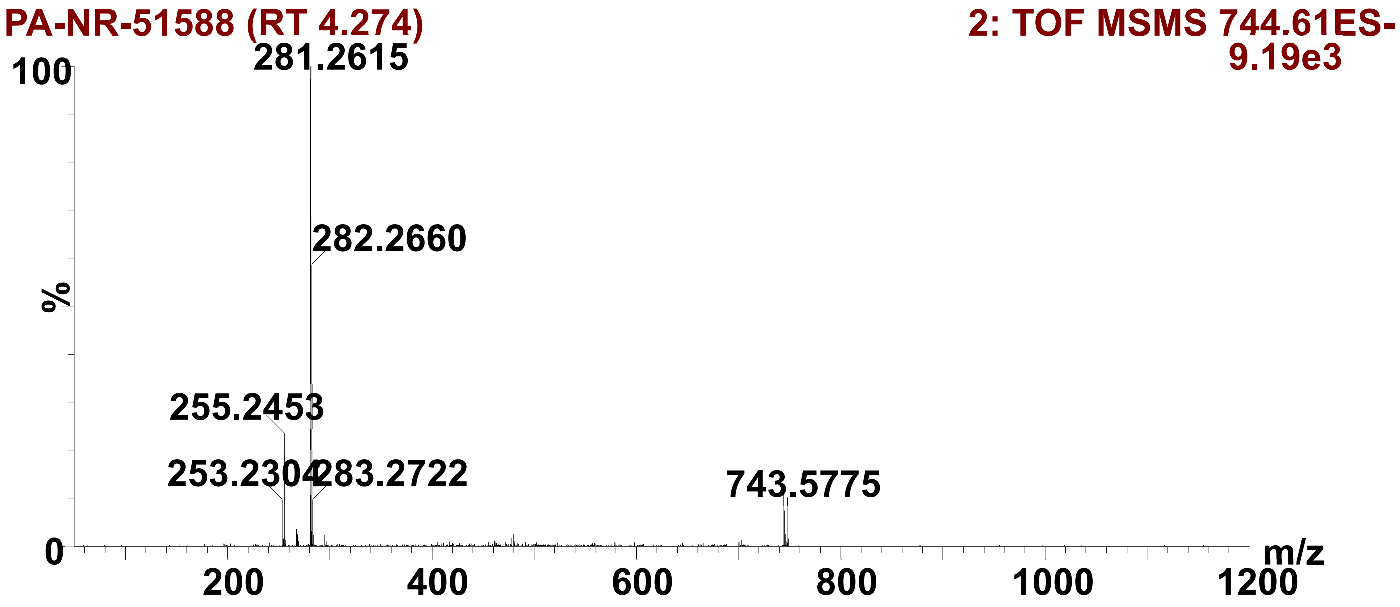

SI Figure 49. MSMS spectra of compound ID 4.274_744.61.

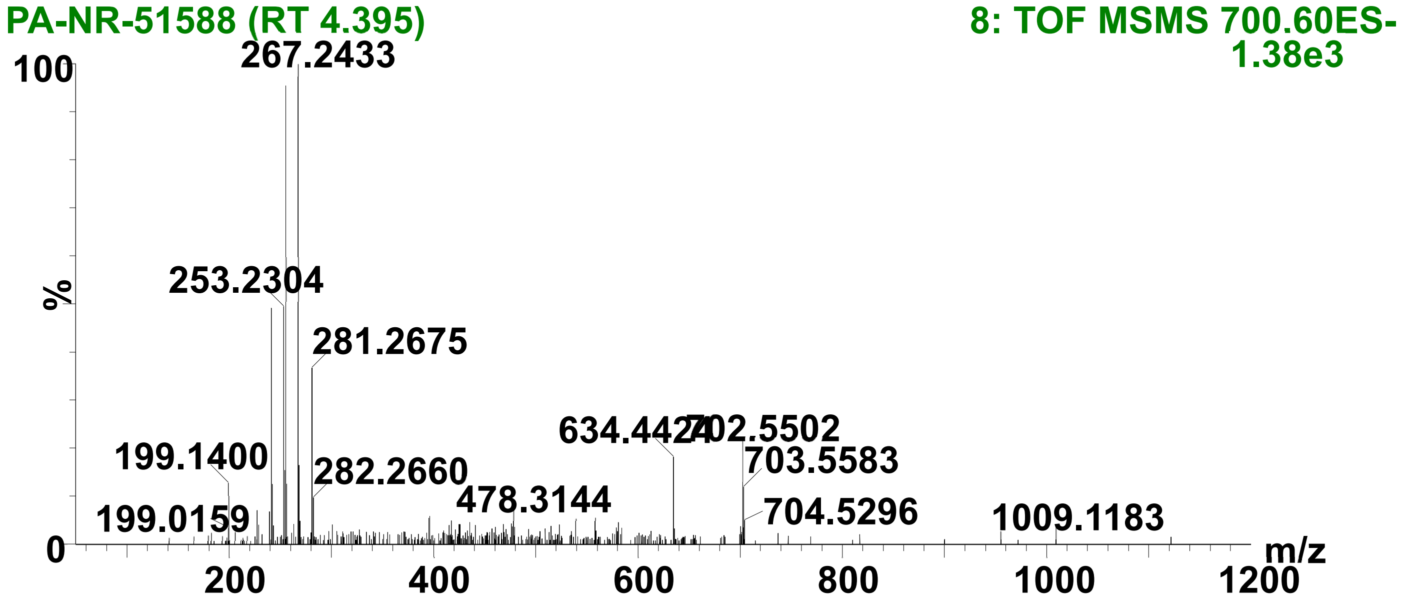

SI Figure 50. MSMS spectra of compound ID 4.395_700.60.

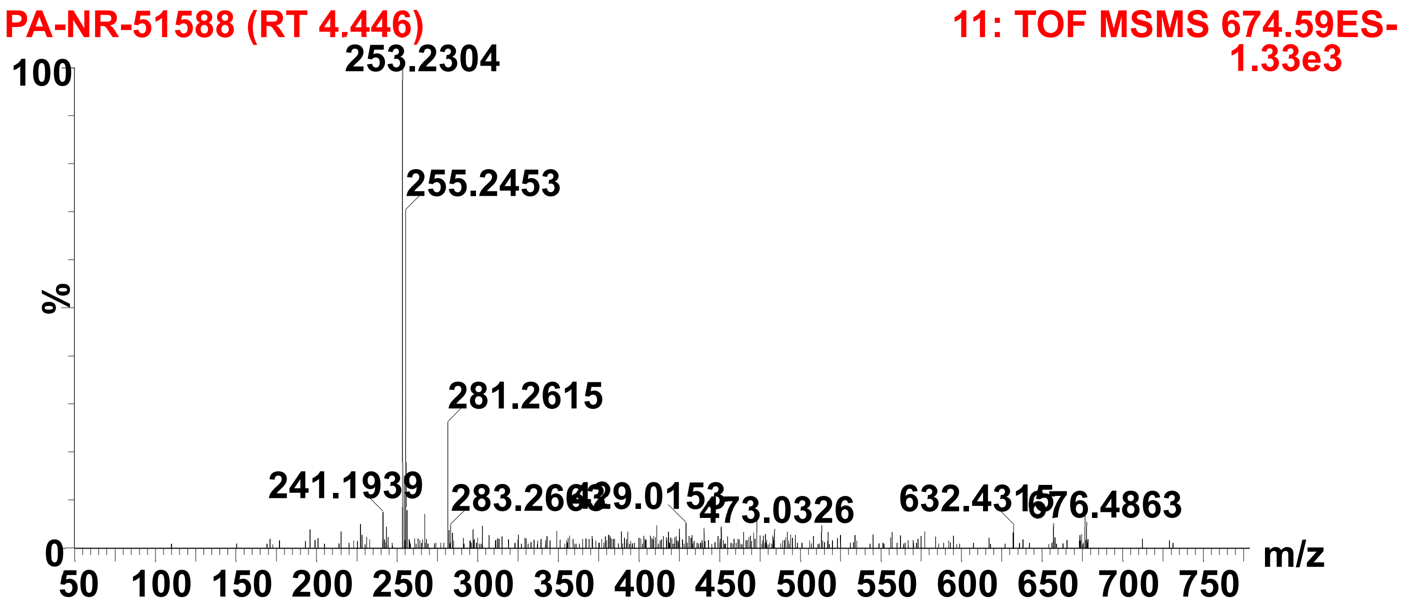

SI Figure 51. MSMS spectra of compound ID 4.446_674.59.

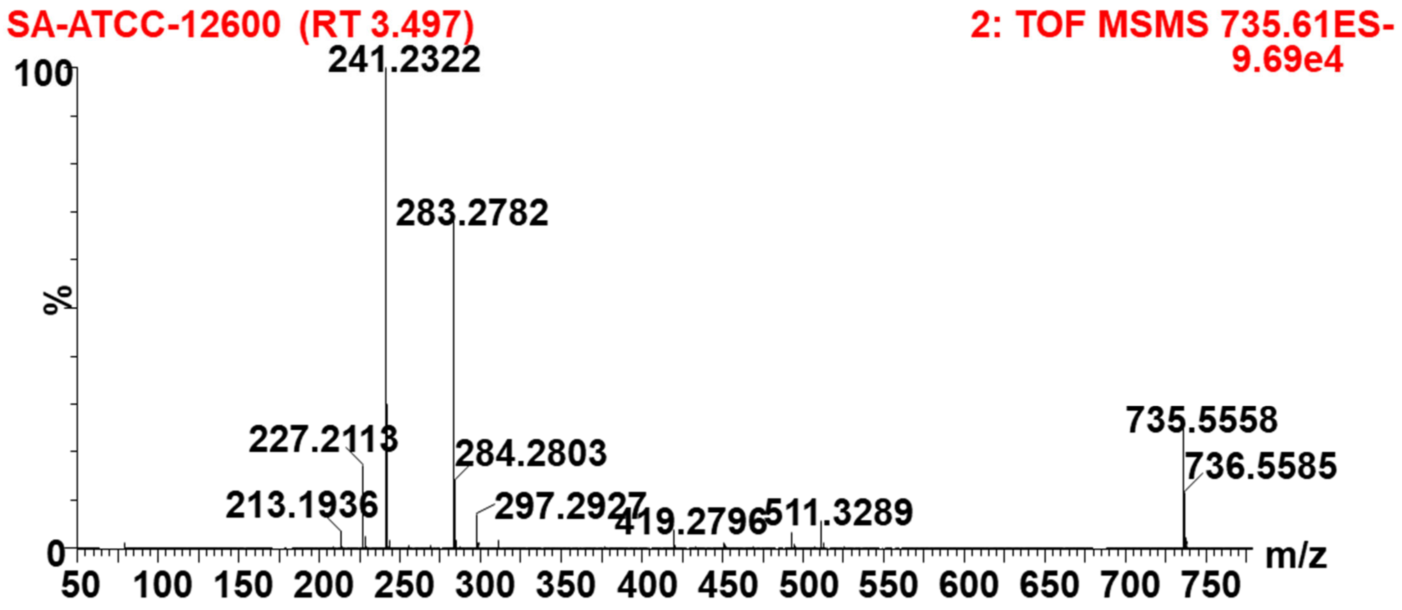

SI Figure 52. MSMS spectra of compound ID 3.497_735.61.

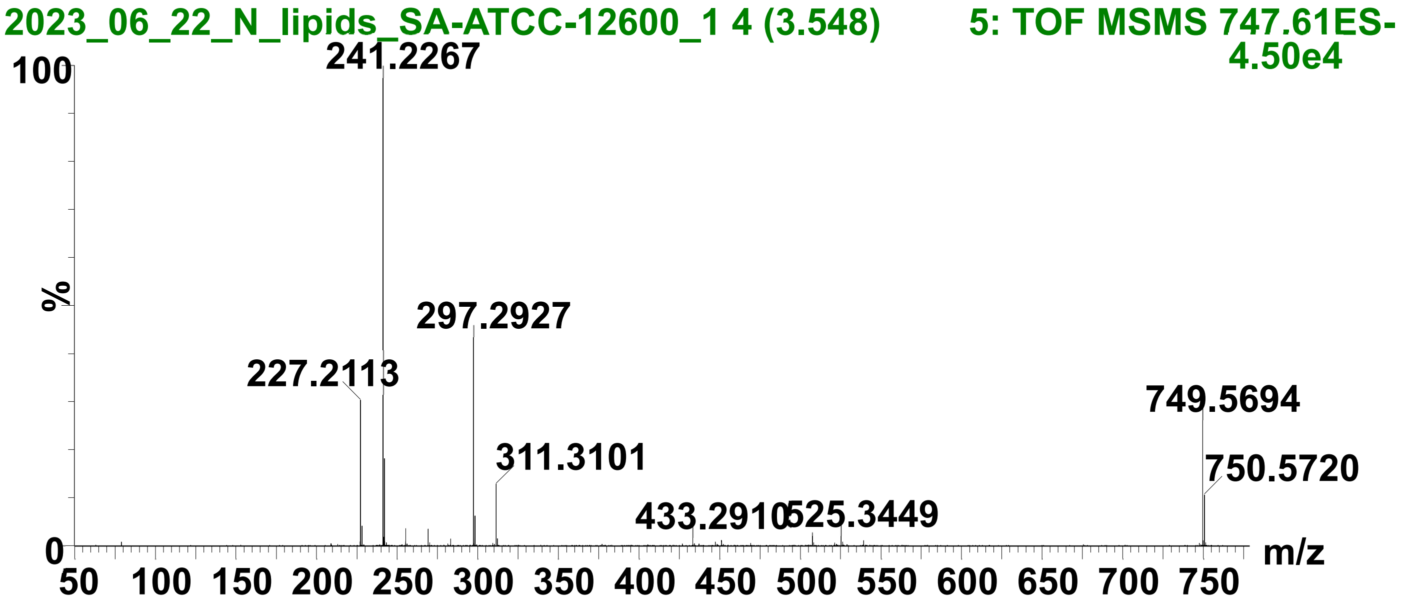

SI Figure 53. MSMS spectra of compound ID 3.548_747.61.

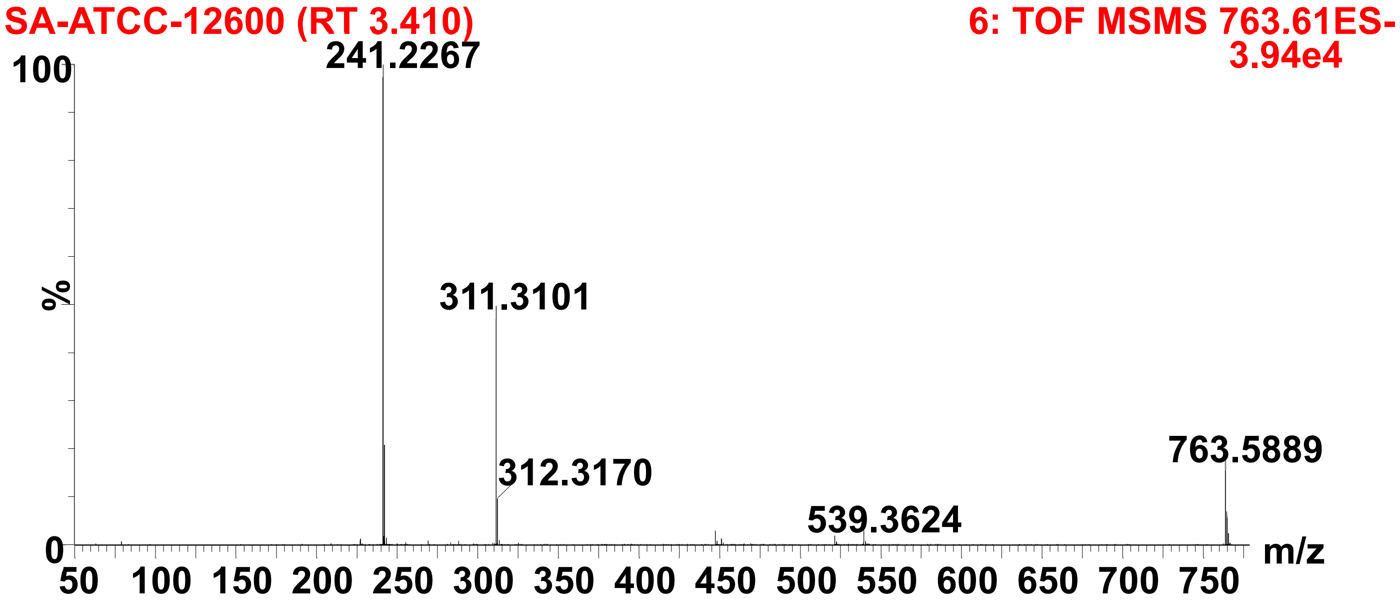

SI Figure 54. MSMS spectra of compound ID 3.410_763.61.

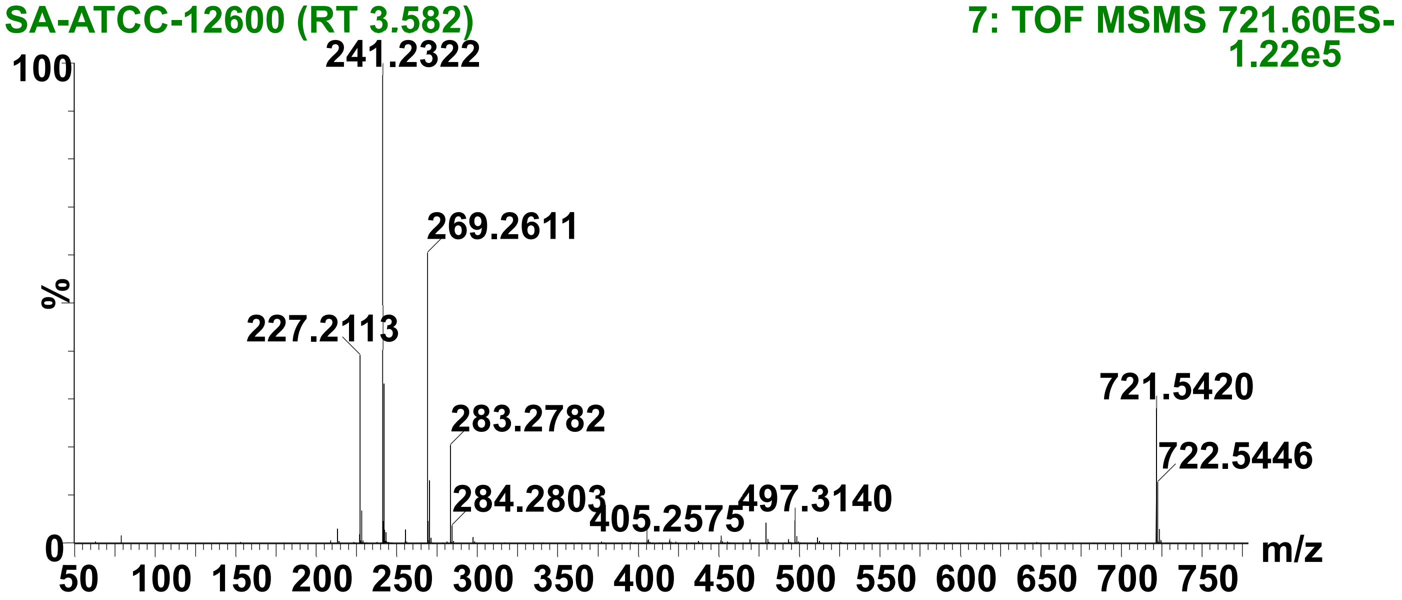

SI Figure 55. MSMS spectra of compound ID 3.582_721.60.

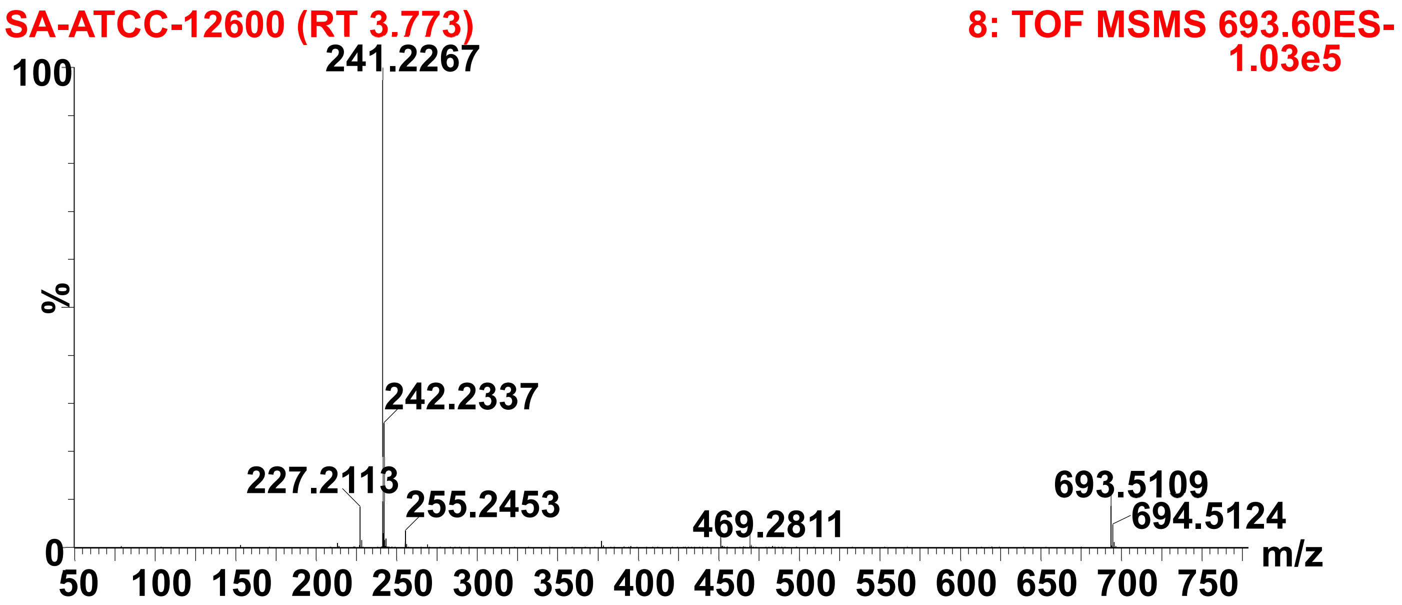

SI Figure 56. MSMS spectra of compound ID 3.773_693.60.

GNPS MSMS Spectra
Negative Mode

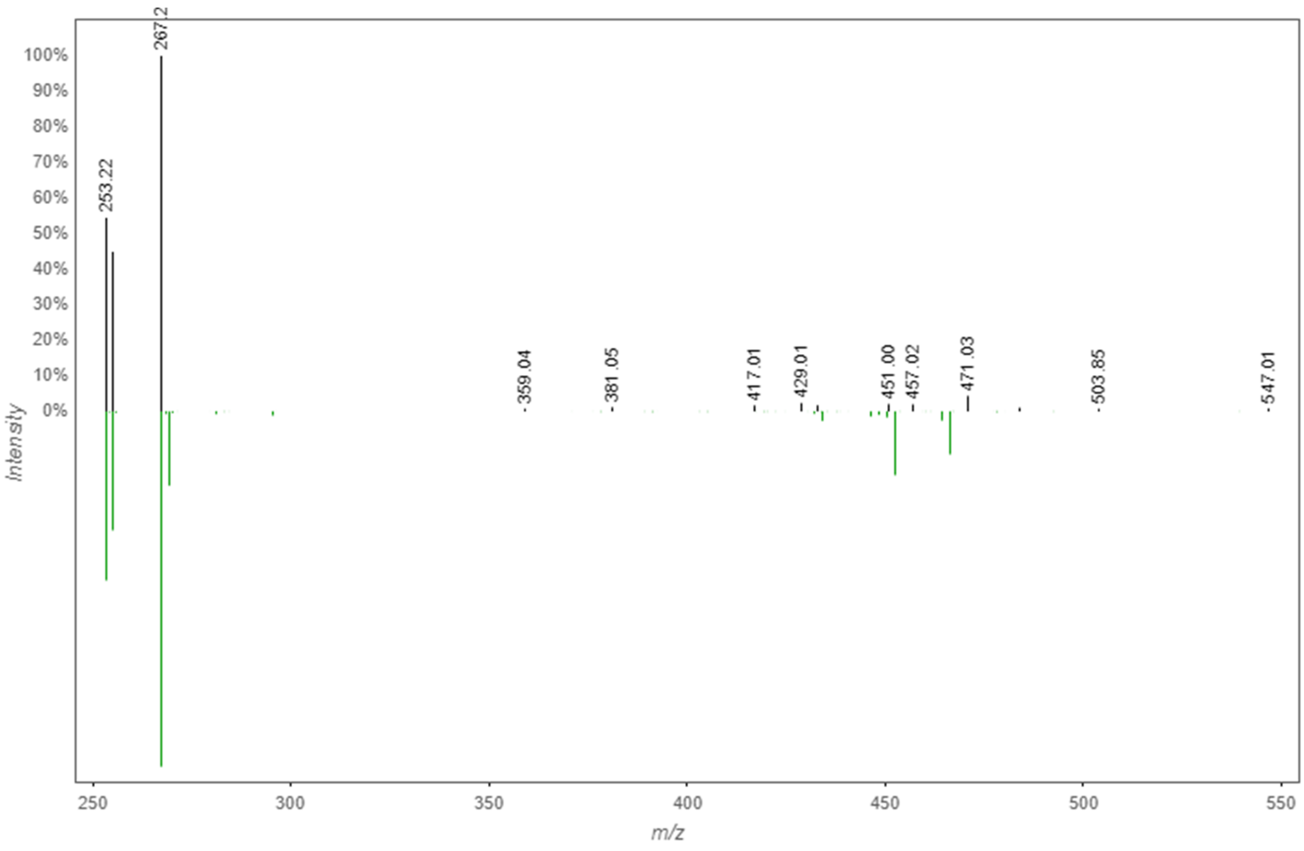

SI Figure 57. GNPS MSMS spectra of **702.60 m/z PE (16:0/17:1) [M-H]-**.

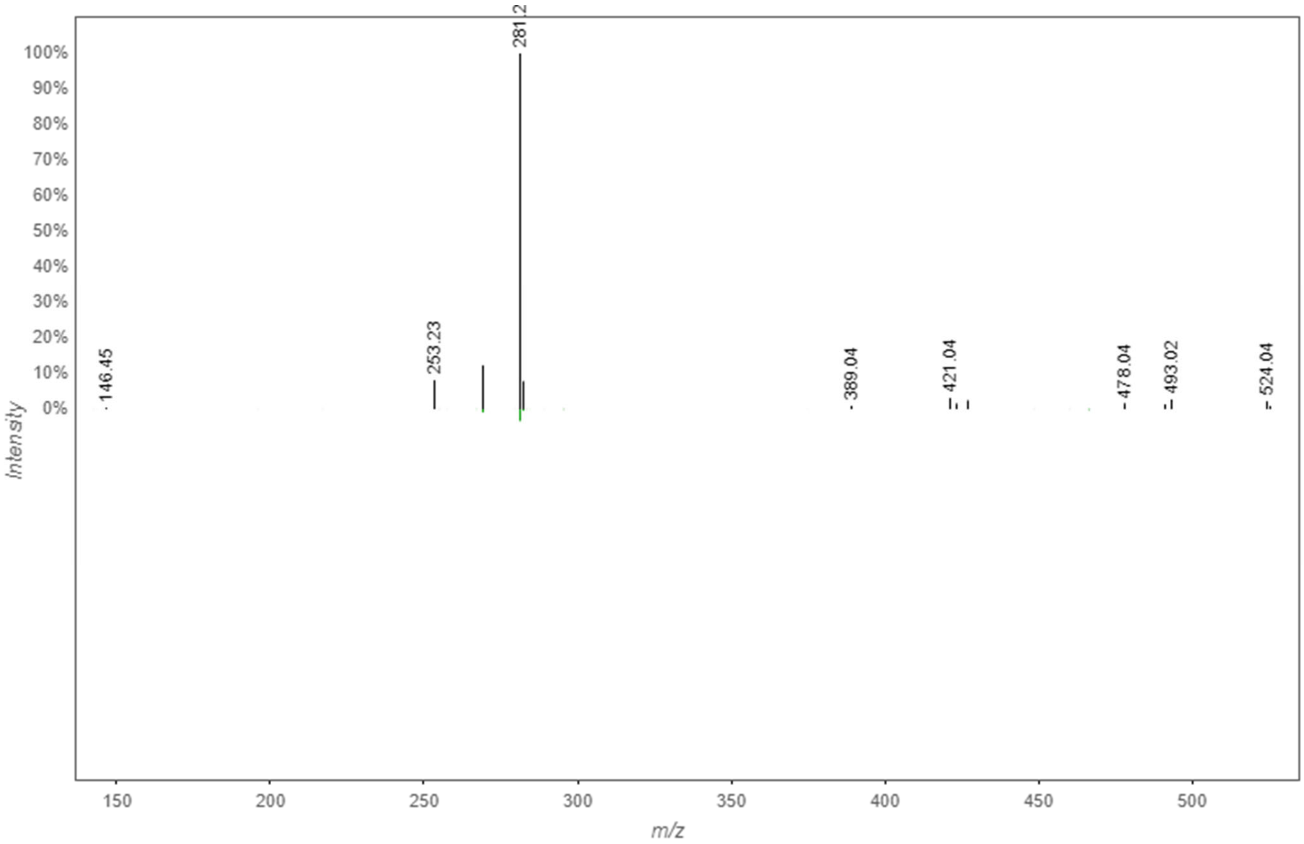

SI Figure 58. GNPS MSMS spectra of **700.60 m/z PE (16:0/17:1) [M-H]-**.

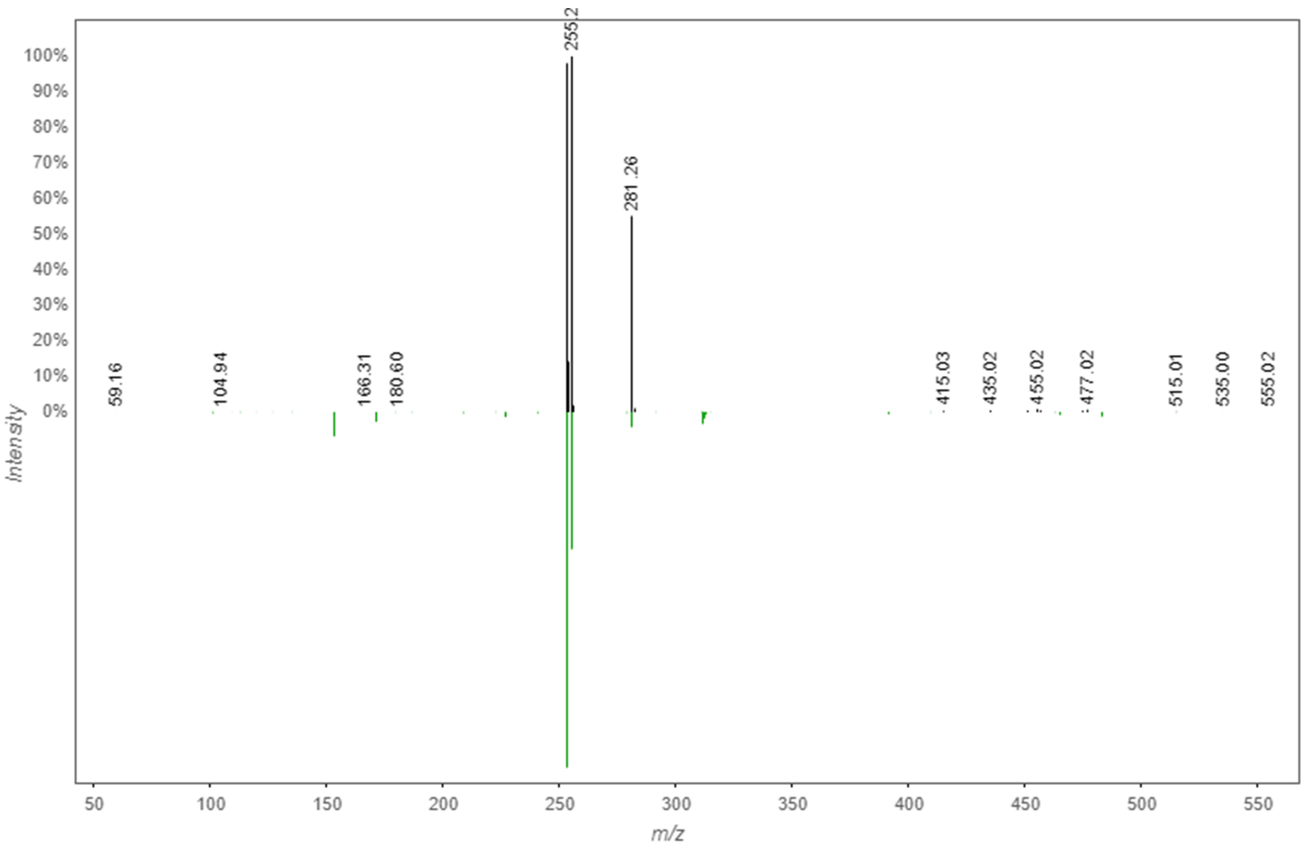

SI Figure 59. GNPS MSMS spectra of **730.530 m/z PE (17:0/18:1) [M-H]-**.

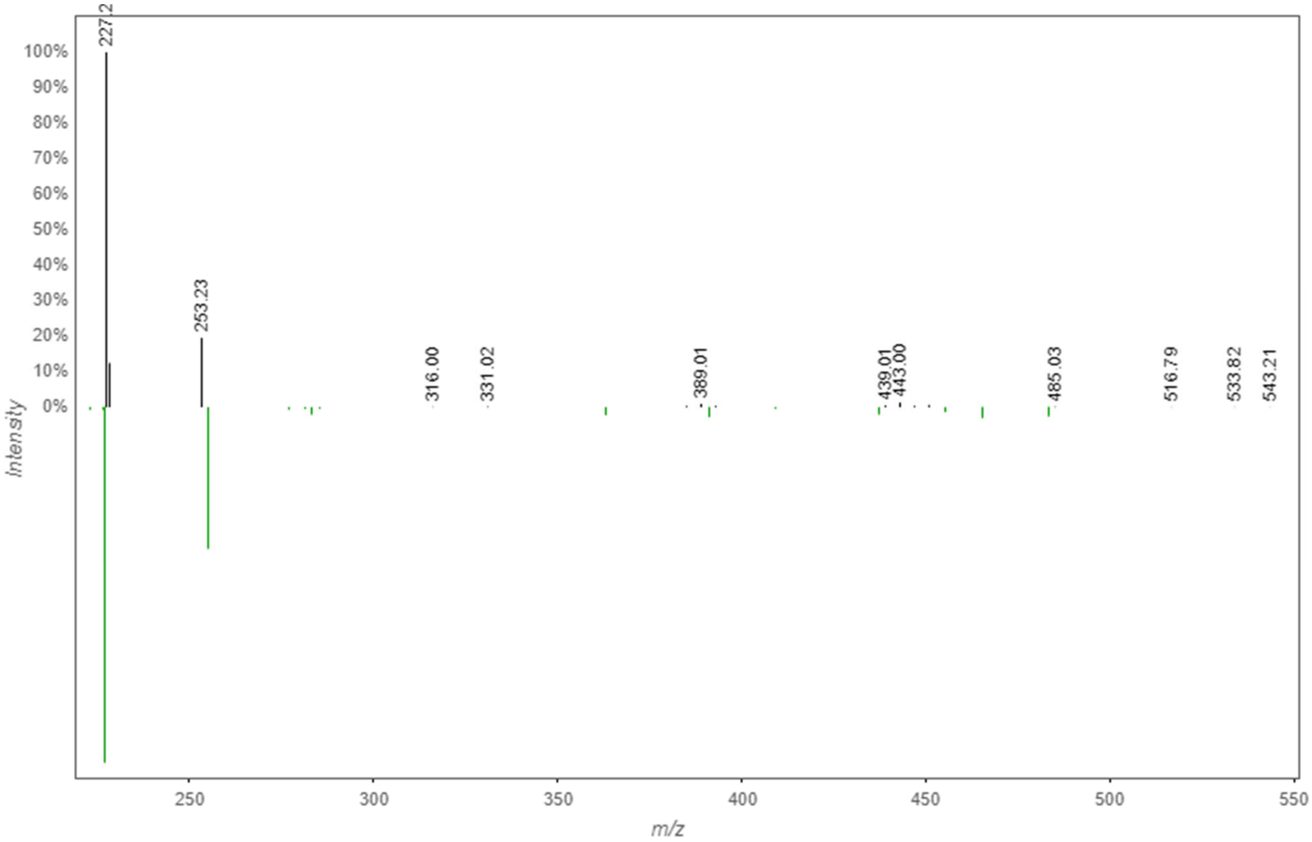

SI Figure 60. GNPS MSMS spectra of **719.69 m/z PG (16:0/16:1) [M-H]-**.

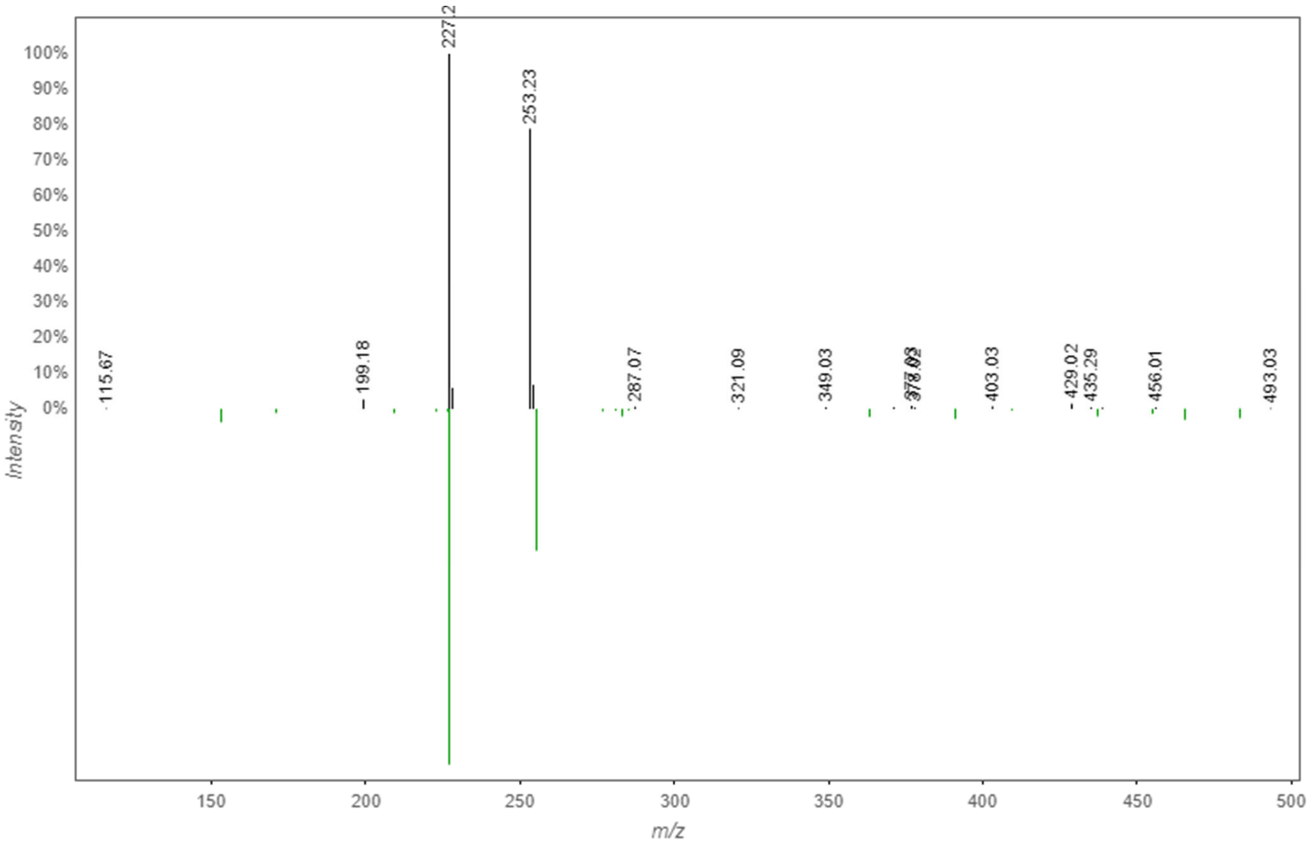

SI Figure 61. GNPS MSMS spectra of **691.50 m/z PG 30:0 [M-H]-**.

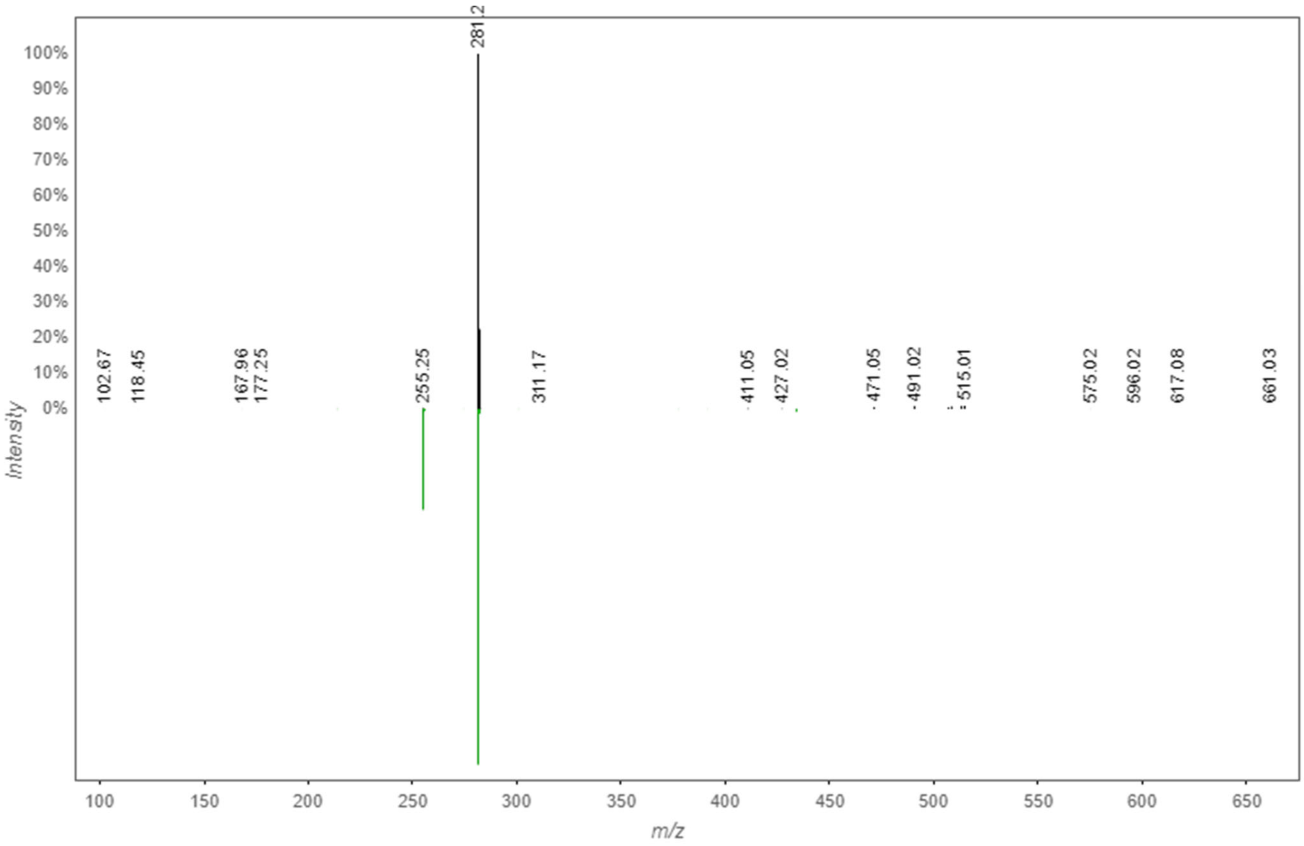

SI Figure 62. GNPS MSMS spectra of **716.60 m/z PE 16:0-18:1 [M-H]-**.

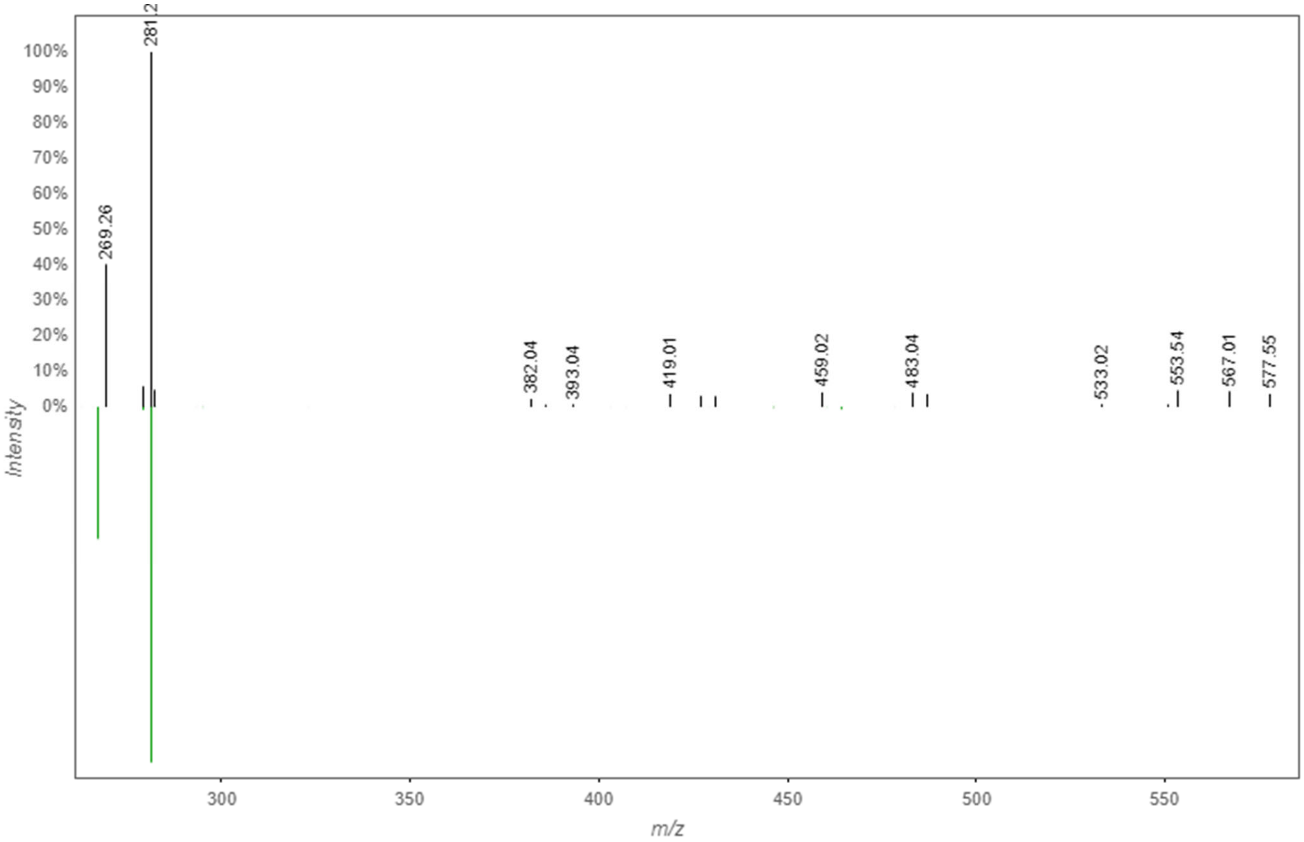

SI Figure 63. GNPS MSMS spectra of **730.31 m/z PE (17:1/18:1) [M-H]-.**

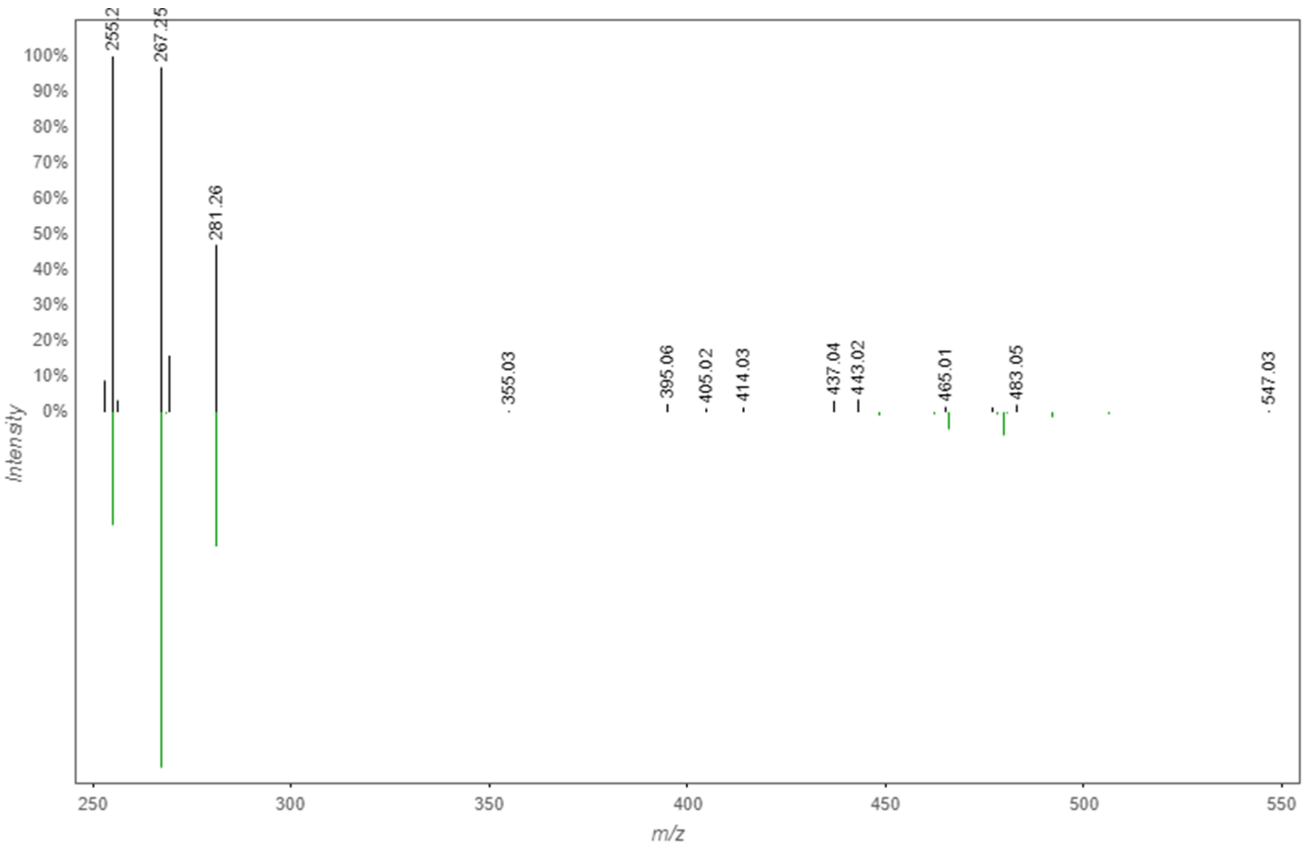

SI Figure 64. GNPS MSMS spectra of **728.96 M/Z PC 16:0-17:1 / 15:0-18:1 [M-H]-.**

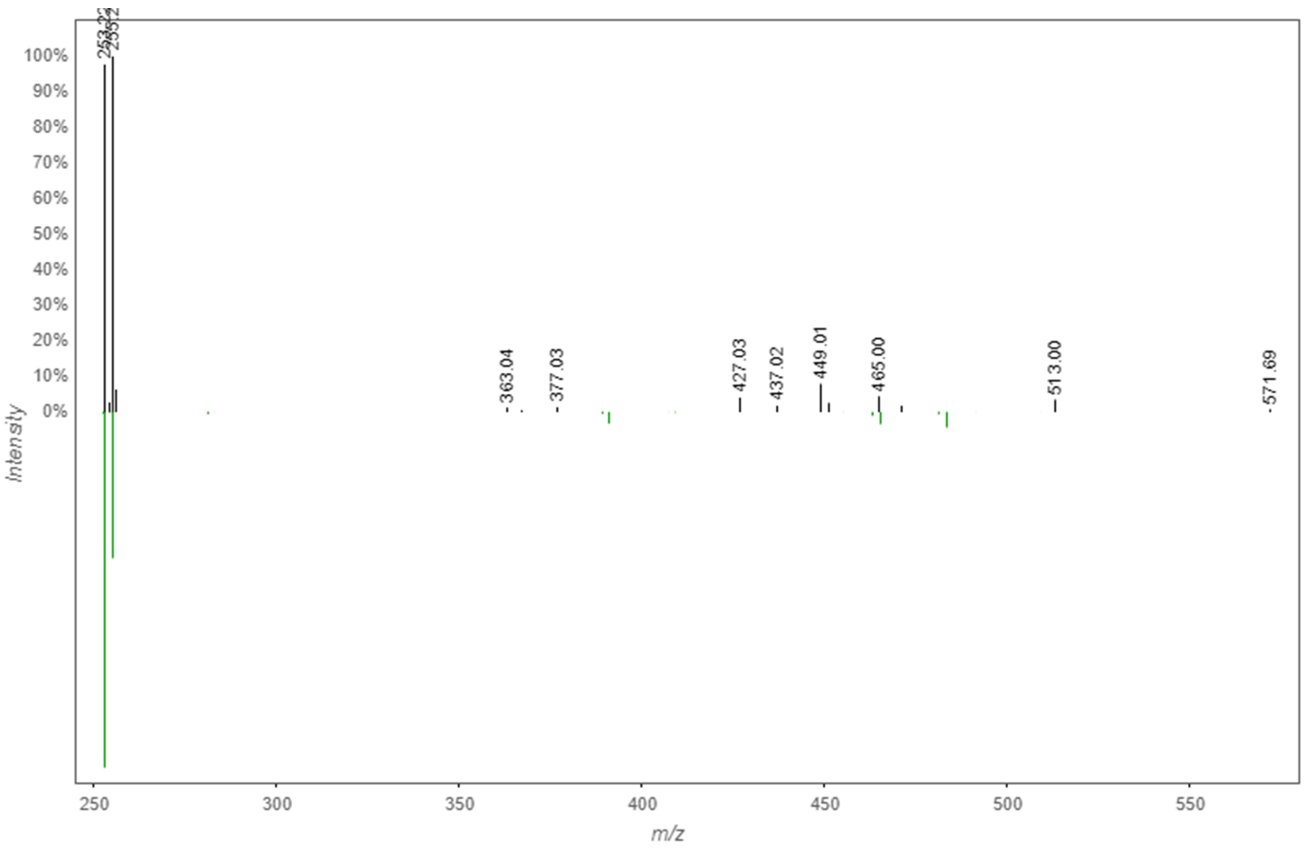

SI Figure 65. GNPS MSMS spectra of **719.6 m/z PG 32:1 16:0/16:1 [M-H]-.**

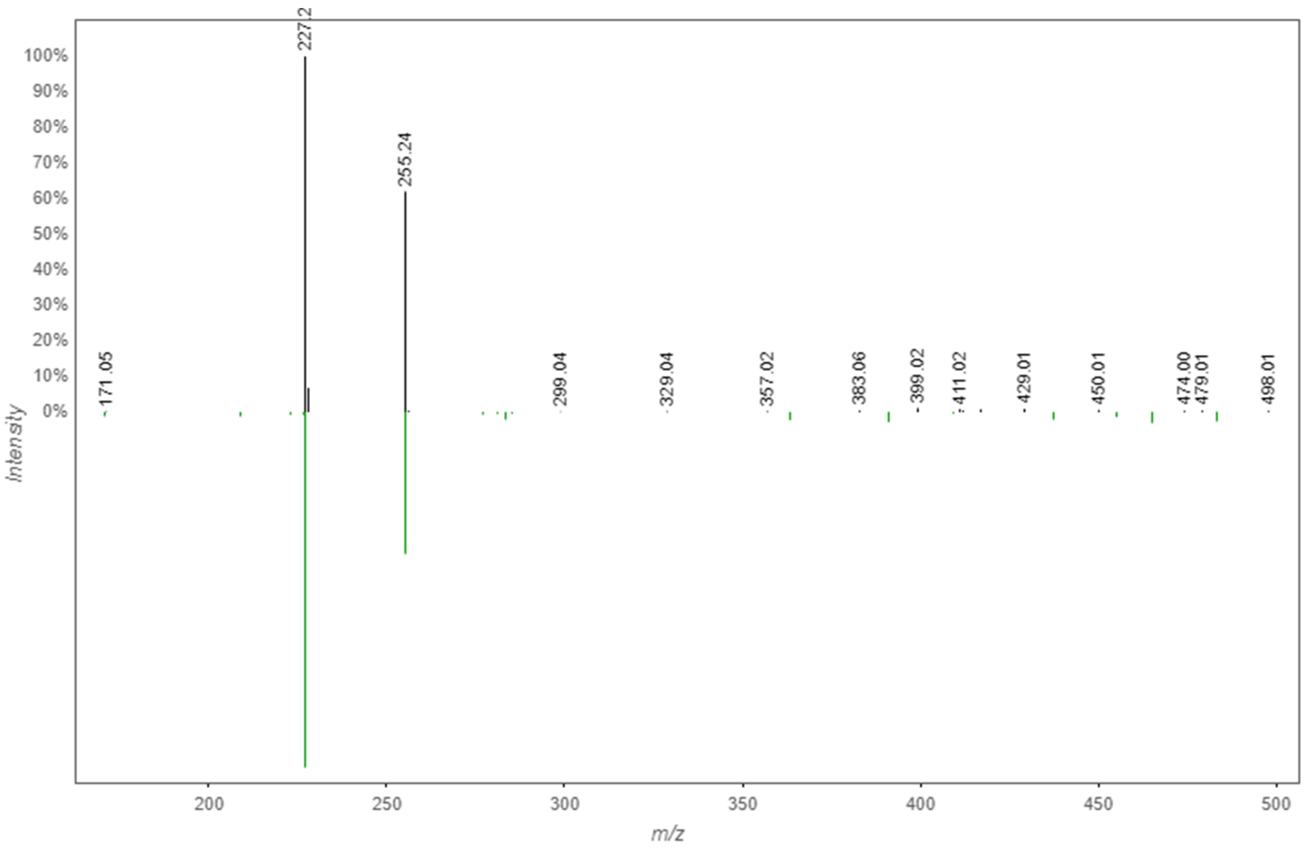

SI Figure 66. GNPS MSMS spectra of **693.60 m/z PG 30:0 14:0/16:0 [M-H]-.**

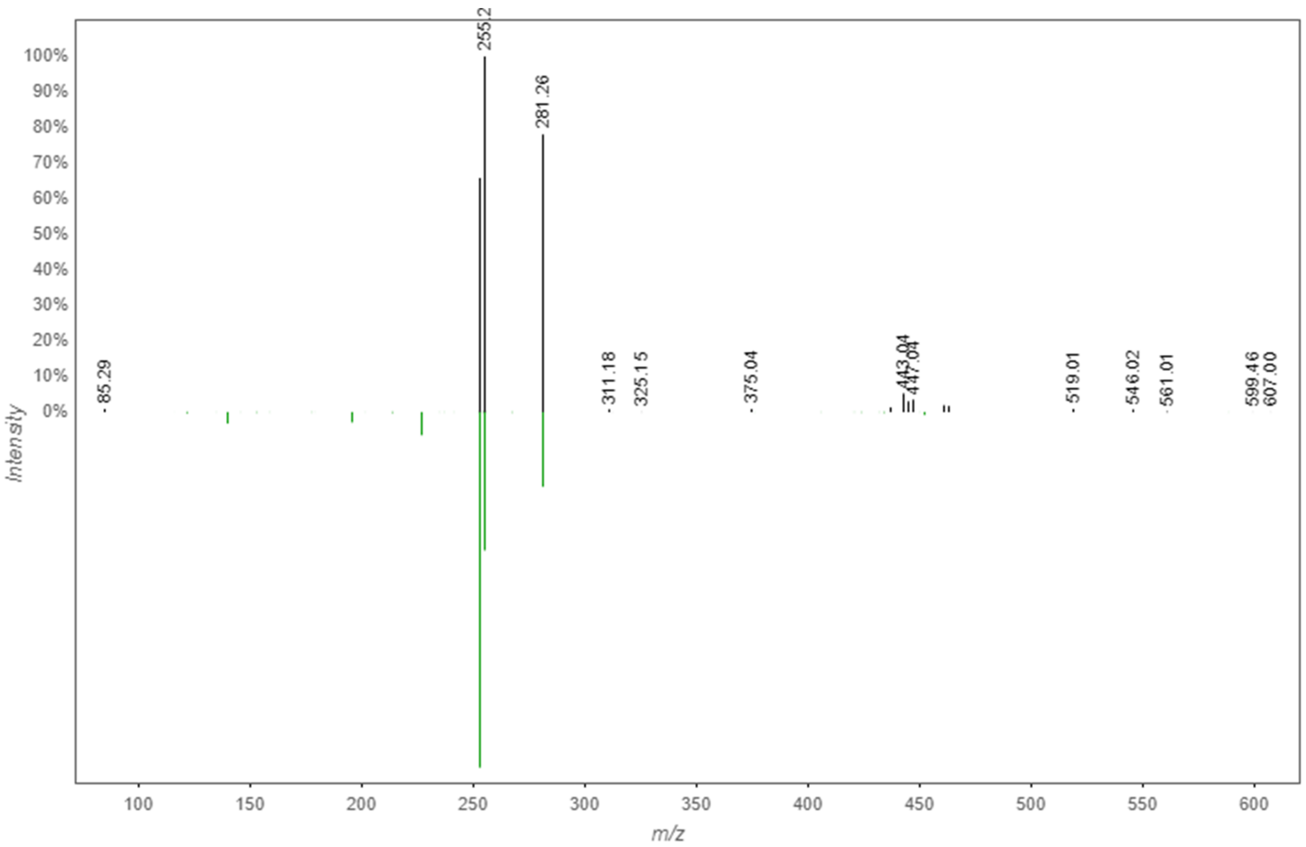

SI Figure 67. GNPS MSMS spectra of **686.60 m/z PE (16:0/16:1) [M-H]-.**

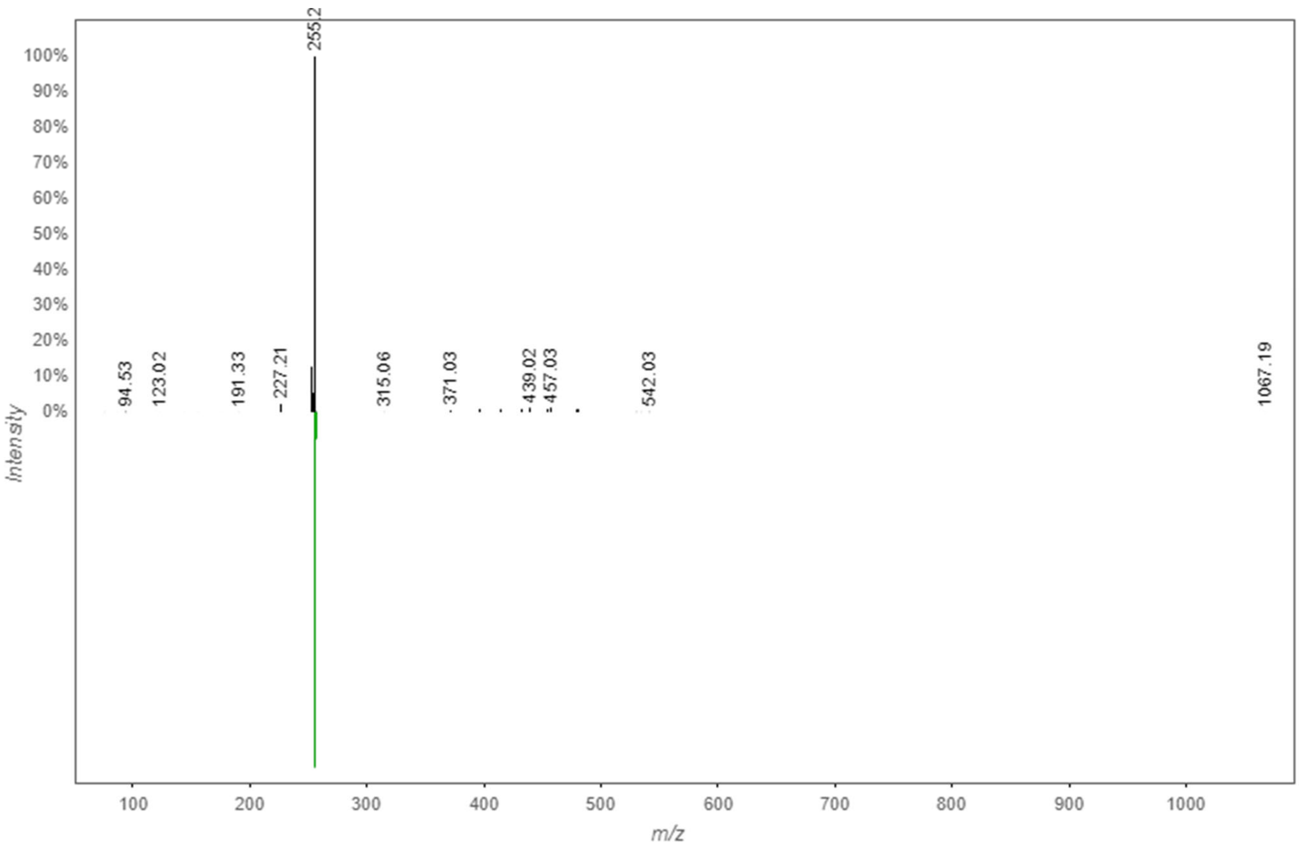

SI Figure 68. GNPS MSMS spectra of **721.5 m/z PG 32:0 16:0/16:0 [M-H]-.**

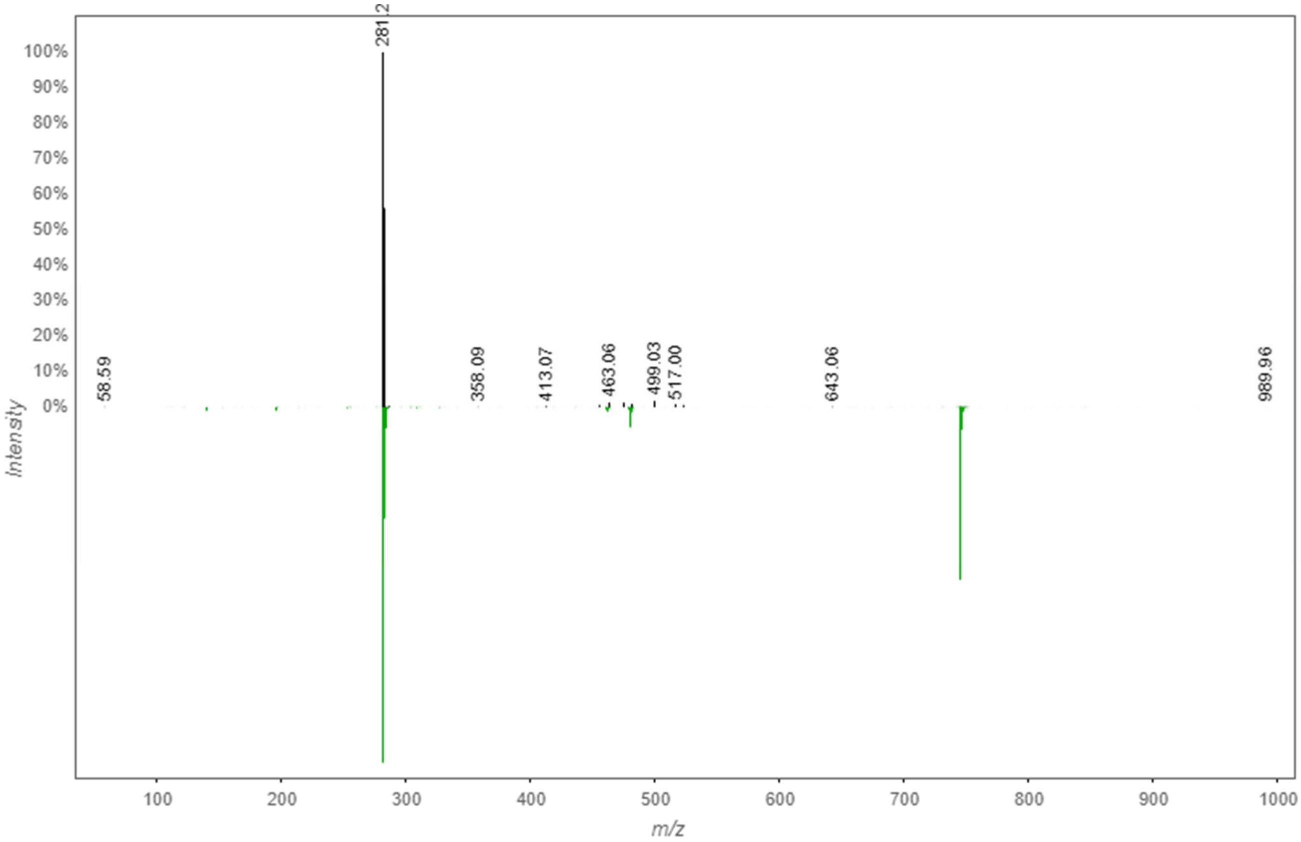

SI Figure 69. GNPS MSMS spectra of **744.61 M/Z PE 36:1 18:0/18:1 [M-H]-.**

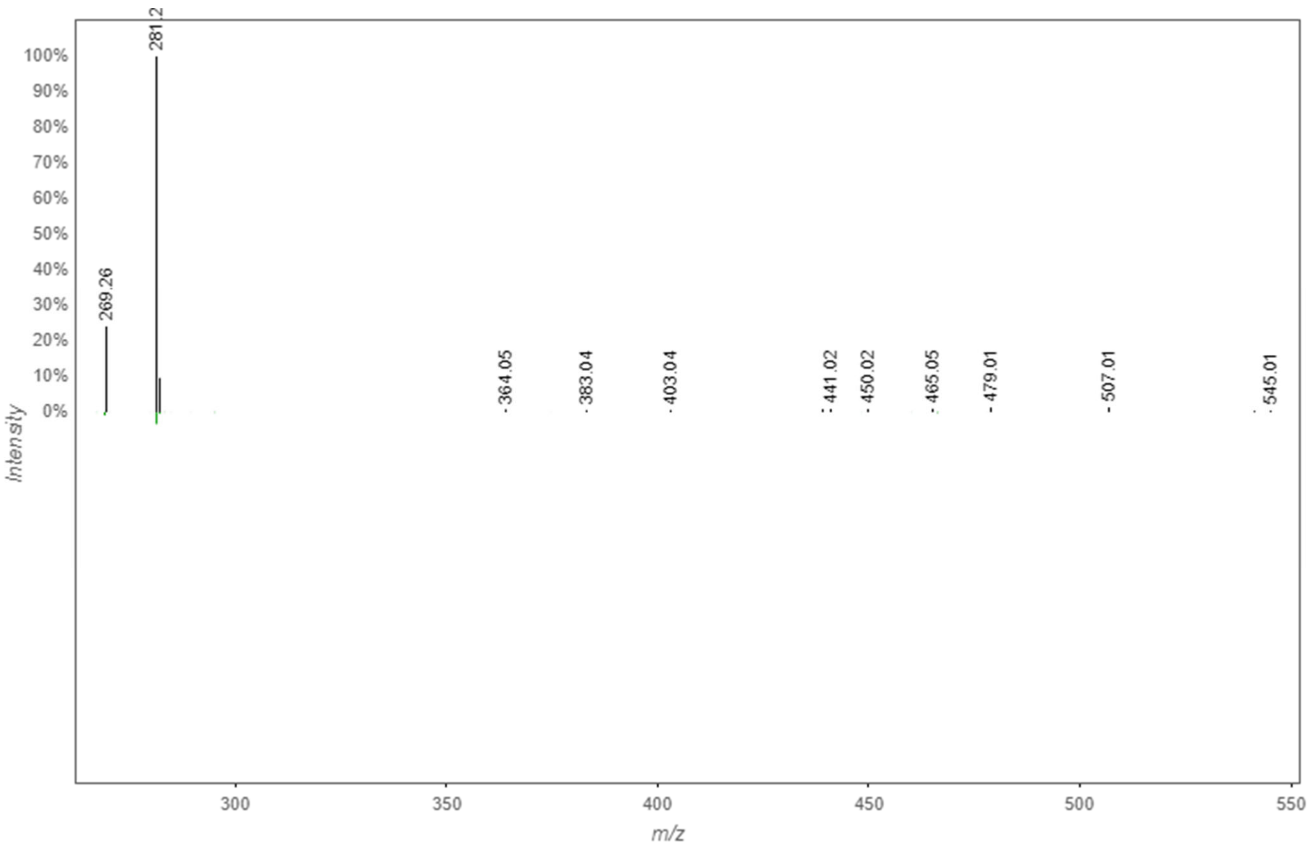

SI Figure 70. GNPS MSMS spectra of **729.52 M/Z PE(17:0/18:1); [M-H]-.**

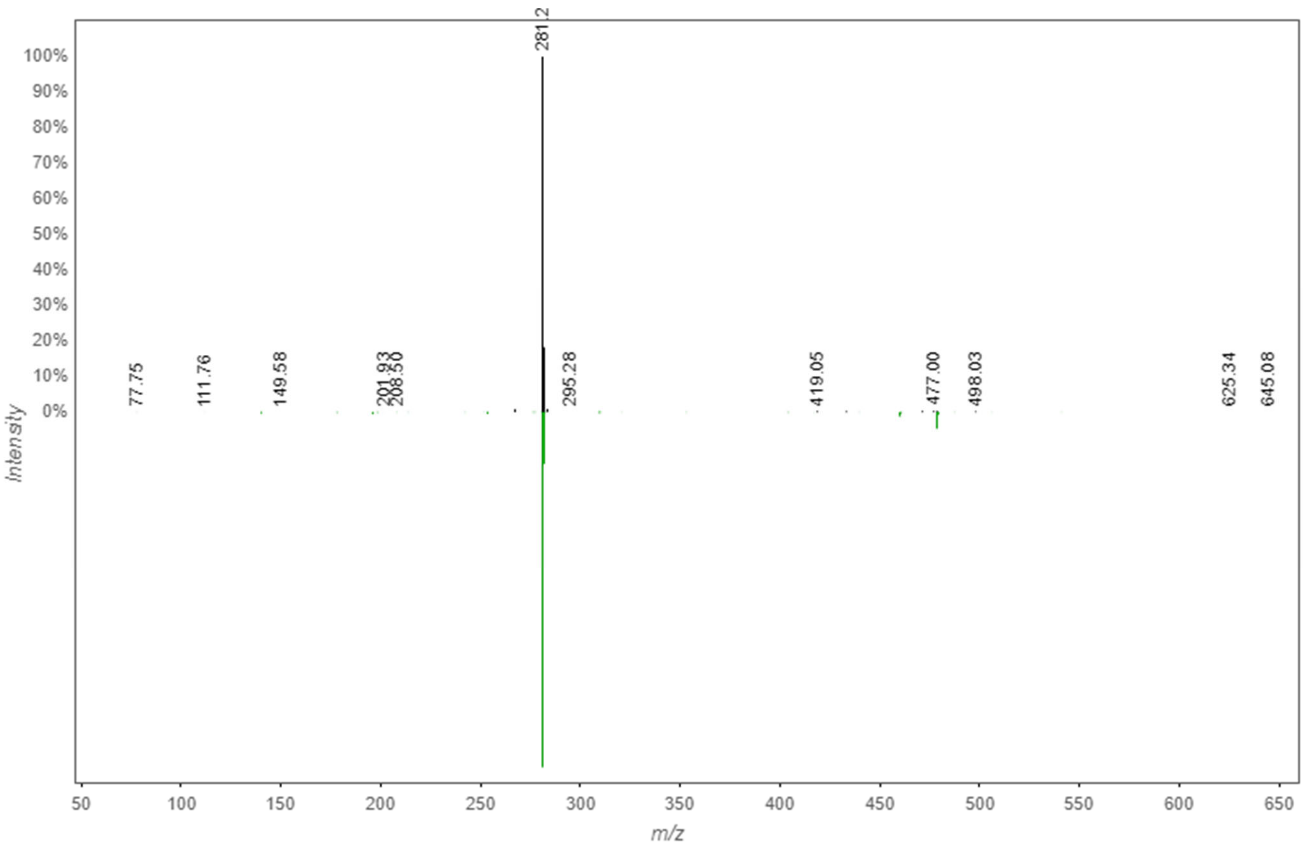

SI Figure 71. GNPS MSMS spectra of **742.61 m/z PE (18:1/18:1) [M-H]-.**

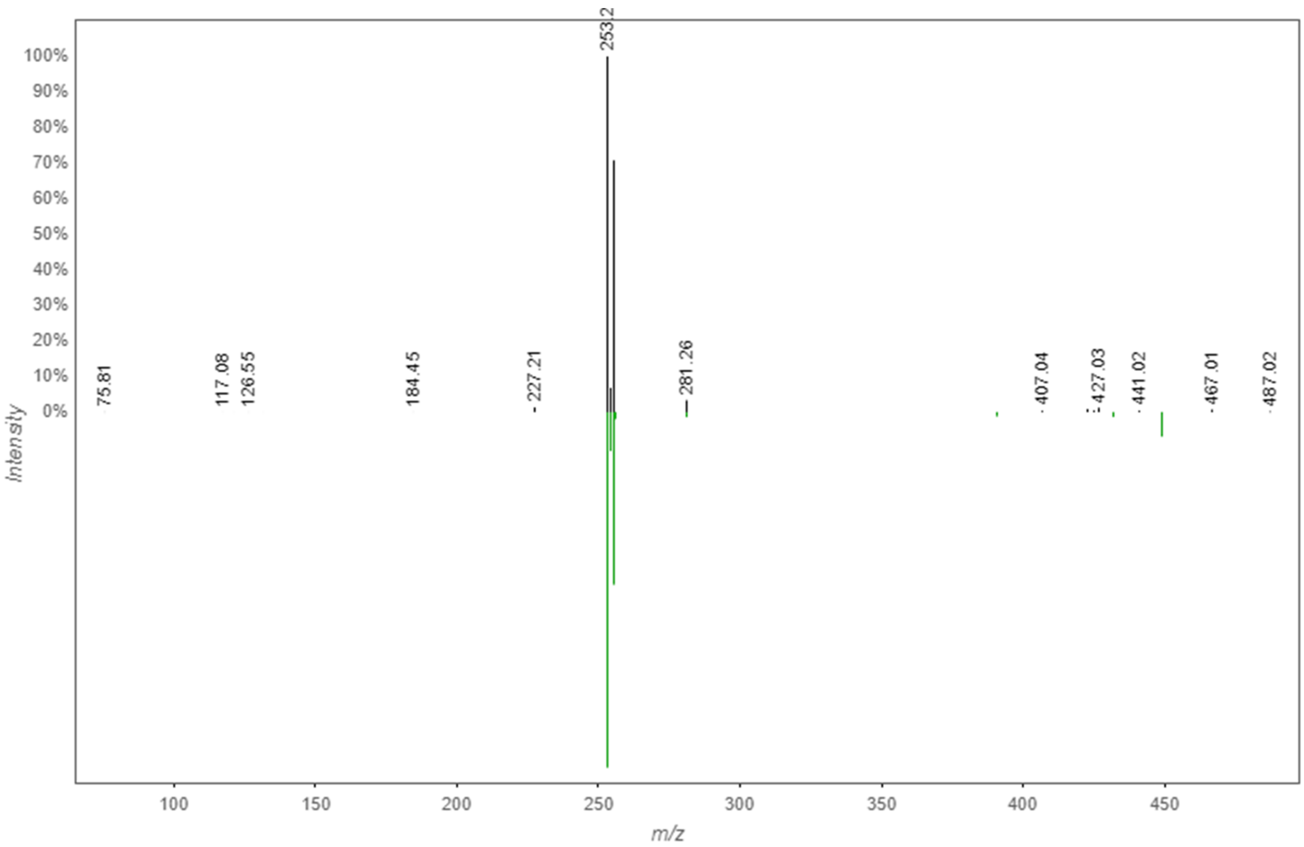

SI Figure 72. GNPS MSMS spectra of **688.6 m/z PE 16:0-16:1 [M-H]-.**

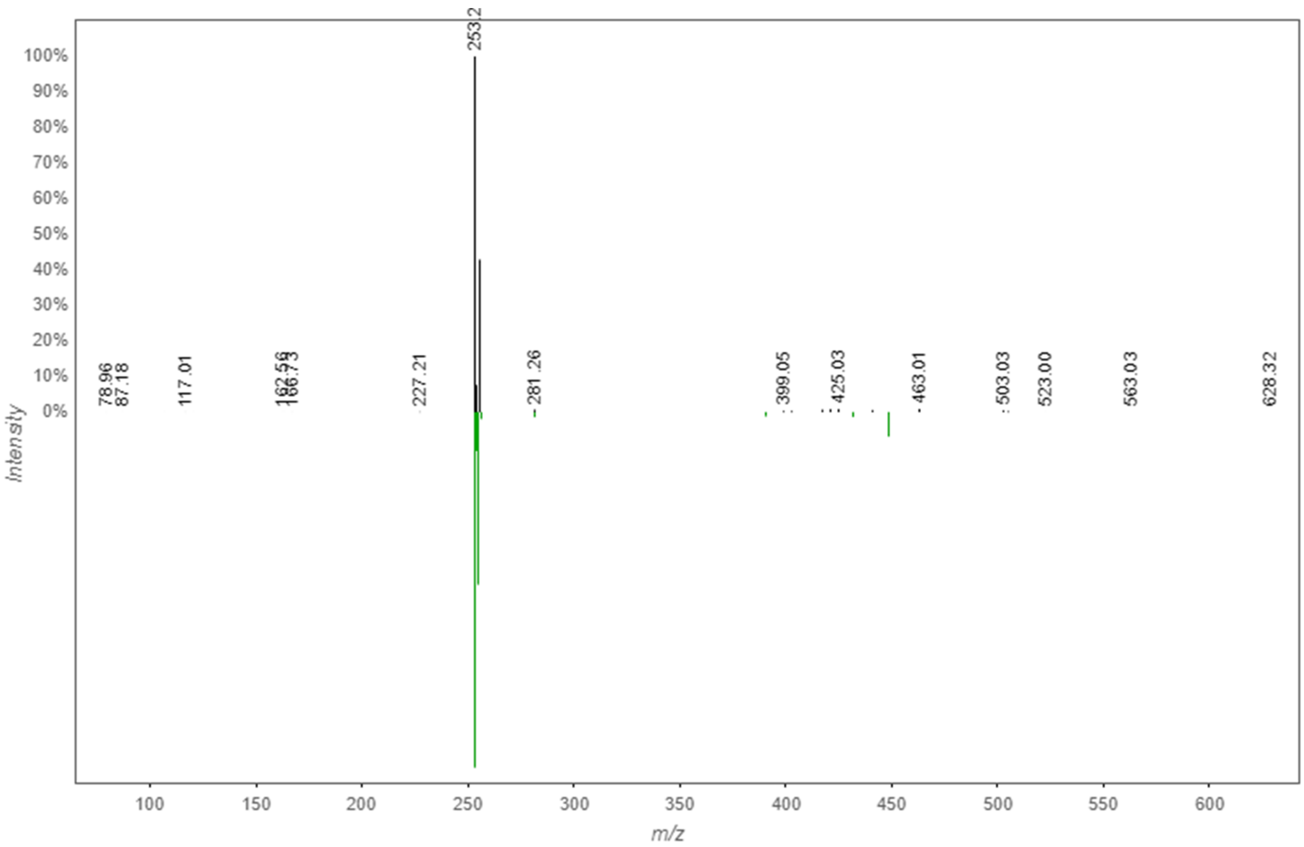

SI Figure 73. GNPS MSMS spectra of **686.6 m/z PE 16:0-16:1 [M-H]-.**

.

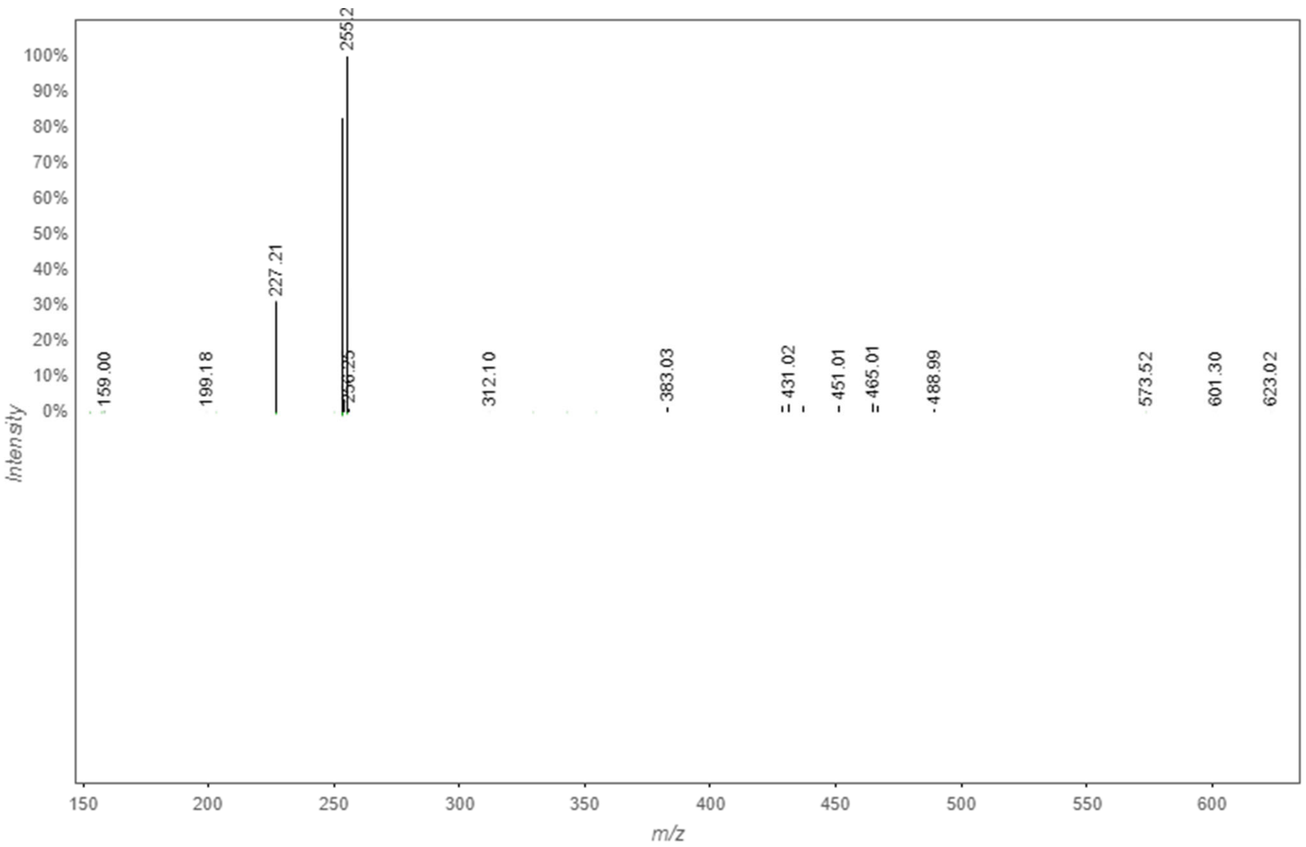

SI Figure 74. GNPS MSMS spectra of **691.5 M/Z PG(14:0/16:1) [M-H]-.**

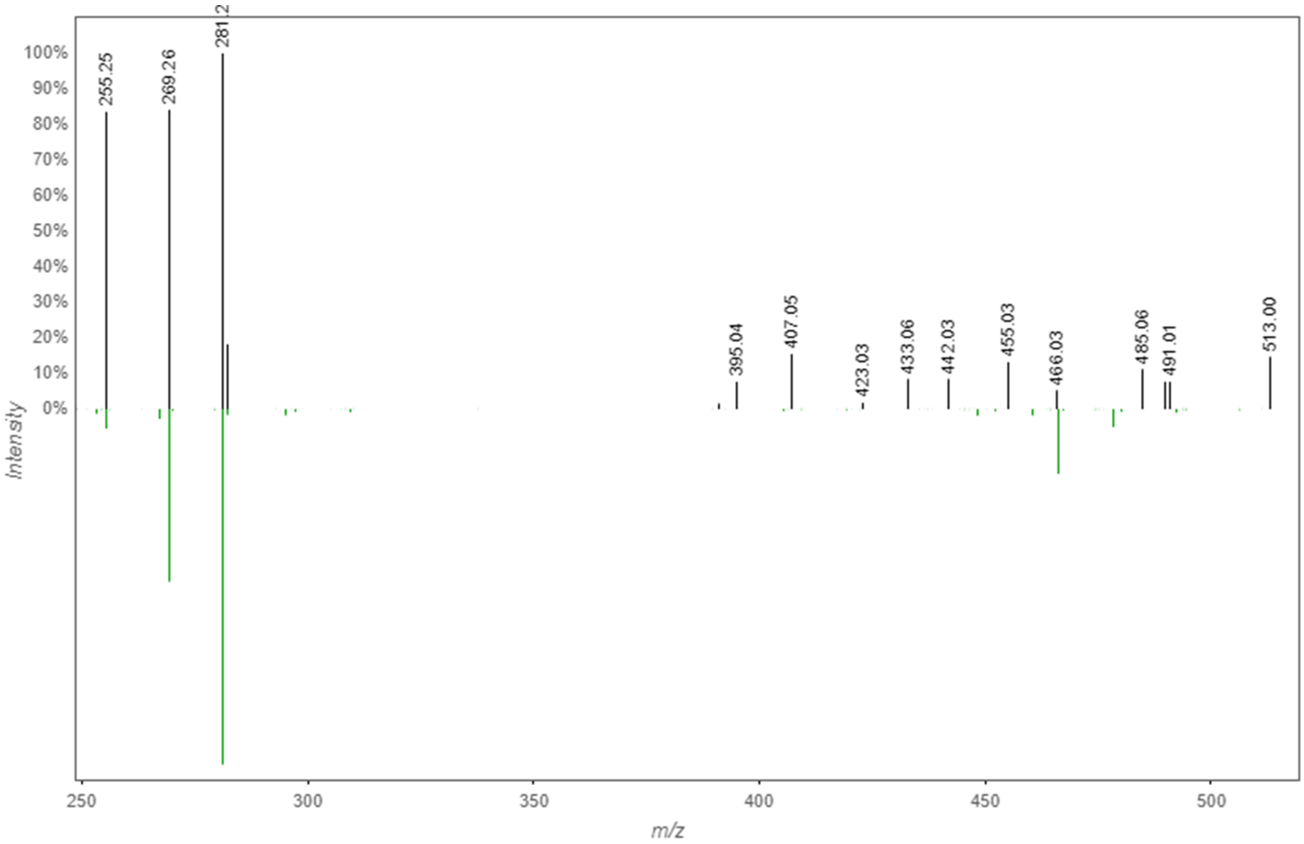

SI Figure 75. GNPS MSMS spectra of **730.61 M/Z PE (17:0/18:1) [M-H]-.**

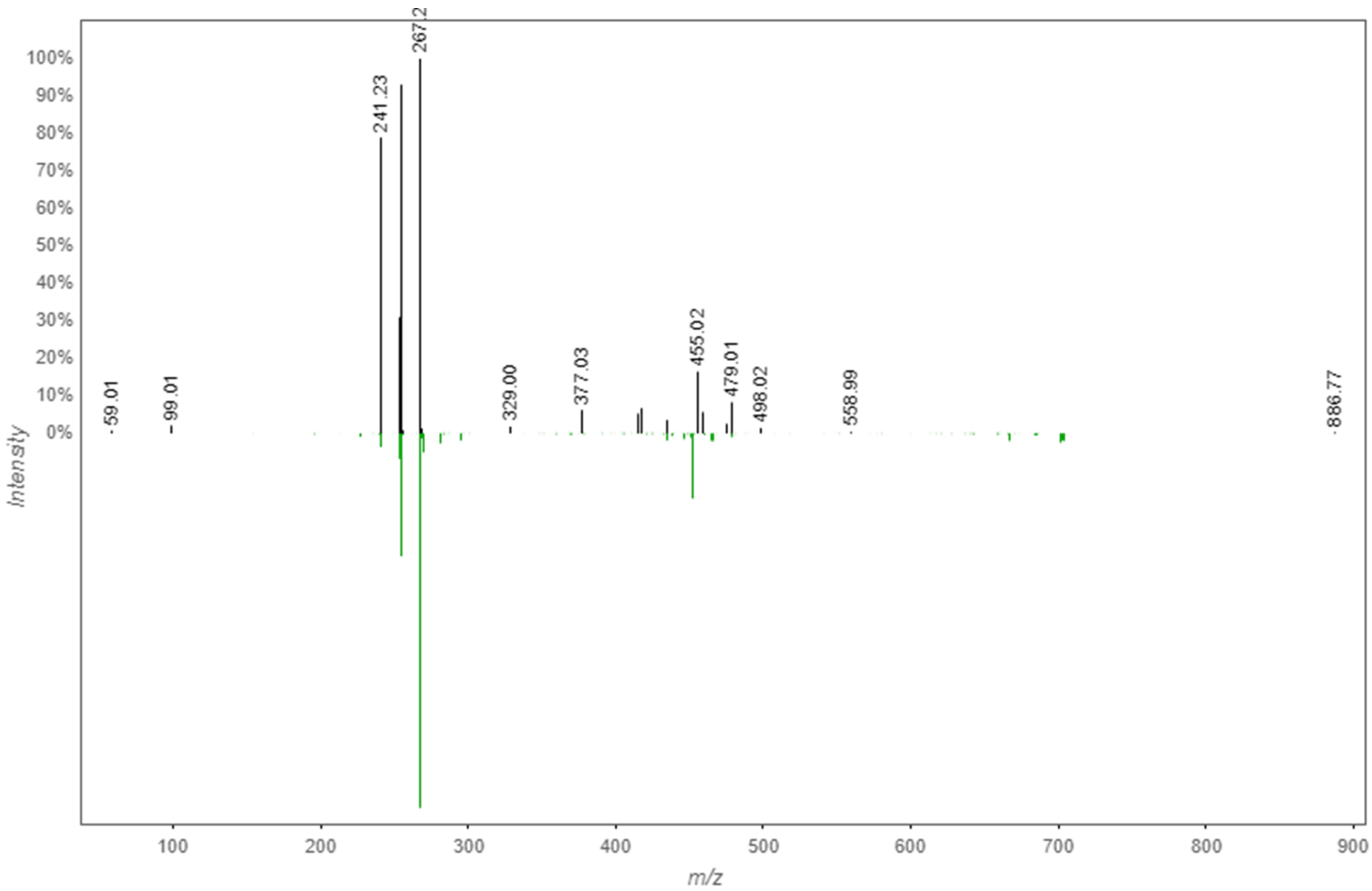

SI Figure 76. GNPS MSMS spectra of **702.60 M/Z PE (16:0/17:1) [M-H]-.**

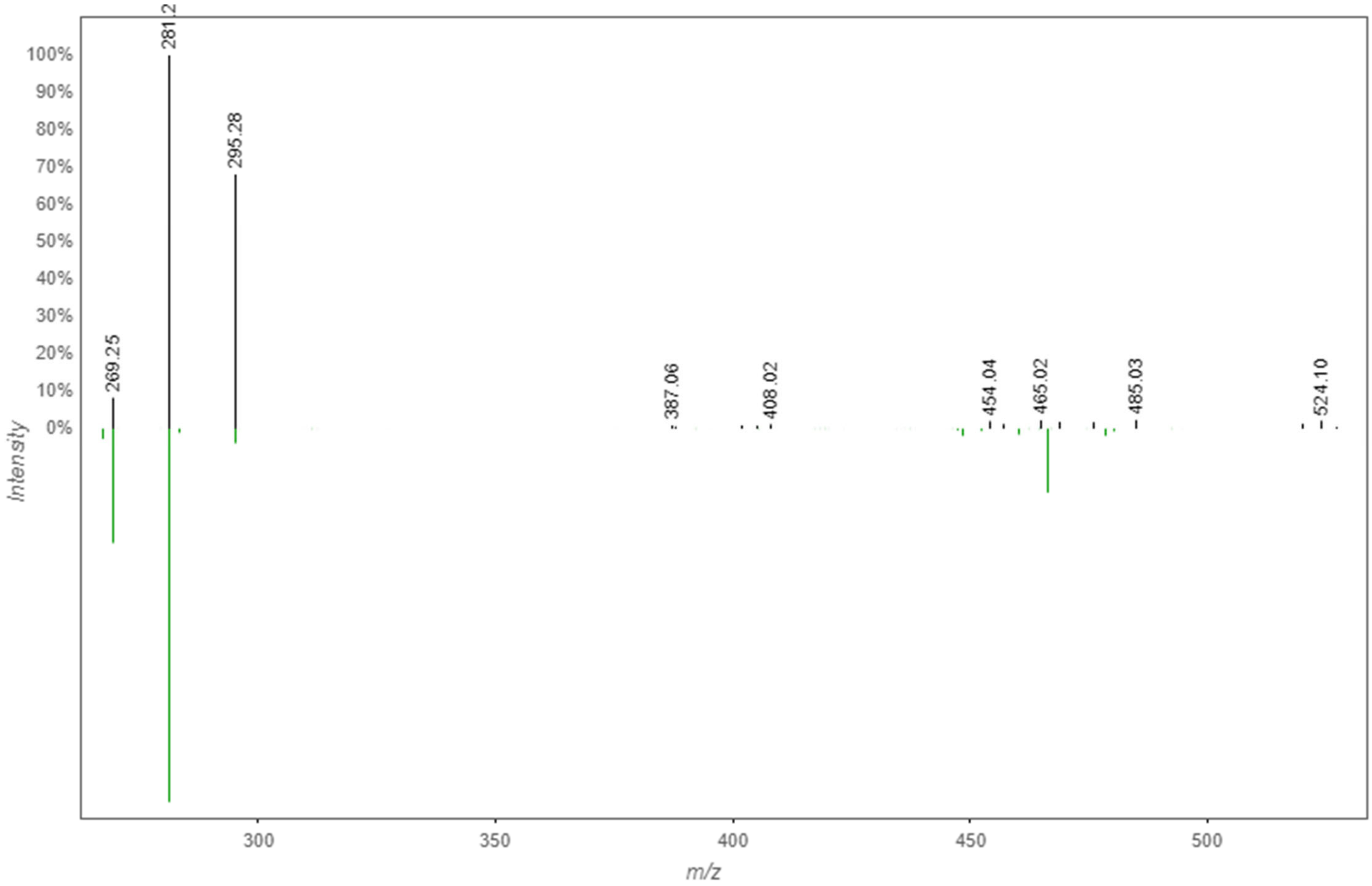

SI Figure 77. GNPS MSMS spectra of **730.61 M/Z PE (17:0/18:1) [M-H]-.**

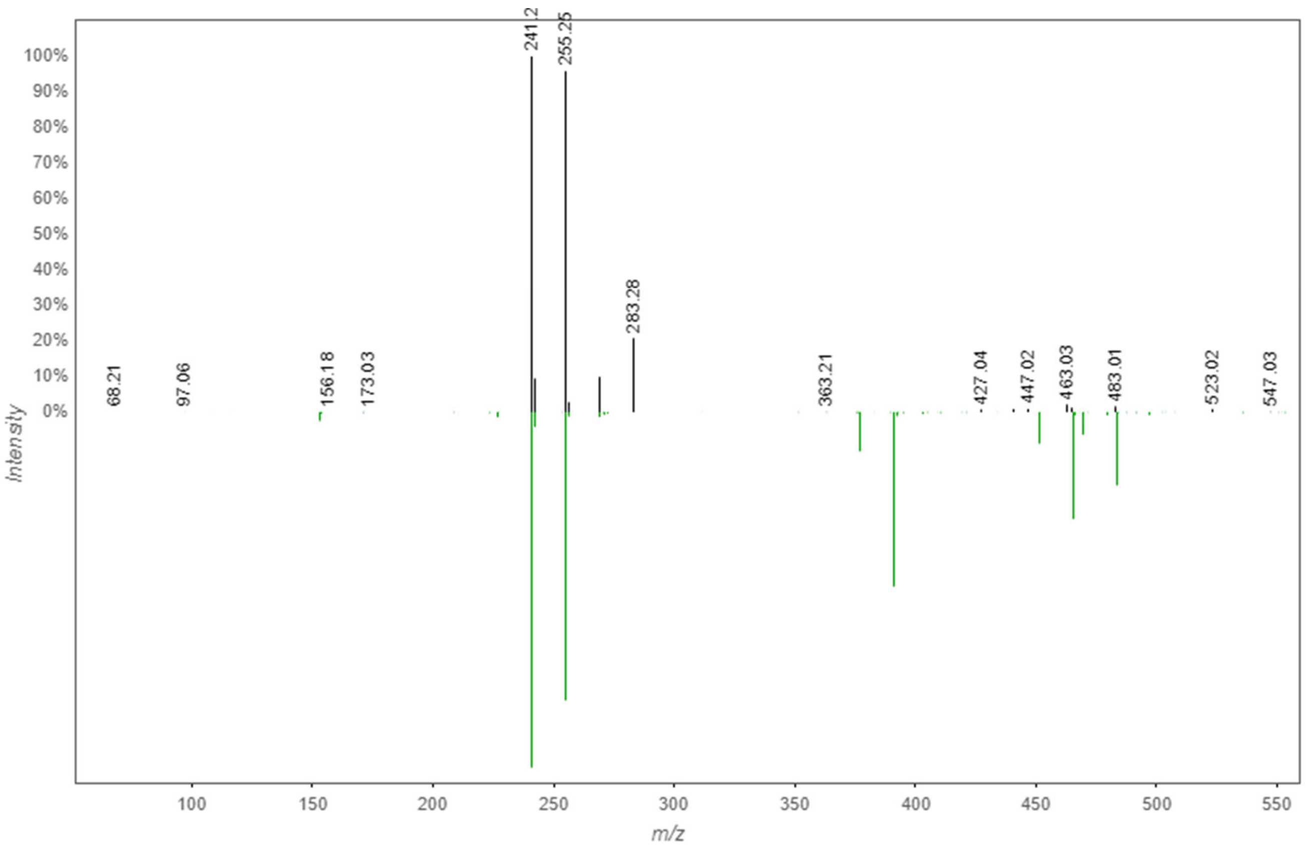

SI Figure 78. GNPS MSMS spectra of **707.60 M/Z PG(15:0/16:0) [M-H]-.**

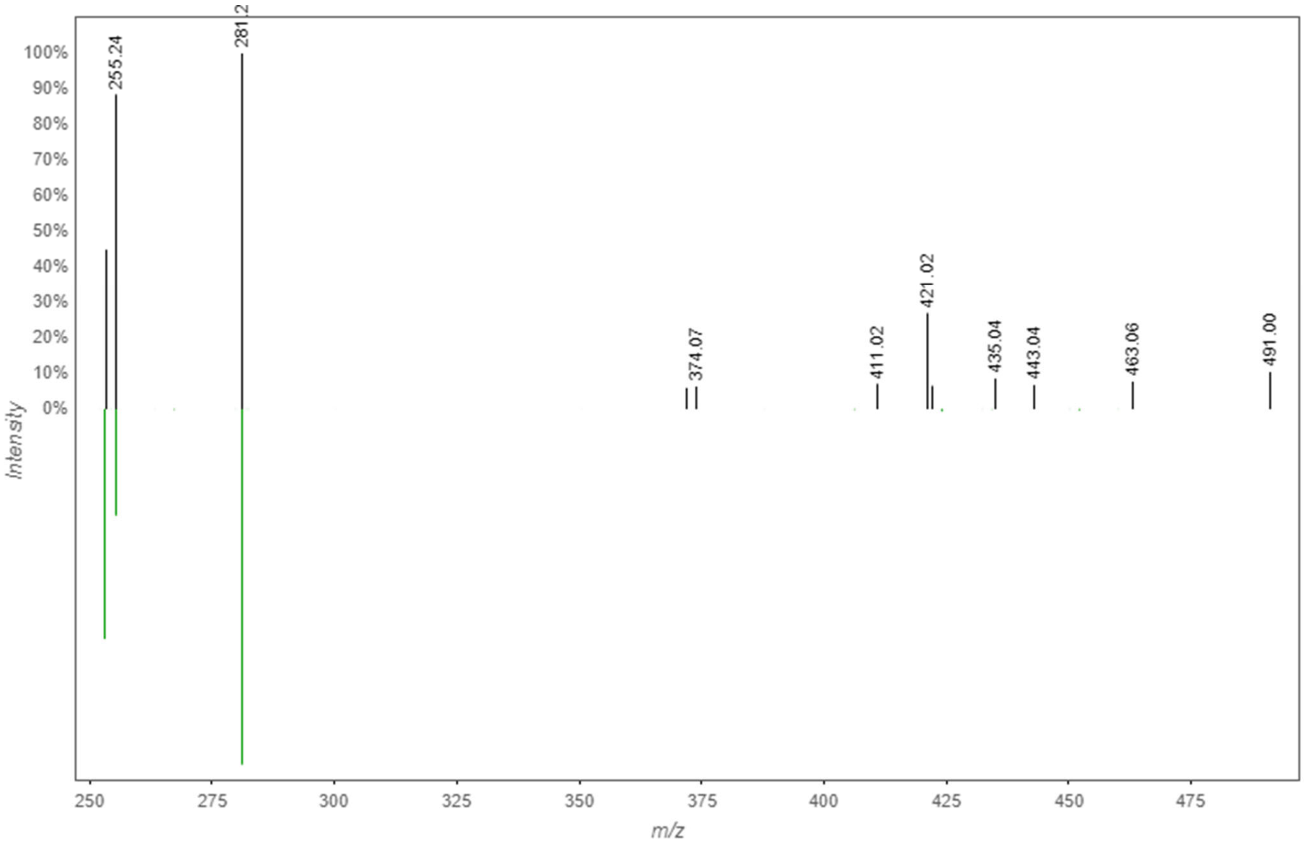

SI Figure 79. GNPS MSMS spectra of **688.60 M/Z PE (16:0/16:1) [M-H]-.**

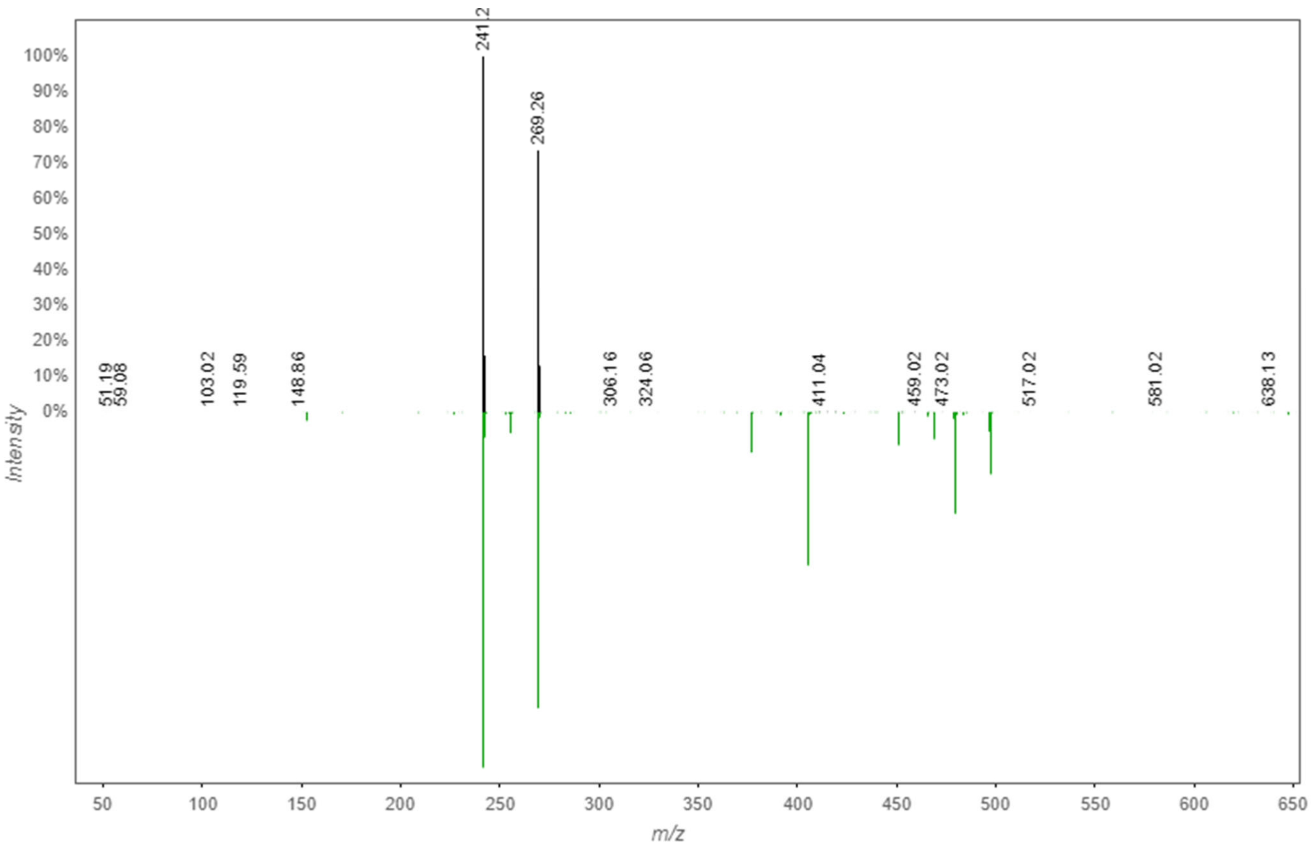

SI Figure 80. GNPS MSMS spectra of **721.50 m/z PG (15:0/17:0) [M-H]-.**

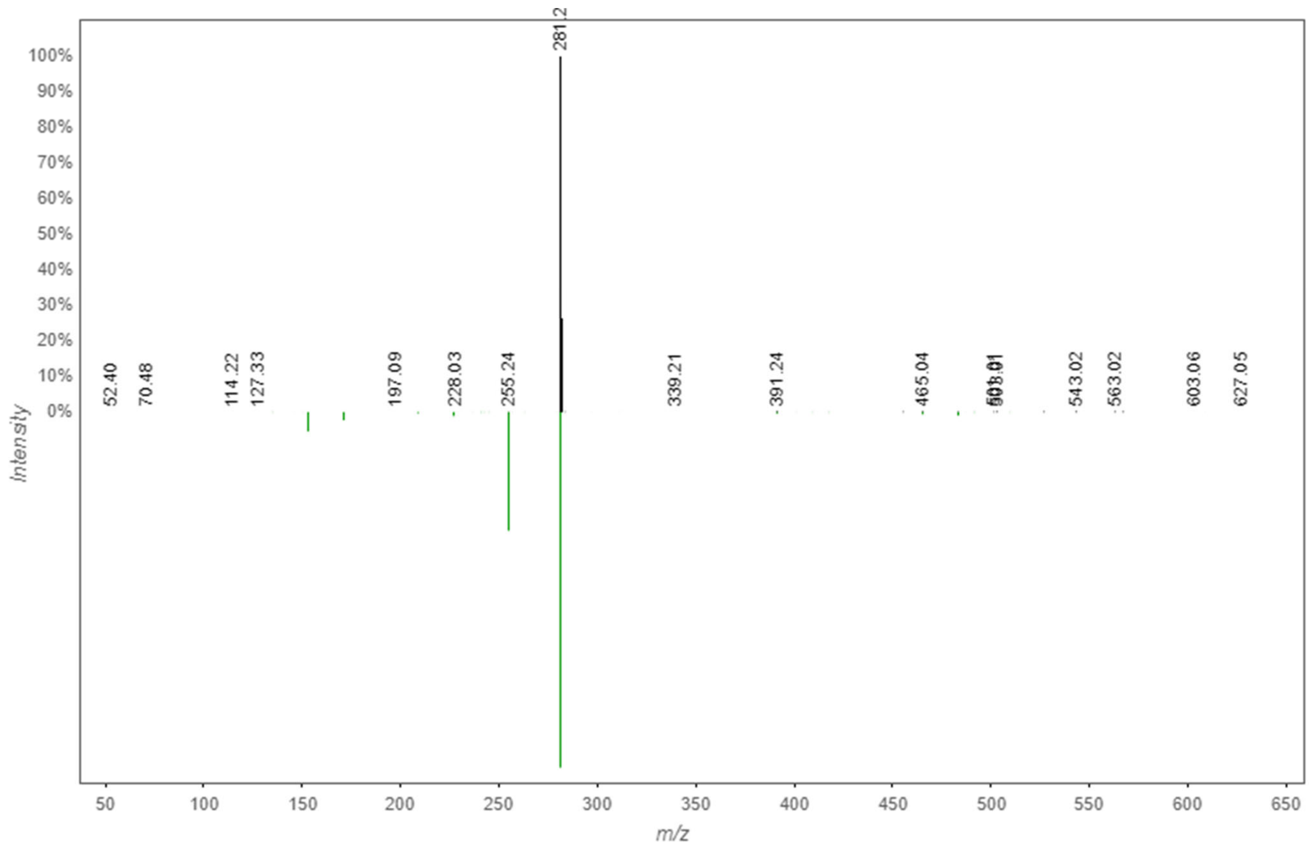

SI Figure 81. GNPS MSMS spectra of **747.61 M/Z PG (16:0/18:1) [M-H]-.**

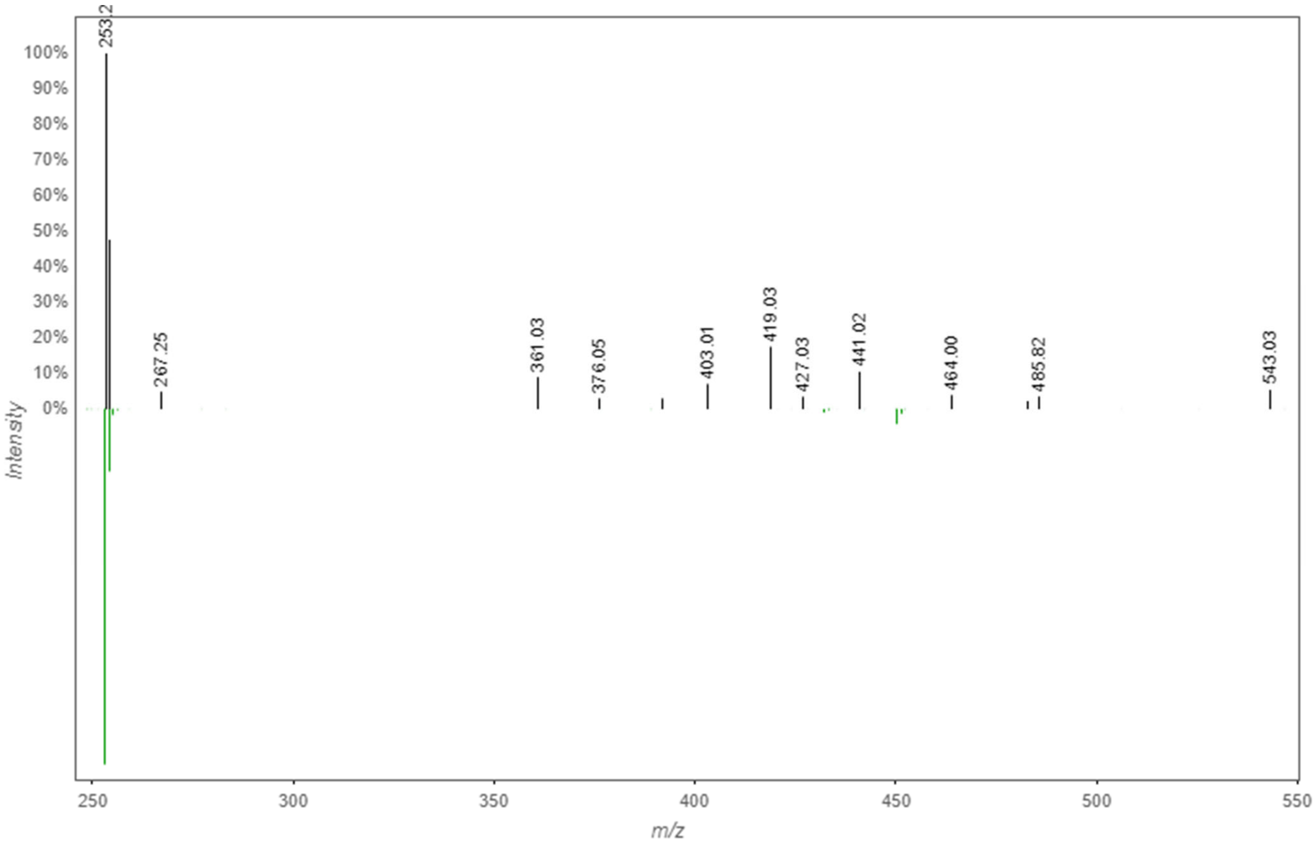

SI Figure 82. GNPS MSMS spectra of **686.60 m/z PE (16:1/16:1) [M-H]-.**

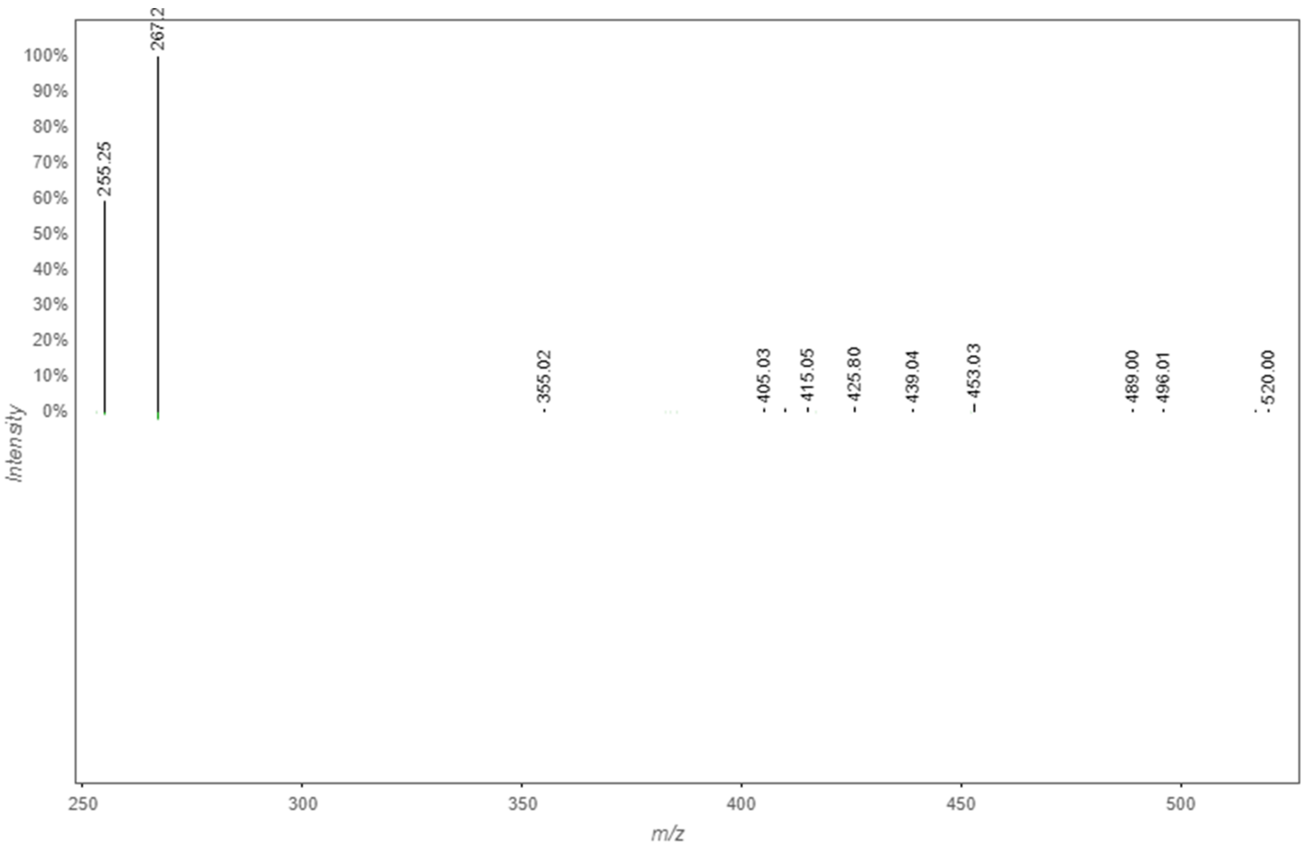

SI Figure 83. GNPS MSMS spectra of **728.61 M/Z PE (17:0/18:1) [M-H]-.**

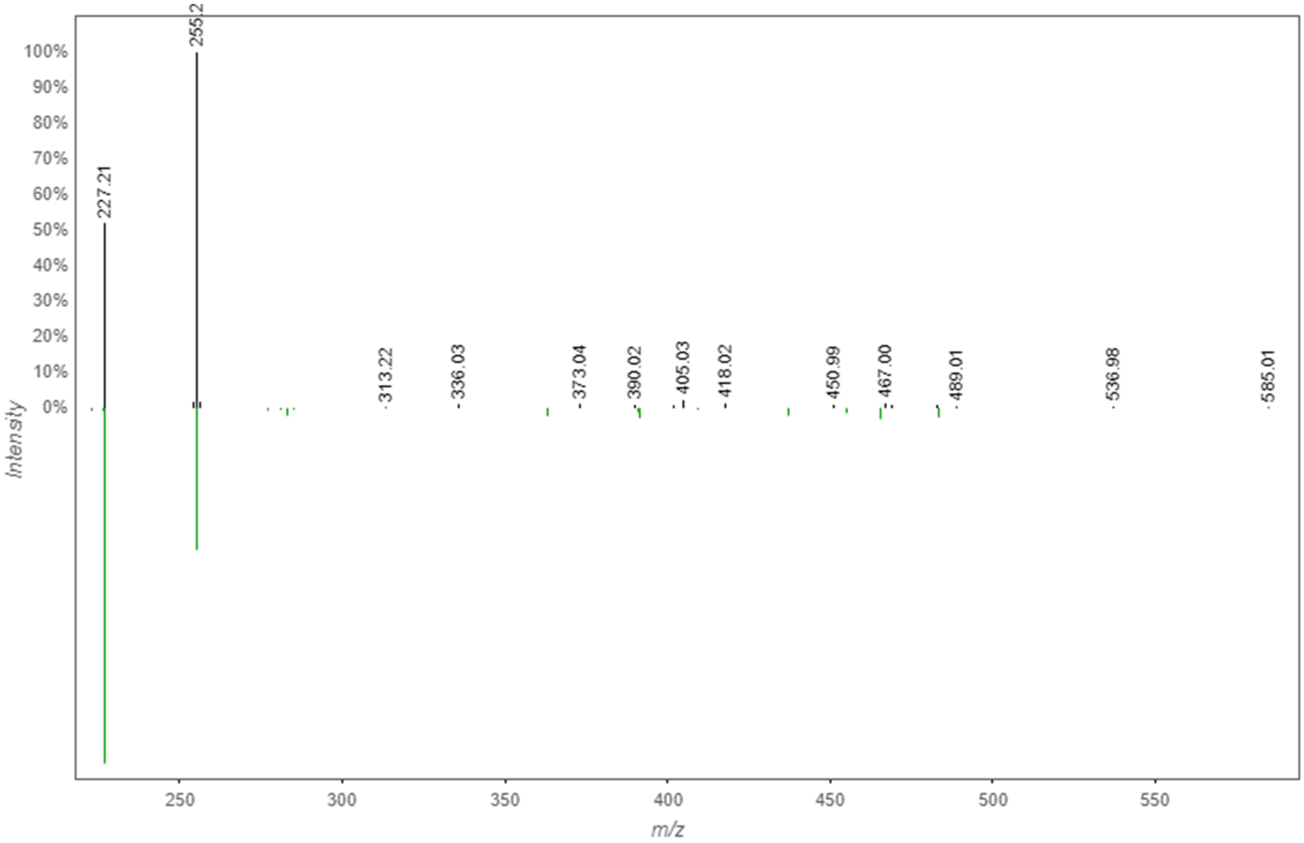

SI Figure 84. GNPS MSMS spectra of **693.60 M/Z PG 30:0 [M-H]-.**

GNPS MSMS Spectra
Positive Mode

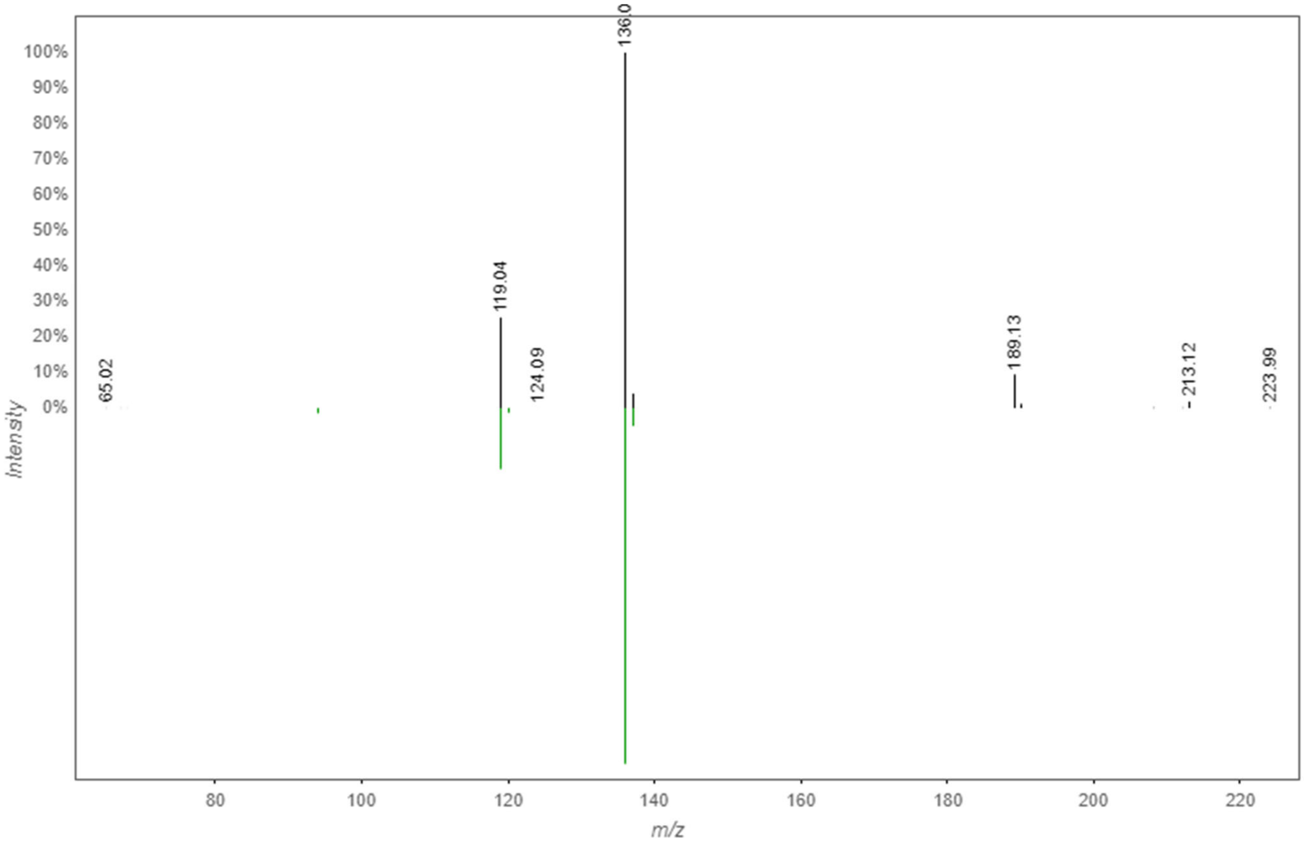

SI Figure 85. GNPS MSMS spectra of **Adenosine 268.10 m/z [M+H]+.**

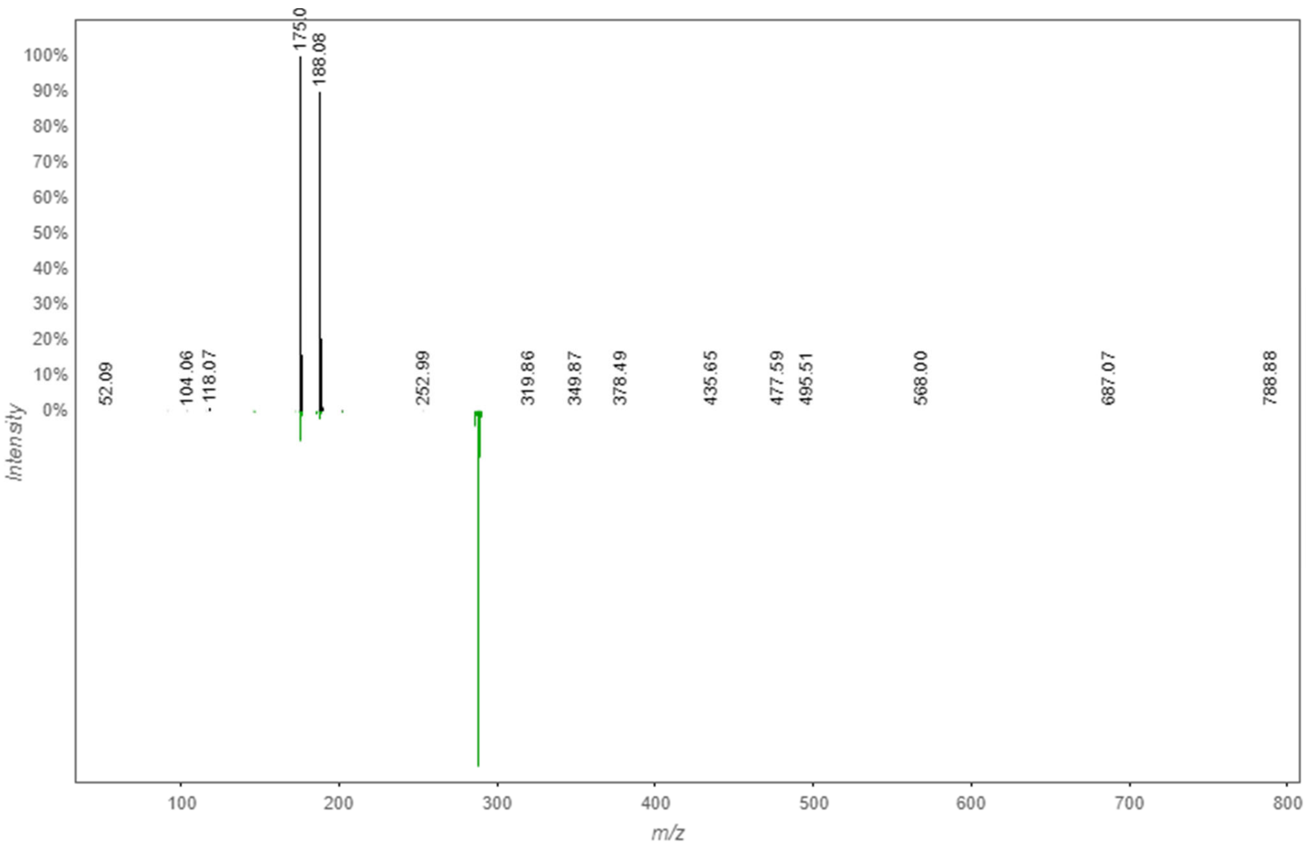

SI Figure 86. GNPS MSMS spectra of **2-heptylquinoline-3,4-diol Pseudomonas quinolone signal 288.68 m/z [M+H]+.**

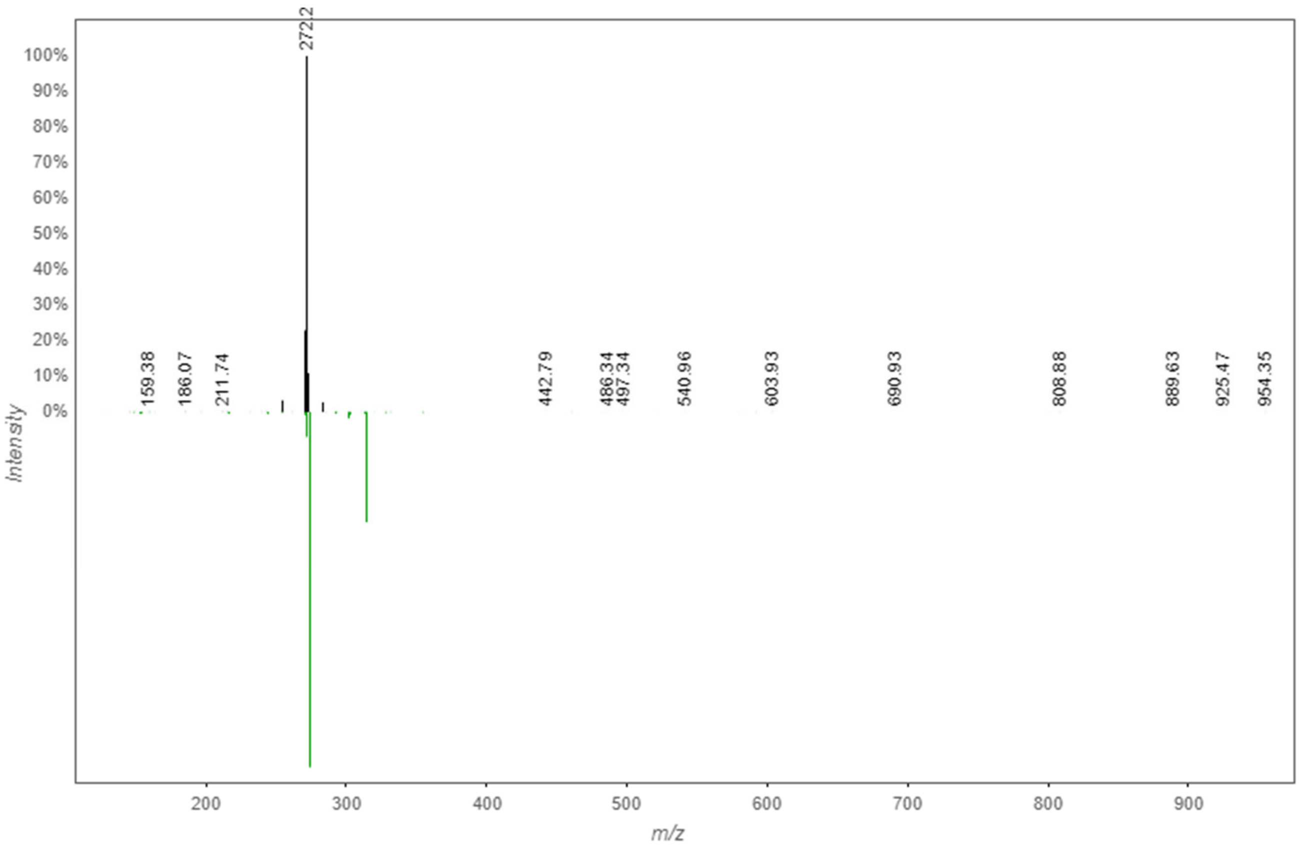

SI Figure 87. GNPS MSMS spectra of **2-[2-(4-methylphenyl)-4-oxochromen-3-yloxy]ethanenitrile 312.32 m/z [M+H]+.**

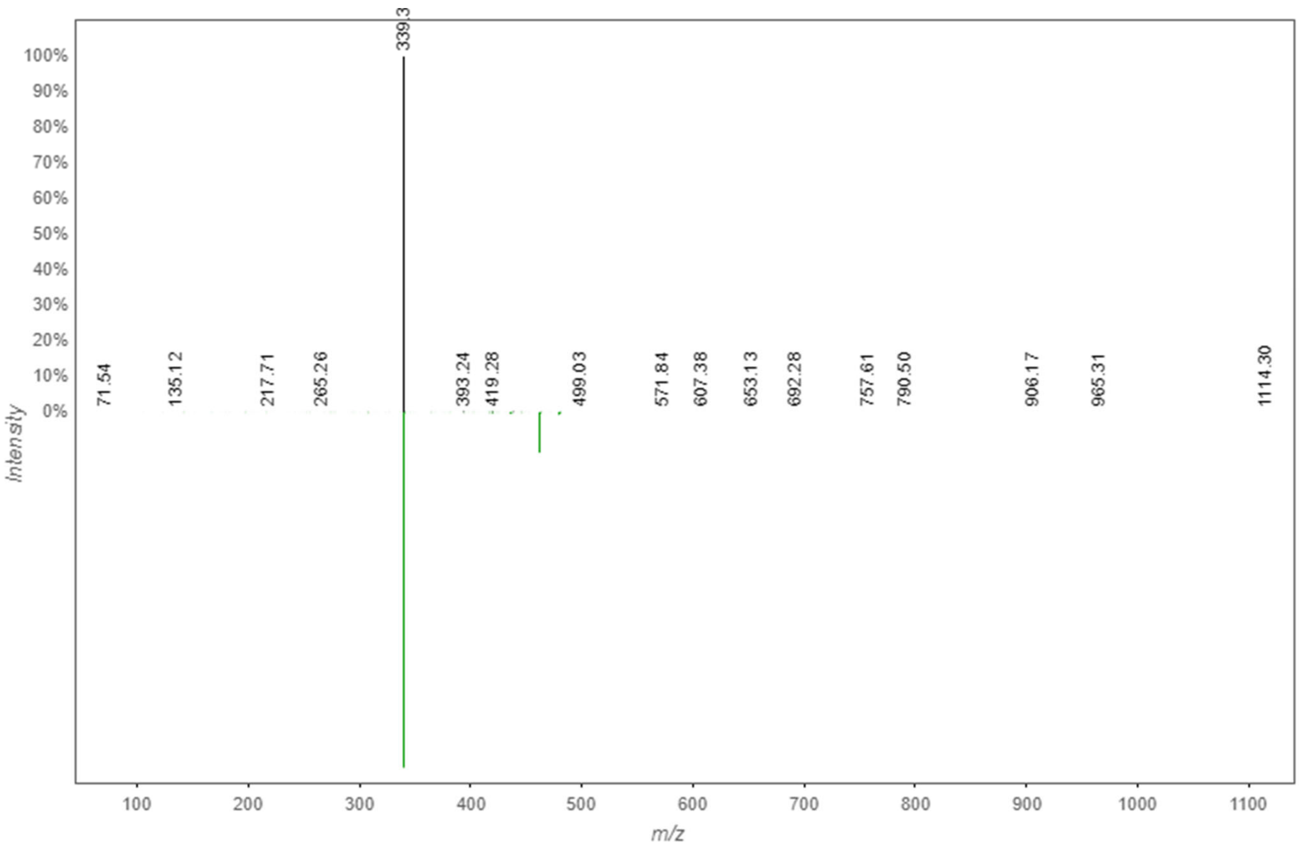

SI Figure 88. GNPS MSMS spectra of **480.72 m/z lysoPE(18:1/0:0) [M+H]+.**

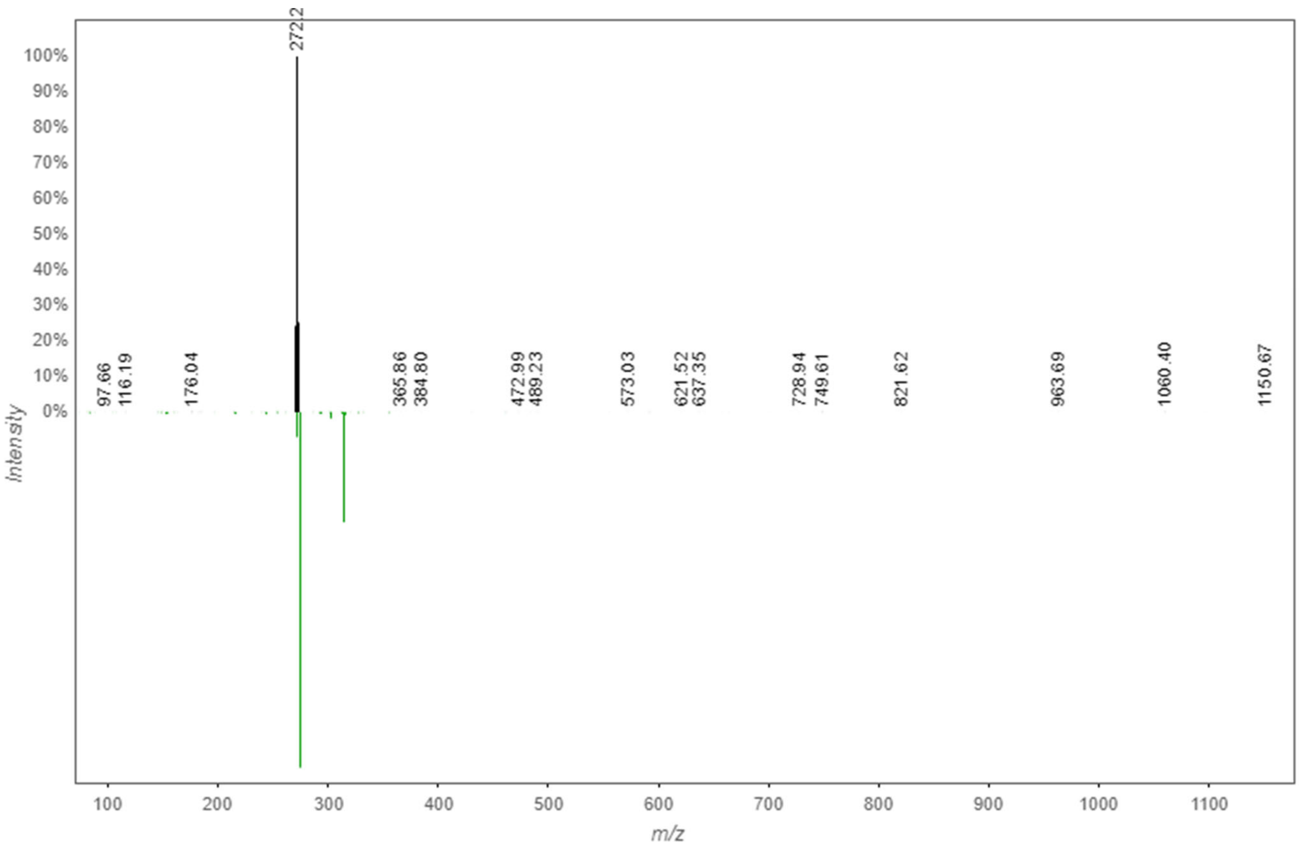

SI Figure 89. GNPS MSMS spectra of **312.32 m/z 2-[2-(4-methylphenyl)-4-oxochromen-3-yloxy]ethanenitrile [M+H]+.**

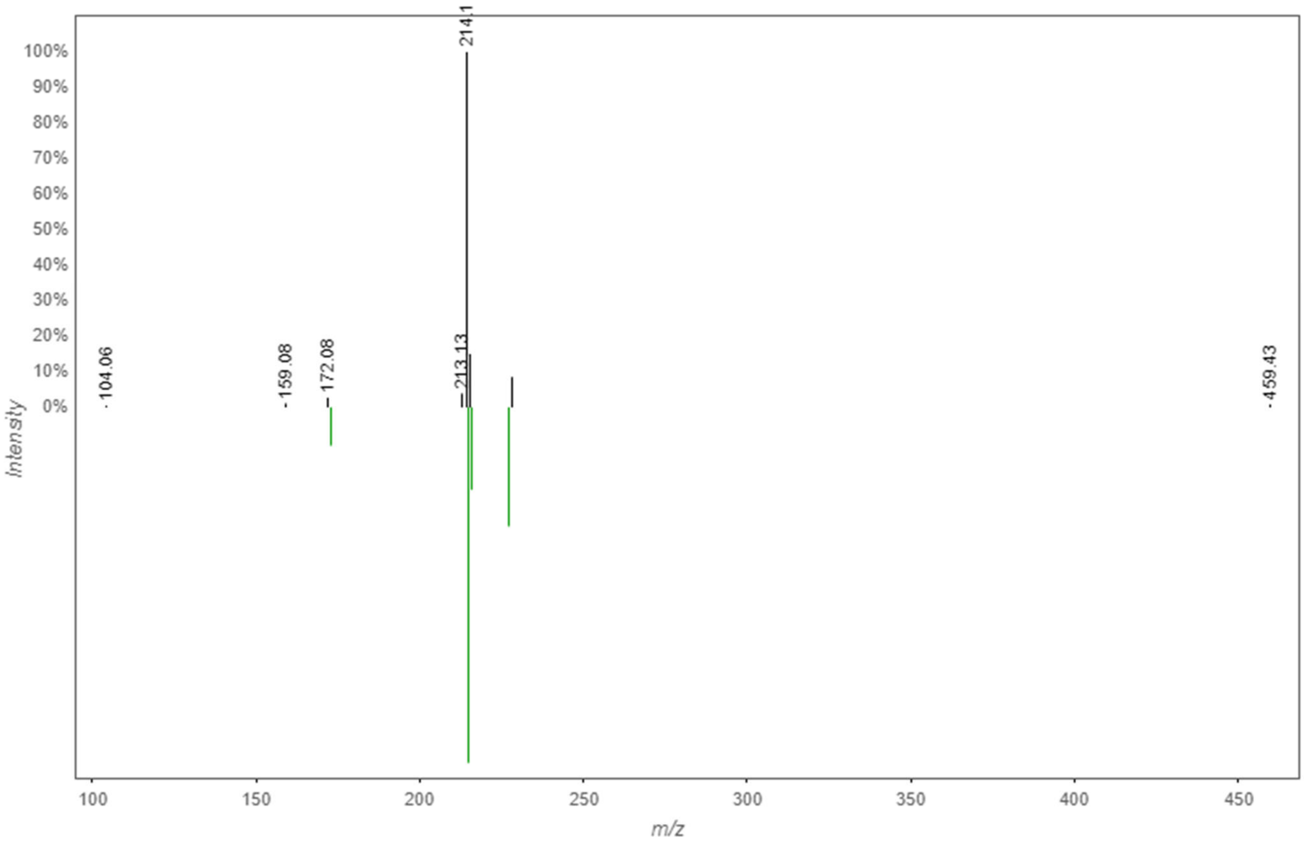

SI Figure 90. GNPS MSMS spectra of **288.41 m/z methyl 3-(4-hydroxy-6-methyl-2-oxo-2H-pyran-3-yl)-3-phenylpropanoate [M+H]+.**

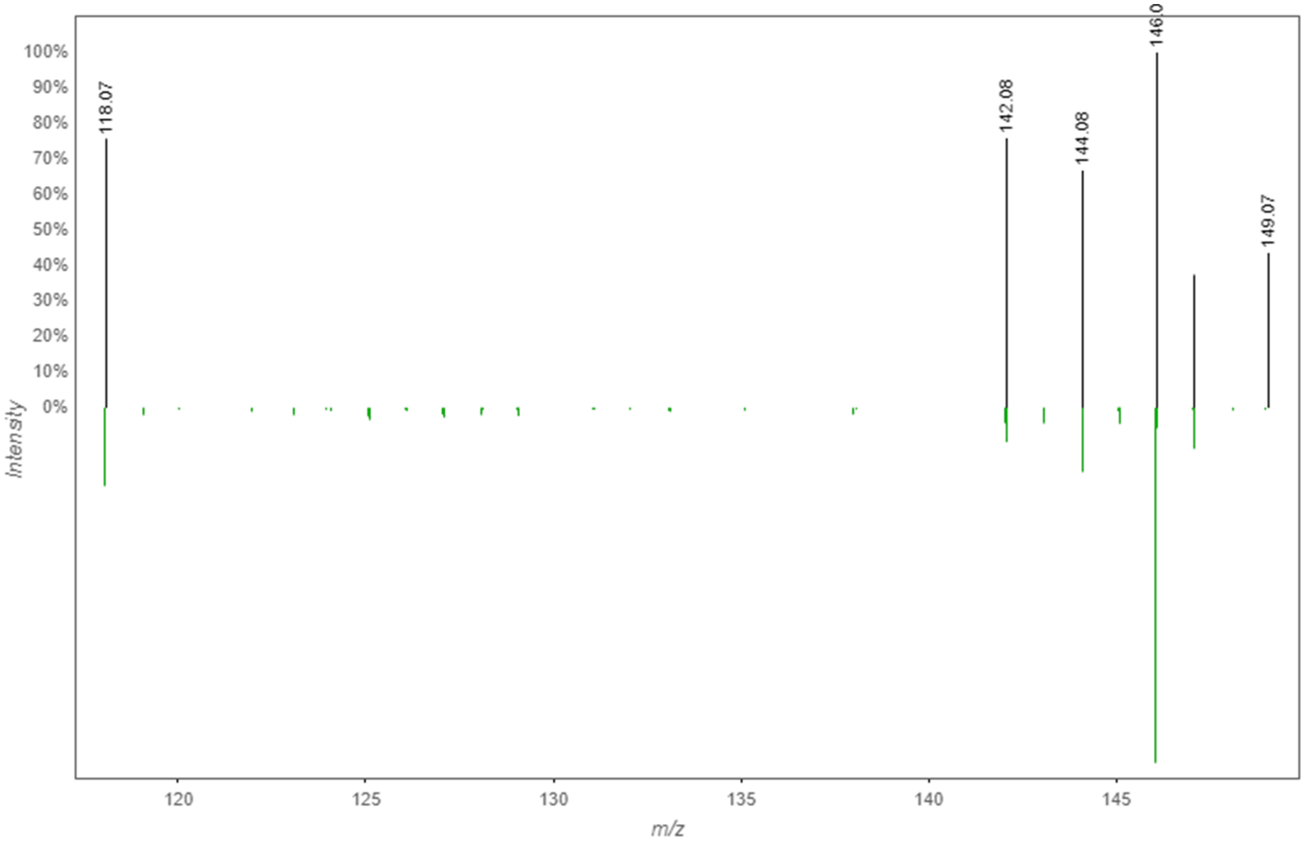

SI Figure 91. GNPS MSMS spectra of **188.18 m/z L-Tryptophan [M+H]+.**

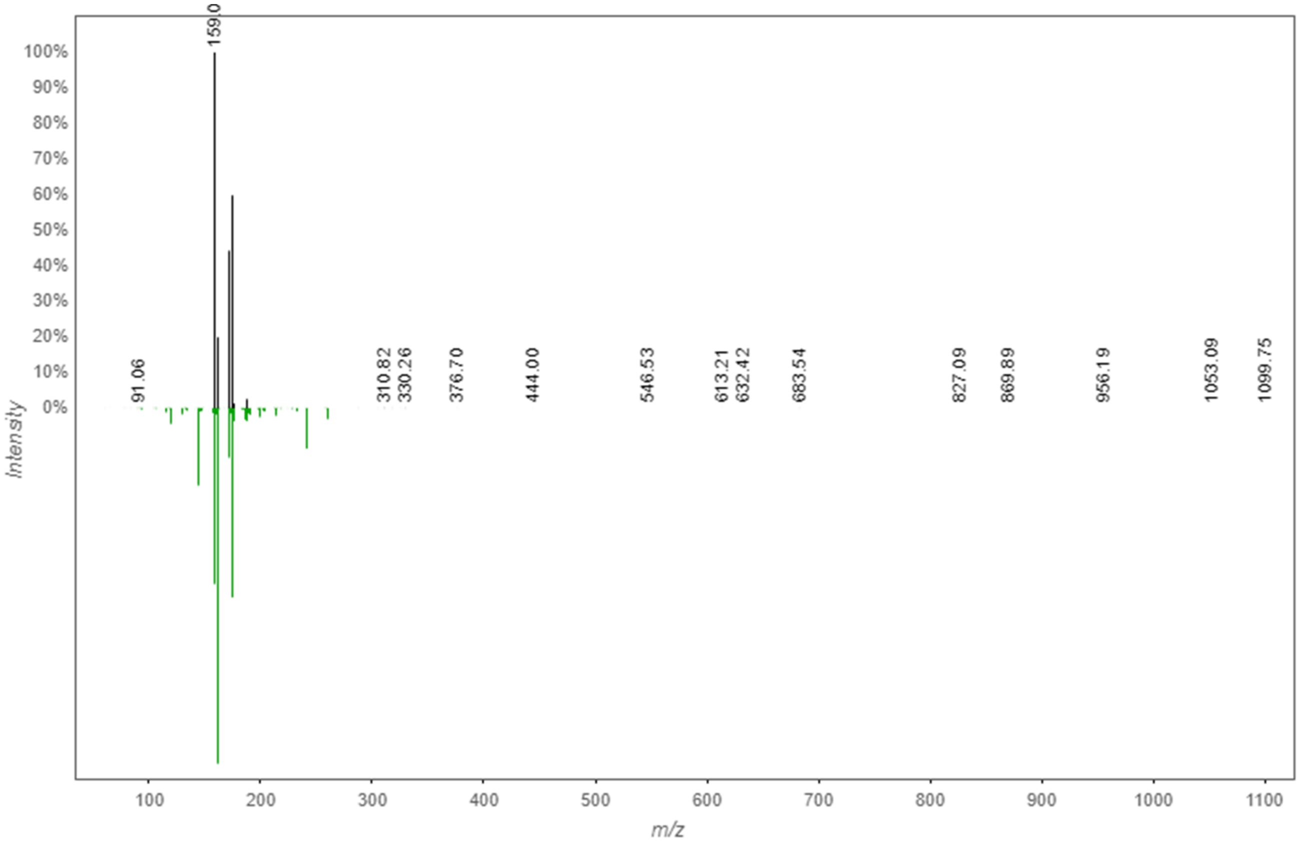

SI Figure 92. GNPS MSMS spectra of **4-hydroxy-2-heptylquinoline N-oxide 258.30 m/z [M+H]+.**

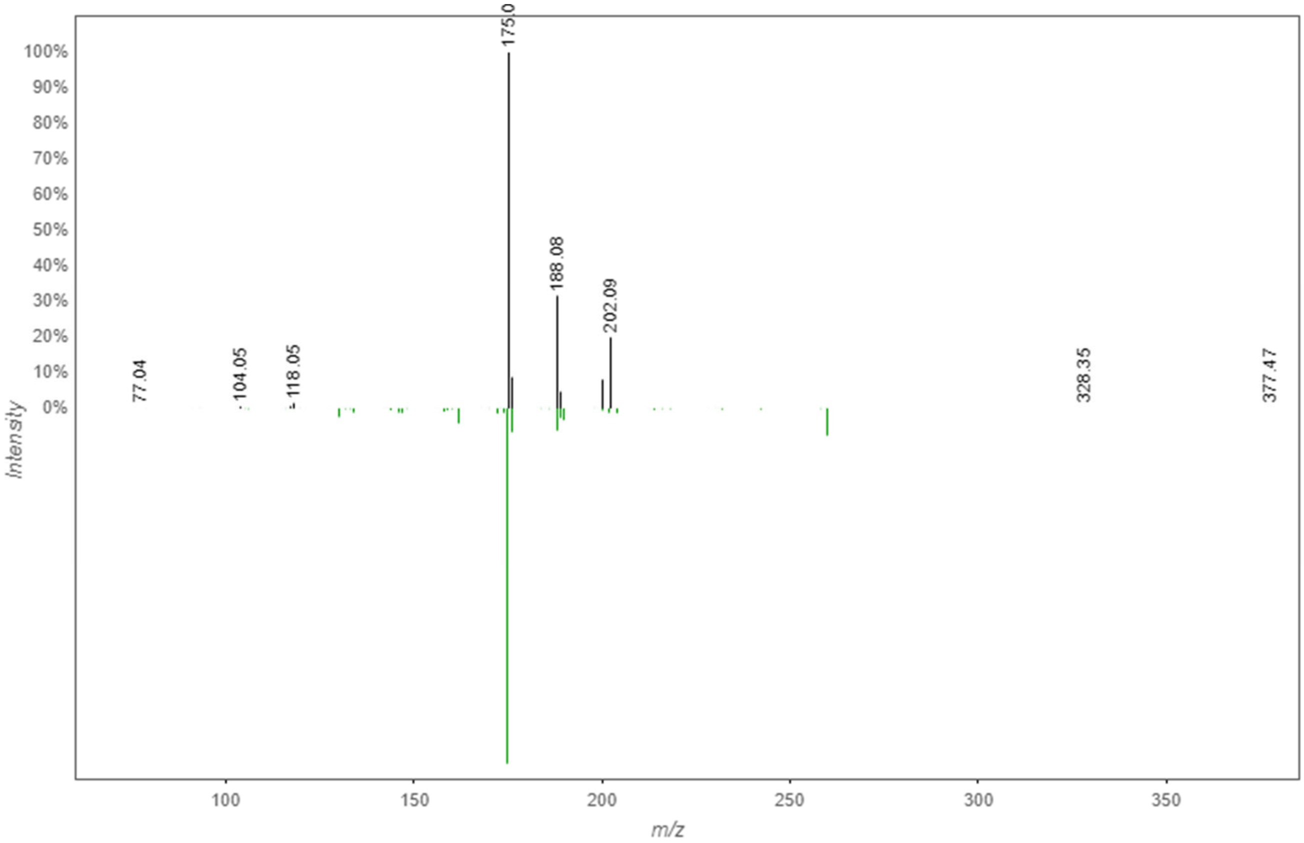

SI Figure 93. GNPS MSMS spectra of **2-heptyl-3-hydroxy 4-quinolone 260.30 m/z [M+H]+.**

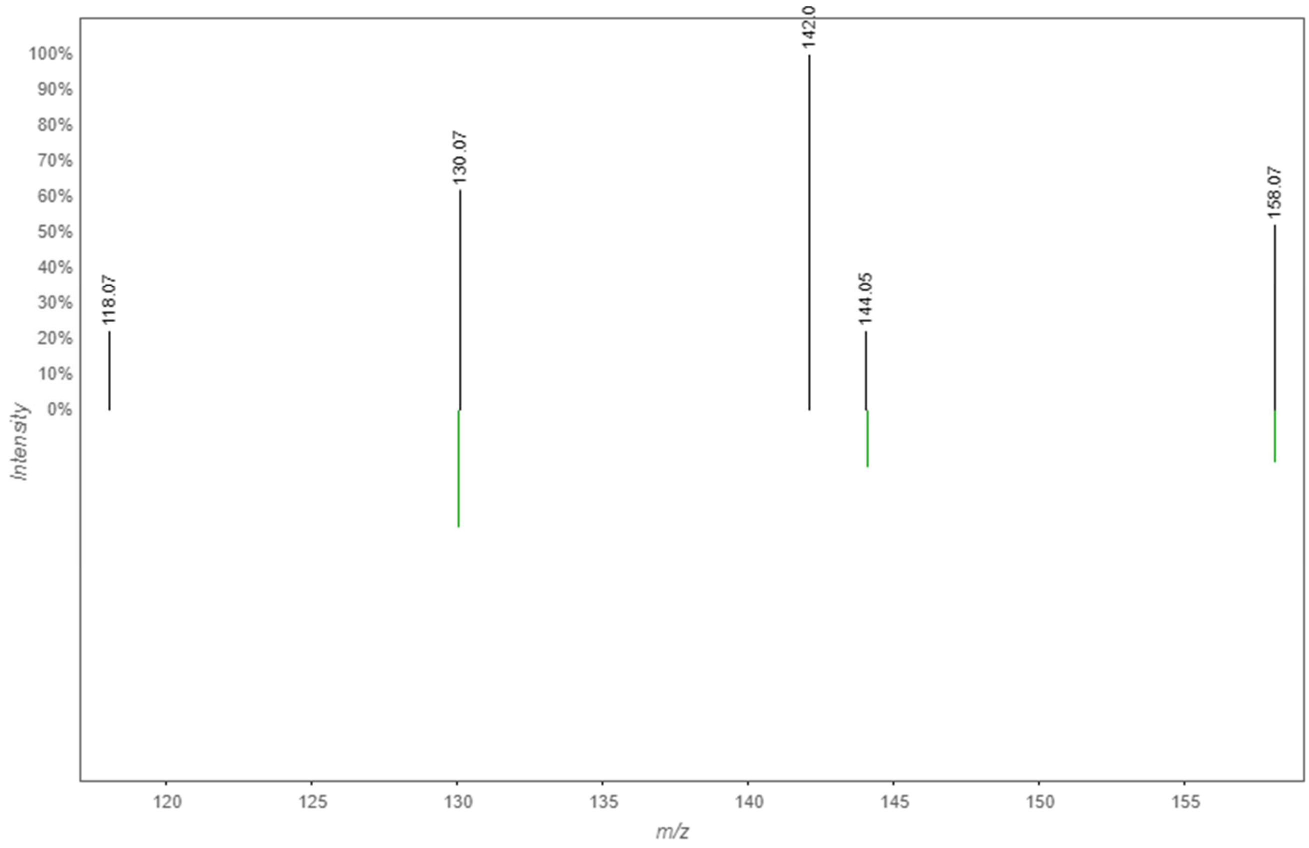

SI Figure 94. GNPS MSMS spectra of **2,3-dihydro-1H-carbazol-4(9H)-one 184.18 m/z [M+H]+.**

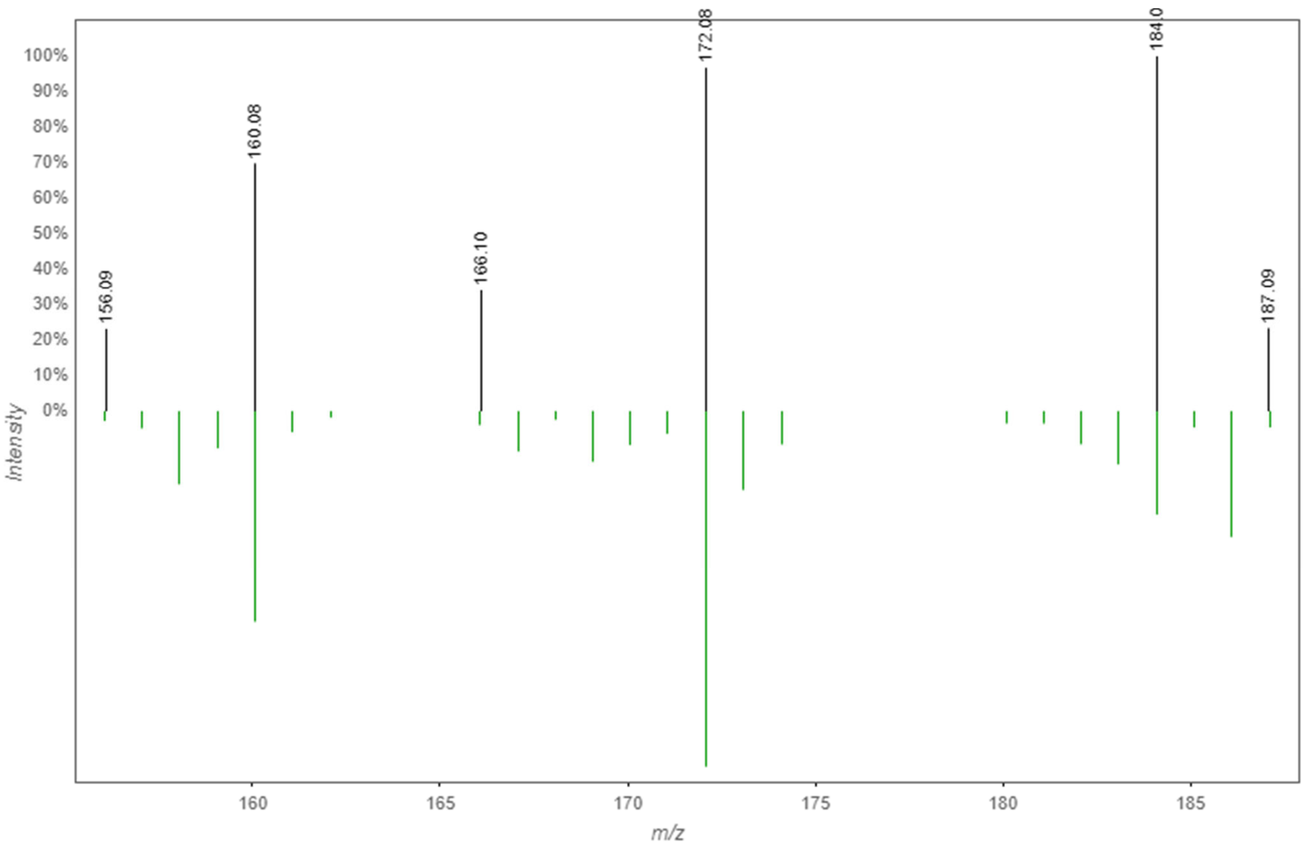

SI Figure 95. GNPS MSMS spectra of **Quinine 326.42 m/z [M+H]+.**

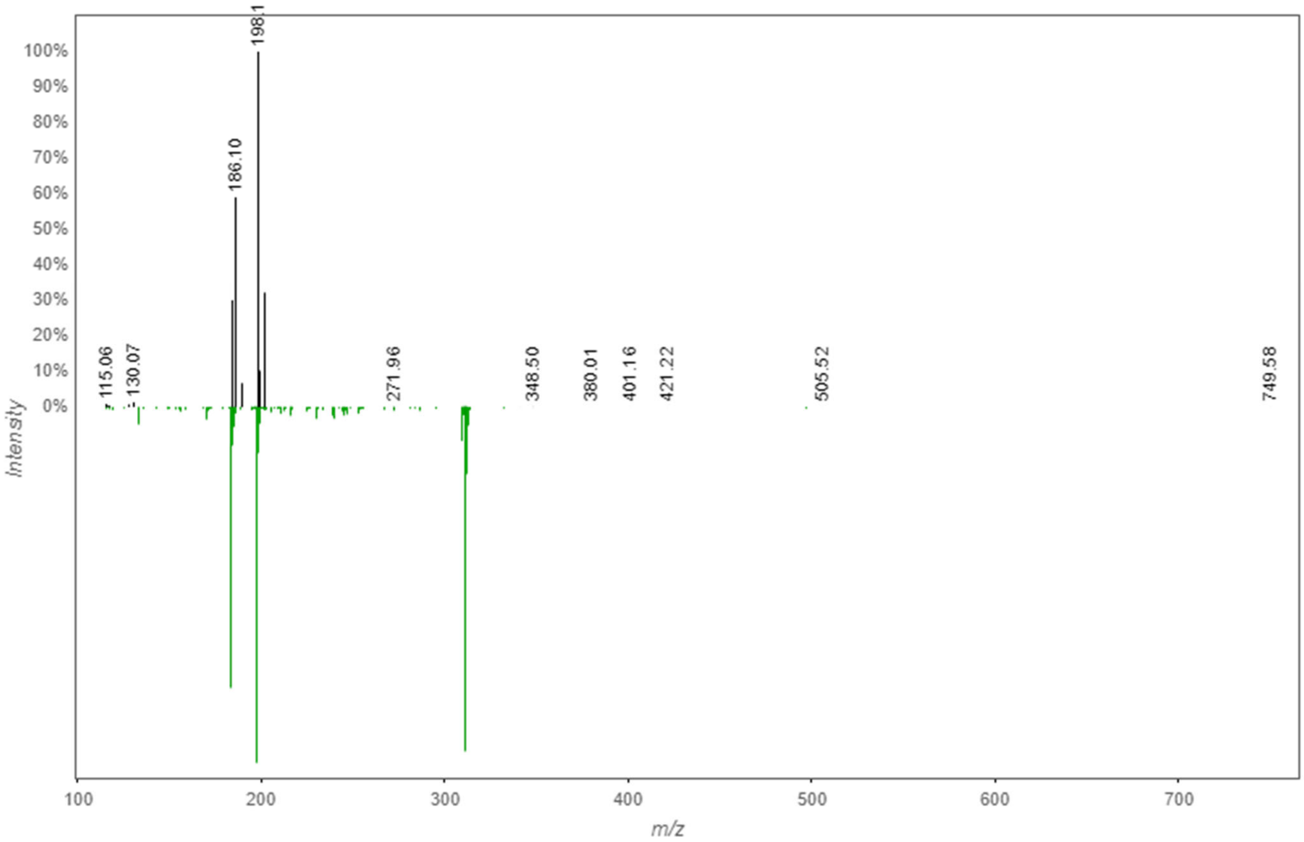

SI Figure 96. GNPS MSMS spectra of **Benzenesulfonic acid 159.15 m/z [M+H]+.**

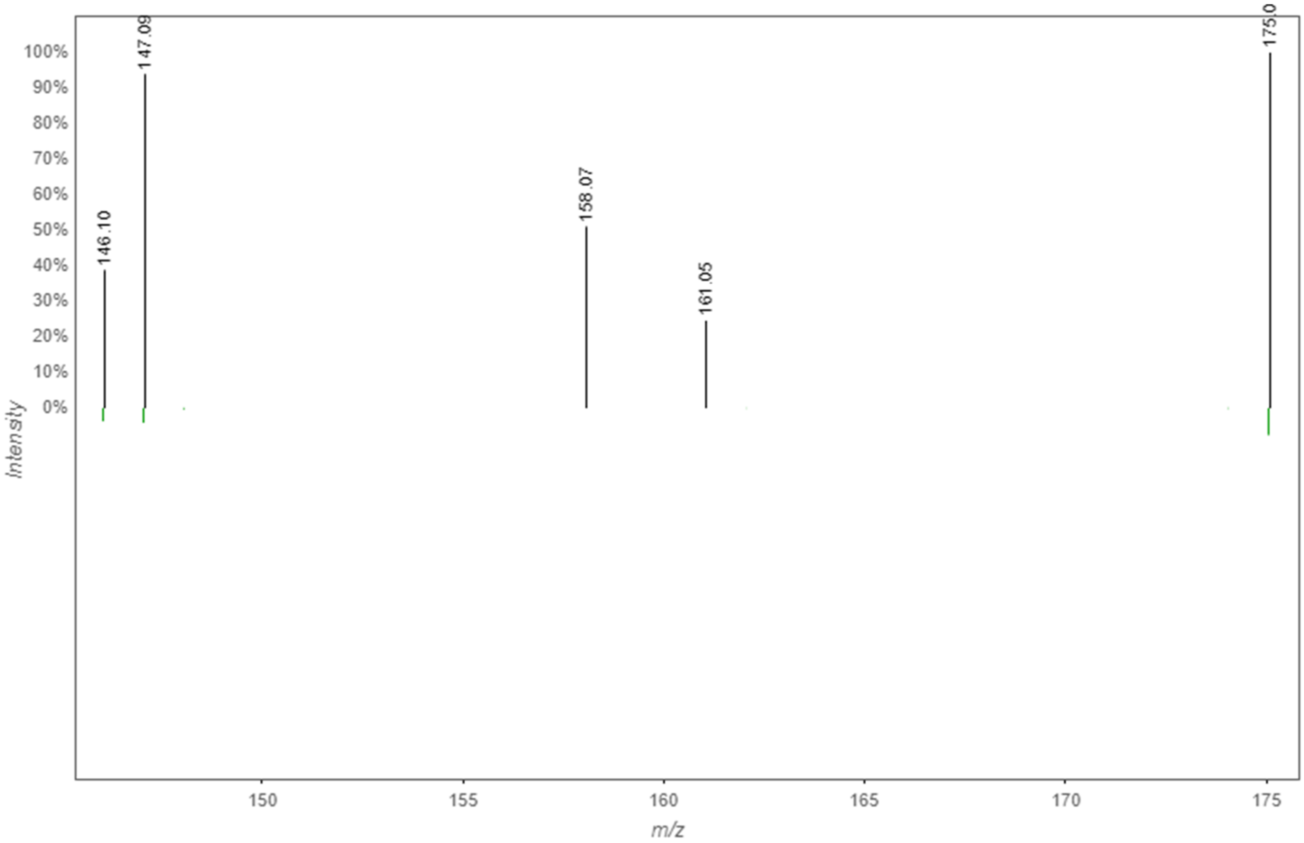

SI Figure 97. GNPS MSMS spectra of **PQS 260.30 m/z [M+H]+.**

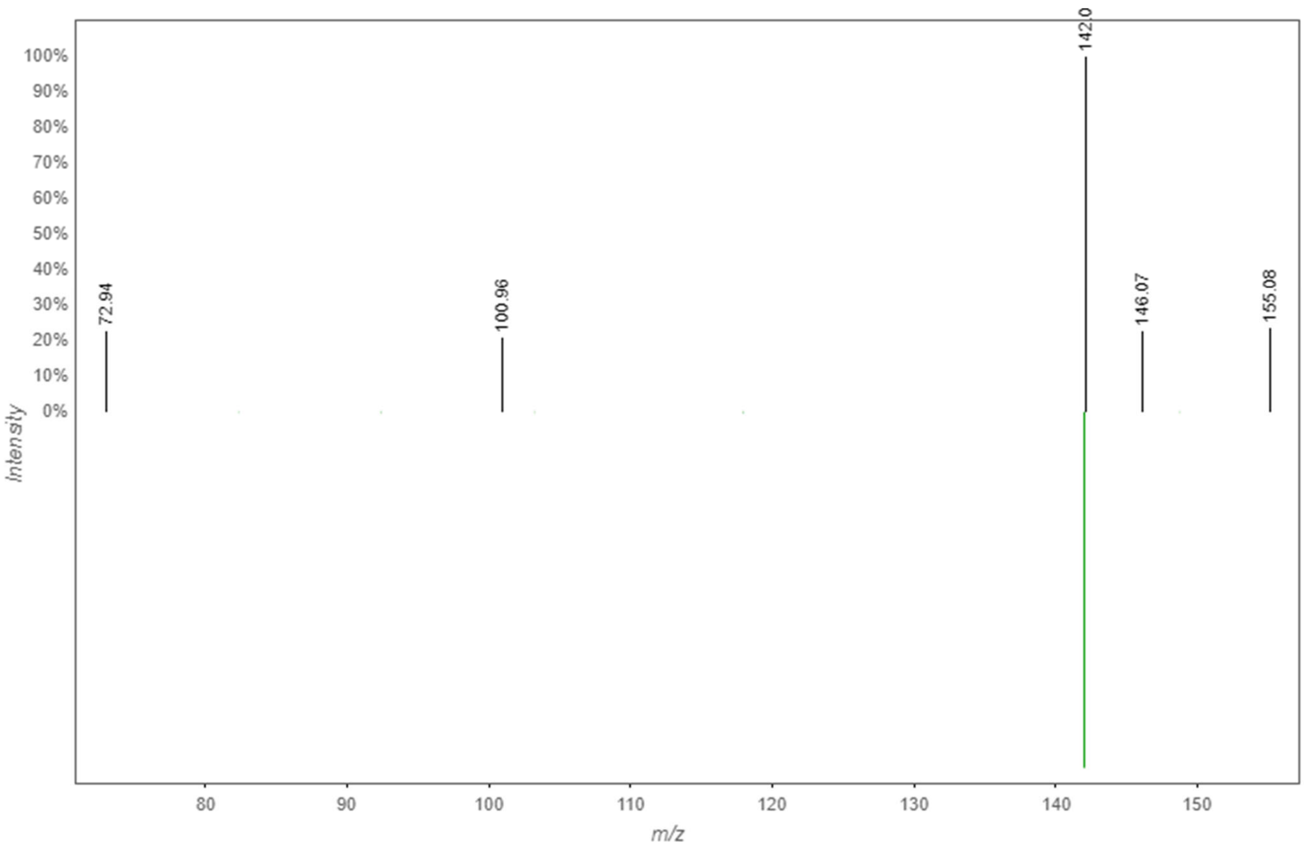

SI Figure 98. GNPS MSMS spectra of **4-methylquinoline-2-carboxylic acid 184.18 m/z [M+H]+.**

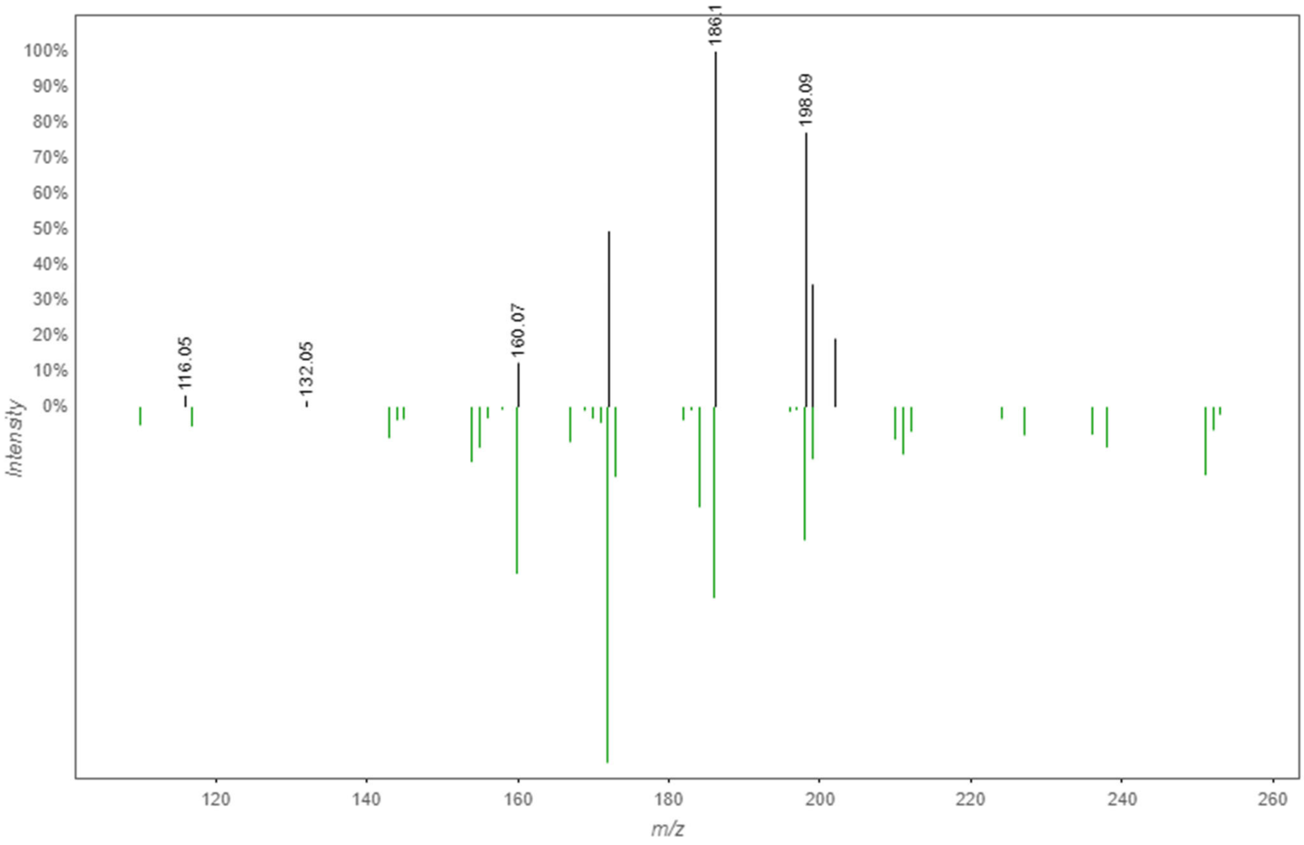

SI Figure 99. GNPS MSMS spectra of **326.42 m/z hydroquinidine [M+H]+.**

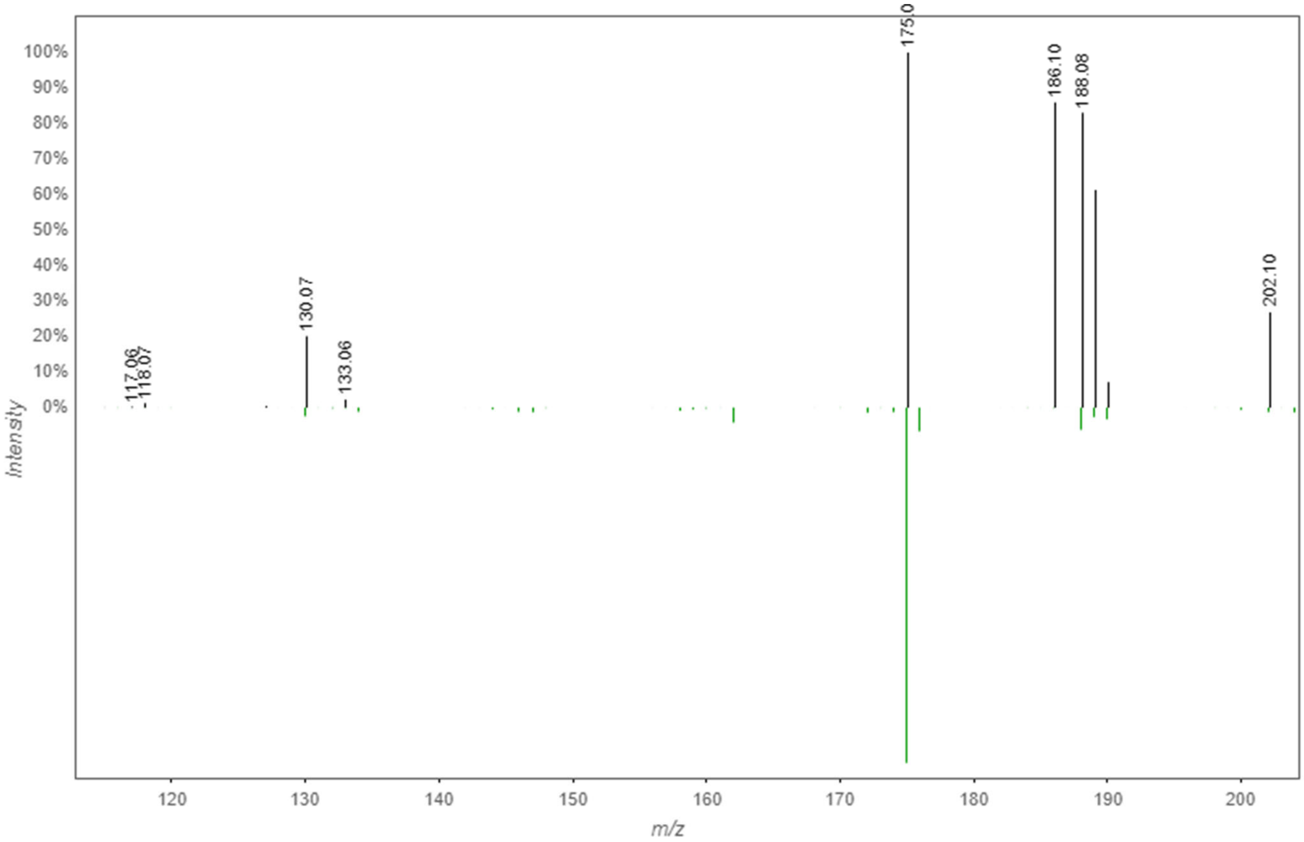

SI Figure 100. GNPS MSMS spectra of **258.30 m/z 2-heptyl-3-hydroxy 4-quinolone [M+H]+.**

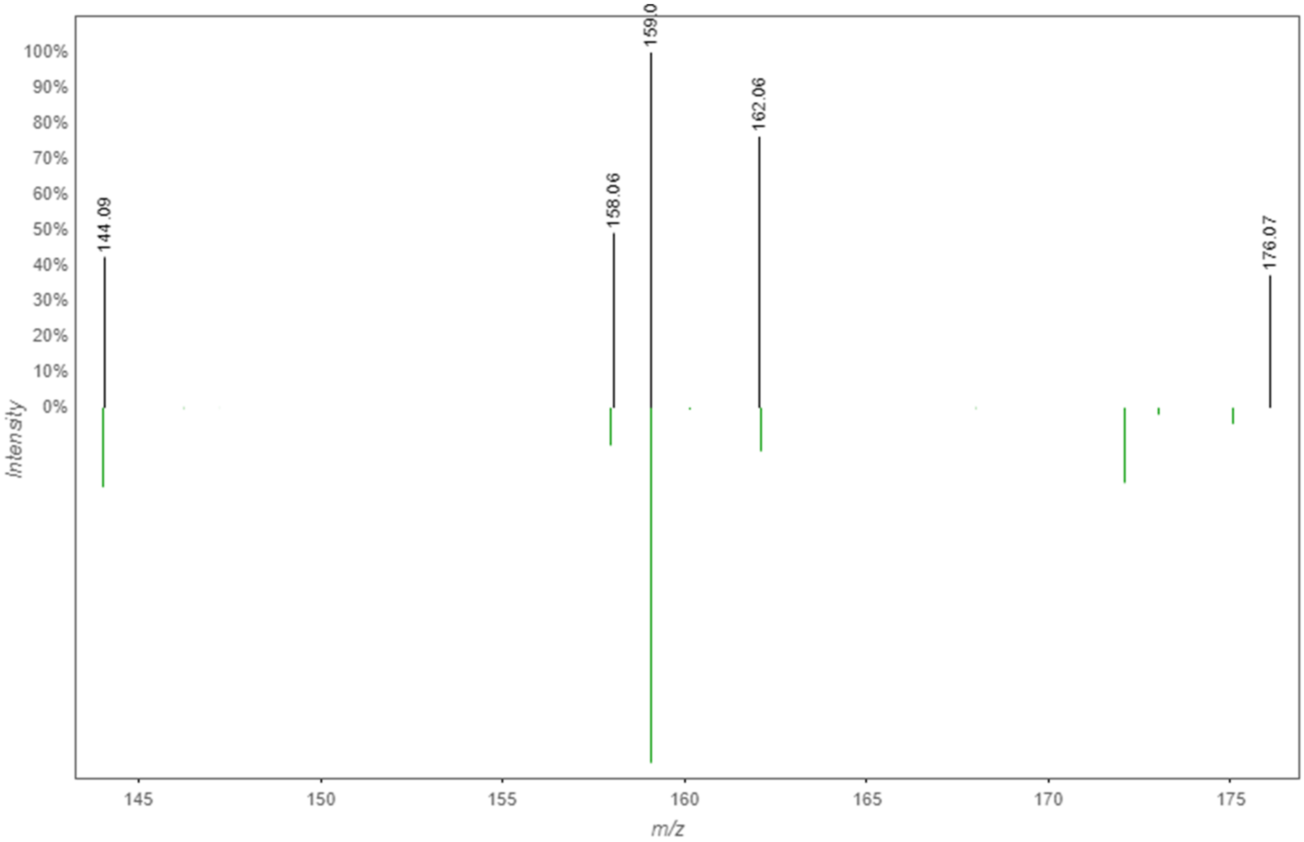

SI Figure 101. GNPS MSMS spectra of **288.41 m/z 2-nonyl-4-quinolone N-oxide (NQNO) [M+H]+.**

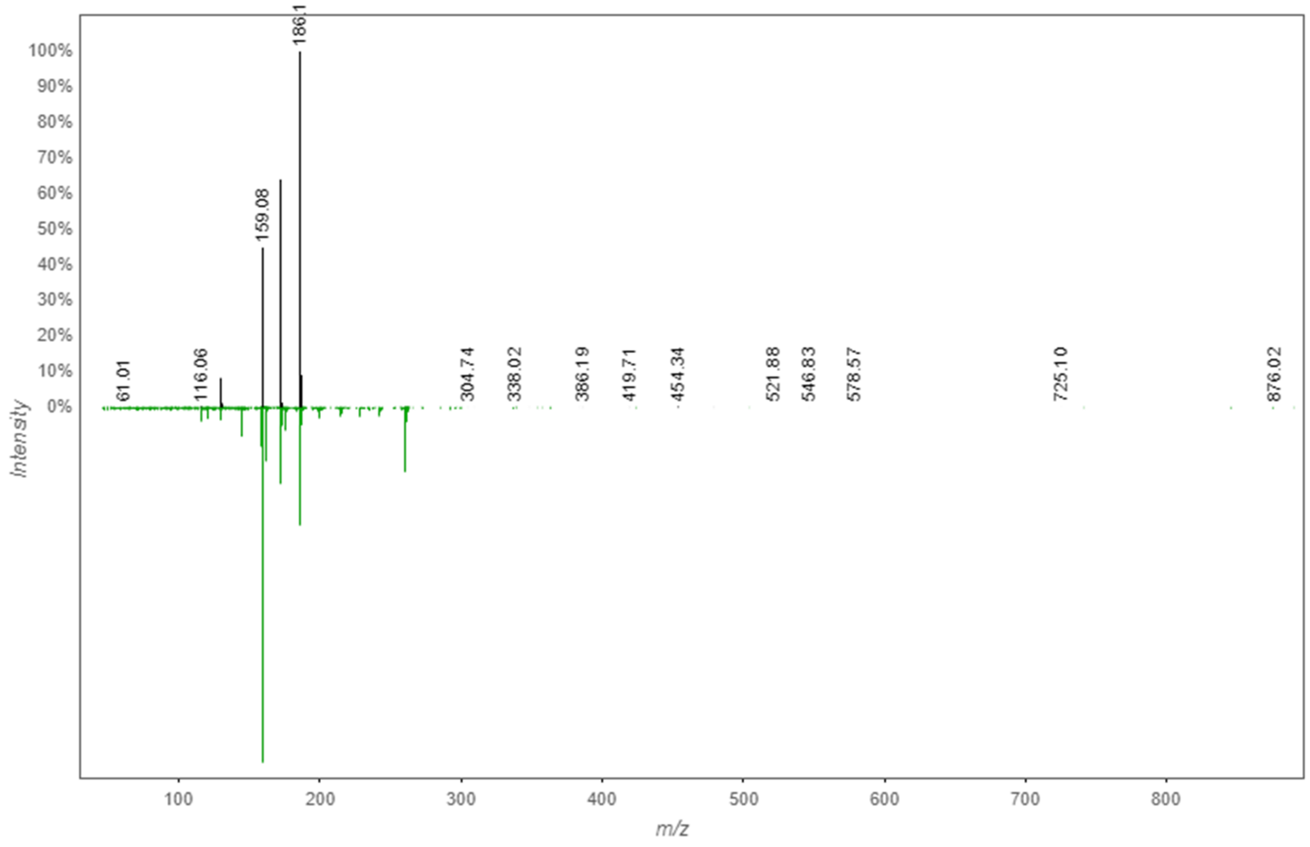

SI Figure 102. GNPS MSMS spectra of **258.30 m/z HQNO (2-heptyl-4-Hydroxyquinolone) [M+H]+.**

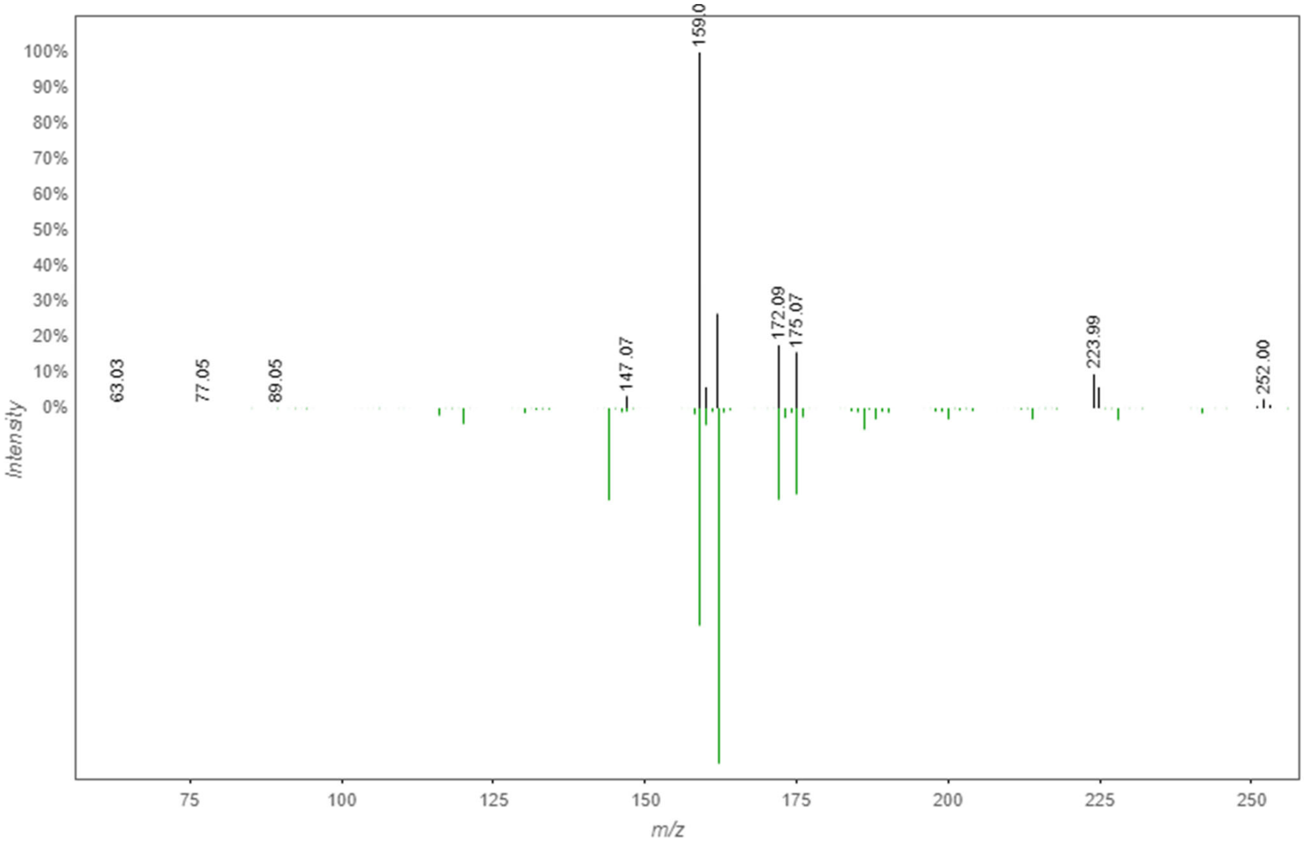

SI Figure 103. GNPS MSMS spectra of **288.41 m/z 2-nonyl-3-hydroxy 4(1H)-quinolone [M+H]+.**

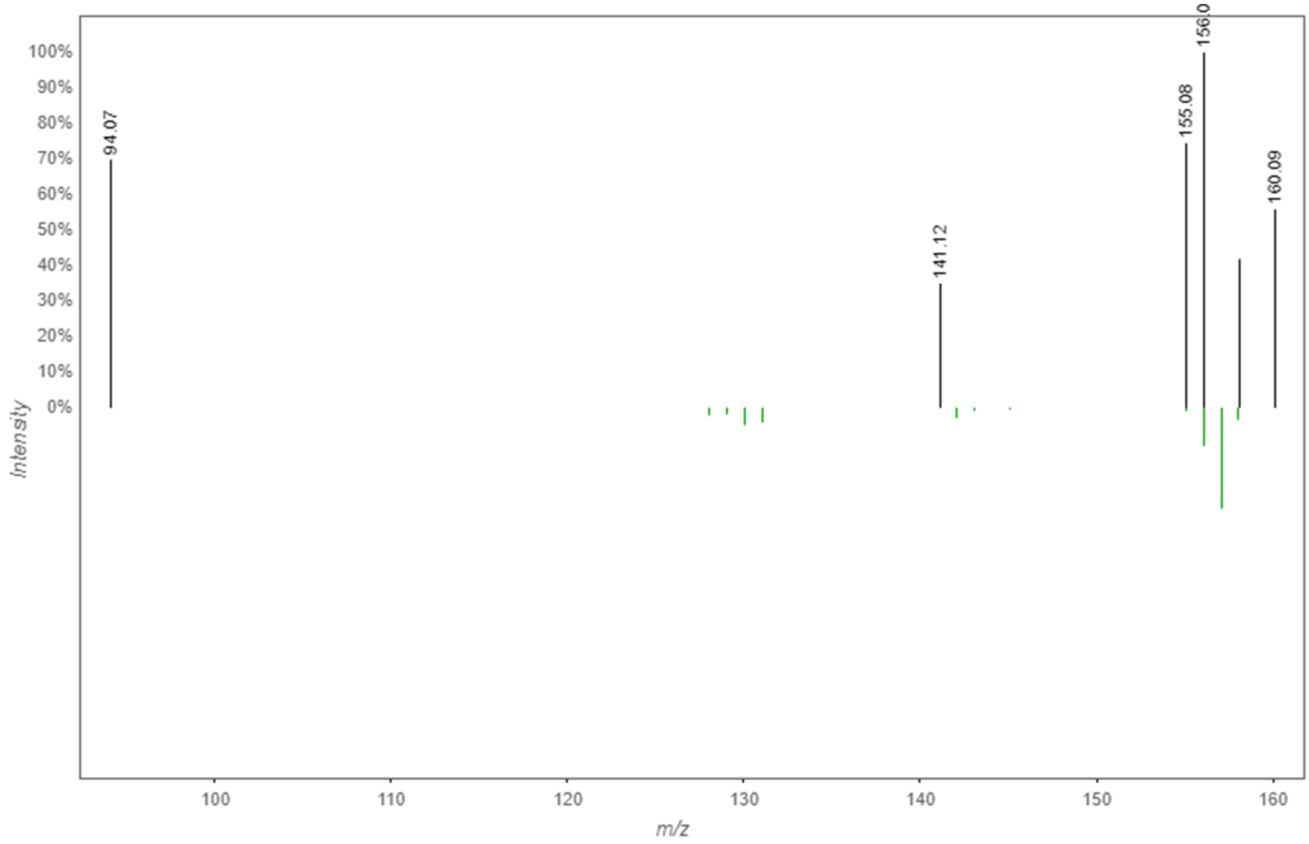

SI Figure 104. GNPS MSMS spectra of **198.18 m/z 2-Amino-3-methyl-imidazo[4,5-f]quinoline (IQ) [M+H]+.**

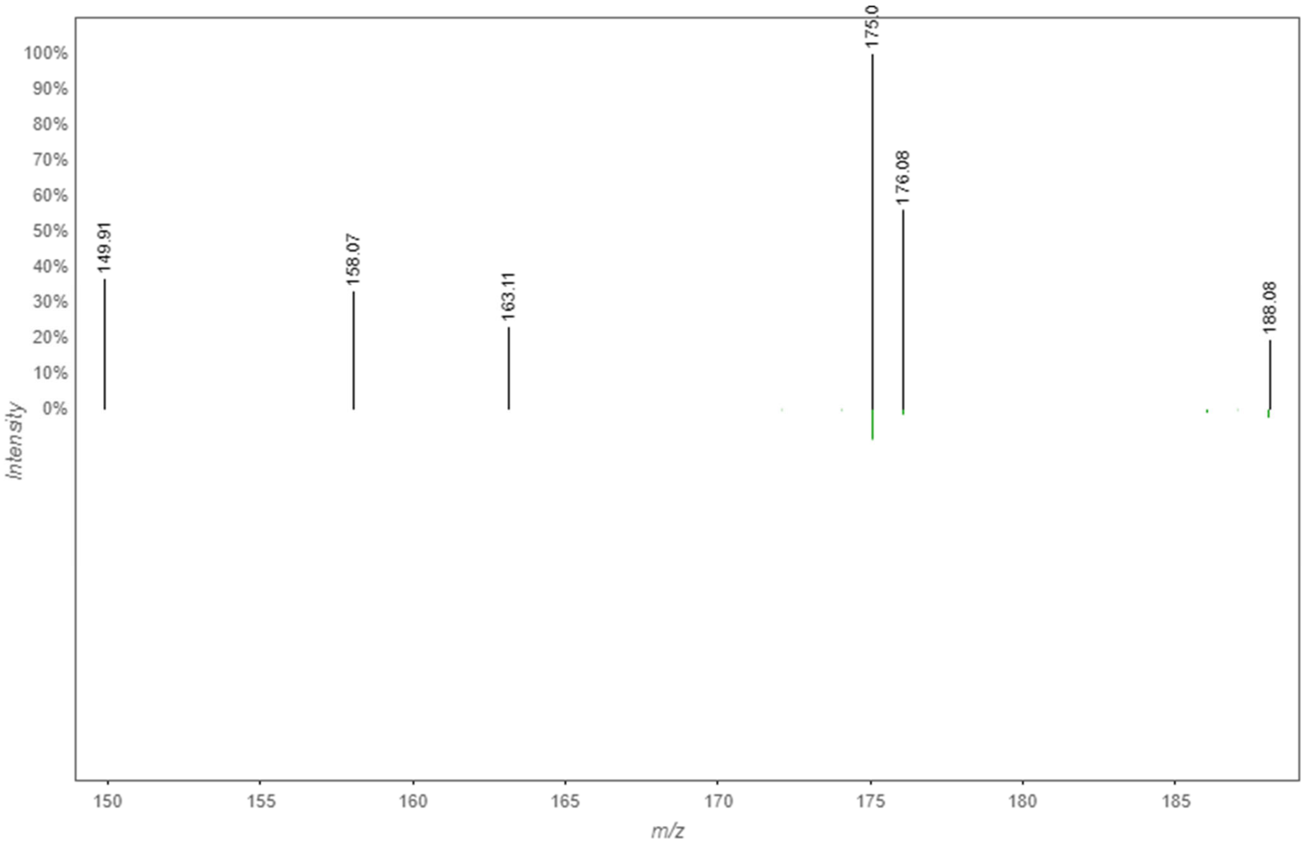

SI Figure 105. GNPS MSMS spectra of **289.31 m/z 2-heptylquinoline-3,4-diol [M+H]+.**

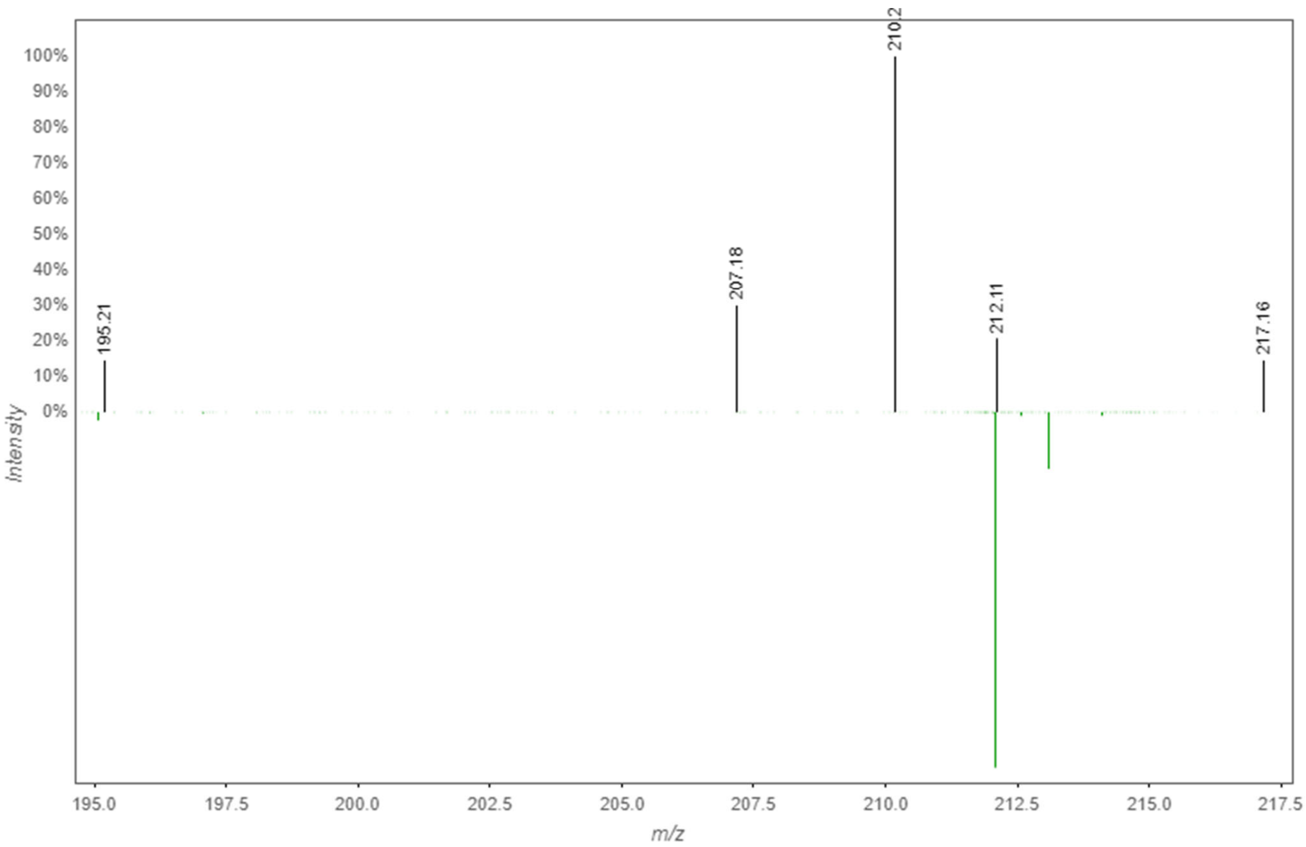

SI Figure 106. GNPS MSMS spectra of **342.33 m/z N6-phenyladenosine [M+H]+.**

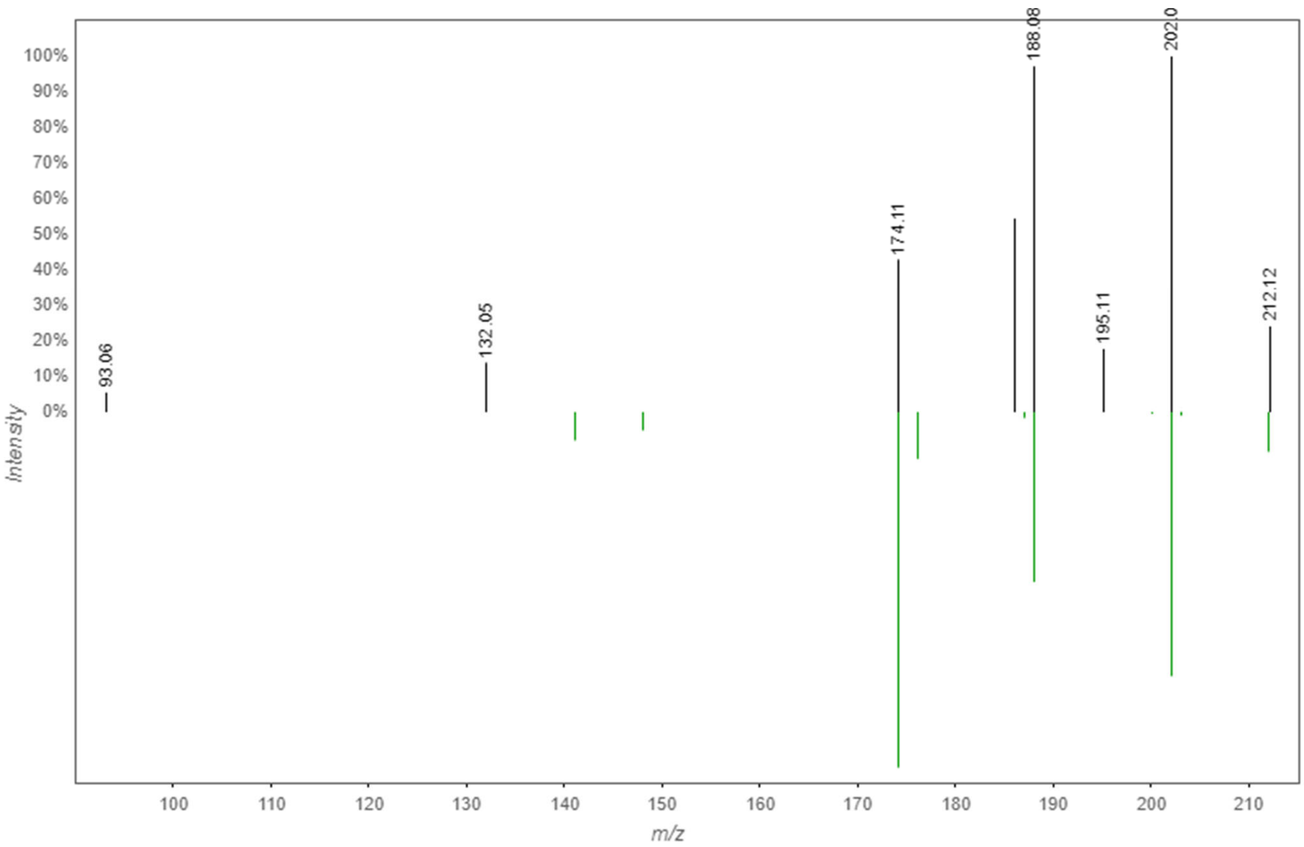

SI Figure 107. GNPS MSMS spectra of **314.32 m/z 3-(6-methoxy-3,4-dihydro-1H-pyrido[3,4-b]indol-2(9H)-yl)-1-methylpyrrolidine-2,5-dione [M+H]+.**

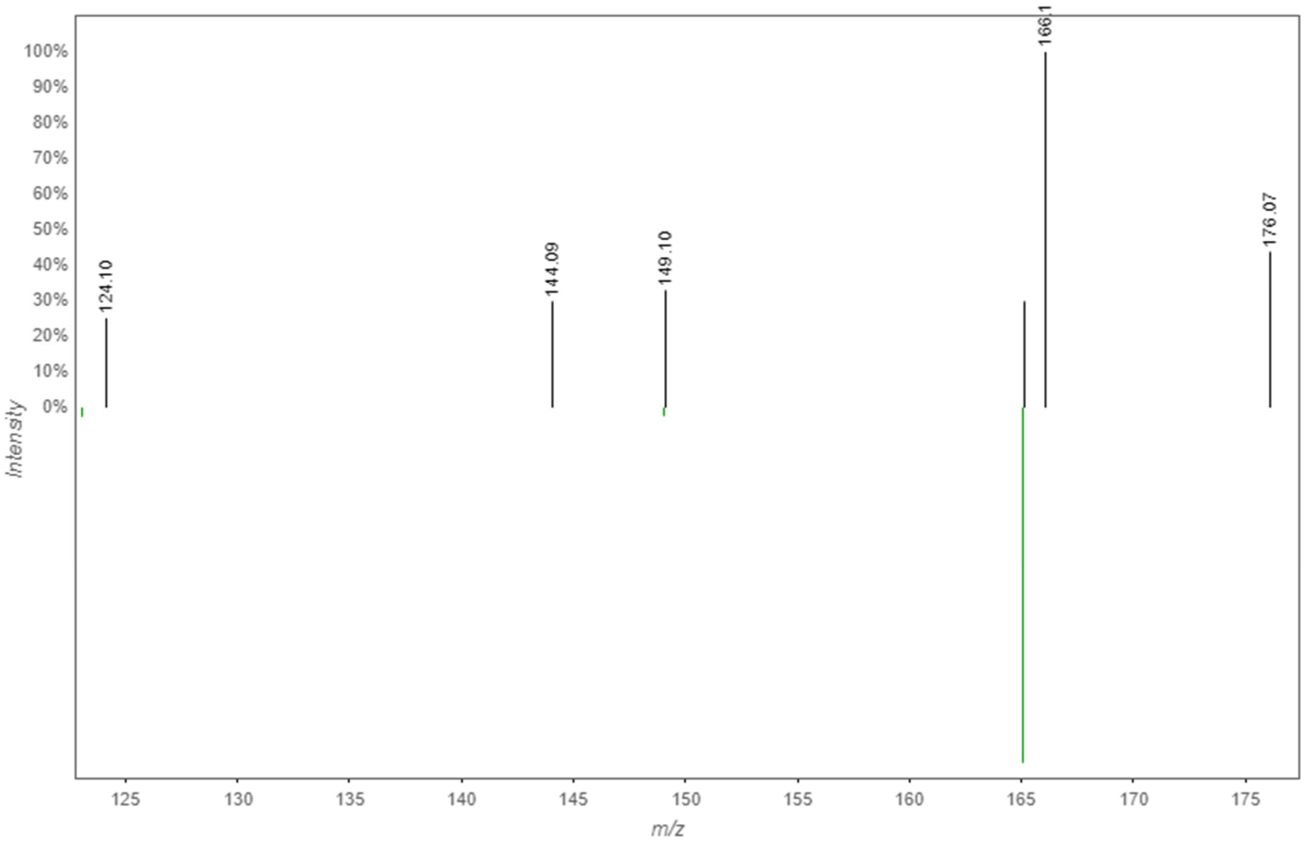

SI Figure 108. GNPS MSMS spectra of **328.42 m/z 4-Acetyl-3-hydroxy-5-methylphenyl beta-D-glucopyranoside [M+H]+.**

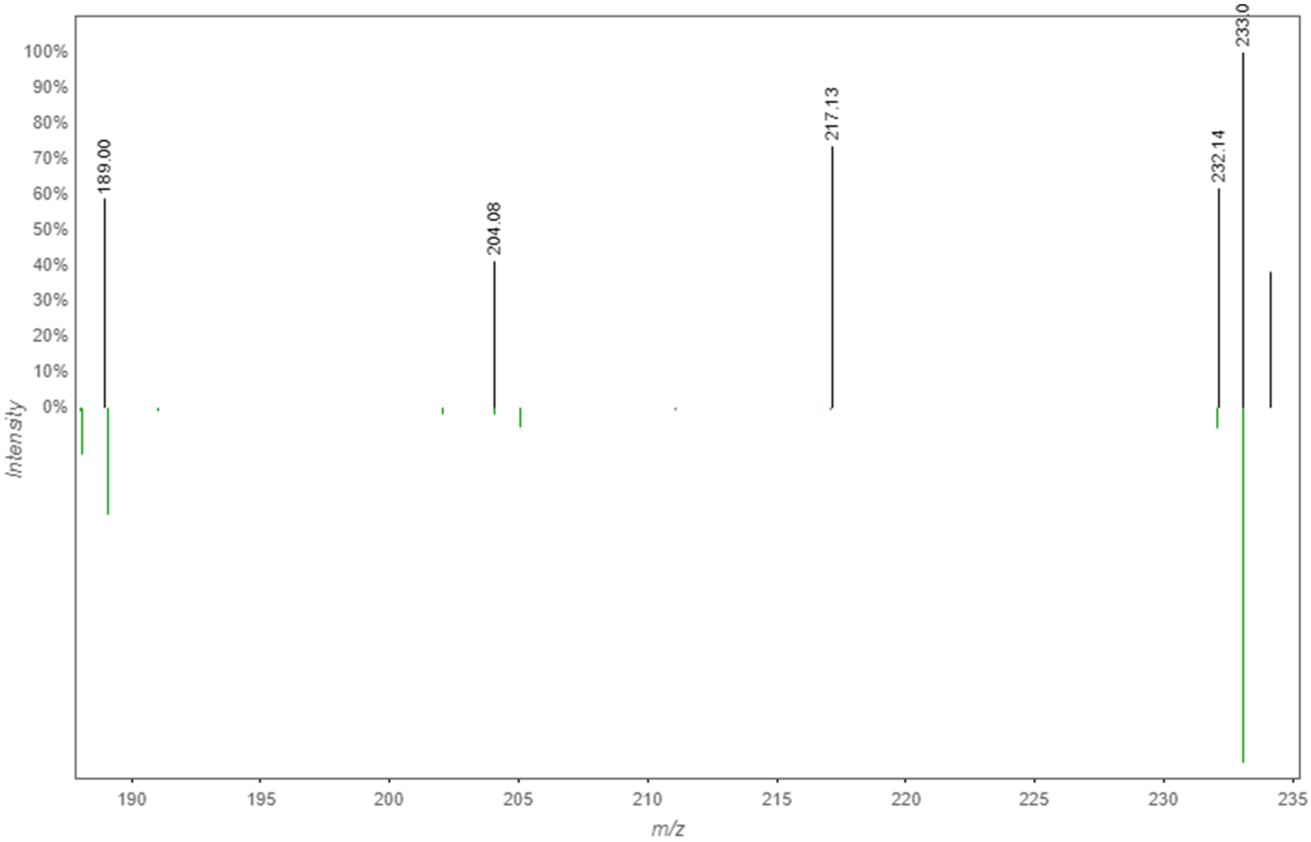

SI Figure 109. GNPS MSMS spectra of **274.31 m/z methylethyl (2E)-3-(3,5-dimethoxyphenyl)-2-cyanoprop-2-enoate [M+H]+.**

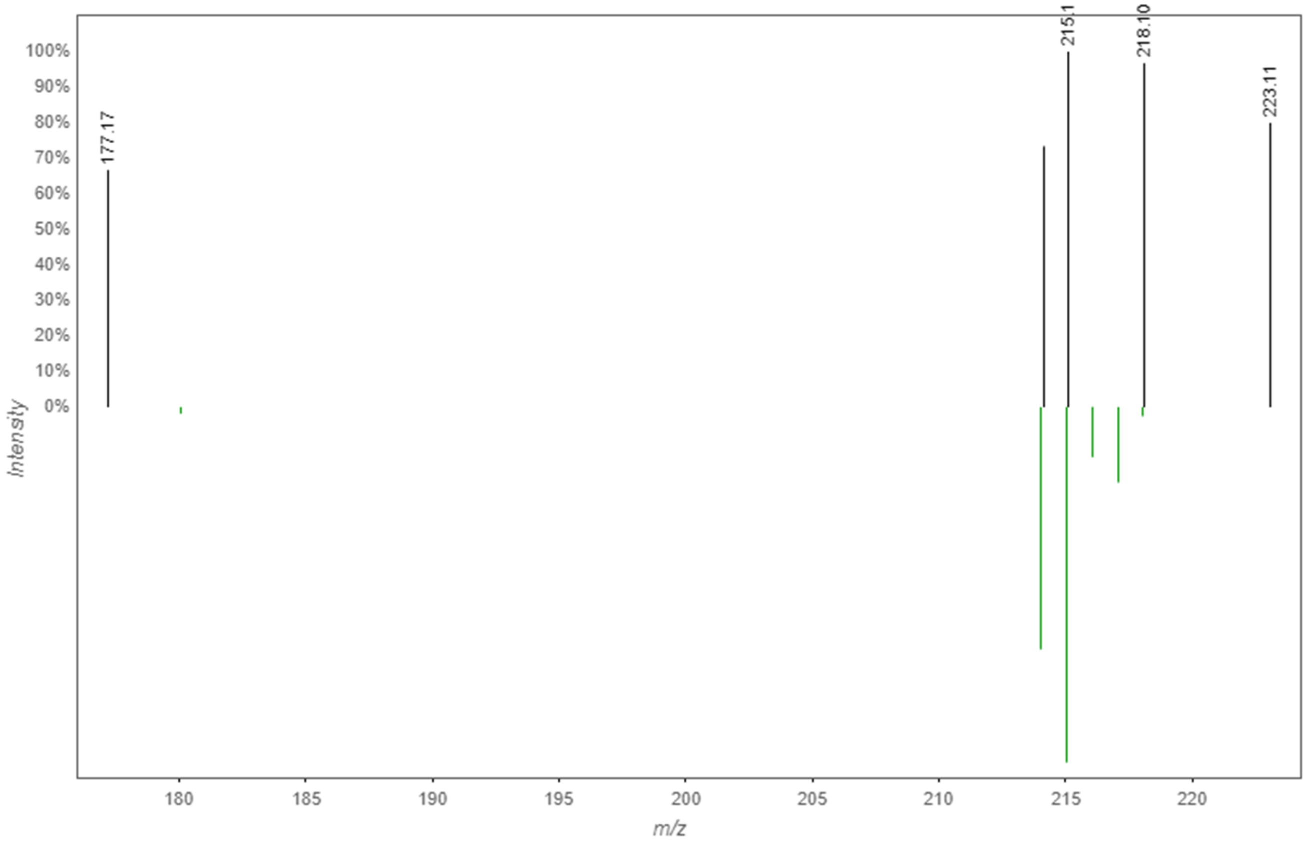

SI Figure 110. GNPS MSMS spectra of **297.42 m/z Diclofenac|2-[2-(2,6-dichloroanilino)phenyl]acetic acid [M+H]+.**

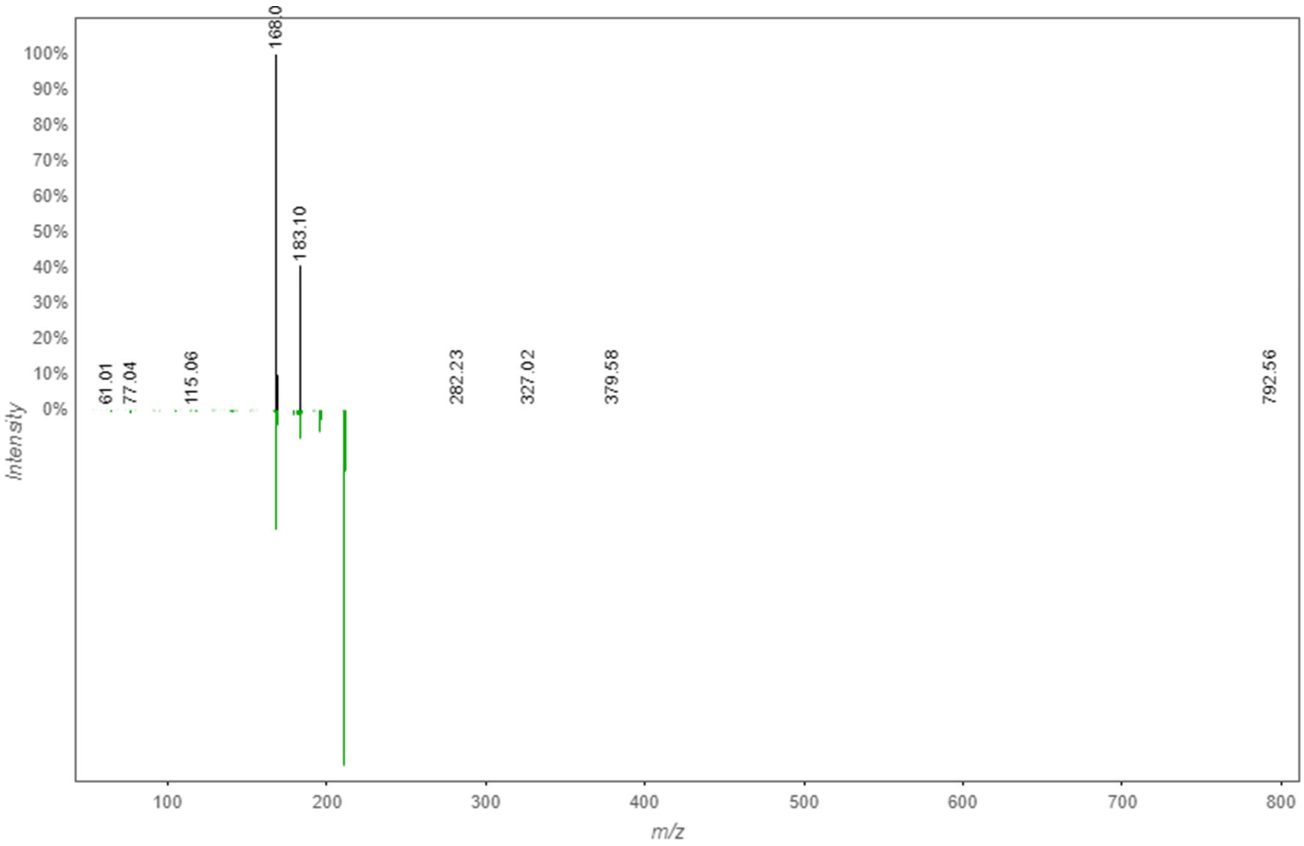

SI Figure 111. GNPS MSMS spectra of **Pyocyanin 211.18 m/z [M+H]+.**

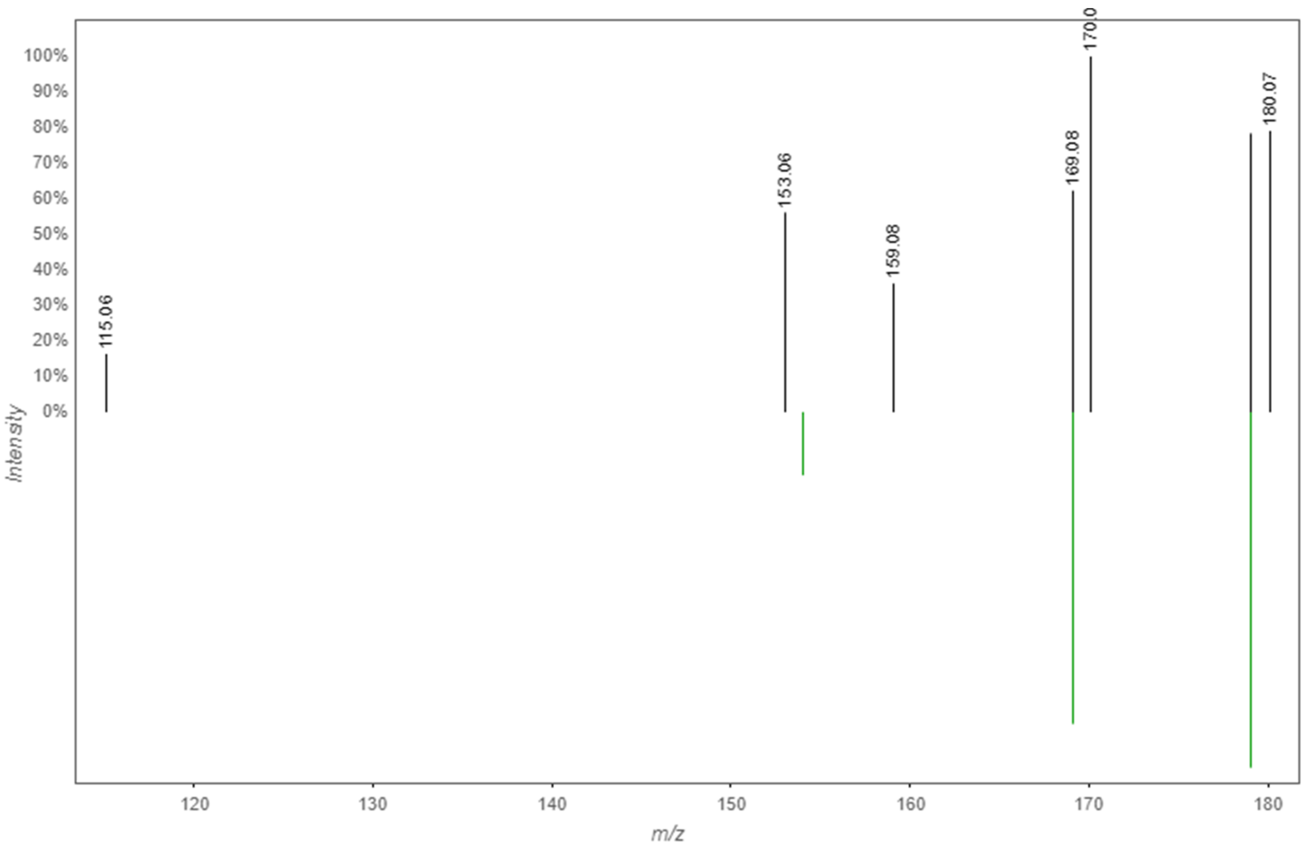

SI Figure 112. GNPS MSMS spectra of **1-hydroxyphenazine 198.18m/z [M+H]+.**

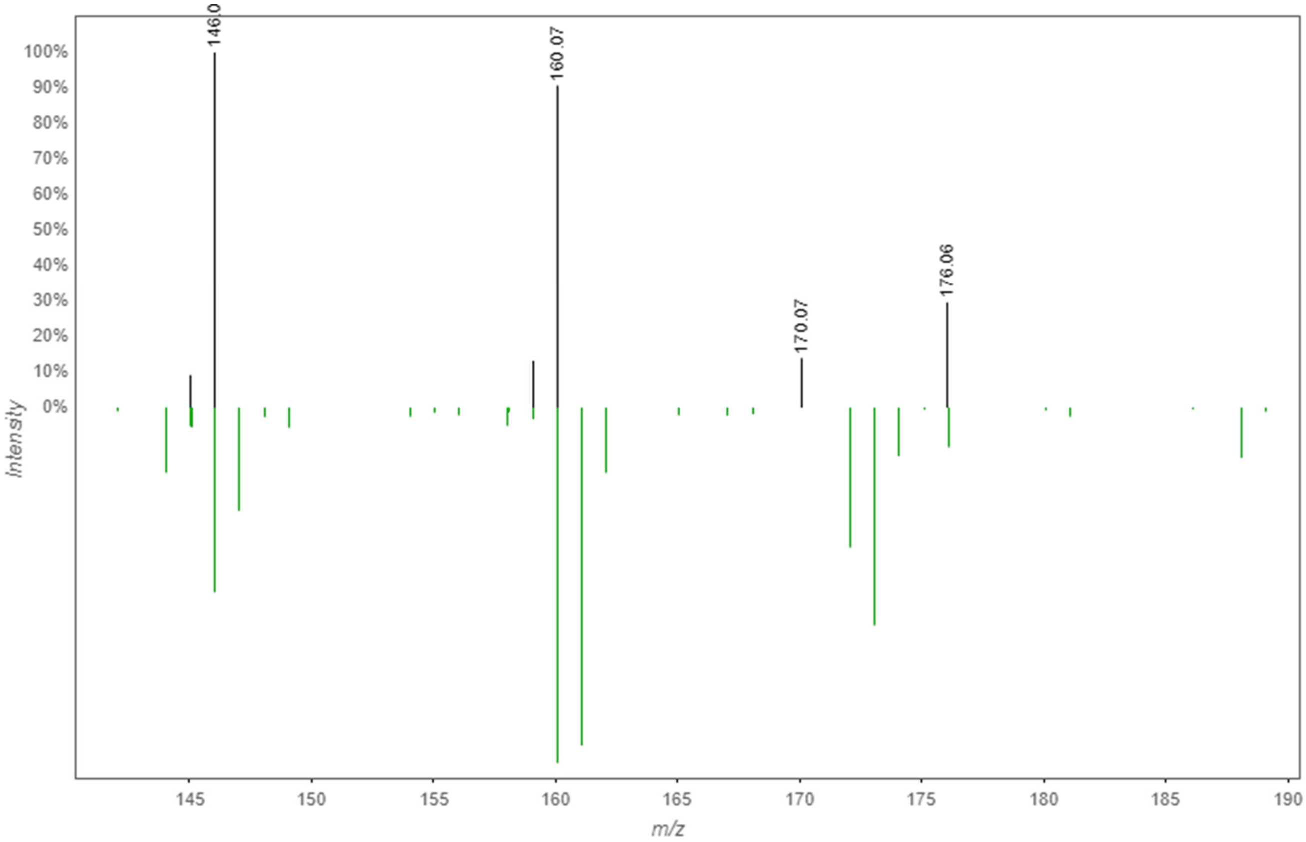

SI Figure 113. GNPS MSMS spectra of **6,7-dimethoxy-1-phenyl-3,4-dihydro-isoquinoline 288.37 m/z [M+H]+.**

MetaboAnalyst Analysis

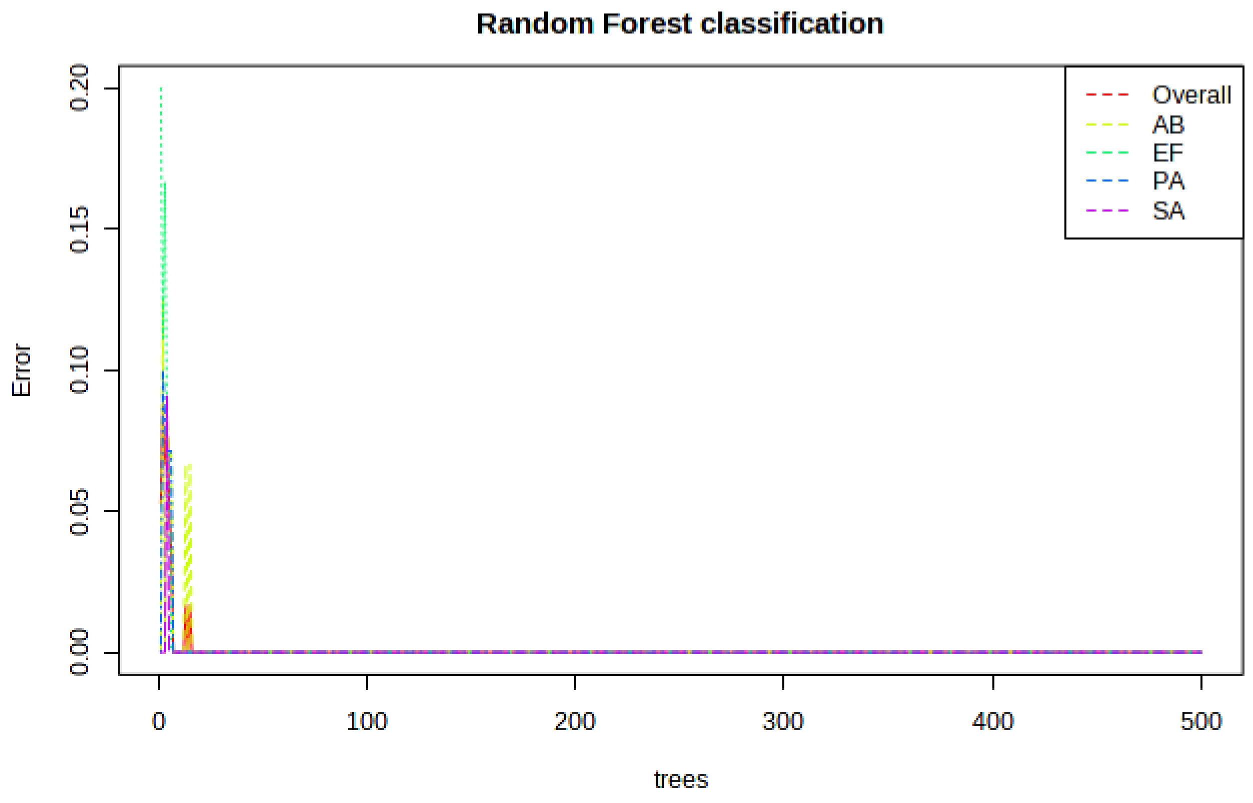

SI Figure 116. Random Forest: Classification. A Pareto-scaling was used to normalize the data before processing, and using the random forest classification option, we can see classification of species with less than 50 trees in the positive mode dataset.

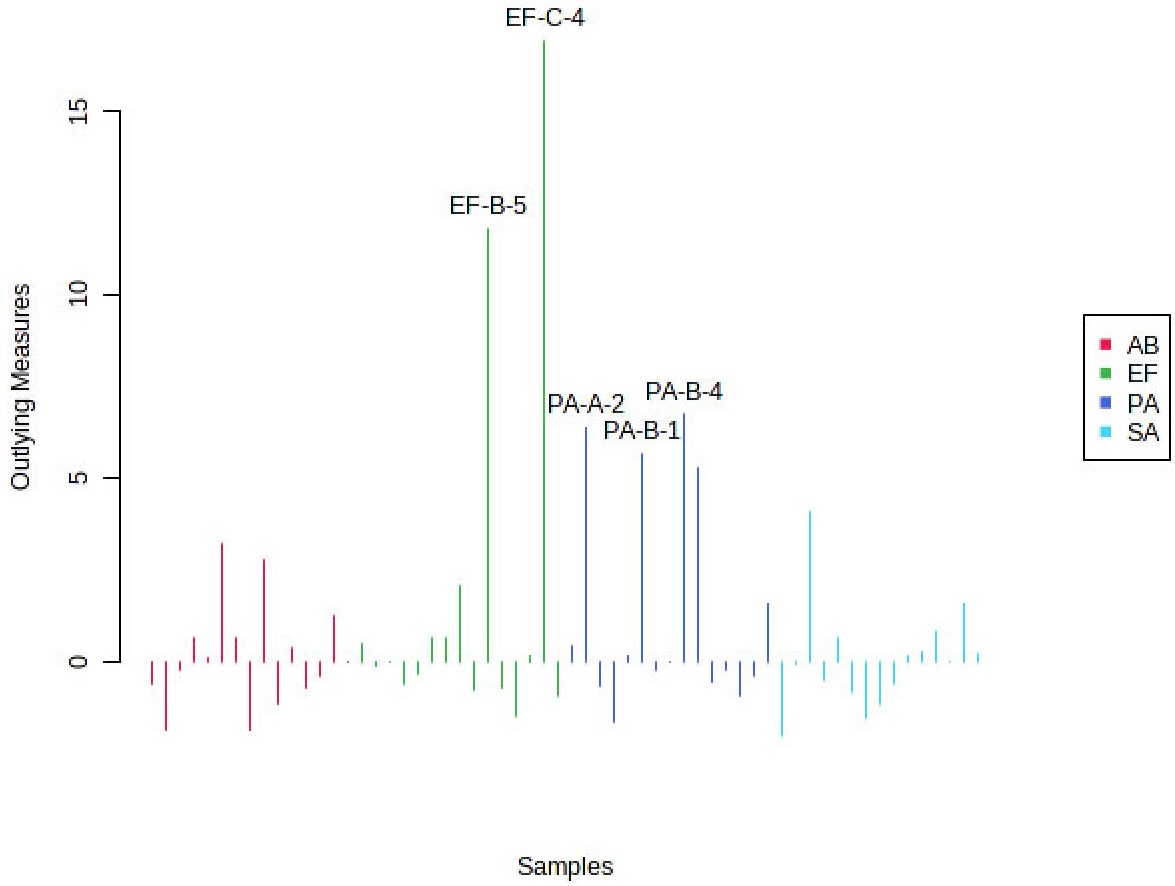

SI Figure 117. Random Forest: Variable Outlier Detection. The shortest path for random-tree depth can be explained by prominent outlying data points seen in species like *E. faecium* and *P. aeruginosa* in the positive mode dataset.

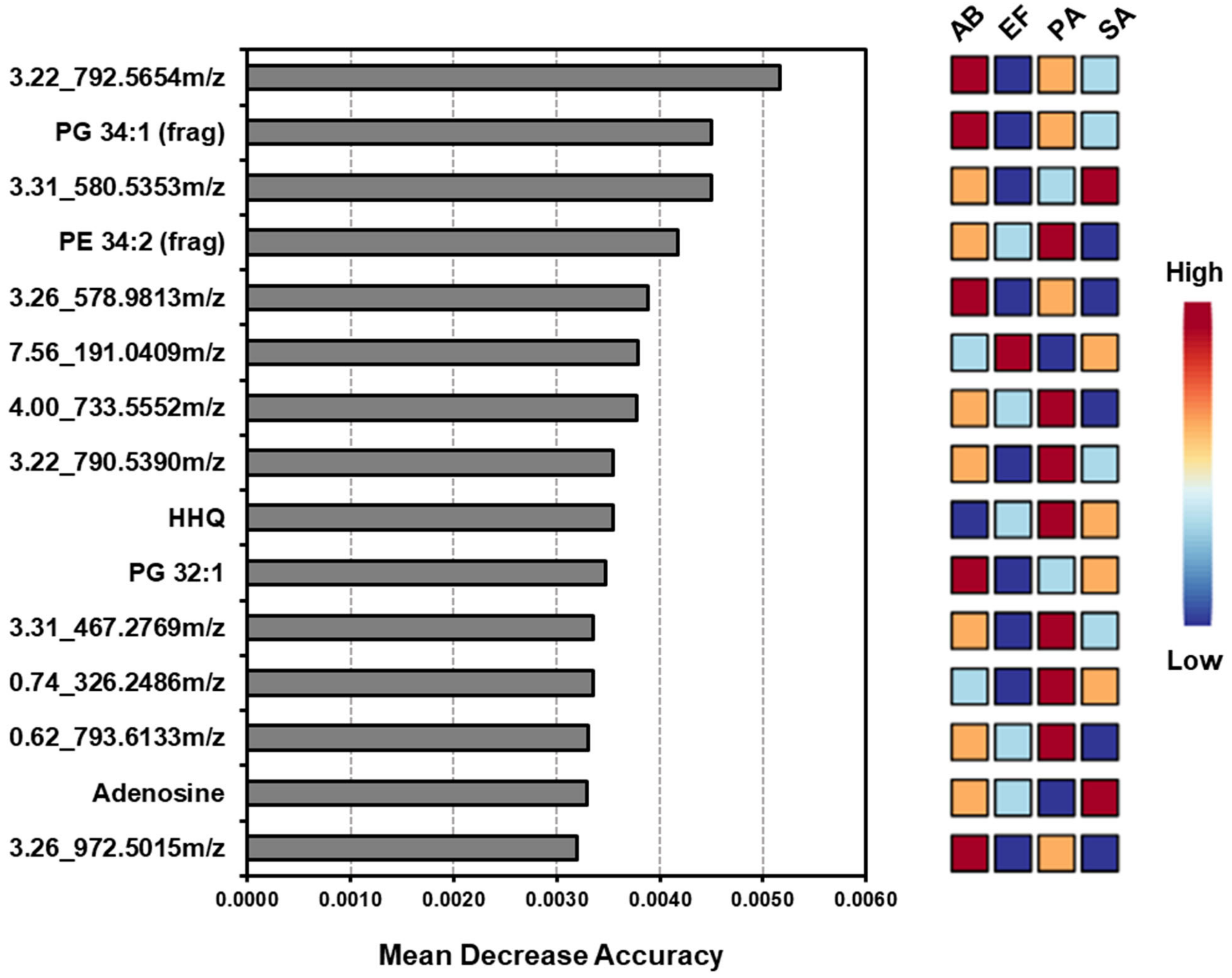

SI Figure 118. Random forest: Variable Importance Projection (VIP). The top 15 features that explain the most variance between species are showcased in the plot above. Some compound IDs have been annotated based on validated identifications in the positive mode dataset.

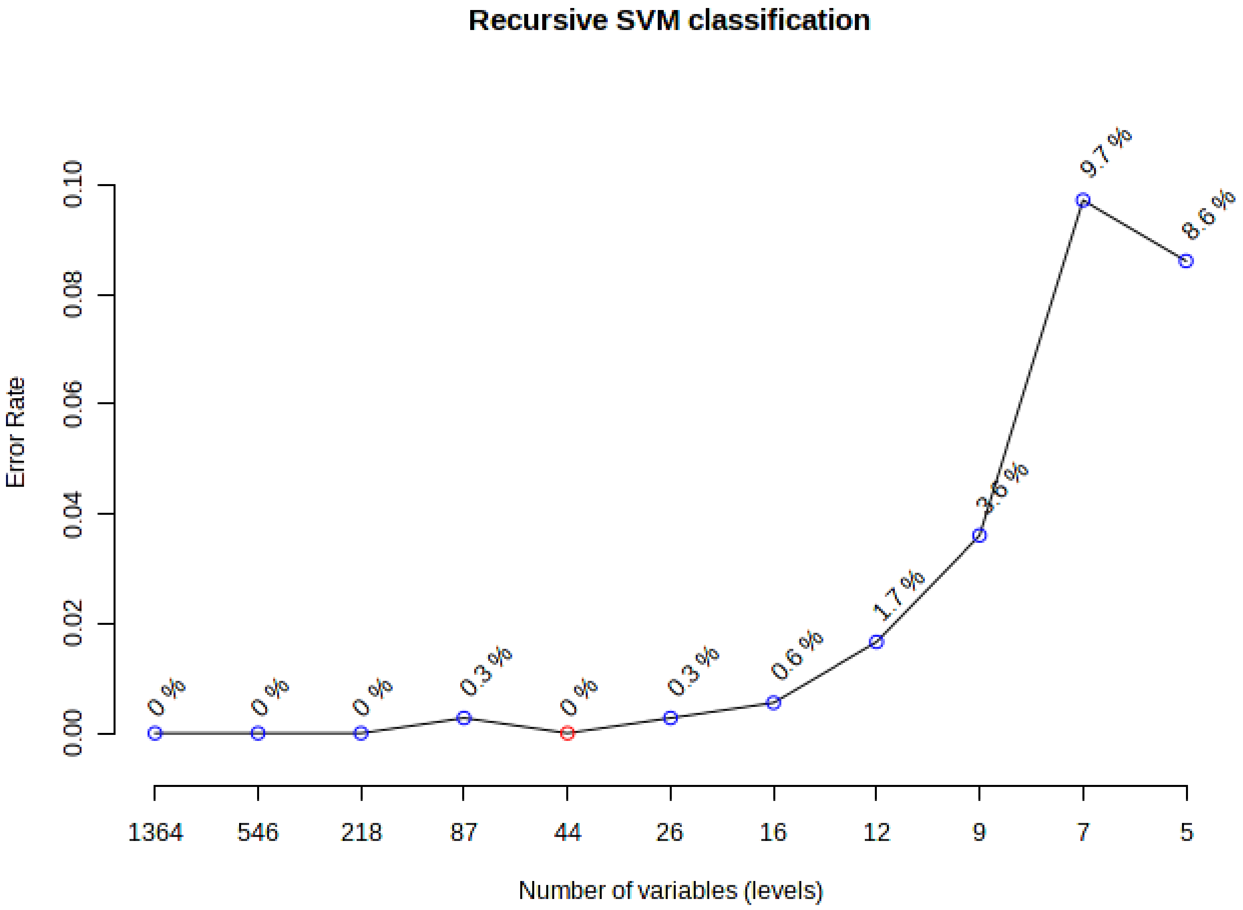

SI Figure 119. Support Vector Machine (SVM): Classification. SVM is linear kernel machine learning model for recursive classification using a cycle of features in subsets. Above we see (outlined in red) the number of variables that best classify Gram-negative versus Gram-positive in the positive mode dataset.

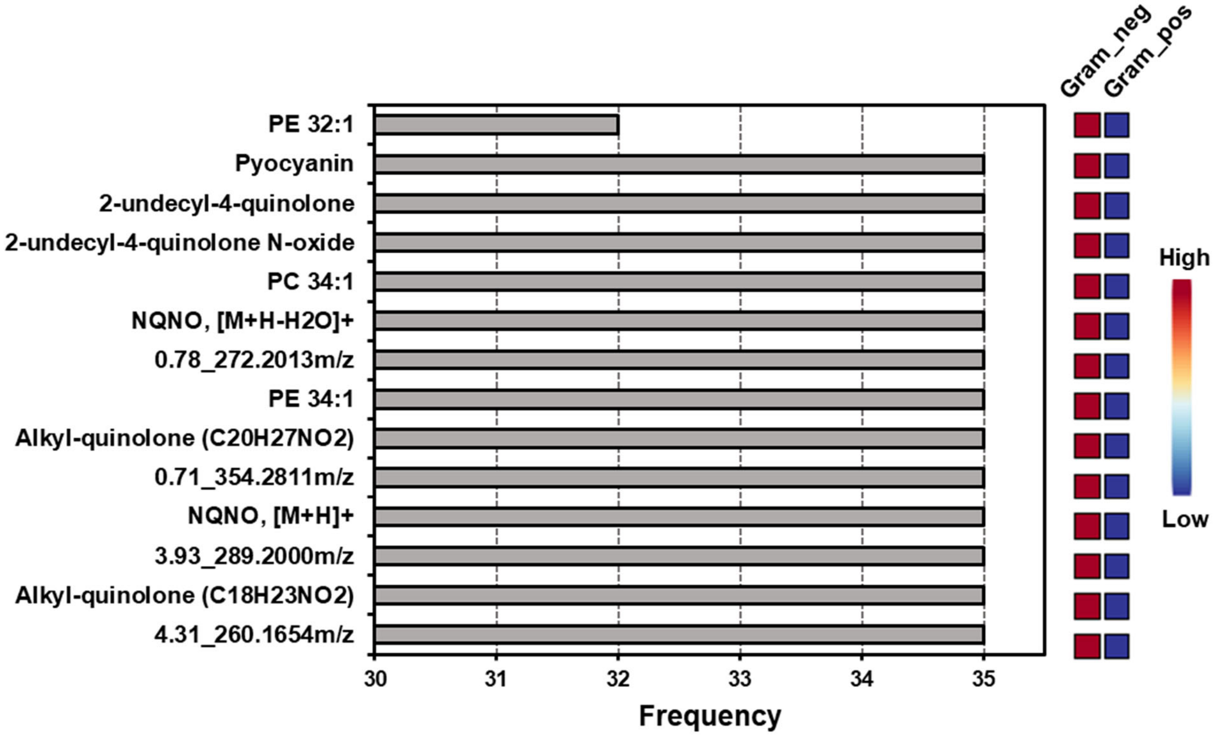

SI Figure 120. Support Vector Machine (SVM): Feature Frequency. These top 15 features are ranked based on their frequency of being selected as a best classifier based on their contribution that best describes the variability between Gram-negative and Gram-positive bacteria from the positive mode dataset.
